# Integrated Analyses Resolve Conflicts over Squamate Reptile Phylogeny and Reveal Unexpected Placements for Fossil Taxa

**DOI:** 10.1371/journal.pone.0118199

**Published:** 2015-03-24

**Authors:** Tod W. Reeder, Ted M. Townsend, Daniel G. Mulcahy, Brice P. Noonan, Perry L. Wood, Jack W. Sites, John J. Wiens

**Affiliations:** 1 Department of Biology, San Diego State University, San Diego, California, 92182, United States of America; 2 Laboratories of Analytical Biology, Smithsonian Institution, 10th & Constitution Aves. NW, Washington, D.C., 20560, United States of America; 3 Department of Biology, University of Mississippi, Box 1848, Mississippi, 38677, United States of America; 4 Department of Biology and Bean Life Science Museum, Brigham Young University, Provo, Utah, 84602, United States of America; 5 Department of Ecology and Evolutionary Biology, University of Arizona, Tucson, Arizona, 85721, United States of America; Penn State University, UNITED STATES

## Abstract

Squamate reptiles (lizards and snakes) are a pivotal group whose relationships have become increasingly controversial. Squamates include >9000 species, making them the second largest group of terrestrial vertebrates. They are important medicinally and as model systems for ecological and evolutionary research. However, studies of squamate biology are hindered by uncertainty over their relationships, and some consider squamate phylogeny unresolved, given recent conflicts between molecular and morphological results. To resolve these conflicts, we expand existing morphological and molecular datasets for squamates (691 morphological characters and 46 genes, for 161 living and 49 fossil taxa, including a new set of 81 morphological characters and adding two genes from published studies) and perform integrated analyses. Our results resolve higher-level relationships as indicated by molecular analyses, and reveal hidden morphological support for the molecular hypothesis (but not vice-versa). Furthermore, we find that integrating molecular, morphological, and paleontological data leads to surprising placements for two major fossil clades (Mosasauria and Polyglyphanodontia). These results further demonstrate the importance of combining fossil and molecular information, and the potential problems of estimating the placement of fossil taxa from morphological data alone. Thus, our results caution against estimating fossil relationships without considering relevant molecular data, and against placing fossils into molecular trees (e.g. for dating analyses) without considering the possible impact of molecular data on their placement.

## Introduction

Squamate reptiles (lizards and snakes) are an important and diverse group of terrestrial vertebrates, with >9,000 species [1]. Squamates are an especially significant group for humans because venomous squamates cause tens of thousands of deaths every year [2] and yet their venom toxins are a crucial resource for diverse medicines [3]. Squamates are also widely used as model systems for research in ecology and evolutionary biology, given their diverse ecologies, body forms, reproductive modes (e.g. viviparous and oviparous species), sexual systems (e.g. sexual and asexual species), and other characteristics [4–7]. However, studies of squamate biology are presently hampered by uncertainty over their phylogeny.

Higher-level squamate phylogeny is currently considered unresolved because of strong conflicts between hypotheses based on separate analyses of morphological and molecular datasets [8, 9]. Most attention has focused on the placement of iguanians (including iguanas, anoles, chameleons, dragons, and relatives), which are placed at the base of the squamate tree in morphological analyses, and in a clade (called Toxicofera) with snakes and anguimorphs (including monitor and alligator lizards, the Gila monster, and relatives) in molecular analyses. To date, the largest morphological dataset (in characters) included 189 squamate taxa (140 living and 49 fossil; plus 3 outgroup taxa) and 610 characters (~33% missing data; [8]; Gauthier et al., GEA hereafter). The largest molecular dataset (in terms of characters) included 161 living taxa (plus 10 outgroup taxa) for up to 44 nuclear protein-coding loci (33,717 base pairs/characters; ~20% missing data) ([10]; Wiens et al., WEA hereafter). Given the unresolved conflict between these two large datasets over the placement of Iguania, some authors have considered higher-level squamate relationships to be unresolved [9]. Some recent, prominent studies have considered the traditional, morphological tree only [11], ignoring the molecular hypothesis altogether.

Here, we perform integrated analyses to resolve this conflict and further elucidate the relationships of both living and fossil squamates. First, we generated an expanded morphological dataset (S1 Appendix) with taxon sampling largely matching that of GEA [8] for extant taxa, adding new data from 81 additional characters (primarily from squamation) to the mostly osteological dataset of GEA [8]. This is a 13% increase in characters (to 691), and the largest morphological dataset for squamates. Next, we expanded the molecular dataset of WEA [10] by including published sequences from two additional loci (nuclear *c-mos*; mitochondrial *ND2;* see S1 Table for GenBank numbers) for closely matched species yielding up to 46 protein-coding loci and 35,673 characters for each of 161 taxa. We then performed separate and combined analyses of each dataset using likelihood, Bayesian, and parsimony approaches, and evaluated the potential causes of conflict by examining trees from subsets of the molecular and morpohological data. Combined analyses included reweighting the molecular data such that genes were treated as equivalent to morphological characters. Note that for brevity and clarity, many of these ancillary analyses are explained and justified in the Results, rather than in the Methods.

## Materials and Methods

### Ethics statement

This study obtained new data only from non-living, previously preserved and accessioned museum specimens, and therefore no specific IACUC permission was needed.

### Maximum likelihood analyses

All maximum likelihood analyses were conducted using RAxML-HPC2 version 7.6.3 [12], with most conducted on the XSEDE (Extreme Science and Engineering Discovery Environment) at CIPRES (Cyberinfrastructure for Phylogenetic Research). Maximum likelihood analyses each used 1000 bootstrap replicates integrated with 200 searches for the optimal tree. Single gene analyses used the GTR + Γ substitution model (general time reversible with the gamma distribution of among-site rate variation) and were partitioned by codon positions. Analyses of the concatenated 46 loci data set used the GTR + Γ model and data were partitioned by both genes and codon positions (a total of 138 partitions; previous analyses including almost all loci strongly supported this partitioning scheme; [10]). We used the GTR model given that this is the only model supported in RAxML, and is also the most general model (other standard models are simply special cases of GTR; [13]). We used only the Γ parameter to account for among-site rate heterogeneity given that the large number of rate categories used during RAxML searches should account for the proportion of invariant sites (the I parameter used in many analyses), and following the recommendations of the developer of RAxML [12].

Analyses involving the morphological data (alone and in combination with the concatenated DNA data) were performed using the Mk model [14] with the Γ parameter added to account for rate heterogeneity among characters. Note that current versions of RAxML only allow for unordered analysis of morphological characters. However, our Bayesian analyses of the morphology alone (see below) were conducted both with all characters unordered and with selected characters ordered (all ordered analyses followed the ordering scheme recommended in [8] for those characters and for the new characters in S1 Appendix). The results of these analyses showed that ordering had little impact on the results (S1–S4 Figs.). Furthermore, these likelihood and ordered parsimony analyses both gave similar results regarding placement of mosasaurs (see below), further suggesting that ordering alone did not explain the differences between methods.

### Bayesian analyses

All Bayesian phylogenetic analyses were conducted using MrBayes version 3.2.2 [15]. Analyses of the concatenated 46 loci were partitioned by genes and codon positions, with gene-specific models selected using the AIC in jModelTest [16]. For the 44 loci from WEA (10), the same models were used. For the two additional protein-coding loci (c-mos and ND2), we found that the GTR + I + Γ was the best model for each codon position. As for the likelihood analyses, morphological data were analyzed using the Mk + Γ model. We performed morphology-only and combined analyses treating the morphological characters as either all unordered or else treating some as ordered (see above).

Given the computational burden of these large datasets (requiring ~10–30 days on the Smithsonian Institution’s Topaz cluster), we performed single analyses of 40–75 million generations each (depending on dataset), consisting of two independent runs with 4 chains per run. Also, to facilitate and speed-up the time to convergence onto the posterior distribution, Squamata was constrained to be monophyletic (monophyly of Squamata is consistently well-supported in previous analyses). Stationarity was assessed based on inspection of plots of likelihood over time, effective sample size (ESS) values (≥200 for lnL and other model parameters in Tracer; [17]), standard deviation of split frequencies (< ~0.05), and potential scale reduction factors (~1.0). The first 50% of the generations were conservatively deleted as burn-in.

### Parsimony analyses

Parsimony analyses were conducted using PAUP* 4.0b10 [18], with a heuristic search with 10,000 random-addition-sequence replicates, each with tree-bisection-reconnection (TBR) branch swapping, and retaining all shortest trees. Clade support was assessed using nonparametric bootstrapping [19], with 1000 pseudoreplicates. Each pseudoreplicate consisted of 20 random-addition-sequence replicates (with TBR branch swapping), holding and saving only one tree per replicate (although a given pseudoreplicate may ultimately save multiple equally parsimonious trees). All characters were weighted equally. Ordering of multi-state morphological characters was as described above. Note that in parsimony tree searches Squamata was again constrained to be monophyletic (following [8]). However, this constraint was not possible in the bootstrap analyses.

Some analyses of the morphological partitions (see below) proved to be extremely slow, given the large number of trees generated (apparently caused by the combination of few characters and many taxa). For these analyses, we reduced the number of replicate searches to 500 and retained only 10,000 equally parsimonious trees from each search.

### Rogue taxon identification

Placement of some fossil taxa was highly problematic. In general, certain taxa (called “rogue taxa” [20]) can dramatically reduce branch support throughout the tree (e.g. bootstrap values; [21]) due to their ambiguous placement. Thus, various methods have been developed to identify such taxa (e.g. [22]). Recently, Aberer et al. [23] developed an algorithm (RogueNaRok) that evaluates taxa in a set of trees (e.g. from bootstrapping) and identifies those taxa that have the largest impact on support values (i.e. pruning taxa which are ambiguously placed in the original pool of trees increases support values in the reduced consensus trees). This algorithm is implemented in the RogueNaRok Webservice (http://exelixis-lab.org/roguenarok.html). The specific optimality criterion attributed to each taxon (or set of taxa, called a “drop set”) is the relative bipartition information criterion (RBIC), but RogueNaRok also reports the “raw improvement” for each drop set. The raw improvement represents the fraction of overall improvement in support values (e.g. bootstrap values) across the tree when the taxa of a particular drop set are removed. We uploaded the RAxML-inferred bootstrap trees and optimal likelihood tree from the combined dataset that included the morphological and DNA data for all taxa in our study. Using three different drop sets (i.e. pruning a maximum of up to 1, 2, or 3 taxa; see S2–S4 Tables), RogueNaRok identified several taxa with optimal and near optimal RIBC values (i.e. highest improvements in support values in the pruned consensus trees), and among these taxa, the deletion of two (Sineoamphisbaenia and Huehuecuetzpalli) consistently generated the greatest improvements in support values, as evident in the raw improvement scores (e.g. for the drop set of 1, pruning *Huehuecuetzpalli* resulted in a total increase of 147% in summed bootstrap values across the tree, relative to support values in trees including this taxon; S2 Table). Across the different drop-set analyses, deletion of six additional taxa (Aciprion, AMNH FR 21444 [a fossil of uncertain affinity, known primarily by its museum specimen number; see ref. [8]], Eichstaettisaurus, Eupodophis, Hassiophis, and Pachyrhachis) resulted in varying improvements in support values. Upon examining the results from the combined-data likelihood analysis with all taxa included (S8 Fig.), we found that AMNH FR 21444, Eichstaettisaurus, Huehuecuetzpalli and Sineoamphisbaenia are ambiguously placed and do not clearly fall into any higher-level clades. Thus, these four taxa seem to largely explain the low bootstrap values for higher-level squamate relationships (S8 Fig.). In contrast, the three fossil snakes (Eupodophis, Hassiophis, and Pachyrachis) identified as rogues are strongly supported as a clade and are strongly placed within Alethinophidea. Therefore, the ambiguity associated with their placement is only within Alethinophidea (i.e. in drop set 3 there is a 143% improvement in summed bootstrap values when these taxa are pruned, but almost all improvement is within Alethinophidea). Likewise, Aciprion is strongly placed within Iguania. Given that our overall goal was to infer relationships among as many living and fossil squamate taxa as possible, we excluded only four rogue taxa (AMNH FR 21444, Eichstaettisaurus, Huehuecuetzpalli, and Sineoamphisbaenia) from subsequent combined-data analyses because their inclusion caused low bootstrap support across the tree (versus eroding support within smaller clades).

Our evaluations of rogue taxa were restricted to the likelihood analyses. Given the computational burden of performing Bayesian analyses on the combined data set (i.e. single analyses taking >30 days on a supercomputer), it was not feasible to perform similar analyses in a Bayesian context. However, we applied the results of the likelihood-based selection of rogue taxa to the Bayesian analyses. These analyses showed that some aspects of the Bayesian combined analyses were somewhat sensitive to the exclusion of rogue taxa (in terms of both topology and branch support), but these results were generally very sensitive overall (e.g. changes in character ordering for the morphological data impacted the results, even though such changes had little impact on the analyses of morphological data alone).

## Results

### Resolving higher-level squamate relationships

Results of the separate analyses of the expanded molecular and morphological datasets are largely congruent with those of the previously largest morphological and molecular analyses [8, 10]. Specifically, our morphological results (S1–S4 Figs.) also show strong support for basal placement of iguanians and monophyly of scleroglossans (i.e. all squamates excluding iguanians), but with the enigmatic fossil taxon *Huehuecuetzpalli mixtecus* as sister to the clade of iguanians and scleroglossans. However, there are also several differences relative to the preferred hypothesis of GEA [8] (their Fig. 3 vs. our S1–S4 Figs.), mostly involving branches that are weakly supported in both studies. The molecular trees are almost identical to those of WEA ([10]; our S5–S7 Figs.). Specifically, placement of iguanians with snakes and anguimorphs (the clade Toxicofera) is strongly supported, as in other recent molecular studies [24–29]. Most other higher-level relationships are strongly supported by the molecular data.

**Fig 1:**
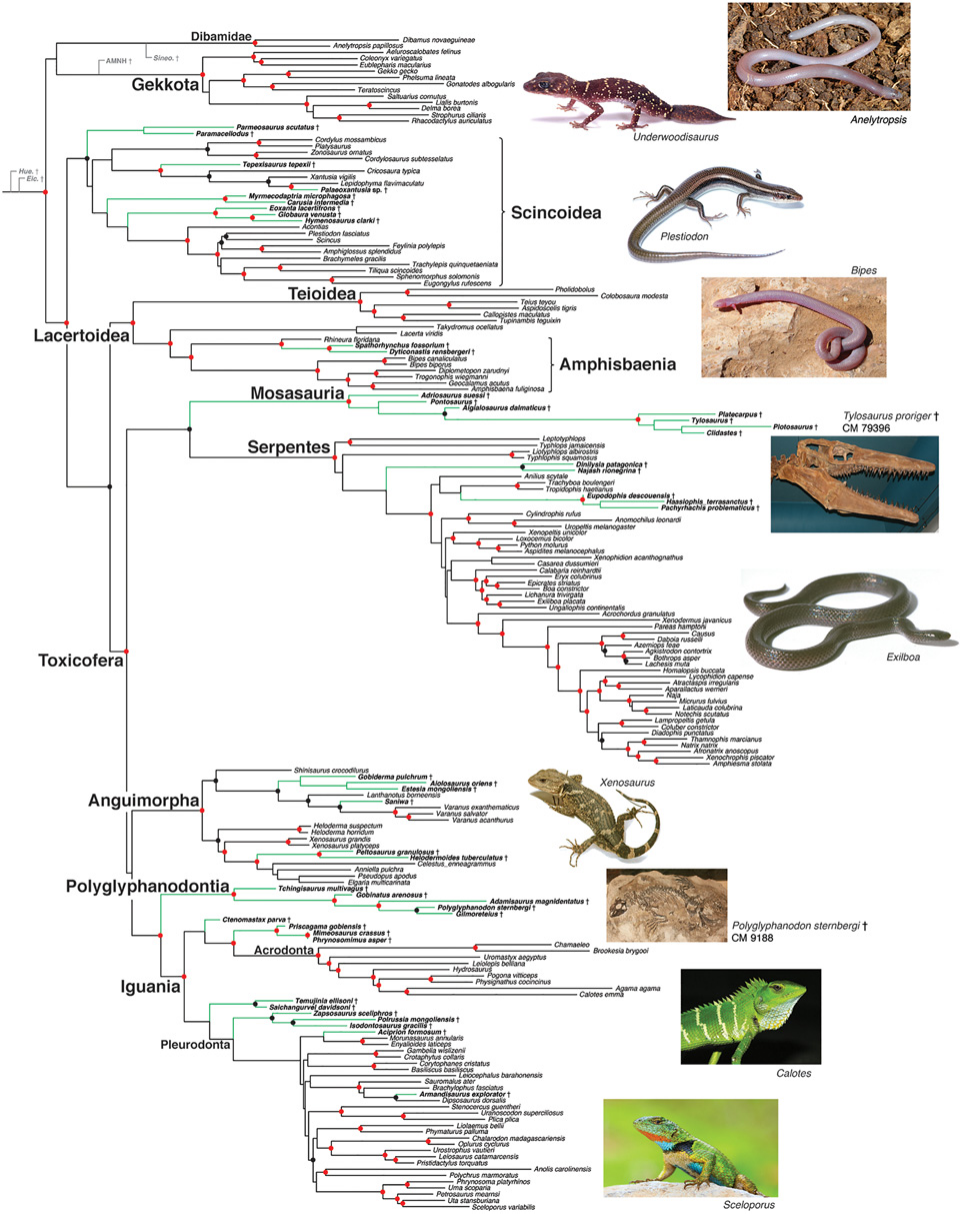
Estimated phylogeny of squamate reptiles from likelihood analysis of combined morphological and molecular data, after removal of four “rogue” fossil taxa (-ln*L* = 979285.16; see S8 Fig. for tree including all taxa). Red dots indicate clades with bootstrap values from 90–100%, black dots indicate values from 70–89% (values <70% not shown; for bootstrap values for all branches see S9 Fig.). Fossil taxa are indicated with “≪” and green branches. The four abbreviated fossil taxa in gray at the base of the phylogeny are the four rogue taxa (*Eichstaetisaurus*, *Huehuecuetzpalli*, *Sineoamphisbaenia*, AMNH FR 21444), shown in their phylogenetic positions as inferred in the combined analysis including all taxa (S8 Fig.). Photos include representatives of Dibamidae (*Anelytropsis*), Gekkota (Carphodactylidae: *Underwoodisaurus*), Scincoidea (Scincidae: *Plestiodon*), Amphisbaenia (Bipedidae: *Bipes*), Mosasauria (*Tylosaurus*), Serpentes (Boidae: *Exiliboa*), Anguimorpha (Xenosauridae: *Xenosaurus*), Polyglyphanodontia (*Polyglyphanodon*), Acrodonta (Agamidae: *Calotes*), and Pleurodonta (Phrynosomatidae: *Sceloporus*). See Acknowledgments for photo credits (except for *Anelytropsis* from T. M. Townsend).

Results of the combined-data (morphological-molecular) likelihood analyses support the relationships suggested by molecular data for the extant taxa (Fig. 1; S8, S9 Figs.), including the strongly supported placement of iguanians with snakes and anguimorphs. Some relationships amongst the deepest clades are weakly supported in combined analyses including all taxa. For example, relationships among Gekkota, Scincoidea, Lacertoidea, Anguimorpha, Iguania, and Serpentes all have bootstrap support values less than 50% (S8 Fig.). However, this weak support appears to be an artifact of the uncertain placement of four “rogue” fossil taxa (AMNH FR 21444 [see above], *Eichstaetisaurus*, *Huehuecuetzpalli*, *Sineoamphisbaenia*), identified using RogueNaRok [23]. When these four taxa are removed and the data are re-analyzed (Fig. 1), the estimated relationships are very similar to those in the initial analysis (excepting the weakly supported relationships among the major clades within Toxicofera), but most of the deepest clades become strongly supported (for detailed support values see S9 Fig.). For example, the clade containing all squamates above Gekkota + Dibamidae has a bootstrap support value of 92%, Lacertoidea (teiids, gymnophthalmids, lacertids, amphisbaenians) + Toxicofera (anguimorphs, iguanians, snakes) has 87%, and Toxicofera has 93%. Thus, these combined likelihood analyses yield an overall strongly supported tree for squamate relationships that integrates molecular and morphological data and living and fossil taxa (Fig. 1). This tree overwhelmingly supports the relationships suggested by the molecular data for the living taxa. Bayesian and parsimony analyses of the combined data give similar results (S10–S15 Figs.).

### Reweighting the molecular data in the combined analysis

The combined-data analysis should provide the best hypothesis of squamate relationships (Fig. 1), by incorporating the largest amount of relevant character data. However, some authors have expressed concern over combined analyses of squamates because of the greater number of molecular characters [8]. Nevertheless, approaches that downweight molecular characters in combined analyses have been widely rejected by phylogeneticists. For example, combined analyses of molecular and morphological data are common in the literature, but few (if any) utilize such a weighting approach. In fact, our previous combined, unweighted analyses of squamate relationships [27] showed that adding 363 morphological characters could overturn strongly-supported relationships among living taxa based on 15,794 molecular characters (i.e. strong support for non-monophyly of amphisbaenians from molecular data, but strong support for their monophyly in the combined-data tree). This same issue occurs (to a lesser extent) in the present analysis, with parsimony analysis of the molecular data supporting non-monophyly of amphisbaenians (S7 Fig.), whereas likelihood and Bayesian molecular analyses (S5, S6 Figs.) and all combined analyses (S8–S15 Figs.) support amphisbaenian monophyly. Thus, the idea that the sheer number of molecular characters always predetermines the outcome of a combined molecular-morphological analysis is demonstrably untrue.

Despite the general consensus that downweighting molecular characters in combined analysis is problematic, we nevertheless performed such a weighted, combined analysis that very strongly favored the morphological data. We simply coded each of 44 genes as a binary character, with the derived state present in Iguania and also in the sister group to Iguania inferred by that gene (we excluded PTGER4 and ND2, in which overall rooting is problematic; see S5 Table). However, under this coding, genes that support either Anguimorpha or Serpentes (but not both) as sister to Iguania are not counted as supporting monophyly of Toxicofera. Therefore, we added 19 binary characters to represent these genes that support monophyly of Toxicofera (in addition to supporting Anguimorpha or Serpentes as sister to Iguania; S5 Table). This coding strategy is equivalent to treating 19 of the 44 characters as ordered multi-state characters. We then analyzed the combined matrix using Bayesian, likelihood, and parsimony methods.

Remakably, despite the overwhelming majority of morphological characters in the combined matrix (691 vs. 63 molecular characters), these analyses nevertheless support monophyly of Toxicofera (S16–S27 Figs.) rather than the basal placement of Iguania suggested by morphology alone. However, in some analyses the support was relatively weak. Intriguingly, the support for this clade increases markedly when the fossil taxa are removed, and when 12 limb-reduced, non-snake burrowing taxa are removed (amphisbaenians, the anguid/anniellid Anniella, the dibamids Anelytropsis and Dibamus, and the scincids Acontias and Feylinia; see section “d” below). We emphasize that even though these analyses support Toxicofera, they are very strongly biased against the molecular data, since each of the 46 genes actually contains hundreds of informative characters (e.g. mean parsimony-informative characters per gene = 463.8; range = 210–1161; S6 Table) and the support of these characters for the overall higher-level phylogeny should also influence the support for Toxicofera. Nevertheless, even given this extremely biased weighting that favors the morphological data over the molecular data, the basal placement of Iguania is not supported by the combined data.

### Is there no morphological support for the molecular tree?

Several additional lines of evidence support these combined-data relationships and emphasize the ambiguity of the morphological data. Previous authors [8, 9] have asserted that no unambiguously optimized morphological synapomorphies support the clade of snakes, anguimorphs, and iguanians (Toxicofera). However, mapping morphological traits onto the combined-data likelihood tree using parsimony (in MacClade; [30]) reveals that monophyly of Toxicofera is actually supported by six unambiguous morphological synapomorphies, although none are unique (character numbers 360: subdental shelf of dentary; 470: position of caudal autotomic septa; 500: fenestration of clavicle; 501: proximal expansion of clavicle; 508: length of anterior process of interclavicle; 619: number of internasal scales; five are from GEA; numbering follows GEA for characters 1–610 and S1 Appendix or 611–691). The number of unambiguous morphological synapomorphies supporting Toxicofera in the combined-data tree is similar to that supporting monophyly of Scleroglossa in the morphology-based tree (seven: characters 39: depth of frontal subolfactory process; 83: postorbital-jugal suture, 114: relative length of facial process of maxilla; 205: septomaxilla medial flange; 305: alar process of prootic; 455: number of presacral vertebrae; 555: notching of distal epiphysis of tibia). Note that we follow here the traditional definition of Scleroglossa (i.e. all non-iguanian squamates), including Polyglyphanodontia and Mosasauria, despite some recent ambiguity [8]. Among the seven synapomorphies supporting Iguania near the squamate root, only one (character 83) is unique to Scleroglossa (no homoplasy). However, the apparent uniqueness of this character is contingent on the morphological tree being correct (if it is not, then this character also shows homoplasy). In summary, examination of the combined data and combined-data tree reveals that the morphological data contain hidden support for this key aspect of the molecular hypothesis (i.e. monophyly of Toxicofera), and the quality and quantity of this support is similar to the quality and quantity of the morphological support for the morphological hypothesis (i.e. monophyly of Scleroglossa).

### Are the morphological data misleading?

The idea that the morphological characters are generally reliable indicators of higher-level squamate phylogeny is strongly undermined by the phylogenetic pattern associated with burrowing, limb-reduced taxa in the morphology-only trees. Specifically, our analyses of the morphological data alone place snakes, amphisbaenians, dibamids, and the anguimorph *Anniella* together in a clade nested inside the family Scincidae (S1–S4 Figs.). This clade of burrowing taxa is also present (in some form) in other recent morphological analyses [8, 31]. These trees therefore render the long-standing family Scincidae as paraphyletic (Fig. 3 of reference [8]; see also [31]), and render the traditionally recognized clade Anguimorpha as polyphyletic (i.e. the anguimorph *Anniella* is placed with other burrowing taxa rather than with non-burrowing anguimorphs in the families Anguidae, Helodermatidae, Lanthanotidae, Shinisauridae, Varanidae, and Xenosauridae). Thus, although not widely appreciated, recent morphology-based trees are actually very strongly at odds with traditional hypotheses and classifications based on morphology, and appear to have been misled by false phylogenetic signal associated with morphological convergence in distantly related burrowing taxa (see also [27]). In contrast, both Scincidae and Anguimorpha are strongly supported as monophyletic in our molecular and combined-data analyses (e.g. Fig. 1).

There may also be misleading signal in the morphological data related to the distinctive feeding behavior (and related morphological traits) of iguanians. Importantly, this behavior is also present in *Sphenodon* (Rhyncocephalia), the living sister-group to squamates [24]. This pattern of trait distribution may help explain why iguanians are placed near the squamate root by morphological data alone [24]. Specifically, iguanians and Sphenodon share lingual prey prehension, as opposed to the jaw prehension used by most other lizards and amniotes [24]. Lingual prey prehension is largely absent in the living sister group to Lepidosauria (Archosauria, including turtles, crocodilians, and birds), suggesting that lingual prey prehension is potentially convergent between Iguania and Sphenodon rather than necessarily primitive for Lepidosauria or Squamata [24]. Vitt et al. [32] summarized many additional differences between Iguania and Scleroglossa (all other non-iguanian lizards) that might also be related to these differences in feeding modes, including: (a) a dramatic difference in diet, including a higher percentage of ants and beetles in iguanian diets, (b) greater use of vomerolfaction when foraging in most scleroglossans (as opposed to visual foraging in iguanians), (c) more flexible skull and jaws in scleroglossans, and (d) more frequent use of elevated perches by iguanians versus use of terrestrial microhabitats by most scleroglossans (except gekkotans). These differences could help explain widespread convergence in the skull, postcranial skeleton, tongue, and other anatomical systems in iguanians and rhyncocephalians.

We also note that GEA [8] assessed the rooting of their tree only with a single outgroup (Rhyncocephalia, including Sphenodon), instead of the standard use of multiple outgroups. Thus, character states that are convergent between iguanians and rhyncocephalians would be incorrectly interpreted as being primitive in rhyncocephalians and reversals in Iguania (given the combined-data placement of Iguania). In contrast, the molecular and combined-data trees are rooted using multiple outgroups including rhyncocephalians, turtles, crocodilians, birds, and mammals. The hypothesis that the molecular tree requires multiple reversals in morphological characters in Iguania [8, 9] has therefore not been adequately tested, since GEA did not perform a standard outgroup analysis. We did not test this ourselves by collecting morphological data for additional outgroup taxa, given that the morphological data seem to be strongly misleading, regardless of how many outgroups are included.

We also performed analyses of subsets of the morphological data, to evaluate whether support for the basal placement of iguanians is widespread among character partitions (as would be expected if these were the true relationships) or confined to a subset of characters (as might be expected if this were an artifact of convergence, non-independence and misleading signal). However, we caution that this approach will identify the dominate signal in each partition, and individual characters could still support other patterns. We divided the combined morphological data into six subsets (cranial characters: 1–354; characters related to the jaws, teeth, and hyobranchial apparatus: 355–453; characters related to the vertebral column: 454–478; other postcranial osteological characters, mostly related to the limbs and limb girdles: 479–569; miscellaneous morphological characters, including morphology of the osteoderms, scleral ossicles and tongue, 570–610; characters of squamation and external morphology: 611–691). We acknowledge that there are other ways that these characters could be divided, but dividing them into smaller subsets might make the subsets less phylogenetically informative, and combining them into larger subsets might group potentially independent sets of characters.

Analyzing these six subdivisions of the morphological data with likelihood, Bayesian, and parsimony analyses, we found that only the cranial subset (the largest subset) unambiguously supported the basal placement of Iguania (S28–S71 Figs.). In other subsets, relevant relationships were often unresolved, Iguania was non-monophyletic (e.g. Acrodonta sister to other squamates in some analyses of jaw characters), or other groups were supported as sister to all other squamates (e.g. Gekkota by vertebral characters). These results are consistent with the idea that the basal placement of Iguania is not a true historical signal that has left a strong imprint across most subsets of morphological characters. On the other hand, these morphological subsets do not support monophyly of Toxicofera either. However, Bayesian and likelihood analyses show that four of the six subsets are strongly influenced by the widespread morphological convergence associated with burrowing taxa (cranial, vertebral, postcranial, and scalation subsets), placing seemingly unrelated burrowing taxa (dibamids, amphisbaenians, and some anguimorphs and skinks) in a single clade with snakes, a pattern strongly inconsistent with monophyly of Toxicofera (parsimony analyses are more ambiguous given the poorly resolved trees, but the cranial data clearly show this pattern). Interestingly, this pattern contrasts with our initial expectation that misleading signal associated with convergence should not be widespread across character systems.

In a similar vein, we performed approximately unbiased tests [33] of the morphological partitions using maximum likelihood, focusing on the extant taxa (S2 Appendix). These tests evaluate whether a given dataset significantly rejects a given clade [33]. We found (Table 1) that none of the six partitions significantly rejects monophyly of Scleroglossa (and thus, basal placement of Iguania). This is not surprising, given that the morphological data collectively support this relationship. However, the scalation data do reject this clade if the 12 burrowing, limb-reduced taxa (2 dibamids, 7 amphisbaenians, Anniella [Anguimorpha], and Acontias and Feylinia [Scincidae]) are eliminated. Conversely, only 3 of 6 partitions significantly reject monophyly of Toxicofera (Iguania, Anguimorpha, Serpentes), and only 2 do if the 12 burrowing taxa are eliminated (Table 1). These results further illustrate that support for the morphological placement of Iguania is mixed among morphological partitions, and potentially influenced by convergence associated with the burrowing taxa.

**Table 1:** Summary of results of the approximately unbiased test for the morphological data (complete and partitions) for extant squamates.

Dataset	Scleroglossa monophyly—All extant taxa	Scleroglossa monophyly—Burrowing taxa excluded	Toxicofera monophyly—All extant taxa	Toxicofera monophyly—Burrowing taxa excluded
All morphology	NA	NA	***P* = 0.016**	***P* = 0.002**
Cranial only	*P* = 0.412	*P* = 0.500	***P* = 0.040**	***P* = 0.039**
Jaw only	*P* = 0.310	*P* = 0.342	*P* = 0.301	*P* = 0.142
Misc. only	*P* = 0.327	*P* = 0.363	*P* = 0.243	*P* = 0.338
Post-cranial only	*P* = 0.217	*P* = 0.265	***P* = 0.022**	*P* = 0.066
Scalation only	*P* = 0.166	***P* = 0.037**	*P* = 0.122	*P* = 0.062
Vertebral only	*P* = 0.185	*P* = 0.274	***P* = 0.017**	***P* = 0.020**

Significant results (*P*<0.05) are boldfaced), indicating that the dataset rejects the phylogenetic hypothesis listed in that column.

### Are the molecular data misleading?

Potential explanations for molecular-morphological conflict involving the incorrect placement of Iguania by the molecular data seem highly unlikely. Most importantly, the molecular and combined-data matrices include 46 potentially independent loci. Given this, explanations such as molecular convergence (e.g. [34]) are unlikely across many loci with many different functions. Furthermore, we found that none of the 46 separately analyzed likelihood-estimated gene trees support basal placement of Iguania (S5 Table). Although there is incongruence among these estimated gene trees, previous analyses including 96% of these loci show that this incongruence is strongly associated with short branches in the concatenated tree [10]. Thus, incongruence in the molecular data appears to be explained primarily by incomplete lineage sorting (deep coalescence [10]) or by branches that are too short to accumulate sufficient mutations (a problem exacerbated by shorter sequences per gene, see below), and not by hidden signal for the basal placement of Iguania as suggested by morphology.

No genes support the basal placement of Iguania, but not all genes support the exact placement of Iguania suggested by the combined-data tree. Nevertheless, a majority of them do. Overall, 29 of 46 genes support monophyly of Toxicofera, whereas 34 of 46 support snakes and/or anguimorphs as sister to Iguania (S5 Table). Eight of 46 genes support snakes and/or anguimorphs as sister to Iguania but with additional groups in the same clade (e.g. Lacertoidea), such that monophyly of Toxicofera is not supported. In summary, 42 of 46 genes support snakes and/or anguimorphs as sister to Iguania, with or without additional groups. Among the genes that did not show the general relationships found in the combined-data tree, two genes had problematic rooting. PTGER4 placed mammals within Iguania but placed other non-squamate outgroups near Gekkota (as in the combined-data tree). The sole mitochondrial gene (ND2) also had difficulty rooting the tree, placing some archosaurs inside of squamates. Furthermore, six genes did not support monophyly of Iguania (but some genes that do not support iguanian monophyly can still support monophyly of Toxicofera or other relevant clades).

We found a simple explanation for why some genes fail to support the placement of Iguania favored by most other genes. Among the 46 genes, the 29 genes that support monophyly of Toxicofera tend to be longer (i.e. more characters) than the 17 that do not (mean length = 824 vs. 686; data in S5, S6 Tables). This trend is not fully significant (P = 0.08; unpaired t-tests), but becomes significant if the sole mitochondrial gene (ND2; not supporting Toxicofera) is removed (P = 0.04). This pattern strongly suggests that the failure of particular genes to support monophyly of Toxicofera occurs because too few characters were sampled for these genes, rather than misleading signal.

### Placement of fossil taxa

Our combined analyses also offer new insights into the placement of key fossil taxa (Fig. 1). For example, placement of the mosasaurs, a group of giant marine lizards that dominated Cretaceous oceans [35], has been especially unclear, but is strongly resolved by our combined likelihood (Fig. 1) and parsimony analyses (S14–S15 Figs.). Previous studies traditionally placed Mosasauria within Anguimorpha (e.g. [36–38]), including hypotheses in which snakes are placed with mosasaurs (e.g. [38]). In contrast, the preferred analyses of GEA ([8]; their Fig. 3) place Mosasauria near the squamate root. Here, our combined-data likelihood analysis strongly places Mosasauria far from the root and outside of Anguimorpha, as the sister group to snakes (Fig. 1). Nevertheless, our analyses do not support a marine origin for snakes, since we show that the earliest snake lineages are the burrowing scolecophidians (Fig. 1).

Some Bayesian analyses of the combined data (S11–S13 Figs.) weakly support a different placement for mosasaurs than the combined-data likelihood (Fig. 1) and parsimony analyses (S14–S15 Figs.). Nevertheless, all combined analyses agree that Mosasauria is within Toxicofera (S8–S15 Figs.). In some Bayesian analyses, Mosasauria is placed as sister to the clade of polyglyphanodontians and iguanians (S11–13 Figs.), but a key Bayesian analysis (all taxa included, select characters ordered; S10 Fig.) places mosasaurs with snakes with relatively strong support.

A particularly surprising result of our combined likelihood analyses is the strongly supported placement of the enigmatic fossil clade Polyglyphanodontia as sister group to Iguania (Fig. 1). Traditionally, Polyglyphanodontia has been considered closely related to or nested within teiids (e.g. [31, 39, 40]). A recent analysis of morphological data alone [8] weakly placed polyglyphanodontians outside other living scleroglossans, near the squamate root, as do our morphology-only analyses (S1–S4 Figs.). Remarkably, our combined analyses of molecular and morphological data (including likelihood, parsimony, and Bayesian analyses; Fig. 1; S8–S15 Figs.) strongly place Polyglyphanodontia as sister to Iguania, with support that includes 10 unambiguous morphological synapomorphies (characters 22, 83, 111, 154, 208, 283, 285, 434, 455, 521). Some earlier authors noted the similarity between polyglyphanodontians and iguanians, and some polyglyphanodontians were initially considered iguanians [41]. GEA [8] also noted that polyglyphanodontians lacked several scleroglossan synapomorphies. Thus, in some ways, polyglyphanodontians may be morphologically intermediate between iguanians and more traditionally recognized scleroglossans.

## Discussion

Our study had two main goals. First, to resolve higher-level squamate relationships, especially the controversial placement of Iguania. Second, to use the combined data to address the placement of major fossil squamate taxa.

Our combined analyses strongly suggest that the phylogenetic hypothesis for living squamates based on the molecular data is correct. Specifically, our results support the hypothesis that Iguania is placed with snakes and anguimorphs, and not at the squamate root (as suggested by morphological data alone). Our conclusions are based on several lines of evidence, including: (a) combined analyses of the relevant molecular and morphological data supports the molecular placement of Iguania, even when the molecular dataset is reduced to only 63 characters, less than one tenth the size of the morphological dataset, (b) mapping morphological characters on the combined-data tree shows that there is actually hidden support for the molecular hypothesis in the morphological data (similar to the number of characters supporting the morphological hypothesis), (c) the morphological dataset is dominated by misleading phylogenetic signal associated with convergent evolution of a burrowing lifestyle and associated traits, and a similar problem associated with feeding modes may explain the morphological placement of Iguania, and (d) the morphological hypothesis is unambiguously supported by only one of six subsets of the morphological data. Conversely, we find no evidence for hidden signal supporting the morphological hypothesis among the 46 genes in the molecular dataset; no genes support this hypothesis. Further, the failure of some genes to fully support the molecular placement of iguanians in Toxicofera seems to be associated with sampling error (i.e. shorter genes).

Recent authors have suggested that squamate phylogeny is presently unresolved because trees from separately analyzed molecular and morphological datasets do not agree [8, 9]. However, such conflicts between morphological and molecular datasets can never be resolved by simply comparing trees from separately analyzed datasets. For example, using this approach, even if the morphological dataset contained only one character, and the molecular dataset contained two million, the relationships could still never be considered to be resolved. Combined analysis is a key step in resolving such conflicts (e.g. [42–44]), along with identification of causes of error (such as convergent morphological evolution associated with burrowing or feeding modes).

In conclusion, previous authors have emphasized the conflict between morphological and molecular results for higher-level squamate phylogeny. Our analyses suggest that this conflict is now strongly resolved for the living taxa, favoring the molecular tree. However, morphological data still play a critical role in understanding squamate phylogeny, as they offer the only way to directly incorporate fossil taxa. Our results further illustrate that analyses that integrate molecular, morphological, and fossil data can lead to surprising and (in some cases) well-supported placements of fossil taxa (e.g. mosasaurs, polyglyphanodontians). Thus, our results also caution against estimating fossil relationships without considering relevant molecular data [27], even though this remains standard practice in analyses of fossil taxa that include representatives of living clades. Similarly, our analyses suggest that fossil taxa should not simply be assigned to clades in molecular trees (as is routinely done for fossil calibrations in phylogenetic dating analyses) without considering the potential impact of the combined molecular and morphological data on their placement [45].

## Supporting Information

S1 Appendix New morphological data. (DOC)

S2 Appendix Analyzing morphological partitions with the approximately unbiased test. (DOC)

S1 Fig Estimated phylogeny of squamates based on likelihood analysis of 691 morphological characters (-ln*L* = 30399.31; values along branches indicate bootstrap support; branches without values have bootstraps <50%). All multi-state characters are unordered. Daggers indicate fossil taxa.
(PDF)

S2 Fig Estimated phylogeny of squamates based on Bayesian analysis of 691 morphological characters. Selected multi-state characters are ordered. Numbers along branches are posterior probabilities. Daggers indicate fossil taxa.
(PDF)

S3 Fig Estimated phylogeny of squamates based on Bayesian analysis of 691 morphological characters. All multi-state characters are unordered. Numbers along branches are posterior probabilities. Daggers indicate fossil taxa.
(PDF)

S4 Fig Estimated phylogeny of squamates based on parsimony analysis of 691 morphological characters. The phylogeny is a strict consensus of 384 trees (length = 6088 steps). Numbers at nodes are bootstrap support values >50%. Selected multi-state characters are ordered. Daggers indicate fossil taxa.
(PDF)

S5 Fig Estimated phylogeny of squamates based on likelihood analysis of 46 loci (35,673 characters/base pairs;-ln*L* = 945429.70). Numbers at nodes are bootstrap support values >50%.
(PDF)

S6 Fig Estimated phylogeny of squamates based on Bayesian analysis of 46 loci (35,673 characters/base pairs). Numbers along branches are posterior probabilities.
(PDF)

S7 Fig Estimated phylogeny of squamates based on parsimony analysis of 46 loci (35,673 characters/base pairs). The phylogeny is a strict consensus of three trees (length = 197,520 steps). Numbers along branches are bootstrap support values >50%.
(PDF)

S8 Fig Estimated phylogeny of squamates based on likelihood analysis of the combined morphological and molecular data, including all taxa (-ln*L* = 979677.56). Numbers along branches are bootstrap support values >50%. Daggers indicate fossil taxa.
(PDF)

S9 Fig Estimated phylogeny of squamates based on likelihood analysis of the combined morphological and molecular data, excluding four rogue taxa (-ln*L* = 979285.16). Numbers along branches are bootstrap support values >50%. This tree is the same as in Fig. 1, but includes bootstrap values for all branches. Daggers indicate fossil taxa.
(PDF)

S10 Fig Estimated phylogeny of squamates based on Bayesian analysis of the combined morphological and molecular data, including all taxa. Numbers along branches are posterior probabilities. Selected multi-state characters are ordered. Daggers indicate fossil taxa.
(PDF)

S11 Fig Estimated phylogeny of squamates based on Bayesian analysis of the combined morphological and molecular data, excluding the four “rogue taxa” identified from the likelihood analysis. Numbers along branches are posterior probabilities. Selected multi-state characters are ordered. Daggers indicate fossil taxa.
(PDF)

S12 Fig Estimated phylogeny of squamates based on Bayesian analysis of the combined morphological and molecular data, including all taxa. Numbers along branches are posterior probabilities. All multi-state characters are unordered. Daggers indicate fossil taxa.
(PDF)

S13 Fig Estimated phylogeny of squamates based on Bayesian analysis of the combined morphological and molecular data, with four rogue taxa removed. Numbers along branches are posterior probabilities. All multi-state characters are unordered. Daggers indicate fossil taxa.
(PDF)

S14 Fig Estimated phylogeny of squamates based on parsimony analysis of the combined morphological and molecular data, including all taxa. The phylogeny is a strict consensus of 1344 trees (length = 207,375 steps). Numbers at nodes are bootstrap support values >50%. Daggers indicate fossil taxa.
(PDF)

S15 Fig Estimated phylogeny of squamates based on parsimony analysis of the combined morphological and molecular data, excluding the four “rogue taxa” identified from the likelihood analysis. The phylogeny is a strict consensus of 938 trees (length = 207,293 steps). Numbers at nodes are bootstrap support values >50%. Daggers indicate fossil taxa.
(PDF)

S16 Fig Estimated phylogeny of squamates based on likelihood analysis of the combined morphological and molecular data, with the molecular data recoded as 63 binary characters, and showing bootstrap values for all branches (-ln*L* = -31374.68). (PDF)

S17 Fig Estimated phylogeny of squamates based on likelihood analysis of the combined morphological and molecular data, with the molecular data recoded as 63 binary characters, excluding fossil taxa, and showing bootstrap values for all branches (-ln*L* = -27032.48). (PDF)

S18 Fig Estimated phylogeny of squamates based on likelihood analysis of the combined morphological and molecular data, with the molecular data recoded as 63 binary characters, excluding fossil and 12 burrowing taxa, and showing bootstrap values for all branches (-ln*L* = -24363.55). (PDF)

S19 Fig Estimated phylogeny of squamates based on Bayesian analysis of the combined morphological and molecular data, with the molecular data recoded as 63 binary characters. (PDF)

S20 Fig Estimated phylogeny of squamates based on Bayesian analysis of the combined morphological and molecular data, with the molecular data recoded as 63 binary characters, excluding fossil taxa. (PDF)

S21 Fig Estimated phylogeny of squamates based on Bayesian analysis of the combined morphological and molecular data, with the molecular data recoded as 63 binary characters, excluding fossil and 12 burrowing taxa. (PDF)

S22 Fig Estimated phylogeny of squamates based on parsimony analysis of the combined morphological and molecular data, with the molecular data recoded as 63 binary characters. Strict consensus of 1274 shortest trees of length 6277. See S25 Fig. for bootstrap values.
(PDF)

S23 Fig Estimated phylogeny of squamates based on parsimony analysis of the combined morphological and molecular data, with the molecular data recoded as 63 binary characters, excluding fossil taxa. Strict consensus of 67 shortest trees of length 5437. See S26 Fig. for bootstrap values.
(PDF)

S24 Fig Estimated phylogeny of squamates based on parsimony analysis of the combined morphological and molecular data, with the molecular data recoded as 63 binary characters, excluding fossil and 12 burrowing taxa. Strict consensus of 237 shortest trees of length 4845. See S27 Fig. for bootstrap values.
(PDF)

S25 Fig Estimated phylogeny of squamates based on parsimony bootstrap analysis of the combined morphological and molecular data, with the molecular data recoded as 63 binary characters. Bootstrap values less than 50% are not shown and the corresponding branch is collapsed.
(PDF)

S26 Fig Estimated phylogeny of squamates based on parsimony bootstrap analysis of the combined morphological and molecular data, with the molecular data recoded as 63 binary characters, excluding fossil taxa. Bootstrap values less than 50% are not shown and the corresponding branch is collapsed.
(PDF)

S27 Fig Estimated phylogeny of squamates based on parsimony bootstrap analysis of the combined morphological and molecular data, with the molecular data recoded as 63 binary characters, excluding fossil and 12 burrowing taxa. Bootstrap values less than 50% are not shown and the corresponding branch is collapsed.
(PDF)

S28 Fig Estimated phylogeny of squamates based on maximum likelihood analysis of the cranial characters only, including both living and fossil taxa (ln*L* = -14617.74). Numbers at nodes indicate bootstrap support values.
(PDF)

S29 Fig Estimated phylogeny of squamates based on maximum likelihood analysis of the cranial characters only, including living taxa only (ln*L* = -12120.63). Numbers at nodes indicate bootstrap support values.
(PDF)

S30 Fig Estimated phylogeny of squamates based on maximum likelihood analysis of the jaw-related characters only, including both living and fossil taxa (ln*L* = -4814.21). Numbers at nodes indicate bootstrap support values.
(PDF)

S31 Fig Estimated phylogeny of squamates based on maximum likelihood analysis of the jaw-related characters only, including living taxa only (ln*L* = -3919.37). Numbers at nodes indicate bootstrap support values.
(PDF)

S32 Fig Estimated phylogeny of squamates based on maximum likelihood analysis of the vertebral characters only, including both living and fossil taxa (ln*L* = -746.33). Numbers at nodes indicate bootstrap support values.
(PDF)

S33 Fig Estimated phylogeny of squamates based on maximum likelihood analysis of the vertebral characters only, including living taxa only (ln*L* = -631.80). Numbers at nodes indicate bootstrap support values.
(PDF)

S34 Fig Estimated phylogeny of squamates based on maximum likelihood analysis of the postcranial characters only, including both living and fossil taxa (ln*L* = -2586.55). Numbers at nodes indicate bootstrap support values.
(PDF)

S35 Fig Estimated phylogeny of squamates based on maximum likelihood analysis of the postcranial characters only, including living taxa only (ln*L* = -2361.41). Numbers at nodes indicate bootstrap support values.
(PDF)

S36 Fig Estimated phylogeny of squamates based on maximum likelihood analysis of the miscellaneous morphological characters only, including both living and fossil taxa (ln*L* = -891.075714). Numbers at nodes indicate bootstrap support values.
(PDF)

S37 Fig Estimated phylogeny of squamates based on maximum likelihood analysis of the miscellaneous morphological characters only, including living taxa only (ln*L* = -869.32). Numbers at nodes indicate bootstrap support values.
(PDF)

S38 Fig Estimated phylogeny of squamates based on maximum likelihood analysis of the scalation characters only, including living taxa only (ln*L* = -3477.28). Numbers at nodes indicate bootstrap support values.
(PDF)

S39 Fig Estimated phylogeny of squamates based on Bayesian analysis of the cranial characters only, including both living and fossil taxa. Numbers at nodes indicate posterior probabilities.
(PDF)

S40 Fig Estimated phylogeny of squamates based on Bayesian analysis of the cranial characters only, including living taxa only. Numbers at nodes indicate posterior probabilities.
(PDF)

S41 Fig Estimated phylogeny of squamates based on Bayesian analysis of the jaw-related characters only, including both living and fossil taxa. Numbers at nodes indicate posterior probabilities.
(PDF)

S42 Fig Estimated phylogeny of squamates based on Bayesian analysis of the jaw-related characters only, including living taxa only. Numbers at nodes indicate posterior probabilities.
(PDF)

S43 Fig Estimated phylogeny of squamates based on Bayesian analysis of the vertebral characters only, including both living and fossil taxa. Numbers at nodes indicate posterior probabilities.
(PDF)

S44 Fig Estimated phylogeny of squamates based on Bayesian analysis of the vertebral characters only, including living taxa only. Numbers at nodes indicate posterior probabilities.
(PDF)

S45 Fig Estimated phylogeny of squamates based on Bayesian analysis of the postcranial characters only, including both living and fossil taxa. Numbers at nodes indicate posterior probabilities.
(PDF)

S46 Fig Estimated phylogeny of squamates based on Bayesian analysis of the postcranial characters only, including living taxa only. Numbers at nodes indicate posterior probabilities.
(PDF)

S47 Fig Estimated phylogeny of squamates based on Bayesian analysis of the miscellaneous morphological characters only, including both living and fossil taxa. Numbers at nodes indicate posterior probabilities.
(PDF)

S48 Fig Estimated phylogeny of squamates based on Bayesian analysis of the miscellaneous morphological characters only, including living taxa only. Numbers at nodes indicate posterior probabilities.
(PDF)

S49 Fig Estimated phylogeny of squamates based on Bayesian analysis of the scalation characters only, including living taxa only. Numbers at nodes indicate posterior probabilities.
(PDF)

S50 Fig Estimated phylogeny of squamates based on parsimony analysis of the cranial characters only, including both living and fossil taxa. Strict consensus of 10,000 shortest trees (maximum number of trees retained) of length 2923. See S61 Fig. for bootstrap values.
(PDF)

S51 Fig Estimated phylogeny of squamates based on parsimony analysis of the cranial characters only, including living taxa only. Strict consensus of 10,000 shortest trees (maximum number of trees retained) of length 2447. See S62 Fig. for bootstrap values.
(PDF)

S52 Fig Estimated phylogeny of squamates based on parsimony analysis of the jaw-related characters only, including both living and fossil taxa. Strict consensus of 10,000 shortest trees (maximum number of trees retained) of length 993. See S63 Fig. for bootstrap values.
(PDF)

S53 Fig Estimated phylogeny of squamates based on parsimony analysis of the jaw-related characters only, including living taxa only. Strict consensus of 10,000 shortest trees (maximum number of trees) of length 810. See S64 Fig. for bootstrap values.
(PDF)

S54 Fig Estimated phylogeny of squamates based on parsimony analysis of the vertebral characters only, including both living and fossil taxa. Strict consensus of 10,000 shortest trees (maximum number of trees retained) of length 173. See S65 Fig. for bootstrap values.
(PDF)

S55 Fig Estimated phylogeny of squamates based on parsimony analysis of the vertebral characters only, including living taxa only. Strict consensus of 10,000 shortest trees (maximum number of trees retained) of length 145. See S66 Fig. for bootstrap values.
(PDF)

S56 Fig Estimated phylogeny of squamates based on parsimony analysis of the postcranial characters only, including both living and fossil taxa. Strict consensus of 10,000 shortest trees (maximum number of trees retained) of length 481. See S67 Fig. for bootstrap values.
(PDF)

S57 Fig Estimated phylogeny of squamates based on parsimony analysis of the postcranial characters only, including living taxa only. Strict consensus of 10,000 shortest trees (maximum number of trees retained) of length 442. See S68 Fig. for bootstrap values.
(PDF)

S58 Fig Estimated phylogeny of squamates based on parsimony analysis of the miscellaneous morphological characters only, including both living and fossil taxa. Strict consensus of 10,000 shortest trees (maximum number of trees retained) of length 188. See S69 Fig. for bootstrap values.
(PDF)

S59 Fig Estimated phylogeny of squamates based on parsimony analysis of the miscellaneous morphological characters only, including living taxa only. Strict consensus of 10,000 shortest trees (maximum number of trees retained) of length 181. See S70 Fig. for bootstrap values.
(PDF)

S60 Fig Estimated phylogeny of squamates based on parsimony analysis of the scalation characters only, including living taxa only. Strict consensus of 10,000 shortest trees (maximum number of trees retained) of length 660. See S71 Fig. for bootstrap values.
(PDF)

S61 Fig Estimated phylogeny of squamates based on parsimony analysis of the cranial characters only, including both living and fossil taxa, showing the majority rule-consensus tree from a bootstrapping analysis. (PDF)

S62 Fig Estimated phylogeny of squamates based on parsimony analysis of the cranial characters only, including living taxa only, showing the majority rule-consensus tree from a bootstrapping analysis. (PDF)

S63 Fig Estimated phylogeny of squamates based on parsimony analysis of the jaw-related characters only, including both living and fossil taxa, showing the majority rule-consensus tree from a bootstrapping analysis. (PDF)

S64 Fig Estimated phylogeny of squamates based on parsimony analysis of the jaw-related characters only, including living taxa only, showing the majority rule-consensus tree from a bootstrapping analysis. (PDF)

S65 Fig Estimated phylogeny of squamates based on parsimony analysis of the vertebral characters only, including both living and fossil taxa, showing the majority rule-consensus tree from a bootstrapping analysis. (PDF)

S66 Fig Estimated phylogeny of squamates based on parsimony analysis of the vertebral characters only, including living taxa only, showing the majority rule-consensus tree from a bootstrapping analysis. (PDF)

S67 Fig Estimated phylogeny of squamates based on parsimony analysis of the postcranial characters only, including both living and fossil taxa, showing the majority rule-consensus tree from a bootstrapping analysis. (PDF)

S68 Fig Estimated phylogeny of squamates based on parsimony analysis of the postcranial characters only, including living taxa only, showing the majority rule-consensus tree from a bootstrapping analysis. (PDF)

S69 Fig Estimated phylogeny of squamates based on parsimony analysis of the miscellaneous morphological characters only, including both living and fossil taxa, showing the majority rule-consensus tree from a bootstrapping analysis. (PDF)

S70 Fig Estimated phylogeny of squamates based on parsimony analysis of the miscellaneous morphological characters only, including living taxa only, showing the majority rule-consensus tree from a bootstrapping analysis. (PDF)

S71 Fig Estimated phylogeny of squamates based on parsimony analysis of the scalation characters only, including living taxa only, showing the majority rule-consensus tree from a bootstrapping analysis. (PDF)

S1 Table GenBank numbers for the two new loci. GenBank numbers for the two new loci (c-*Mos* and *ND2*) added to the molecular data matrix of Wiens et al. (10) for this study. For species denoted with “*” an exact match to those species used in the original matrix were not available, and related species (same genus or family) were used instead. The species used in the original matrix are in parentheses.
(DOC)

S2 Table Summary of RogueNaRok results, with maximum of 1 species in drop set. Summary of RogueNaRok results, illustrating the impacts of excluding specific sets of taxa, where the maximum number of taxa in a drop set is 1. “Raw improvement” is the overall fraction of improvement in bootstrap values. RIBC is the relative bipartition information criterion.
(DOC)

S3 Table Summary of RogueNaRok results, with maximum of 2 species in drop set. Summary of RogueNaRok results, illustrating the impacts of excluding specific sets of taxa, where the maximum number of taxa in a drop set is 2 (but fewer taxa may be selected). “Raw improvement” is the overall fraction of bootstrap improvement. RIBC is the relative bipartition information criterion.
(DOC)

S4 Table Summary of RogueNaRok results, with maximum of 3 species in drop set. Summary of RogueNaRok results, illustrating the impacts of excluding specific sets of taxa, where the maximum number of taxa in a drop set is 3 (but fewer taxa may be selected). “Raw improvement” is the overall fraction of bootstrap improvement. RIBC is the relative bipartition information criterion.
(DOC)

S5 Table Summary of phylogenetic placement of Iguania across the 46 genes. Summary of phylogenetic placement of Iguania in the 46 genes analyzed in this study, showing the clade or clades reconstructed as the sister group to Iguania, whether Toxicofera is supported or not, and whether monophyly of Iguania is supported. None of the trees show Iguania as sister to all other Squamata.
(DOC)

S6 Table Length (in base pairs) and number of parsimony-informative characters for each of the 46 genes. Length and number of parsimony-informative characters for each of the 46 genes used in the analyses of squamate phylogeny. The first 44 genes are listed alphabetically, whereas the last 2 genes listed are new to this study (relative to ref. [10]).
(DOC)
