# Supplementary material for: Integrated Analyses Resolve Conflicts over Squamate Reptile Phylogeny and Reveal Unexpected Placements for Fossil Taxa
Source: PLoS One. 2015 Mar 24;10(3):e0118199. doi: 10.1371/journal.pone.0118199 (PMC4372529; doi:10.1371/journal.pone.0118199)
Supplement: S1 Appendix — (DOC) [file pone.0118199.s001.doc]

**Appendix S1. New Morphological Data**

**Methods for New Morphological Characters**

External characters, mostly describing variation in scalation, were scored based on alcohol-preserved specimens. Following typical practice in morphological studies, most characters were described qualitatively and coded as binary or multi-state. Multi-state characters that involved quantitative variation along a single axis (e.g. number of scales of a particular type) were ordered, and all others were unordered. Ordering for quantitative characters is justified given that the states of these characters were defined assuming that similarity in trait values is informative. Therefore, it is only logical to use this same assumption in ordering the states. The alternative is to assume that similarity in quantitative trait values is not informative, in which case many taxa would have to be given a unique state for these characters (most taxa are not identical), the states would be unordered, and the characters would be largely uninformative. Nevertheless, we explore the impact of ordering vs. unordering these multi-state characters on our analyses.

Characters were coded so that they could be analyzed by likelihood, Bayesian, and parsimony methods. Unfortunately, available versions of MrBayes do not allow for use of step matrices or >5 ordered character states (>5 unordered states are allowed, however). Therefore, it was not possible to use frequency coding of polymorphic characters or gap-weighting of quantitative characters (despite some advantages of these methods; [46, 47]), and a few multistate morphological characters had to be recoded for Bayesian analysis by lumping states greater than five into a single state.

Given our focus on higher-level relationships, sample sizes within species were generally limited (*n* = 1–3, mode = 2). Most polymorphism was coded using the polymorphic method (review in [46]), given that frequency methods would be difficult to implement in MrBayes and RAXML, and the majority method can only be applied arbitrarily to a frequency of 50%.

**Descriptions of New Morphological Characters**

The references associated with these descriptions indicate the first paper in which the character was used in an explicit phylogenetic analysis. The numbering of characters includes the number in the combined morphological data matrix (in parentheses). Characters 1–610 are from Gauthier et al. [8]. Ordered characters are indicated with an asterisk.

# External morphology

**Nasal region**

1 (611). Enlarged rostral scale: (0) absent, undifferentiated, (1) present.

2 (612). Rostral scale: (0) not greatly expanded and dominating anterior portion of head, (1) expanded dorsally (onto top of skull), posteriorly, and laterally, dominating anterior portion of head.

3 (613). Posterior split in rostral scale: (0) absent, (1) present (division along posterior margin of scale near midline).

4 (614). External narial opening: (0) enclosed entirely within nasal scale, (1) at edge of nasal scale.

5 (615). External narial opening: (0) separated from rostral, (1) partially in rostral.

6 (616). External narial opening: (0) separated from supralabial, (1) partially in supralabial.

7 (617). Nasal-rostral contact: (0) absent, separated by scales, (1) present. When the narial opening is between scales rather than entirely within a scale, state 1 is used when one of these scales bordering the nasal contacts the rostral.

8 (618). Contact between nasal and supralabial scales: (0) absent (separated by other scales), (1) present. When the narial opening is between scales rather than entirely within a scale, state 1 is used when one of these scales bordering the nasal contacts the supralabial.

*9 (619). Minimum number of scales separating the nasal scales medially: (0) none (nasal scales contact medially), (1) one scale, (2) two, (3) three, (4) four or more scale rows. Previously used phylogenetically (in modified form) by Wiens ([48]; his char. 22). In species that varied intraspecifically, the mean number was used. Although this character ranges up to much higher scale counts, the states were truncated to allow for the five ordered states possible in current implementations of Bayesian analysis. If the nasal opening is surrounded by two or more scales, the count is from the medial-most scale containing the nasal opening, but does not include that scale. The rostral is not counted. Ordered.

**Eye region**

10 (620). Dorsal cephalic scales: (0) relatively small; (1) enlarged (larger than adjacent neck scales).

11 (621). Dorsal cephalic scales: (0) smooth, (1) some or all keeled.

12 (622). Head scales vertically elongate: (0) no, (1) yes, including scales associated with eye and nostril (extending almost from mouth to top of skull).

13 (623). Enlarged frontal scale between orbits: (0) absent, (1) present. Previously used phylogenetically by Wiens ([48]; his char. 18).

14 (624). Median contact of frontoparietals: (0) present, (1) absent, frontoparietals reduced and separated by contact of frontal and interparietal. Scored as unknown in taxa lacking frontals, frontoparietals, and an interparietal.

15 (625). Circumorbital scales (distinct semicircular row of scales bordering orbital region): (0) absent, (1) present.

16 (626). Enlarged supraoculars: (0) absent, (1) present (scales over eye larger than surrounding scales).

17 (627). Supraoculars: (0) two or more rows of scales, (1) single row of enlarged scales between superciliary and frontal. Taxa with multiple rows of scales in this region were given state 0, regardless of whether or not they had well-differentiated supraoculars. Previously used phylogenetically by Wiens ([48]; his char. 25).

18 (628). Single enlarged supraocular scale row between orbit laterally and frontal medially: (0) no, (1) yes, supraciliary scales absent.

19 (629). Median contact of enlarged supraoculars: (0) absent, (1) present.

20 (630). Supercilary scales: (0) non-overlapping, (1) overlapping. Used previously in modified form by Etheridge and de Queiroz ([49]; their char. 46) and subsequent authors.

21 (631). Supralabial scales: (0) not entering orbit, (1) entering orbit. In most squamates examined, one or more enlarged scales (suboculars) separate the supralabials from the rim of the orbital opening. In others, supralabials extend to the orbital opening.

22 (632). Enlarged subocular scale (elongate scale ventral to orbit): (0) absent (or series of smaller scales), (1) present (single scale larger than surrounding scales). In many taxa, there is a single scale below the orbit that is also part of the labial series bordering the upper jaw (see character 21). In these cases, it is possible that the subocular has entered the labial series or that the labial has shifed into the subocular position, and these taxa were coded as unknown for the presence or absence of a subocular. Used previously phylogenetically in modified form by Etheridge and de Queiroz ([49]; their char. 47) and subsequent authors.

23 (633). Subocular-supralabial contact: (0) absent, row of one or more scales between scales above lip (supralabials) and below the orbit (suborbitals), (1) present. Taxa lacking any subocular scales below the eye (e.g., many snakes) were coded as inapplicable.

24 (634). Enlarged interparietal scale: (0) absent, (1) present (scale distinctly larger than surrounding head scales present over parietal region of skull). Used in modified form by Etheridge and de Queiroz ([49]; their char. 45) and subsequent authors.

25 (635). Temporal region covered almost exclusively by pair of enlarged temporal scales: (0) no, (1) yes. Derived state is mostly in advanced snakes.

26 (636). Heat-sensing pit in loreal region: (0) absent, (1) present.

**Mouth**

27 (637). Mouth: (0) large, extending posterior to eye, (1) greatly reduced, posterior terminus at or anterior to level of eye.

28 (638). Groove allowing tongue to be extruded when mouth is closed: (0) absent, (1) groove present in rostral (sometimes in mental also, but too difficult to score consistently as a separate state). The derived state is found in snakes.

**Eye**

29 (639). Eye: (0) visible externally, (1) not visible externally. Previously used phylogenetically (in modified form) by Kearney ([50]; her char. 10). Derived state is found only in *Rhineura*.

30 (640). Eye, surrounded by single scale: (0) no, multiple scales, (1) single scale. Previously used phylogenetically (in modified form) by Lee and Scanlon ([51]; their char. 253).

31 (641). Fleshy eyelids: (0) present; (1) absent. Previously used phylogenetically (in modified form) by Lee and Scanlon ([51]; their char. 253).

32 (642). Lower eyelid scales: (0) homogeneous, (1) some enlarged scales.

33 (643). Transparent spectacle in lower eyelid scales: (0) absent, (1) present.

34 (644). Thick granular eyelid covering eye except for small opening for pupil (chamaeleon condition): (0) absent; (1) present. Used previously phylogenetically by Estes et al. ([52]; p. 199).

**Ear**

35 (645). External ear opening (0) present, (1) absent. Previously used phylogenetically by Estes et al. (48).

36 (646). Tympanum: (0) large and superficial; (1) small and inset. Used previously phylogenetically by Estes et al. ([51] and noted by McDowell and Bogert [36]).

37 (647). External ear opening: (0) entirely or mostly uncovered, (1) partially covered by fleshy lobule, (2) partially covered by scales only, no fleshy lobule. Unordered.

**Throat/chin**

38 (648). Complex dewlap apparatus (gular fan), in males: (0) absent; (1) present, extensive flap of skin on throat, usually with modified scales.

39 (649). Mental scale: (0) entire, (1) with a partial split at the posterior end. Previously noted by Frost and Etheridge ([53]; from E. Williams, pers. comm.).

40 (650). Elongate postmentals: (0) absent, (1) present.

41 (651). Postmentals (scales immediately posterior to mental): (0) two (enlarged scales sometimes separated by additional scales) or more scales immediately posterior to mental, (1) one, single unpaired scale immediately posterior to mental.

42 (652). Enlarged scales medial to infralabials: (0) present, (1) absent, scales in chin more-or-less homogeneous.

*43 (653). Infralabials, anteriormost pair(s): (0) separate medially, (1) one row contacting medially posterior to mental, (2) two rows contacting medially. It is possible that the medially contacting infralabials actually represent postmentals that have shifted their position. Ordered.

44 (654). Infralabial and supralabial scales: (0) not greatly expanded, (1) greatly expanded, with single pair of sublabials covering most of chin region (i.e., dibamids).

45 (655). Mental groove: (0) absent, (1) present, a distinct infolding of skin and scales along the anterior midline of the chin region.

*46 (656). Gular fold: (0) present, complete, (1) incomplete; (2) absent. Ordered. Previously used phylogenetically by Etheridge and de Queiroz ([49]; their char. 43) and subsequent authors.

**Dorsal body region**

47 (657). Body scales: (0) not arranged in non-overlapping rings; (1) annulated, arranged in non-overlapping rows. Previously used phylogenetically by Kearney ([50]; her char. 3).

48 (658). *Varanus*-type scale on dorsum, with small scale surrounded by tiny granular scales: (0) absent, (1) present.

49 (659). Dorsal scales: (0) homogeneous, lacking enlarged scales, (1) heterogeneous, with enlarged scales separated by smaller scales, either in rows or scattered.

50 (660). Dorsal body scales: (0) small, granular, non-overlapping, (1) mucronate (pointed, overlapping), (2) cycloid (rounded, overlapping), (3) rectangular. Unordered. Expanded from char. 148 of Estes et al. [52].

51 (661). Dorsal body scales: (0) all smooth; (1) some or all keeled.

52 (662). Dorsals, with longitudinal ridges in addition to mid-dorsal keels: (0) absent (including species lacking keels), (1) present

53 (663). Enlarged middorsal scale row: (0) middorsal scales same size as surrounding scales; (1) middorsal scales enlarged relative to surrounding scales in both sexes; (2) sexually dimorphic, mid-dorsals enlarged in males. Unordered. Expanded and modified from char. 146 of Estes et al. [52].

54 (664). Nuchal crest of enlarged scales: (0) absent, (1) present in both sexes; (2) sexually dimorphic, mid-dorsals enlarged in males. Unordered. Has similar distribution to that of mid-dorsal scale row, but clearly not identical.

55 (665). Lateral body scales: (0) granular, non-overlapping, (1) imbricate overlapping. Although this may seem redundant with the condition of the dorsal scales, several taxa have enlarged and imbricate dorsal scales but granular lateral scales.

56 (666). Lateral fold along side of body: (0) absent, (1) present (often associated with row of granular scales in species with larger scales). Previously used phylogenetically in squamates by Estes et al. [52].

**Venter**

57 (667). Gastrosteges (enlarged mid-ventral scale row in snakes): (0) absent, multiple scale rows; (1) present, single mid-ventral scale row distinctly larger than surrounding scale rows. Previously used phylogenetically (in modified form) by Lee and Scanlon ([51]; their char. 255).

58 (668). Ventrals: (0) same size as adjacent laterals, (1) at least some distinctly larger than adjacent laterals.

59 (669). Ventral body scales: (0) mostly small and granular; (1) large and flat. Note that this character is independent of the preceding character, because scales can be large and flat and still be the same size as the adjacent laterals.

60 (670). Ventral scales: (0) all smooth, (1) some or all keeled.

61 (671). Ventrals, posterior edge: (0) cycloid, mucronate, or otherwise rounded, (1) square (posterior margin more-or-less straight). Some snakes with gastrosteges have variation that approaches the condition in state 1, but were coded as 0.

62 (672). Femoral pores: (0) absent; (1) present in both sexes, (2) sexually dimorphic. Unordered. In some cases, femoral and preanal pores may be difficult to distinguish (e.g. *Gekko gecko, Takydromus ocellatus*). In general, pores on the legs were considered femoral pores, and pores between the legs were considered preanals, but pores that extended from the legs onto the preanal region were considered femorals. Taxa lacking hindlimbs were coded as unknown. Previously used phylogenetically by Estes et al. ([52]; but noted by Camp [54]), their character 144.

63 (673). Preanal pores: (0) absent; (1) present. Previously used phylogenetically by Estes et al. [52], their character 144.

**Tail**

64 (674). Tail tip: (0) pointed, tapered, (1) blunt and rounded

65 (675). Small sharply pointed spike at posterior tail tip: (0) absent, (1) present.

*66 (676). Tail shape (cross-section): (0) dorsoventrally flattened (depressed),(1) rounded, (2) laterally compressed. Ordered.

67 (677). Caudal scales: (0) approximately same size as dorsal body scales: (1) distinctly larger than dorsal body scales

68 (678). Caudals (dorsal): (0) smooth, (1) mostly keeled.

69 (679). Caudal scales: (0) not arranged in non-overlapping vertical rows; (1) annulated, arranged in non-overlapping vertical rows.

70 (680). Enlarged preanal scales: (0) absent, (1) present (preanals enlarged relative to adjacent ventrals).

71 (681). Anal plate: (0) two or more scales, (1) single plate. Species without a distinct anal plate but with multiple scales in the pre-anal region were coded as having state 0. Previously used phylogenetically by Lee and Scanlon ([51]; their char. 257).

72 (682). Enlarged postanal scales (males): (0) absent, (1) present (some scales posterior to vent greatly enlarged relative to adjacent scales).

73 (683). Lateral postanal tubercles: (0) absent, (1) present (enlarged scale or scales on medial surface of anterior end of tail).

*74 (684). Subcaudals (scales on ventral mid-line of tail): (0) divided, two or more scale rows, (1) some single, some divided, (2) all single. Ordered. Previously used phylogenetically (in modified form) by Lee and Scanlon ([51]; their char. 256).

**Limbs**

75 (685). External hindlimb: (0) present, (1) absent in both sexes, (2) sexually dimorphic, present in males, absent in females. Unordered. Taxa in which hindlimbs were observed in all individuals but the sex was uncertain were coded as having state 0.

76 (686). External hindlimbs: (0) cylindrical, (1) flattened and flaplike. Taxa having the fully-developed limbs and those having only a claw were both coded as having state 0.

77 (687). Expanded toe pads: (0) absent, (1) present. Previously used phylogenetically by Etheridge and de Queiroz ([49]; their char. 40) and subsequent authors.

78 (688). Number of rows of subdigital scales along mid-ventral surface of digits (manus): (0) one, (1) two or more (some or all scales). Previously used phylogenetically by Estes et al. (1988) and Etheridge and de Queiroz [49].

79 (689). Keels on subdigital scales (hand, longest finger): (0) absent, (1) present. Used previously phylogenetically by Etheridge and de Queiroz ([49]; their char. 39).

80 (690). Digits connected in groups of 2 and 3 by skin (chameleon “mittens”): (0) no, (1) yes. Previously used phylogenetically (in modified form) by Estes et al. ([52]; p. 198).

81 (691). Claws: (0) not covered by sheath of scales, (1) entirely or mostly covered by sheath of scales.

**Supplementary References**

46. Wiens JJ. Polymorphism in systematics and comparative biology. Ann Rev Ecol Syst. 1999; 30: 327–362.

47. Wiens JJ. Character analysis in morphological phylogenetics: problems and solutions. Syst Biol. 2001; 50, 689–699.

48. Wiens JJ. Phylogenetic relationships of phrynosomatid lizards and monophyly of the *Sceloporus* group. Copeia 1993; 1993: 287–299.

49. Etheridge R, de Queiroz K. A phylogeny of Iguanidae. In: Estes R, Pregill G, editors. The phylogenetic relationships of the lizard families*.* Palo Alto: Stanford University Press; 1988. pp. 283–367.

50. Kearney M. Systematics and evolution of the Amphisbaenia: a phylogenetic hypothesis based on morphological evidence from fossil and recent forms*.* Herpetol Mon. 2003; 17: 1–75.

51. Lee MSY, Scanlon JD. Snake phylogeny based on osteology, soft anatomy and ecology. Biol. Rev. 2002; 77: 333–401.

52. Estes R, de Queiroz K, Gauthier JA. Phylogenetic relationships within the Squamate In: Estes R, Pregill G, editors. The phylogenetic relationships of the lizard families*.* Palo Alto: Stanford University Press; 1988. pp. 119–281.

53. Frost DR, Etheridge R. A phylogenetic analysis and taxonomy of iguanian lizards. Misc. Publ. Univ. Kansas 1989; 81: 1–65.

54. Camp CL. Classification of the lizards. Bull. Am. Mus. Nat. Hist. 1923; 48: 289–481.

**Morphological Specimens Examined**

Alcohol-preserved specimens examined for external morphological characters. Specimens used were from the California Academy of Sciences (CAS), Carnegie Museum of Natural History (CM), Field Museum of Natural History (FMNH), University of Kansas Natural History Museum (KU), Los Angeles County Museum (LACM), Museum of Comparative Zoology, Harvard University (MCZ), Yale Peabody Museum (YPM), and the collection at the University of Texas at Arlington (UTA). Taxa are listed alphabetically by genus within families, and families are listed alphabetically within major clades.

Outgroup

Rhyncocephalia

*Sphenodon punctatus:* FMNH 1115, 72486.

Iguania

Agamidae: *Agama agama:* FMNH 47531, 58295, 62399. *Calotes emma:* FMNH 191302, 255499, 255508. *Leiolepis rubritaeniata:* FMNH 181066, 181072. *Physignathus cocincinus:*  FMNH 258709, 262959. *Pogona vitticeps:* YPM 10051, 12726. *Uromastyx hardwicki:* CM 4368, 65181, 65182.

Chamaeleonidae: *Brookesia brygooi:* FMNH 260015, 260017. *Chamaeleo calyptratus*: FMNH 66181, 66198.

Corytophanidae: *Basiliscus basiliscus:* FMNH 165617, 165619. *Corytophanes cristatus:* FMNH 49240, 69227.

Crotaphytidae: *Crotaphytus collaris:* FMNH 23281, 28504. *Gambelia wislizenii:* FMNH 6950, 213327.

Dactyloidae: *Anolis carolinensis:* FMNH 34819, 34825.

Hoplocercidae: *Enyalioides laticeps*: FMNH 30815, 206133. *Morunasaurus annularis:* FMNH 197965, 197966.

Iguanidae: *Brachylophus fasciatus:* FMNH 60117, 140290. *Dipsosaurus dorsalis:* FMNH 213324, 213918. *Sauromalus ater:* FMNH 174604, 174700.

Leiocephalidae: *Leiocephalus barahonensis:* KU 242347, 242359.

Leiosauridae: *Leiosaurus catamarcensis:* MCZ 58115, 96710. *Pristidactylus torquatus:* FMNH 206964, 206965. *Urostrophus vautieri:* FMNH 28863, 83576.

Liolaemidae: *Liolaemus elongatus:* FMNH 133741, 133749. *Phymaturus palluma:* FMNH 40161, 209123.

Opluridae: *Chalarodon madagascariensis:* FMNH 259821. *Oplurus cyclurus*: FMNH 3730.

Phrynosomatidae: *Petrosaurus mearnsi:* FMNH 8431, 37720. *Phrynosoma platyrhinos:* FMNH 11247, 37717. *Sceloporus variabilis:* FMNH 32994, 33019. *Uma scoparia:* FMNH 1203, 39440. *Uta stansburiana:* FMNH 75912, 75913.

Polychrotidae: *Polychrus marmoratus:* FMNH 45500, 45502.

Tropiduridae: *Stenocercus guentheri:* FMNH 27674, 36711. *Tropidurus plica:* FMNH 49835, 49838. *Uranoscodon superciliosus:* FMNH 26632, 30930.

Anguimorpha

Anguidae: *Diploglossus enneagrammus:* FMNH 111100, 111103. *Elgaria multicarinata:* FMNH 23177, 41524. *Ophisaurus apodus:* FMNH 141673, 141674. Anniellidae: *Anniella pulchra:* FMNH 8051, 41643.

Helodermatidae *Heloderma horridum:* FMNH 38532. *Heloderma suspectum:* FMNH 22866, 25751.

Lanthanotidae: *Lanthanotus borneensis:* FMNH 121986, 134699.

Shinisauridae *Shinisaurus crocodilurus:* FMNH 215542, 261569.

Varanidae: *Varanus acanthurus:* FMNH 97862. *Varanus exanthematicus:* FMNH 153120. *Varanus salvator:* FMNH 129513, 145712.

Xenosauridae *Xenosaurus grandis*: FMNH 102954–102955. *Xenosaurus platyceps:* UTA R-23594–R-23595.

Dibamidae: *Anelytropsis papillosus:* FMNH 69221, 100410. *Dibamus novaeguineae:* CAS 27069, CAS 27070.

Gekkota

Carphodactylidae: *Saltaurius cornutus:* FMNH 57495, 97699.

Diplodactylidae: *Rhacodactylus auriculatus:* CAS 200266, 214446*.* *Strophurus ciliaris:* FMNH 35152, 35153.

Eublepharidae: *Aeluroscalobates felinus:* FMNH 146093, 146102. *Coleonyx variegatus:* FMNH 26217, 213921. *Eublepharis macularius:* FMNH 215851, 217287.

Gekkonidae:  *Gekko gecko:* FMNH 202728, 202732. *Phelsuma lineata:* FMNH 72915, 72921, 260108.

Pygopodidae: *Delma borea:* USNM 128679–128680. *Lialis burtonis:* FMNH 29111, 97561.

Sphaerodactylidae: *Gonatodes albogularis*: FMNH 236177, 236195. *Teratoscincus przewalski:* FMNH 229902.

Lacertoidea

Gymnophthalmidae: *Alopoglossus angulatus:* FMNH 134725. *Colobosaura modesta:* MCZ 154233, USNM 341977, 341978. *Pholidobolus montium:* FMNH 204240, 204241. Lacertidae: *Lacerta viridis:* CM 65063, 65643. *Takydromus ocellatus:* FMNH 252363, 252374.

Teiidae: *Aspidoscelis tigris:* FMNH 29456, 35099. *Callopistes maculatus:* FMNH 212436, 223690. *Teius teyou:* FMNH 10864, 10873. *Tupinambis teguixin:* FMNH 161585, 168228.

Amphisbaenia

Amphisbaenidae: *Amphisbaena fuliginosa:* FMNH 22847, 40015. *Geocalamus acutus:* FMNH 265407, 265412.

Bipedidae: *Bipes biporus:* FMNH 18049, 18050. *Bipes canaliculatus:* FMNH 105041, 105044.

Rhineuridae: *Rhineura floridana:* FMNH 43387–43388.

Trognophidae: *Diplometopon zarudnyi:* FMNH 263883. *Trogonophis wiegmanni:* FMNH 265686, 265695.

Scincoidea

Cordylidae: *Cordylus mossambicus:* YPM 13408. *Platysaurus imperator:* MCZ 67614, 67620.

Gerrhosauridae: *Cordylosaurus subtesselatus:* FMNH 71928, 74083. *Zonosaurus ornatus:* FMNH 259996–259997.

Scincidae: *Acontias percivali:* CM 53630B, 53648. *Amphiglossus splendidus:* FMNH 72804, 72805. *Brachymeles gracilis:* FMNH 176468–176469. *Eugongylus rufescens:* CAS 118116, 118753, FMNH 142306. *Feylinia polylepis:* FMNH 120967–120967. *Plestiodon fasciatus:* FMNH 247206, FMNH 247213. *Scincus scincus:* FMNH 164668, 171834. *Sphenomorphus solomonis:* CAS 110090, 110097. *Tiliqua scincoides:* FMNH 97954, 97955. *Trachylepis quinquetaeniata:* FMNH 205982, 205984.

Xantusiidae: *Cricosaura typica*: FMNH 22538. *Lepidophyma flavimaculatum:* FMNH 60161, 176530. *Xantusia vigilis:* FMNH 23238, 23239.

Serpentes

Acrochordidae: *Acrochordus granulatus:* FMNH 213052, 213056.

Aniliidae : *Anilius scytale:* FMNH 34471, 35687.

Anomalepididae: *Liotyphlops albirostris:* FMNH 216257.

Boidae: *Boa constrictor:* FMNH 34301, 161541. *Epicrates striatus:* FMNH 197, 34678. *Eryx colubrinus:* FMNH 81221, 81223. *Exiliboa placata:* FMNH 202685, 2027669. *Lichanura trivirgata:* FMNH 11058, LACM 122536. *Ungaliophis continentalis:* UTA-R 18523, UTA-R 25119.

Calabariidae: *Calabaria reinhardtii:* FMNH 19479, 58043.

Colubridae: *Afronatrix anoscopus:* FMNH 48618, 190753. *Amphiesma stolata:* FMNH 169409, 169425. *Coluber constrictor:* FMNH 61833, 61834. *Diadophis punctatus:* FMNH 245322, 245366. *Heterodon platyrhinos:* FMNH 48967, 206640. *Imantodes cenchoa:* FMNH 62958, 165479. *Lampropeltis getula:* FMNH 192868, 192871. *Natrix natrix:* FMNH 79157, 79161. *Sonora semiannulata:* FMNH 26870, 26873.

*Thamnophis marcianus:* FMNH 45796, 45797. *Trimorphodon biscutatus:* FMNH 766, 74971, 105202. *Xenochrophis piscator:* FMNH 127253, 127259.

Cylindrophiidae: *Cylindrophis ruffus:* FMNH 158630–158631.

Elapidae: *Laticauda colubrina*: FMNH 198952, 202181. *Micrurus fulvius:* FMNH 8488, 95241. *Naja naja:* FMNH 224204, 240528. *Notechis scutatus:* FMNH 15800, 202669. Homalopsidae: *Homalopsis buccata:* FMNH 259090, 259091.

Lamprophiidae: *Aparallactus werneri:* FMNH 250439, 250440, 250442. *Atractaspis irregularis:* FMNH 34487, 62204. *Lamprophis fuliginosus:* FMNH 3997, 8980. *Lycophidion capense:* FMNH 78206, 78208.

Leptotyphlopidae: *Leptotyphlops dulcis:* FMNH 41783, 41785.

Loxocemidae: *Loxocemus bicolor:* FMNH 39075, LACM 150321–150322.

Pareatidae: *Pareas hamptoni:* FMNH 258682, 258683.

Pythonidae: *Aspidites melanocephalus*: FMNH 97054. *Python molurus:* FMNH 259166. Tropidophiidae: *Trachyboa boulengeri:* FMNH 78106, 131266*.* *Tropidophis haetianus:* FMNH 42117, KU 269425, 269139.

Typhlopidae: *Typhlops jamaicensis*: FMNH 235340, KU 269898, 269916.

Uropeltidae: *Uropeltis melanogaster:* FMNH 167014, 167016.

Viperidae: *Agkistrodon contortrix:* FMNH 41764, 41772. *Azemiops feae:* FMNH 152987, 218627. *Bothrops asper:* FMNH 20161, 20338. *Causus rhombeatus:* FMNH 17678, 74241. *Daboia russelli:* FMNH 169372, 199739. *Lachesis muta:* FMNH 165728, 166035.

Xenodermatidae: *Xenodermus javanicus:* FMNH 138678, 230073.

Xenopeltidae: *Xenopeltis unicolor:* FMNH 259168–259169.
