# Supplementary material for: Integrated Analyses Resolve Conflicts over Squamate Reptile Phylogeny and Reveal Unexpected Placements for Fossil Taxa
Source: PLoS One. 2015 Mar 24;10(3):e0118199. doi: 10.1371/journal.pone.0118199 (PMC4372529; doi:10.1371/journal.pone.0118199)
Supplement: S2 Appendix — (DOC) [file pone.0118199.s002.doc]

**Appendix S2. Analyzing Morphological Partitions with the Approximately Unbiased Test.**

We tested whether the six partitions of the morphological data significantly reject monophyly of Toxicofera, and whether any reject the traditional morphological placement of Iguania. We used the morphological data from each partition for extant taxa only, to avoid any potential artifacts due to missing data and/or the uncertain placement of fossil taxa (and given that including or excluding fossil taxa generally seemed to have little impact on higher-level relationships from these partitions; see electronic supplementary materials, figures S29–71). We performed these tests in a maximum likelihood framework using the approximately unbiased test (AU; [33]). We tested two phylogenetic hypotheses: (1) Toxicofera monophyly (i.e. a clade containing all iguanian, anguimorphan and snake taxa) to the exclusion of all other squamate taxa, and (2) Scleroglossa monophyly (i.e. a monophyletic Iguania being excluded from a clade containing all non-iguanian taxa). For each test and for each partition, we first performed a likelihood analysis of the data in which monophyly of the given clade was constrained (finding the best tree in which that clade is forced to be monophyletic). We then compared this tree to the optimal, unconstrained tree found for that data partition. The optimal unconstrained and constrained likelihood trees were inferred using RAxML-HPC2 v7.6.3 (under the MK model and gamma, see main text methods), and the site likelihoods for the optimal unconstrained and constrained trees (option –f G) were estimated in RAxML-HPC-AVX v8 [55]. The site likelihoods for the optimal alternative hypotheses were then statistically compared to the optimal unconstrained trees using the AU test as implemented in CONSEL v0.1k [56].

**Supplementary References**

55. Stamatakis A. RAxML version 8: A tool for phylogenetic analysis and post-analysis of large phylogenies. Bioinformatics 2014; 30: 1312–1313.

56. Shimodaira H, Hasegawa M. CONSEL: For assessing the confidence of phylogenetic tree selection. Bioinformatics 2001; 17: 1246–1247.
