# Supplementary figures and images for: Integrated Analyses Resolve Conflicts over Squamate Reptile Phylogeny and Reveal Unexpected Placements for Fossil Taxa
Source: PLoS One. 2015 Mar 24;10(3):e0118199. doi: 10.1371/journal.pone.0118199 (PMC4372529; doi:10.1371/journal.pone.0118199)

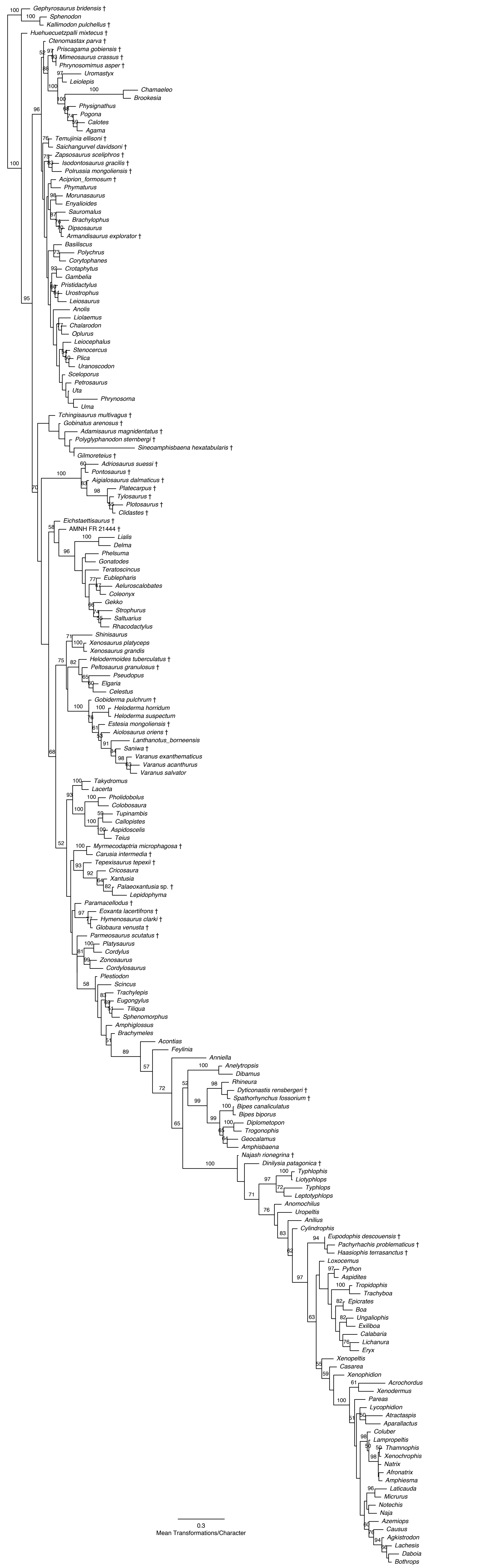

Supplement: S1 Fig — All multi-state characters are unordered. Daggers indicate fossil taxa. (PDF) [file pone.0118199.s003.pdf]

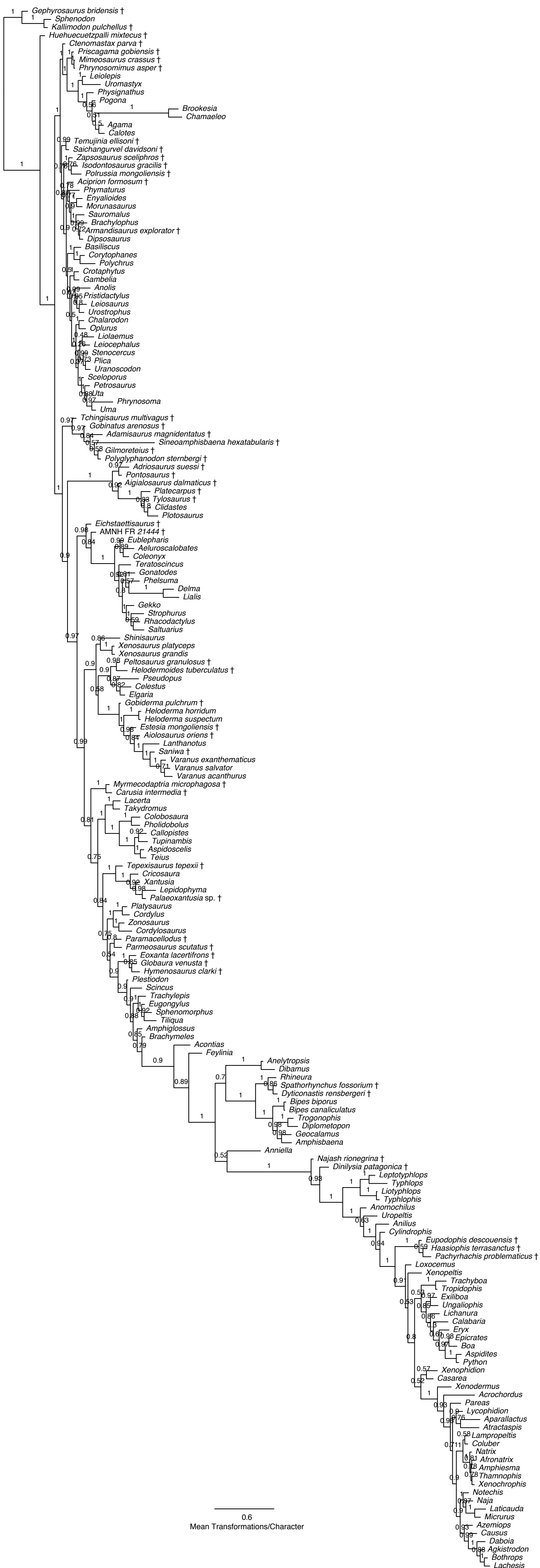

Supplement: S2 Fig — Selected multi-state characters are ordered. Numbers along branches are posterior probabilities. Daggers indicate fossil taxa. (PDF) [file pone.0118199.s004.pdf]

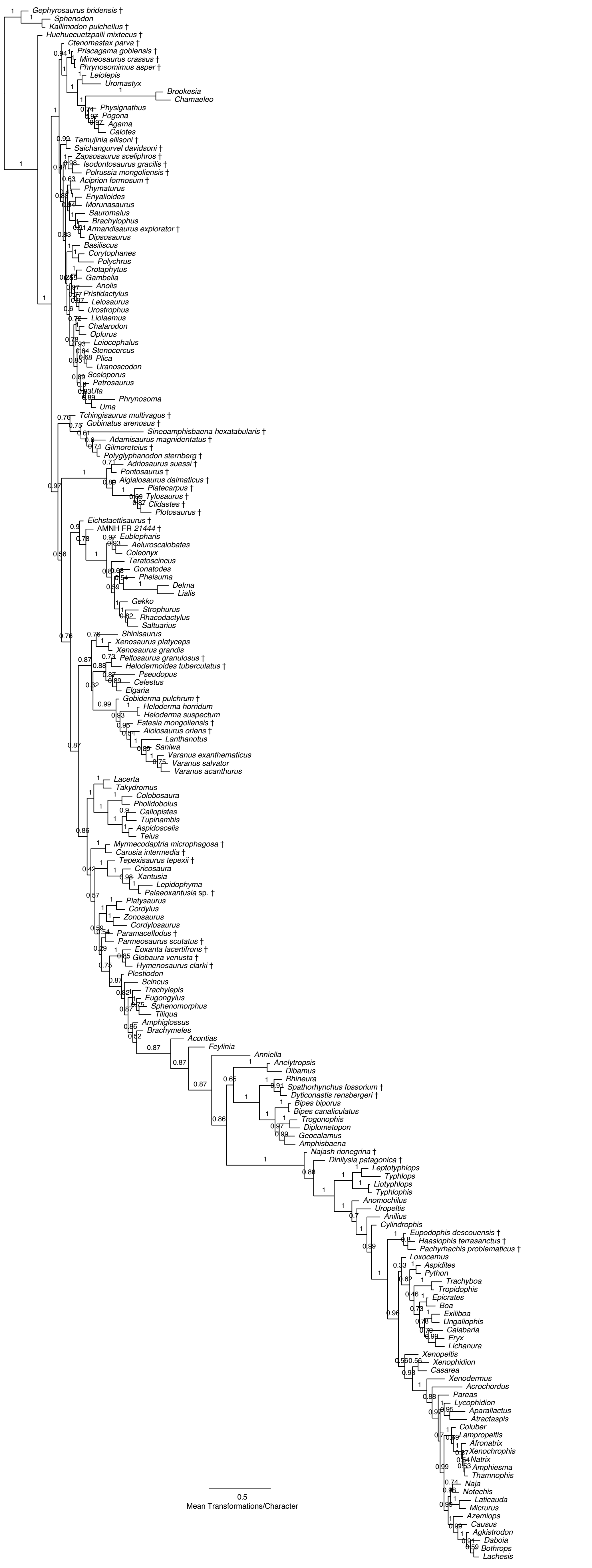

Supplement: S3 Fig — All multi-state characters are unordered. Numbers along branches are posterior probabilities. Daggers indicate fossil taxa. (PDF) [file pone.0118199.s005.pdf]

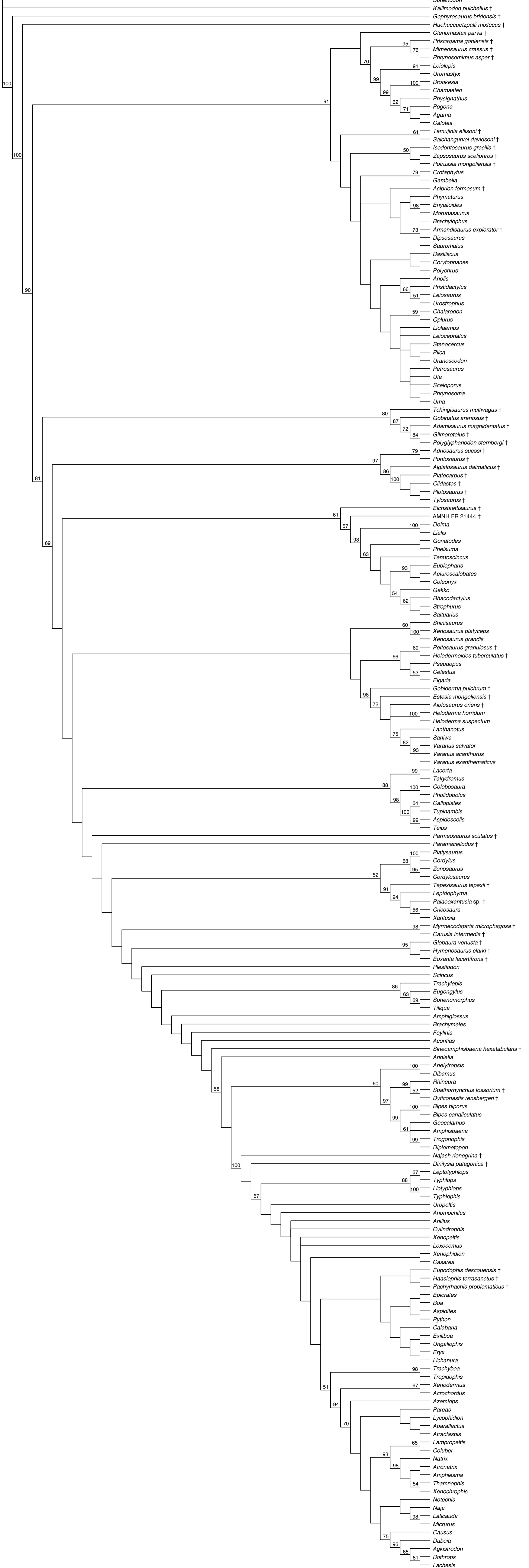

Supplement: S4 Fig — The phylogeny is a strict consensus of 384 trees (length = 6088 steps). Numbers at nodes are bootstrap support values >50%. Selected multi-state characters are ordered. Daggers indicate fossil taxa. (PDF) [file pone.0118199.s006.pdf]

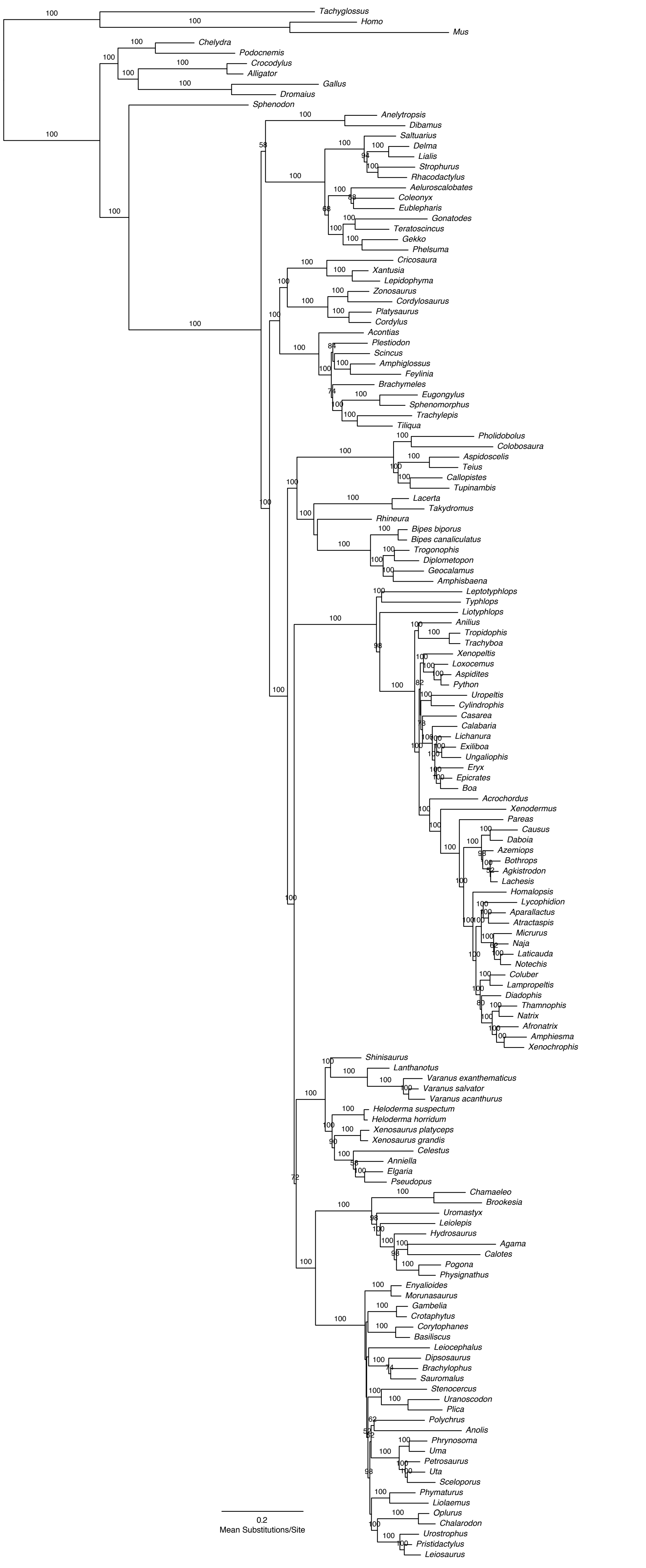

Supplement: S5 Fig — Numbers at nodes are bootstrap support values >50%. (PDF) [file pone.0118199.s007.pdf]

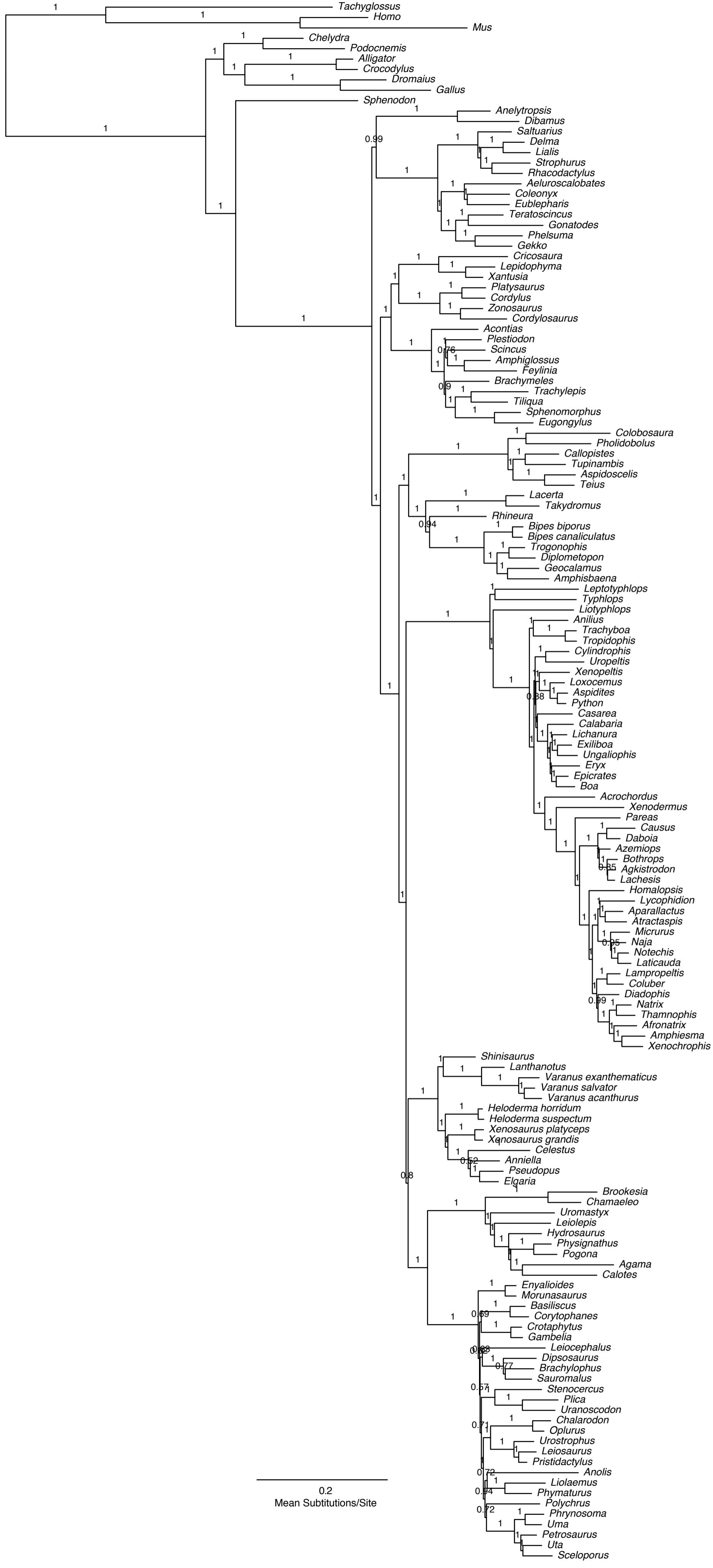

0.2  
Mean Substitutions/Site

Supplement: S6 Fig — Numbers along branches are posterior probabilities. (PDF) [file pone.0118199.s008.pdf]

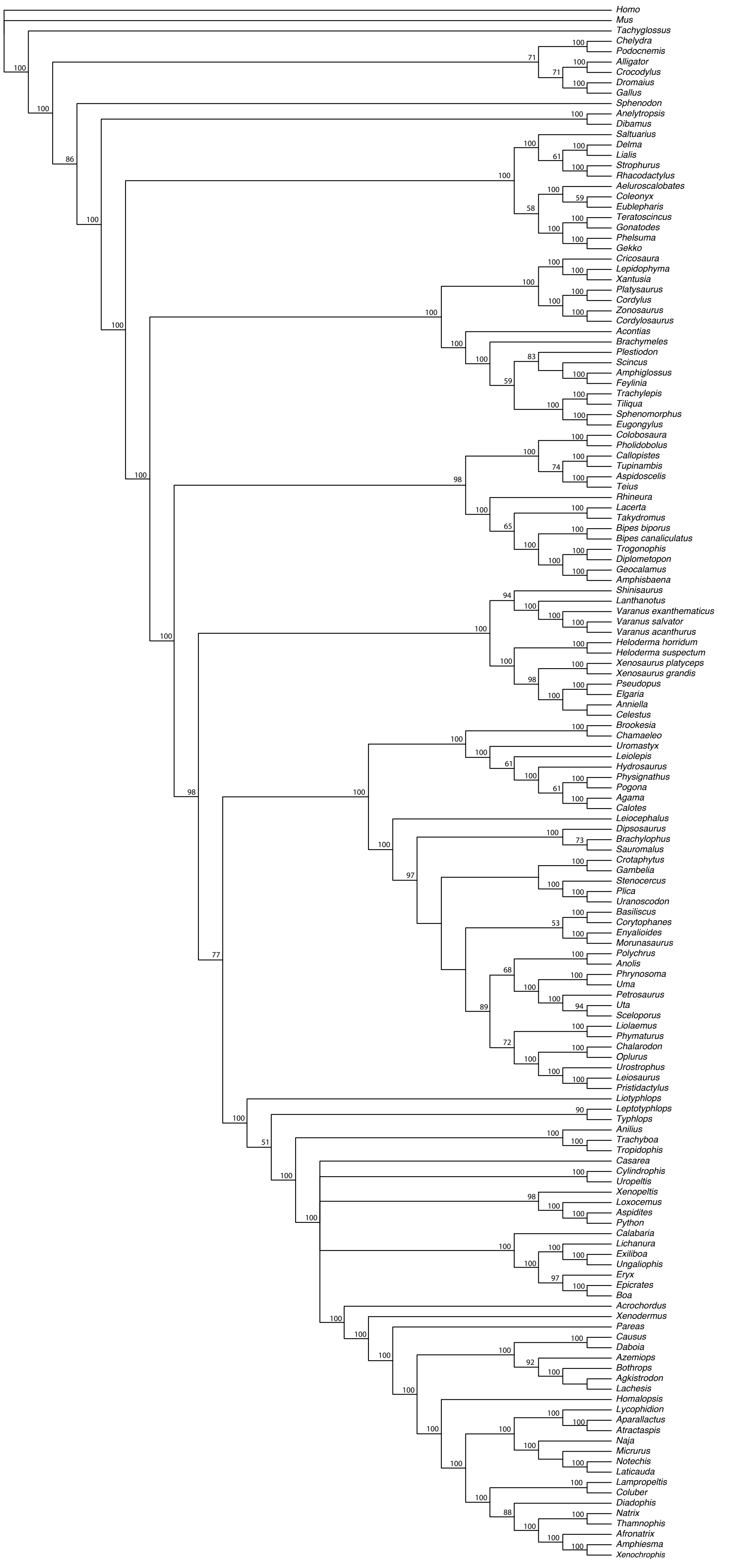

Supplement: S7 Fig — The phylogeny is a strict consensus of three trees (length = 197,520 steps). Numbers along branches are bootstrap support values >50%. (PDF) [file pone.0118199.s009.pdf]

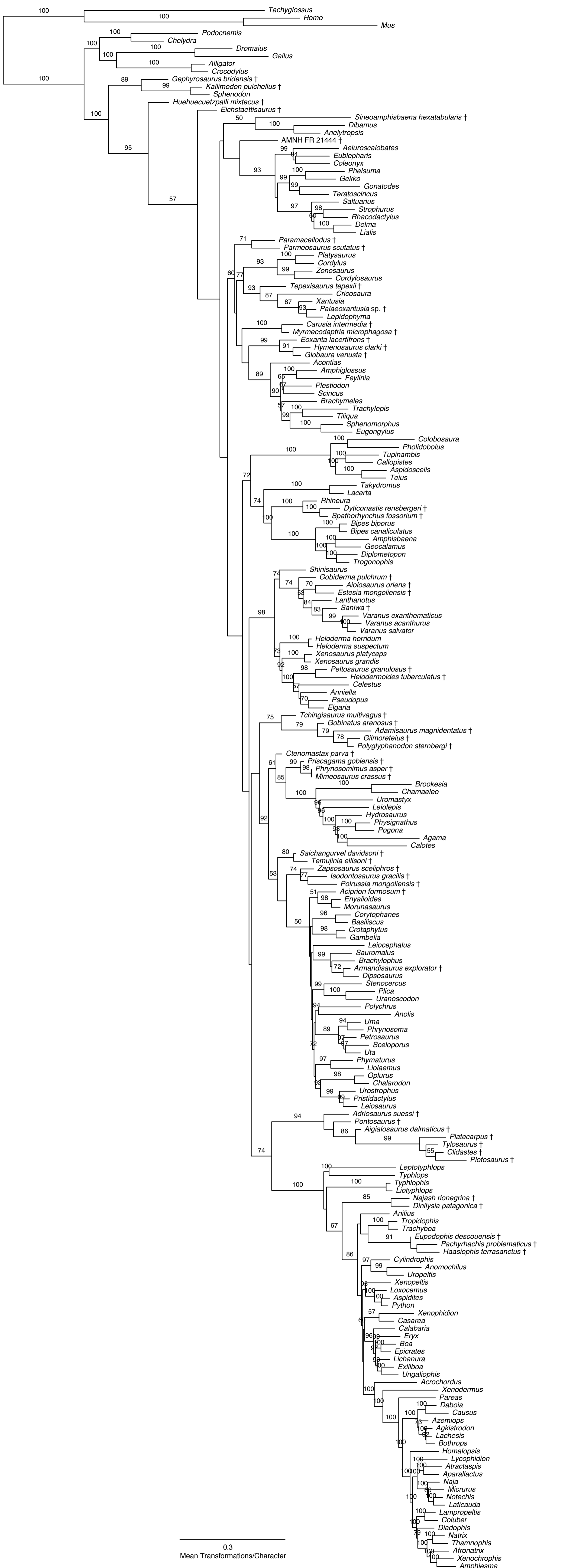

Supplement: S8 Fig — Numbers along branches are bootstrap support values >50%. Daggers indicate fossil taxa. (PDF) [file pone.0118199.s010.pdf]

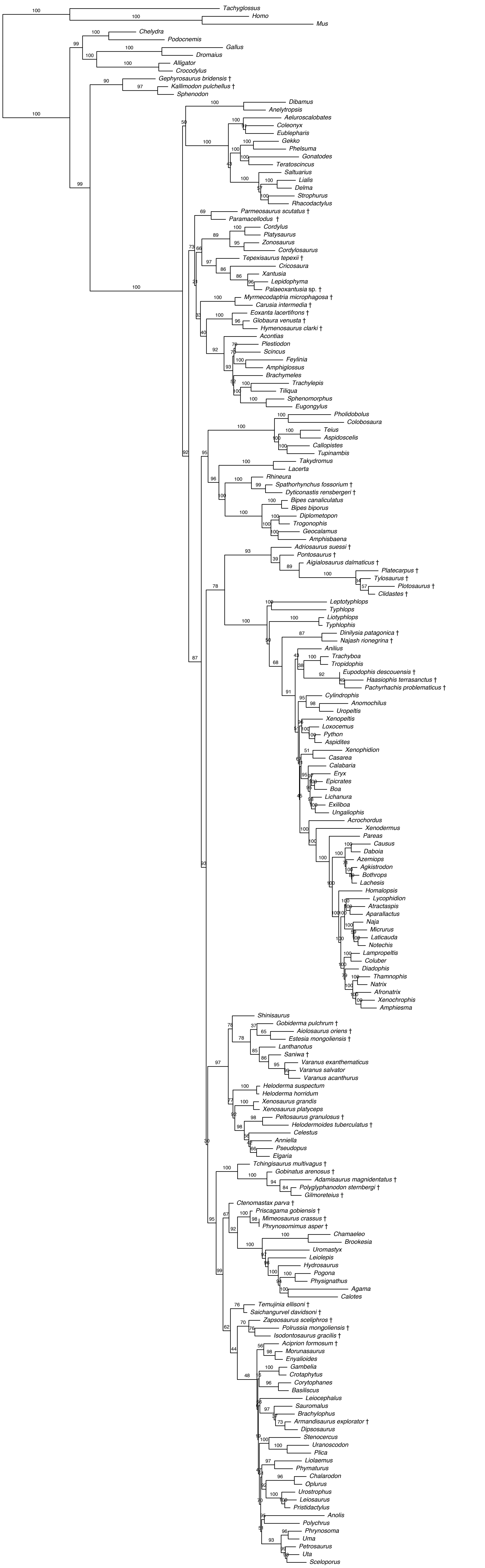

Supplement: S9 Fig — Numbers along branches are bootstrap support values >50%. This tree is the same as in Fig. 1, but includes bootstrap values for all branches. Daggers indicate fossil taxa. (PDF) [file pone.0118199.s011.pdf]

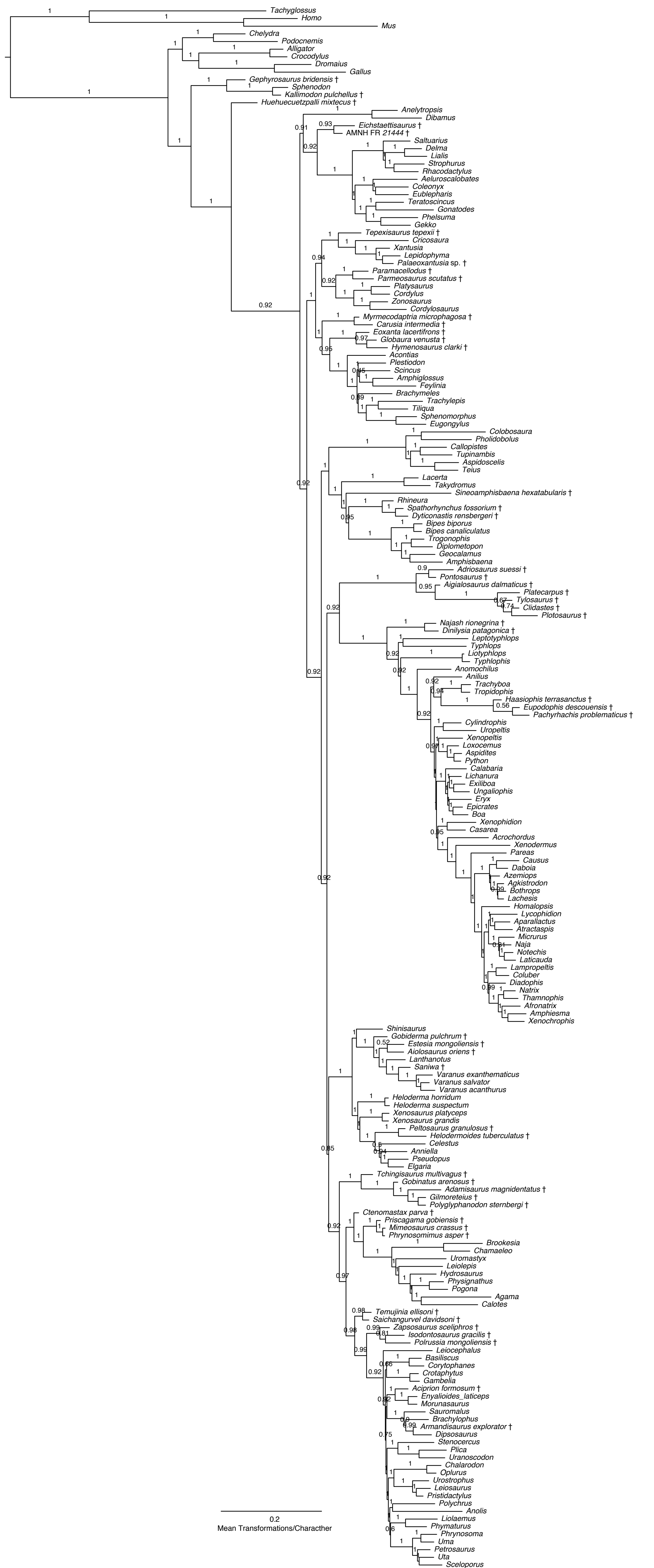

Supplement: S10 Fig — Numbers along branches are posterior probabilities. Selected multi-state characters are ordered. Daggers indicate fossil taxa. (PDF) [file pone.0118199.s012.pdf]

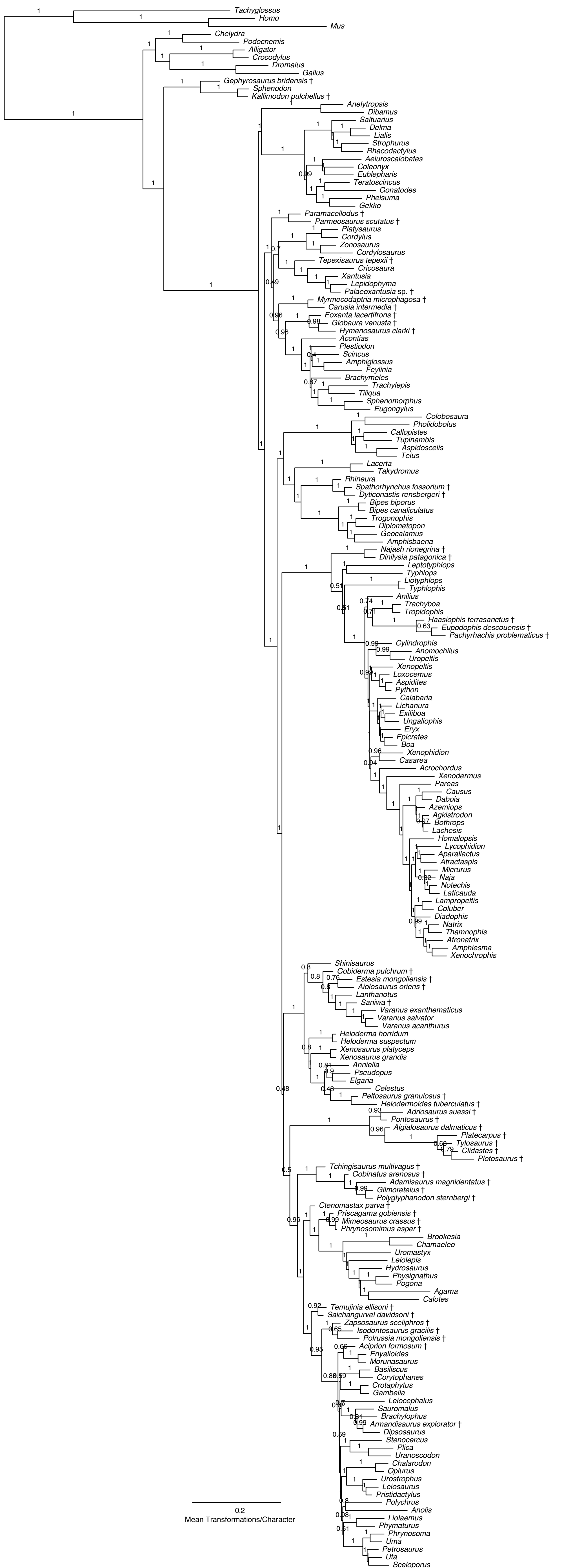

Supplement: S11 Fig — Numbers along branches are posterior probabilities. Selected multi-state characters are ordered. Daggers indicate fossil taxa. (PDF) [file pone.0118199.s013.pdf]

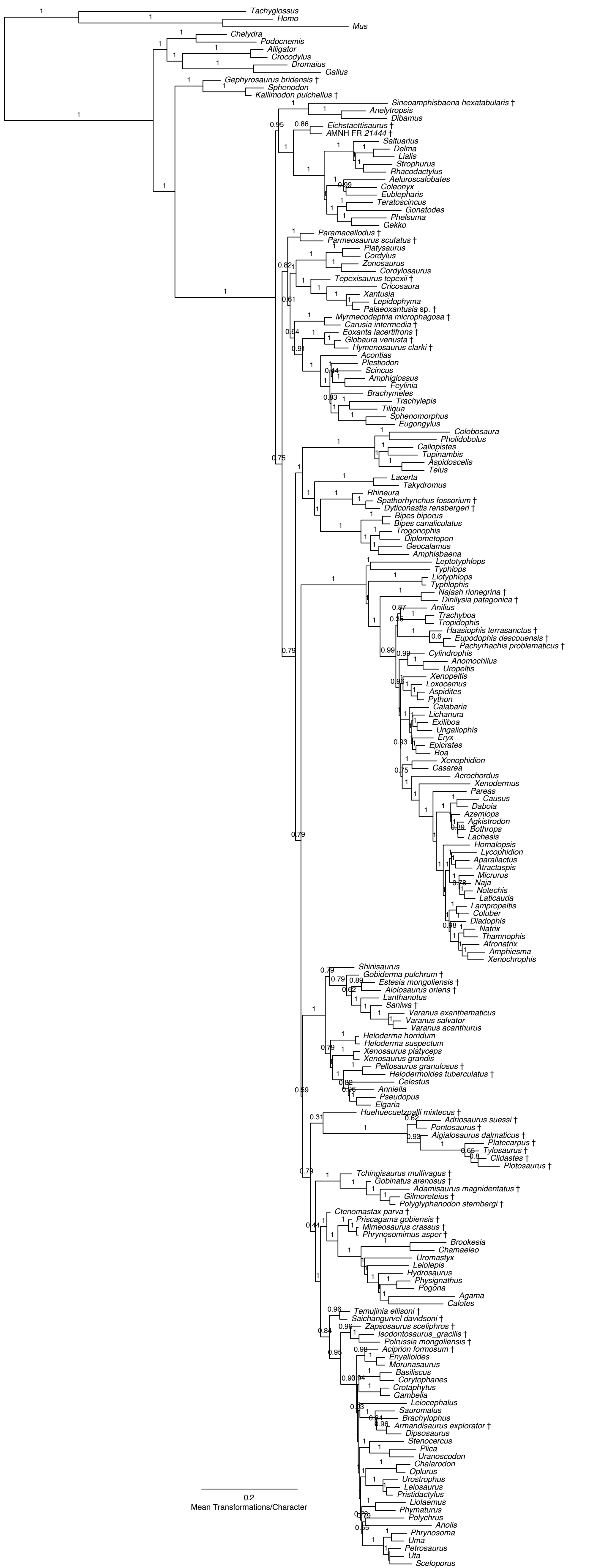

Supplement: S12 Fig — Numbers along branches are posterior probabilities. All multi-state characters are unordered. Daggers indicate fossil taxa. (PDF) [file pone.0118199.s014.pdf]

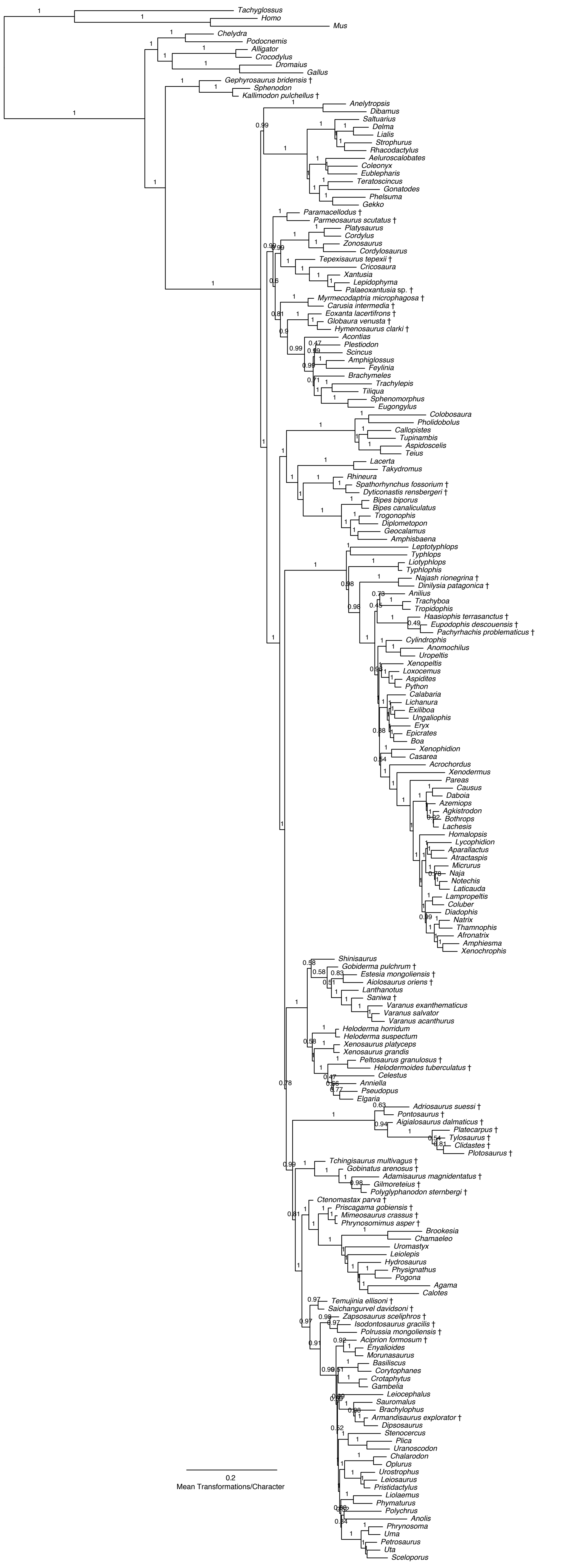

Supplement: S13 Fig — Numbers along branches are posterior probabilities. All multi-state characters are unordered. Daggers indicate fossil taxa. (PDF) [file pone.0118199.s015.pdf]

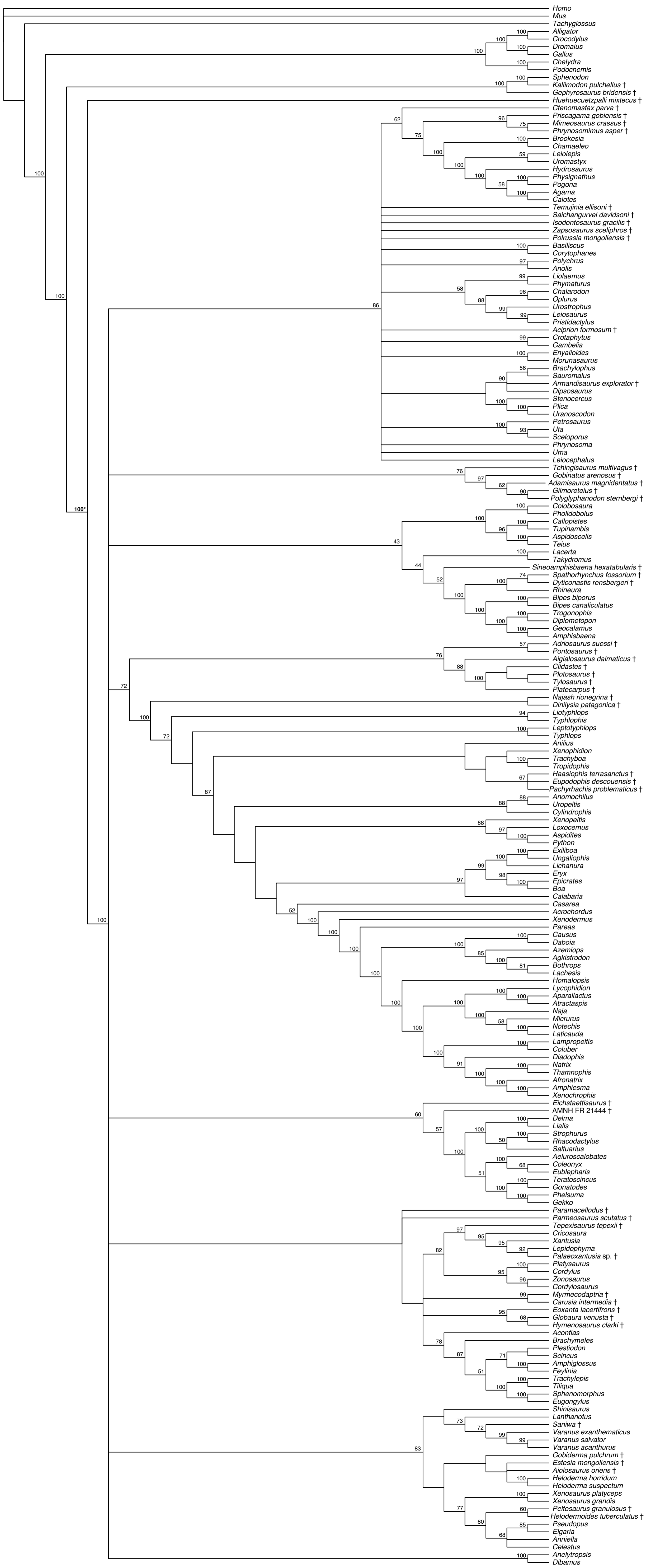

Supplement: S14 Fig — The phylogeny is a strict consensus of 1344 trees (length = 207,375 steps). Numbers at nodes are bootstrap support values >50%. Daggers indicate fossil taxa. (PDF) [file pone.0118199.s016.pdf]

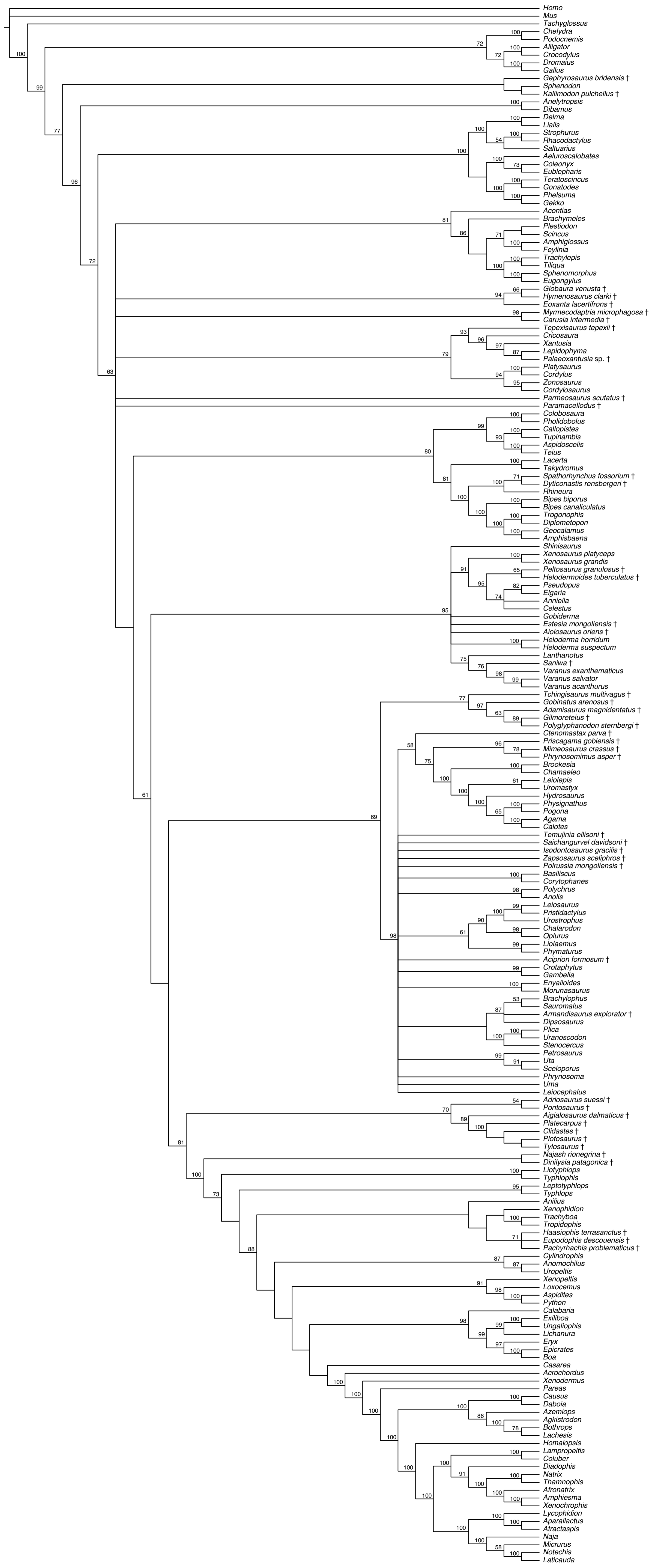

Supplement: S15 Fig — The phylogeny is a strict consensus of 938 trees (length = 207,293 steps). Numbers at nodes are bootstrap support values >50%. Daggers indicate fossil taxa. (PDF) [file pone.0118199.s017.pdf]

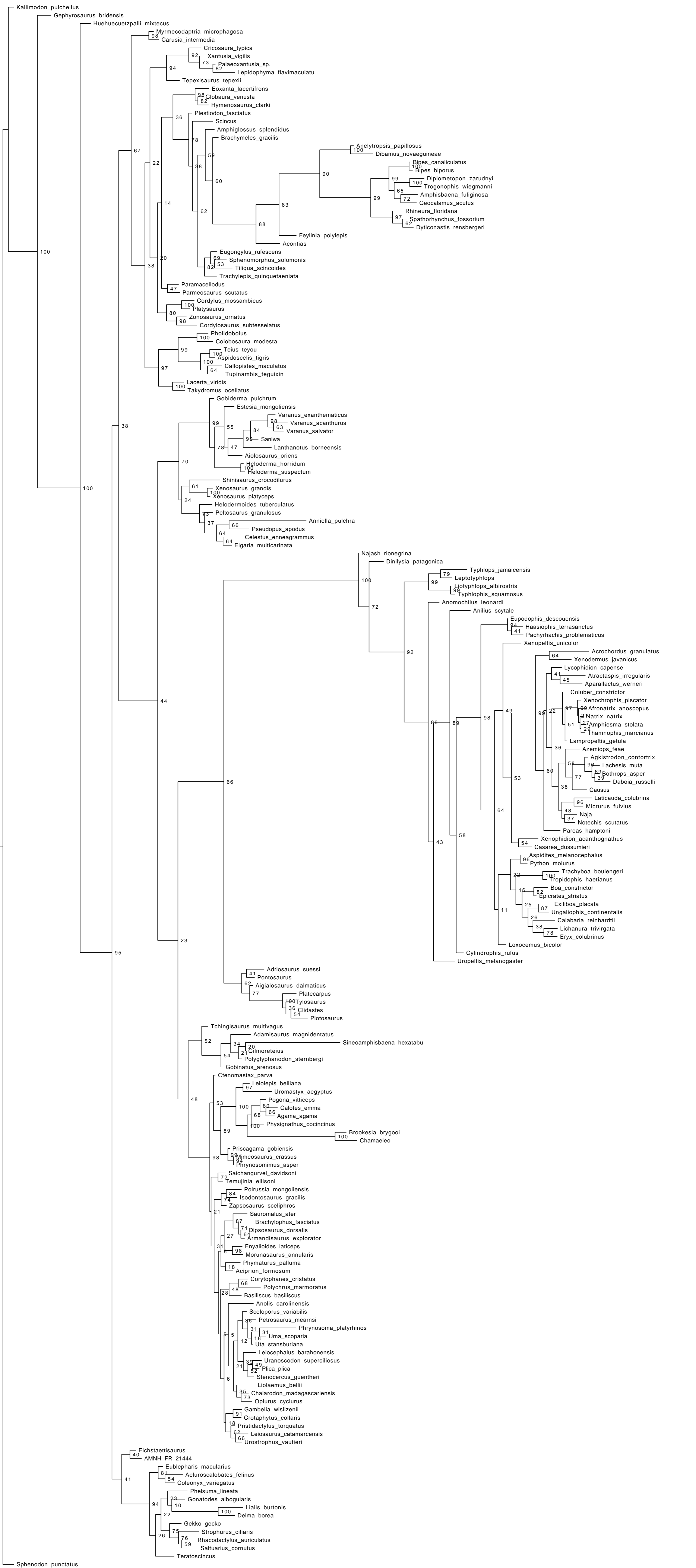

Supplement: S16 Fig — (PDF) [file pone.0118199.s018.pdf]

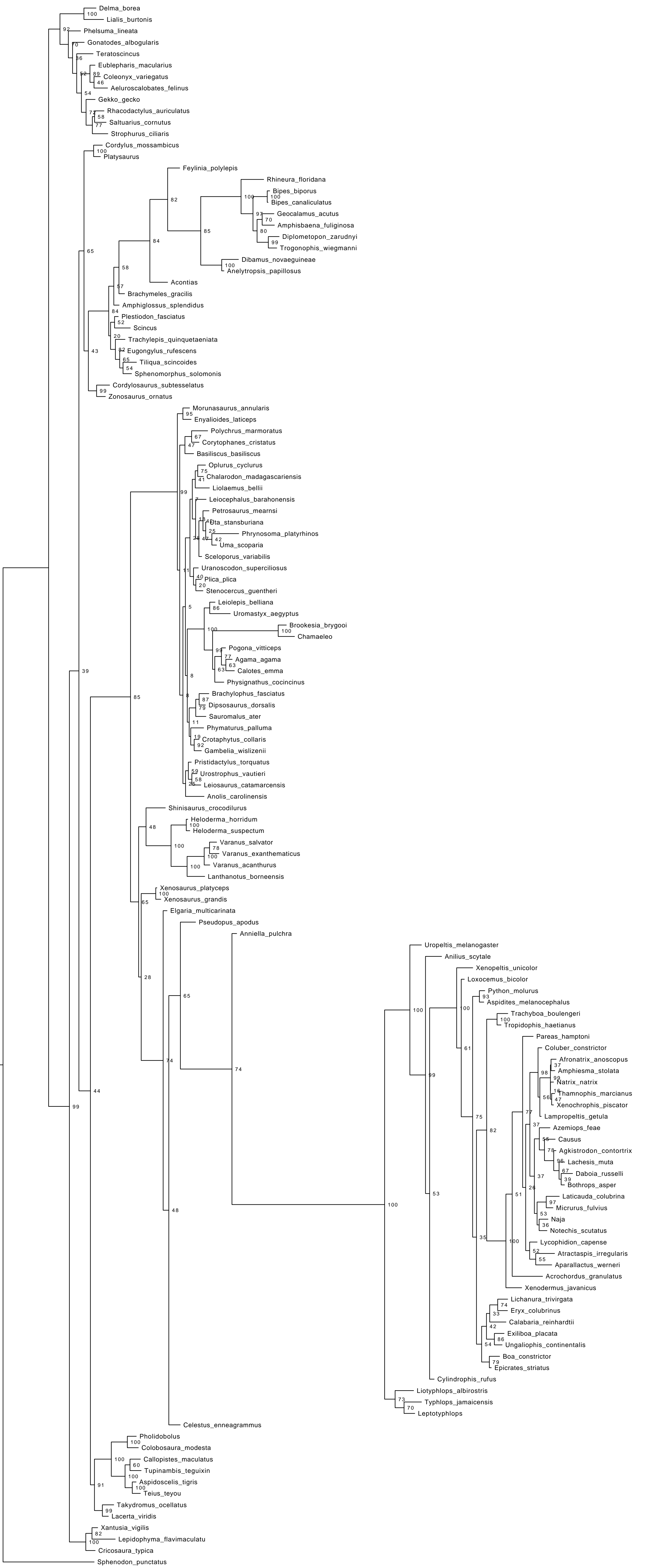

Supplement: S17 Fig — (PDF) [file pone.0118199.s019.pdf]

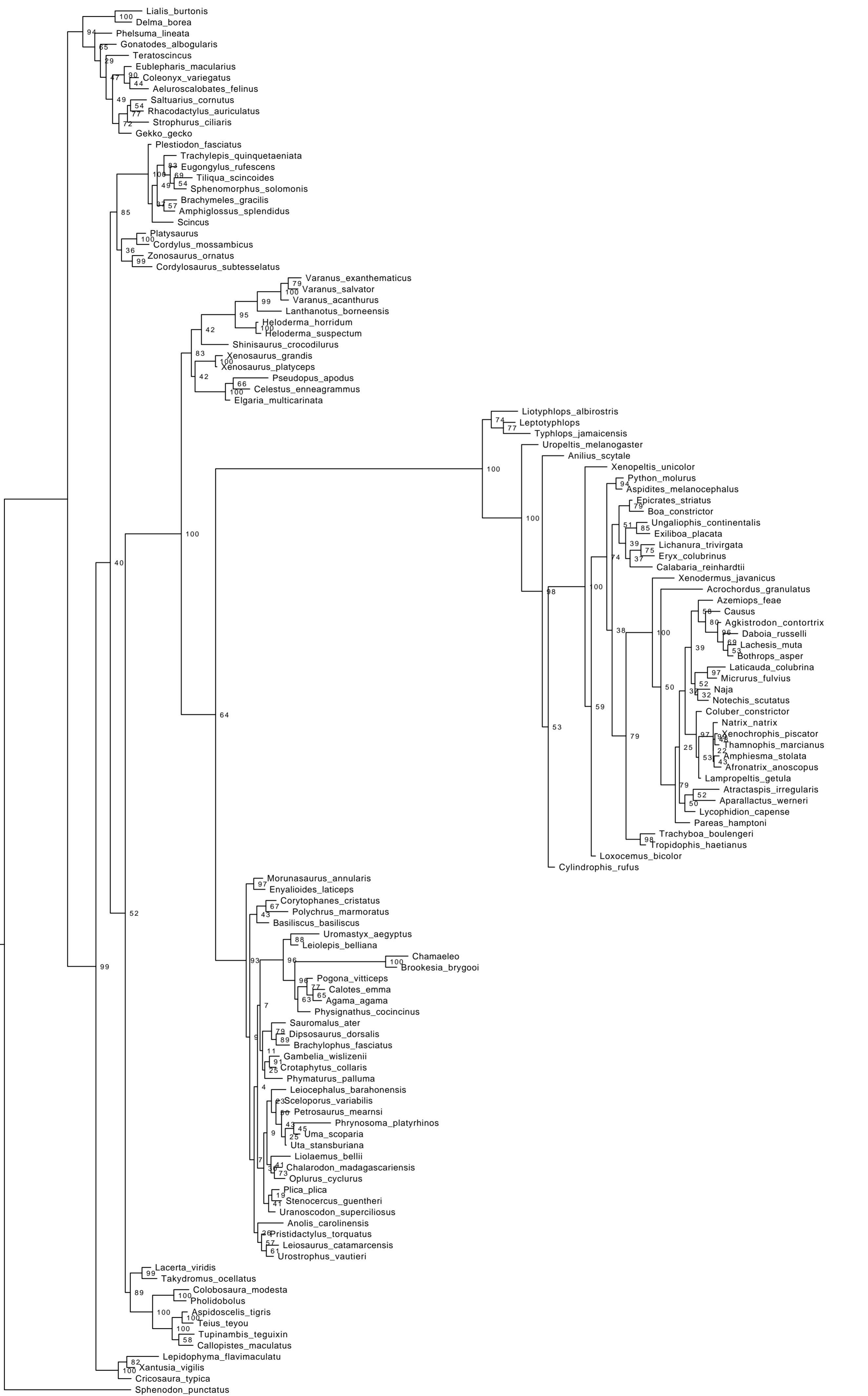

Supplement: S18 Fig — (PDF) [file pone.0118199.s020.pdf]

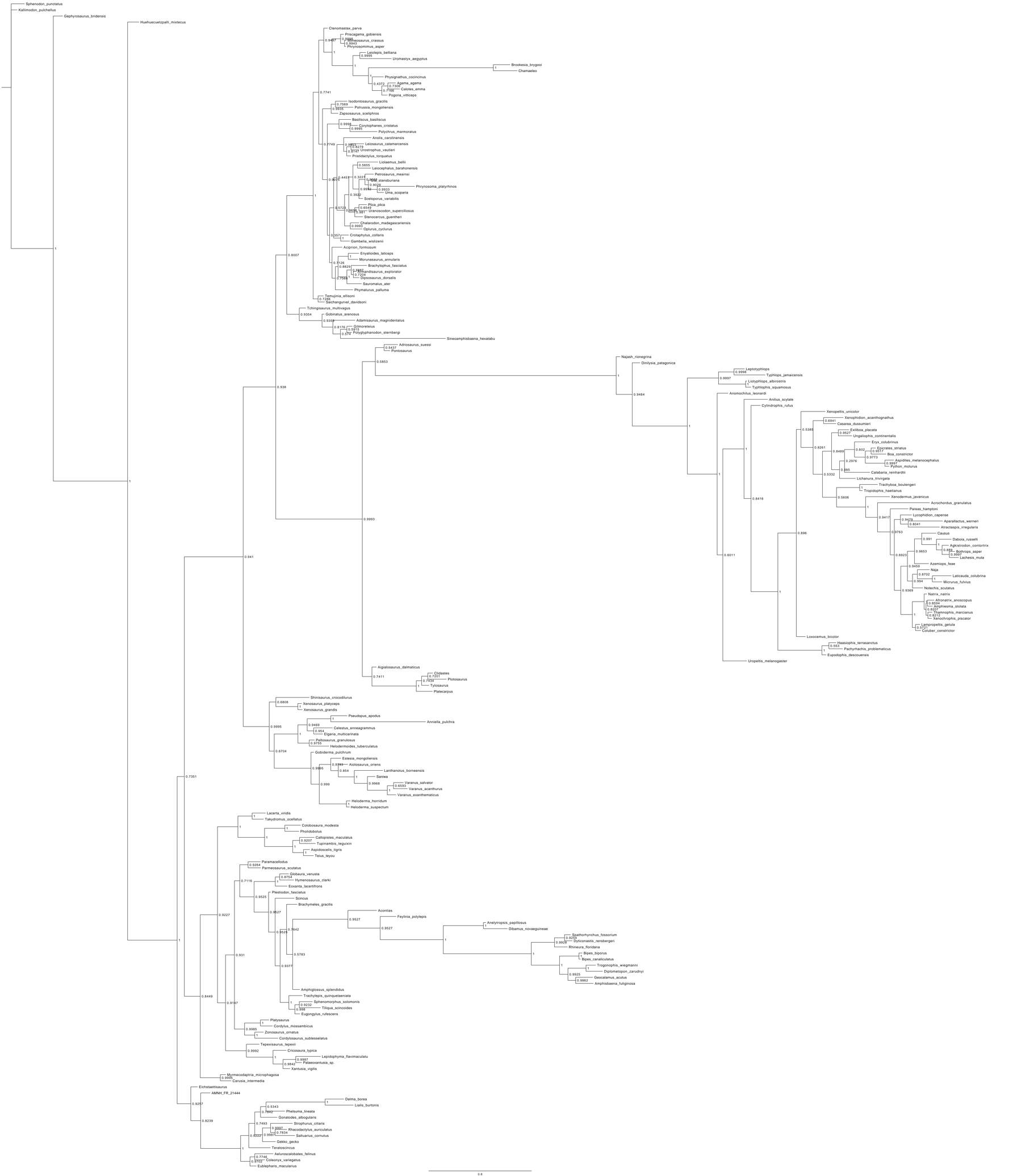

Supplement: S19 Fig — (PDF) [file pone.0118199.s021.pdf]

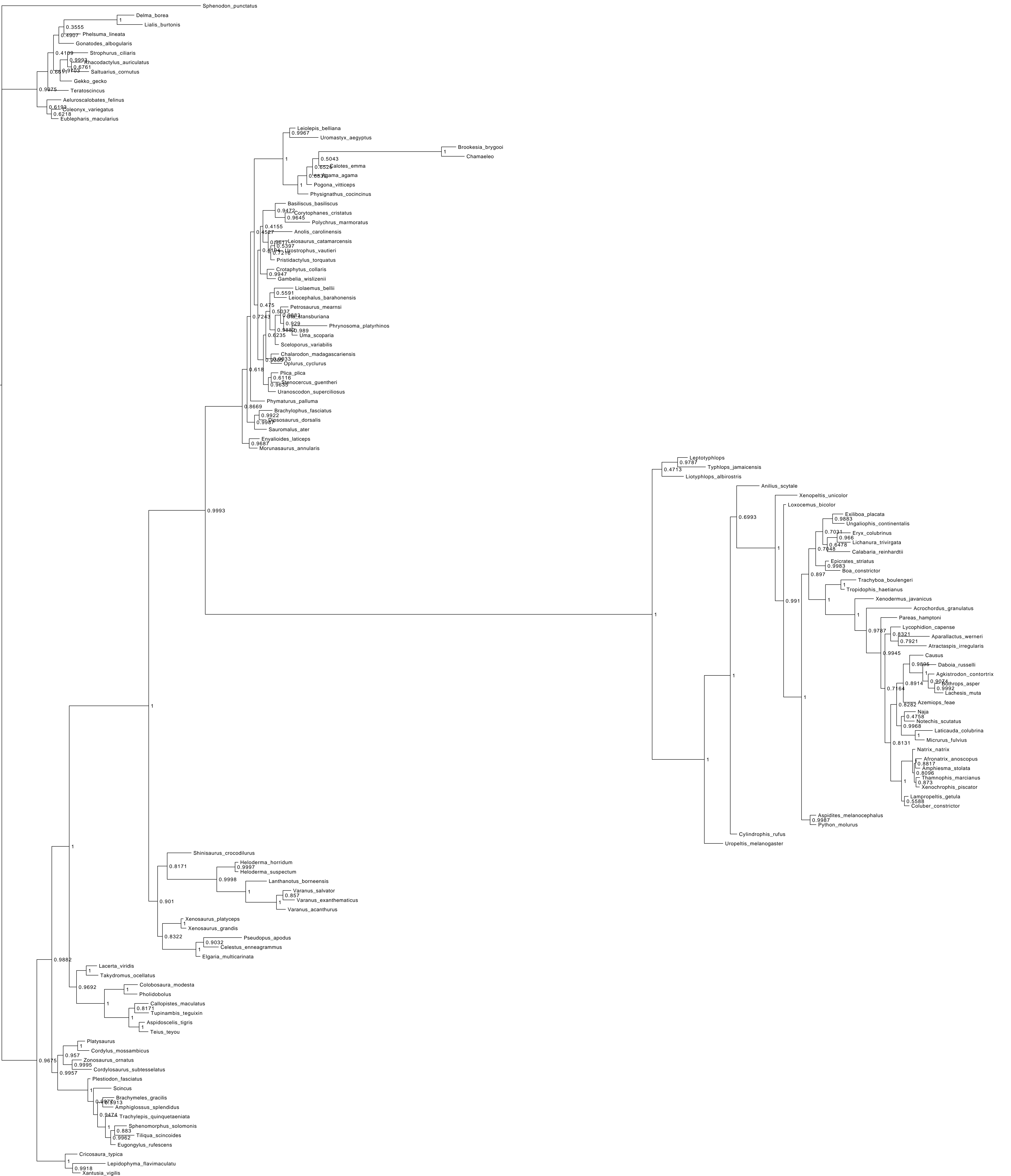

Supplement: S21 Fig — (PDF) [file pone.0118199.s023.pdf]

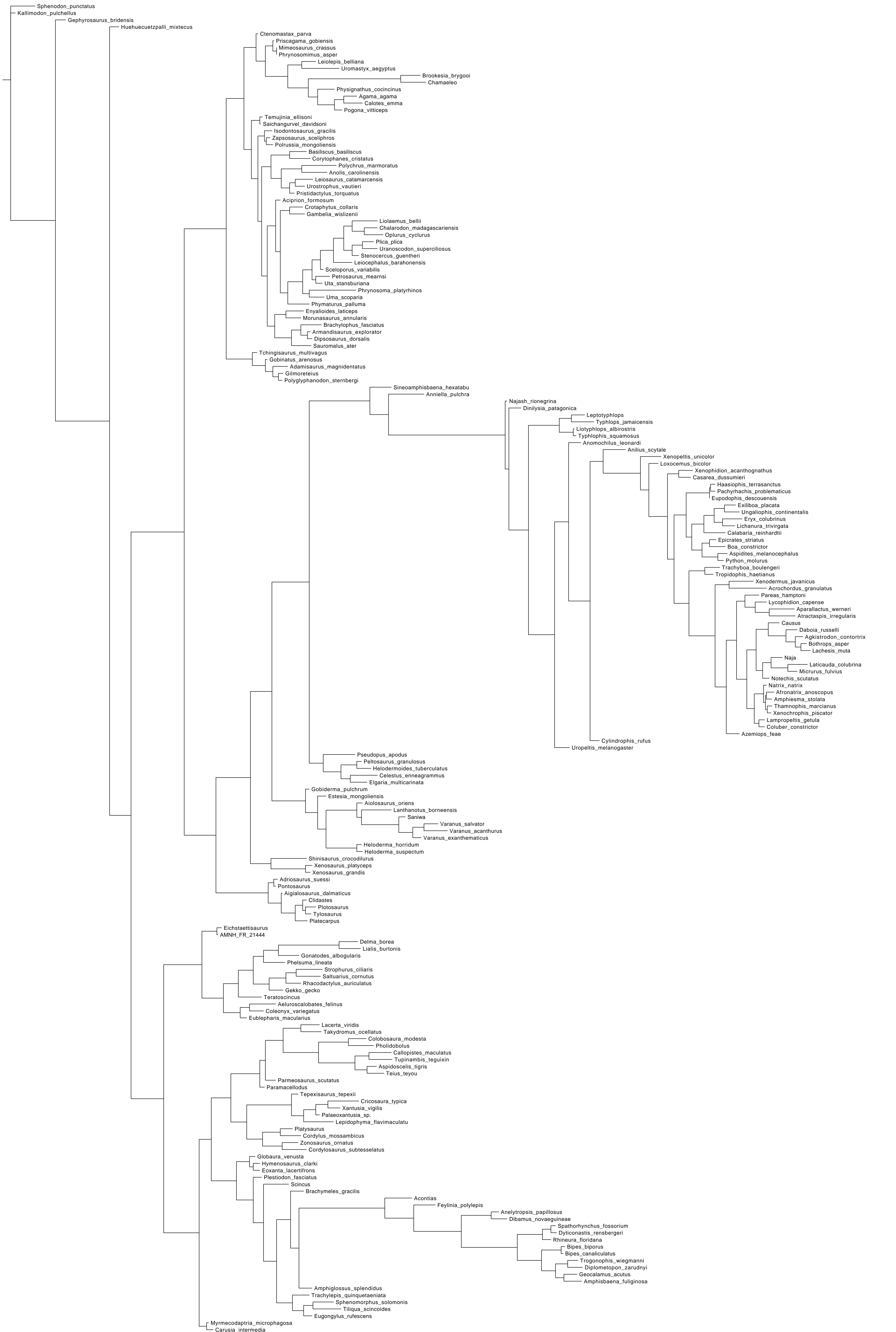

Supplement: S22 Fig — Strict consensus of 1274 shortest trees of length 6277. See S25 Fig. for bootstrap values. (PDF) [file pone.0118199.s024.pdf]

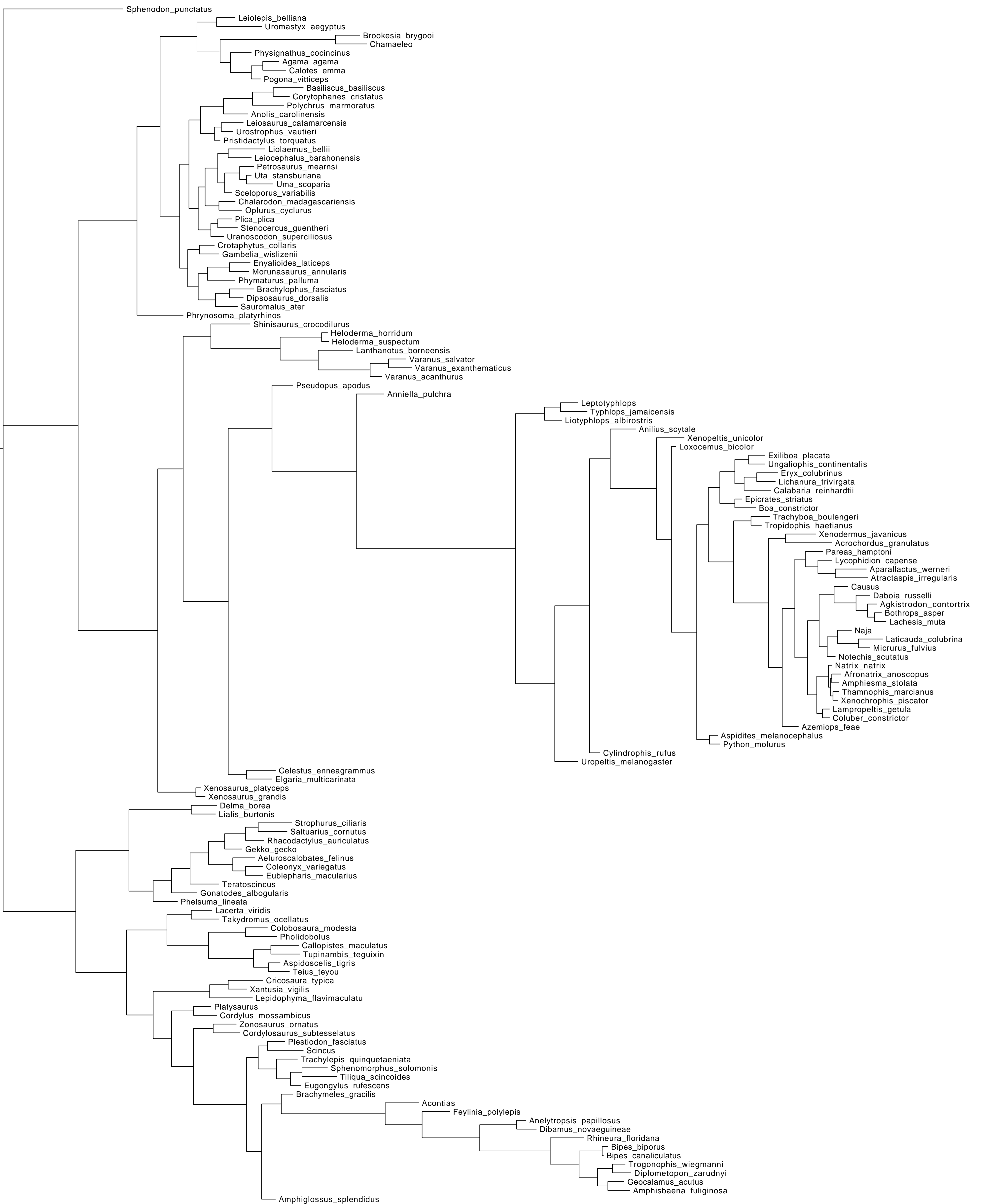

Supplement: S23 Fig — Strict consensus of 67 shortest trees of length 5437. See S26 Fig. for bootstrap values. (PDF) [file pone.0118199.s025.pdf]

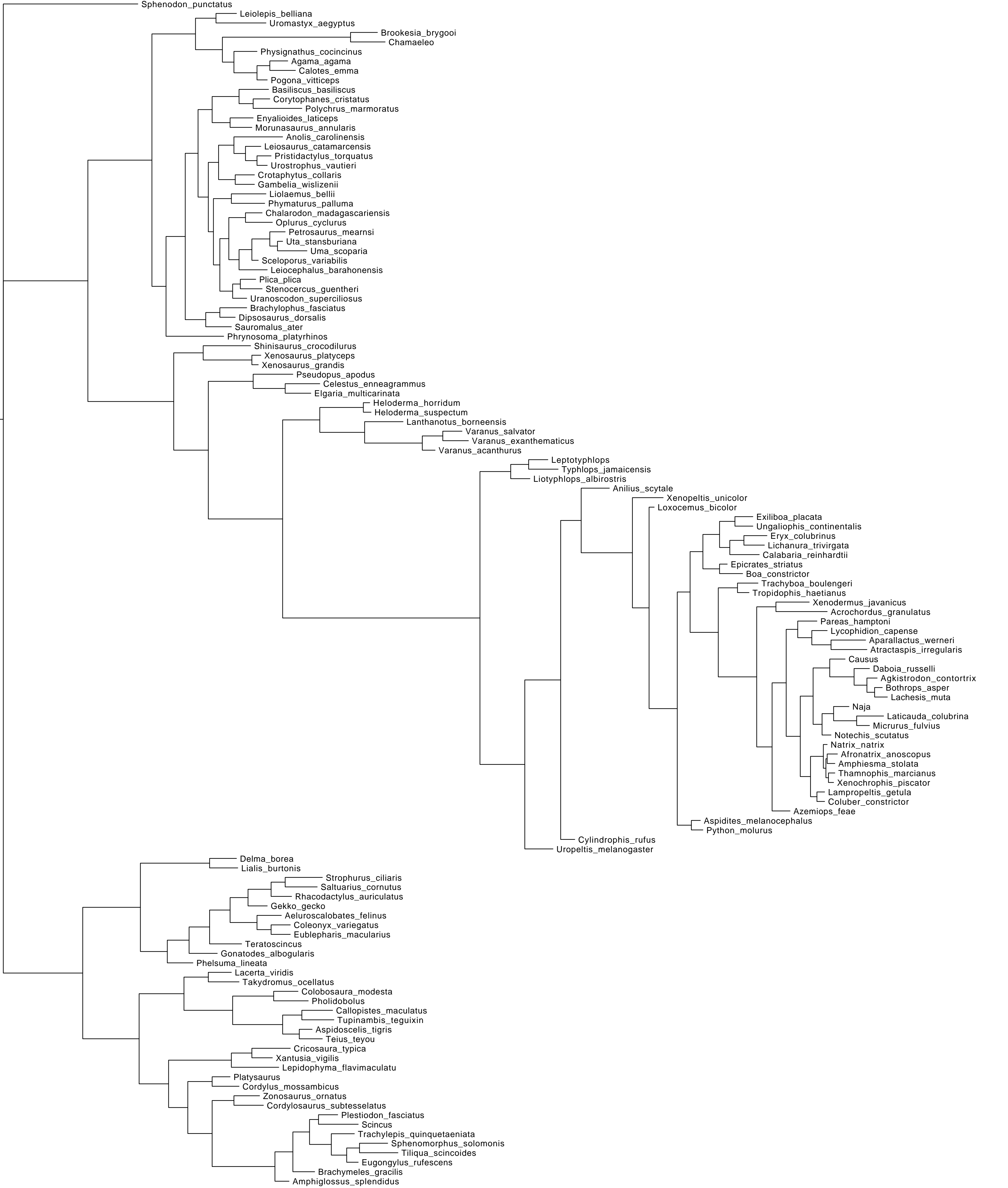

Supplement: S24 Fig — Strict consensus of 237 shortest trees of length 4845. See S27 Fig. for bootstrap values. (PDF) [file pone.0118199.s026.pdf]

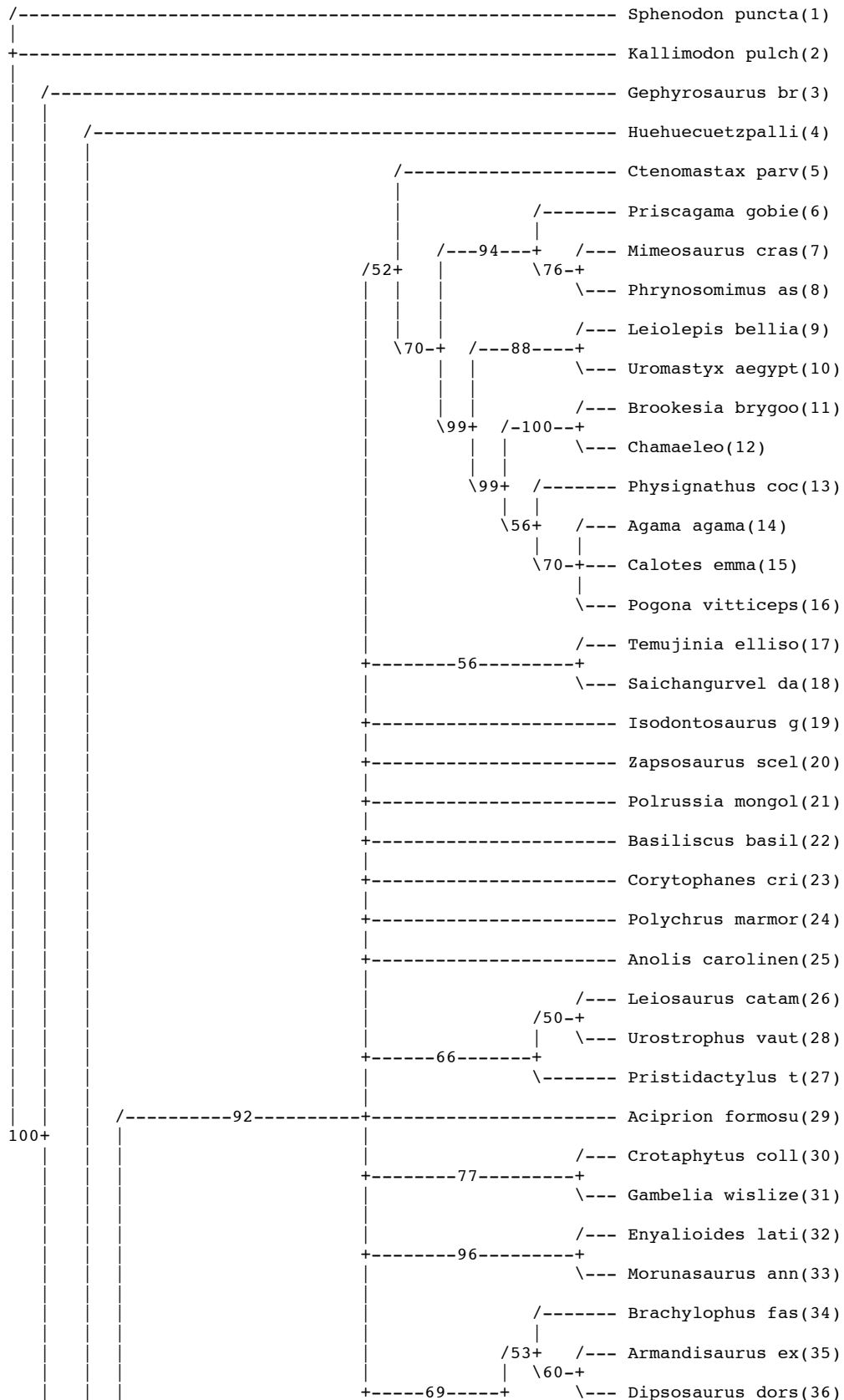

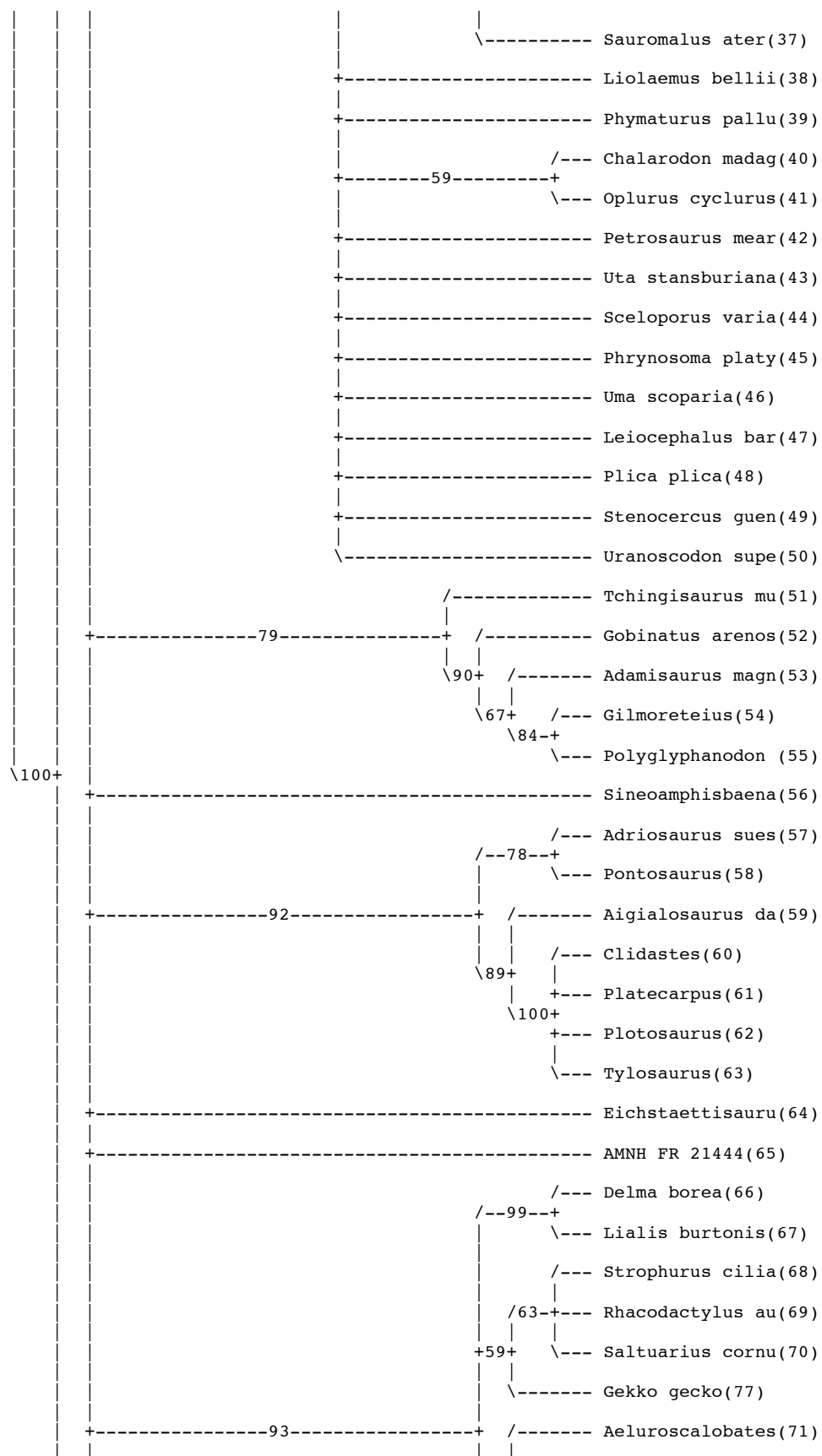

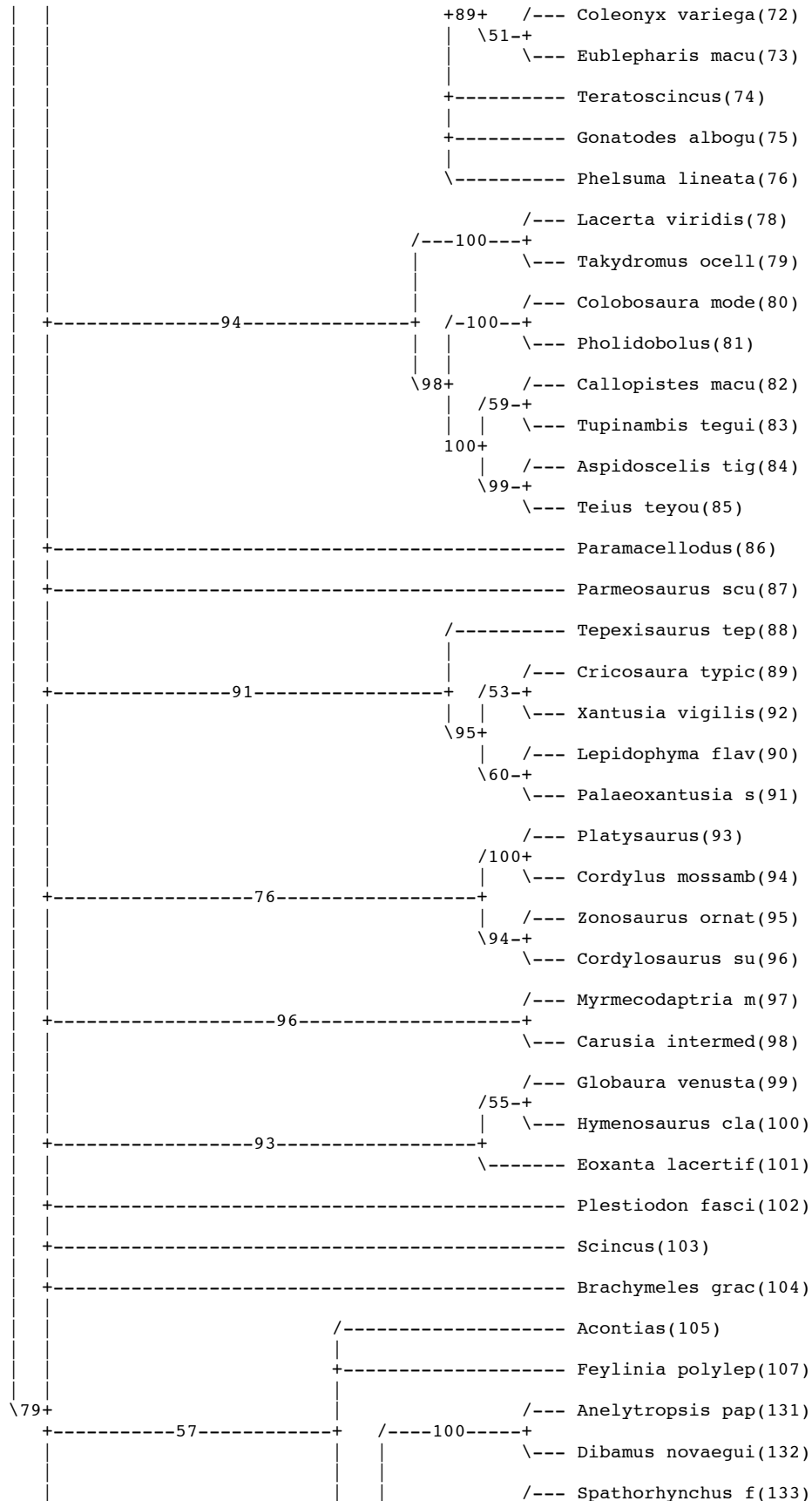

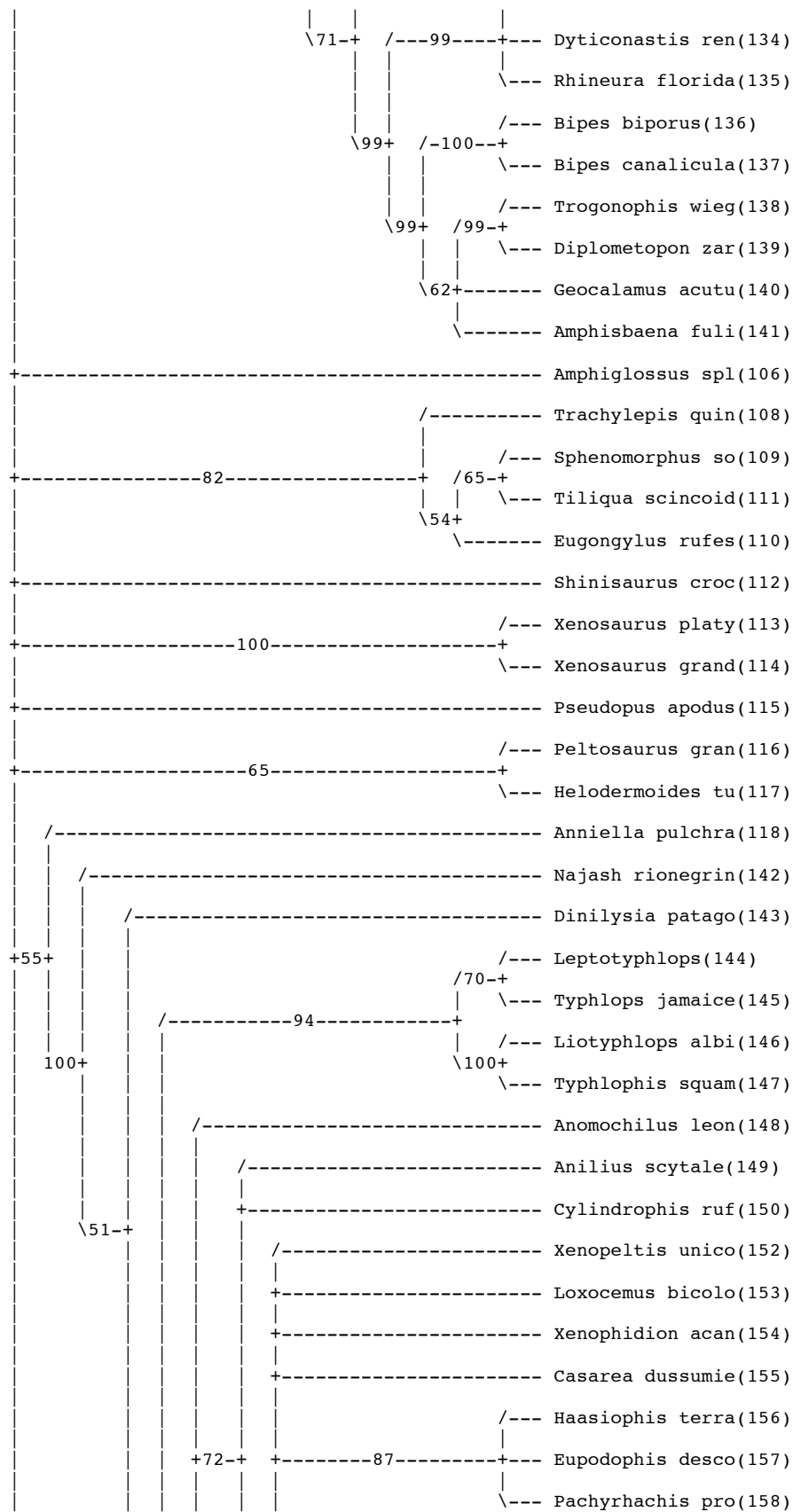

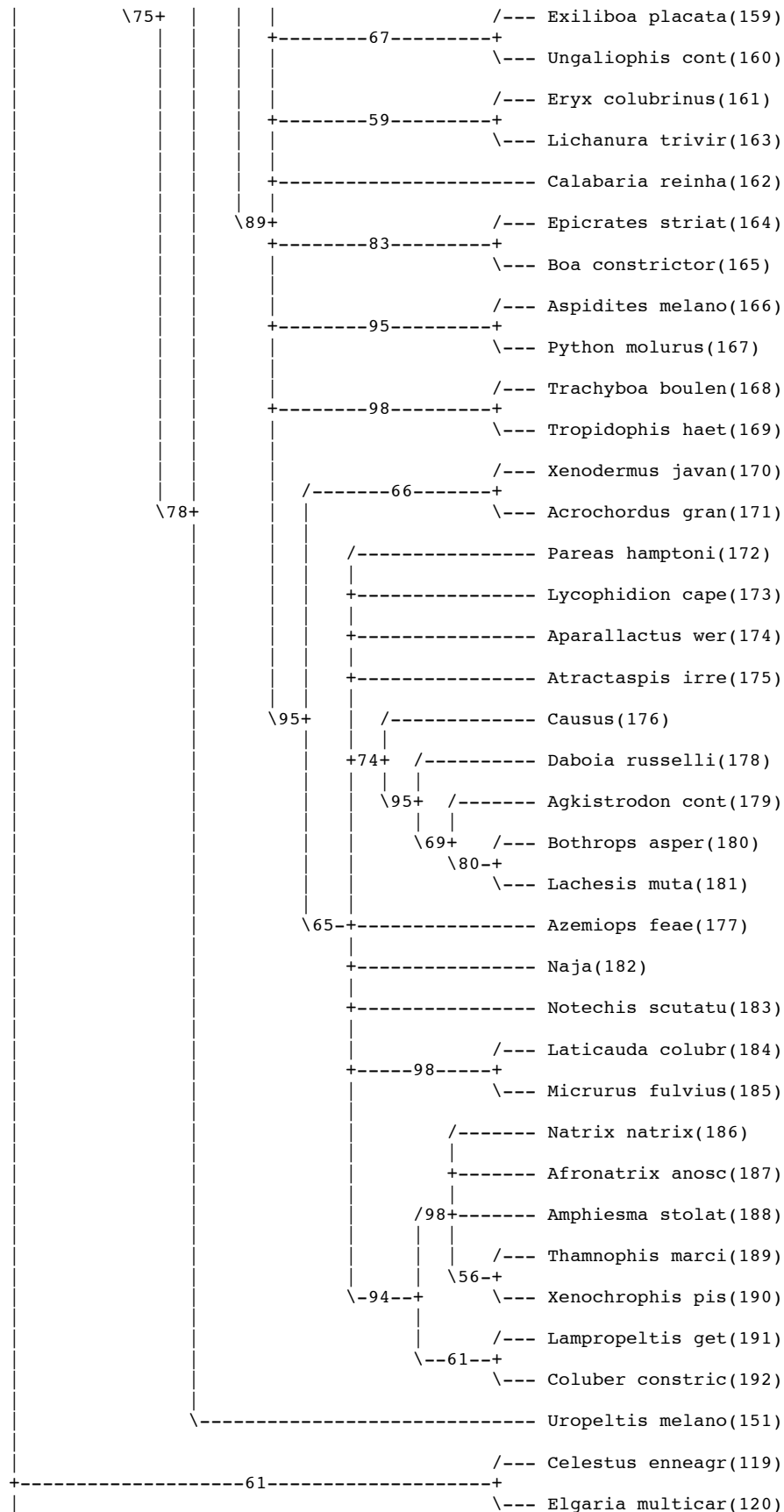

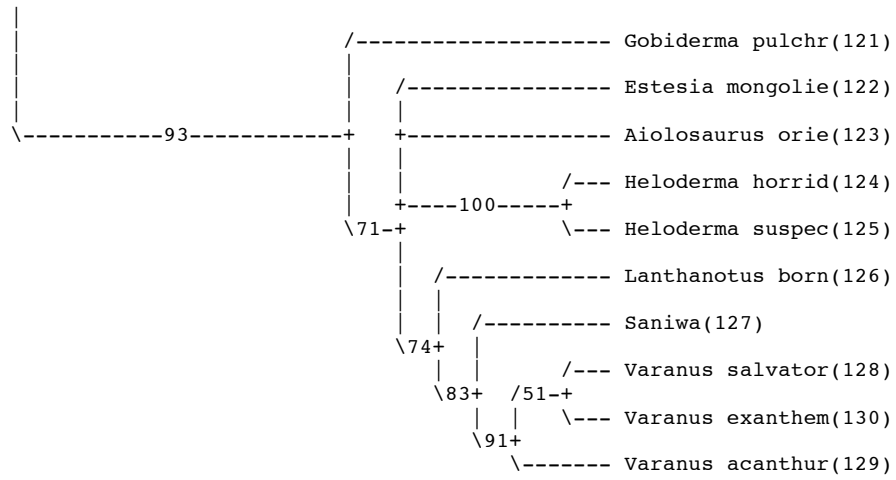

Supplement: S25 Fig — Bootstrap values less than 50% are not shown and the corresponding branch is collapsed. (PDF) [file pone.0118199.s027.pdf]

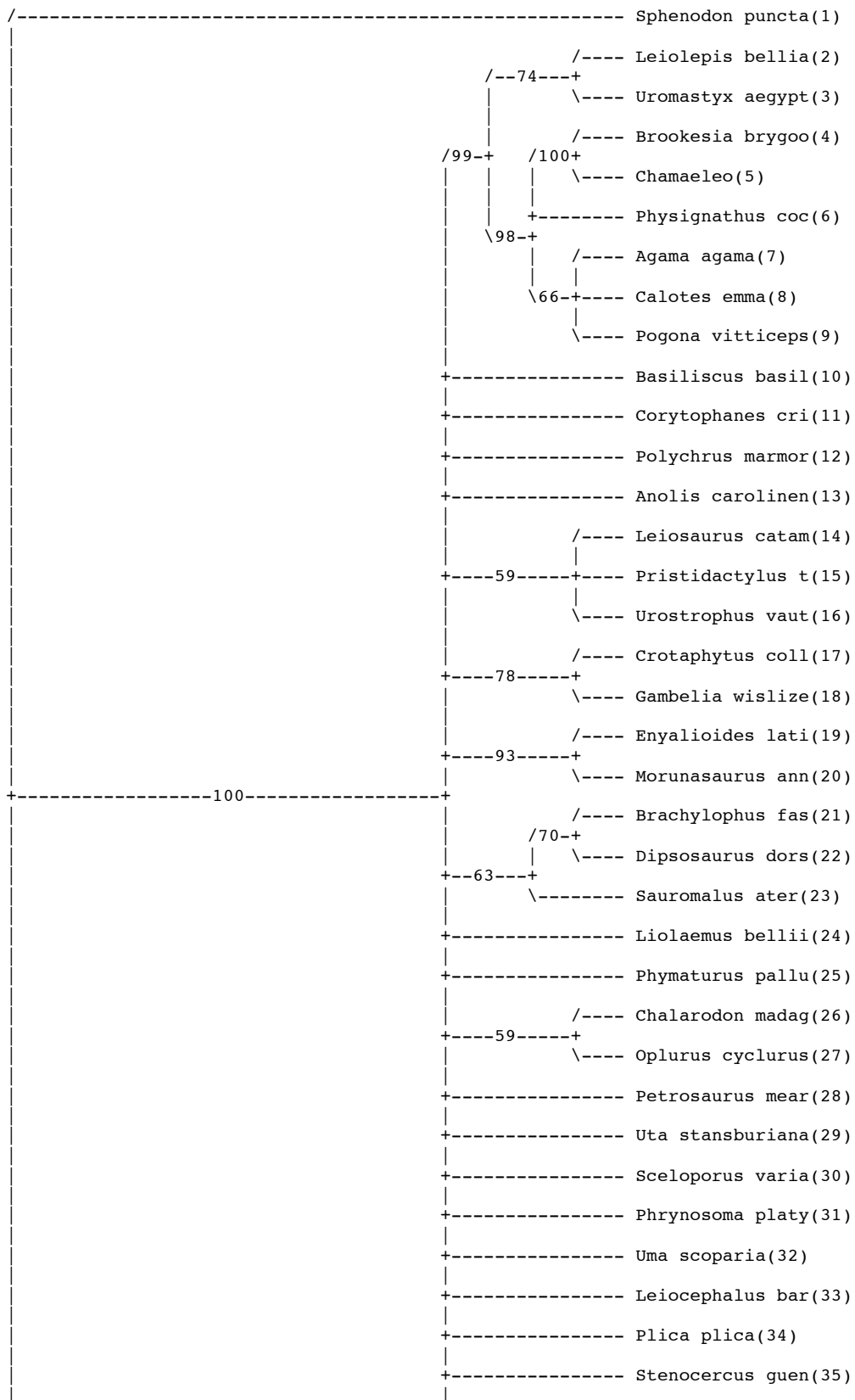

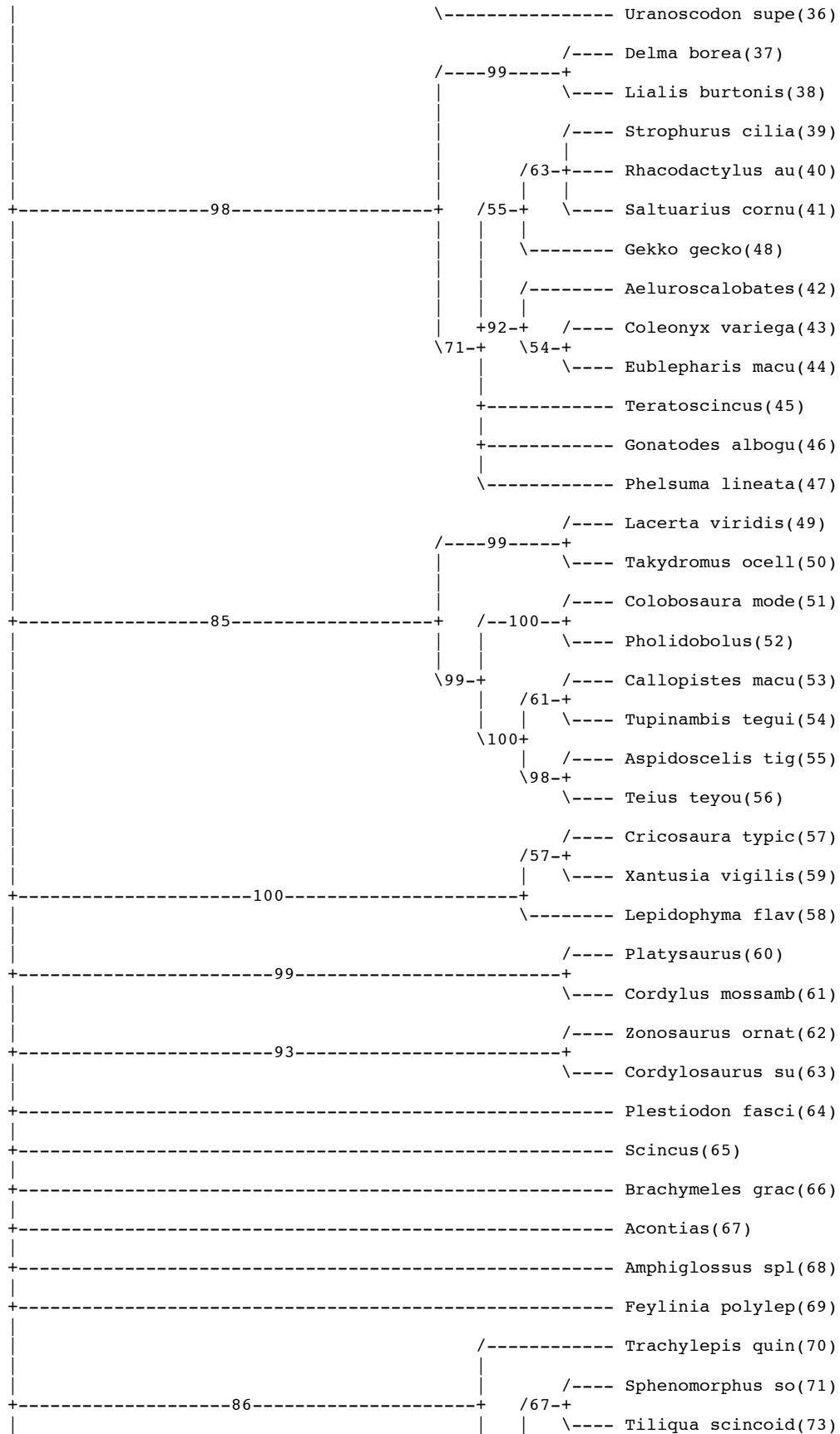

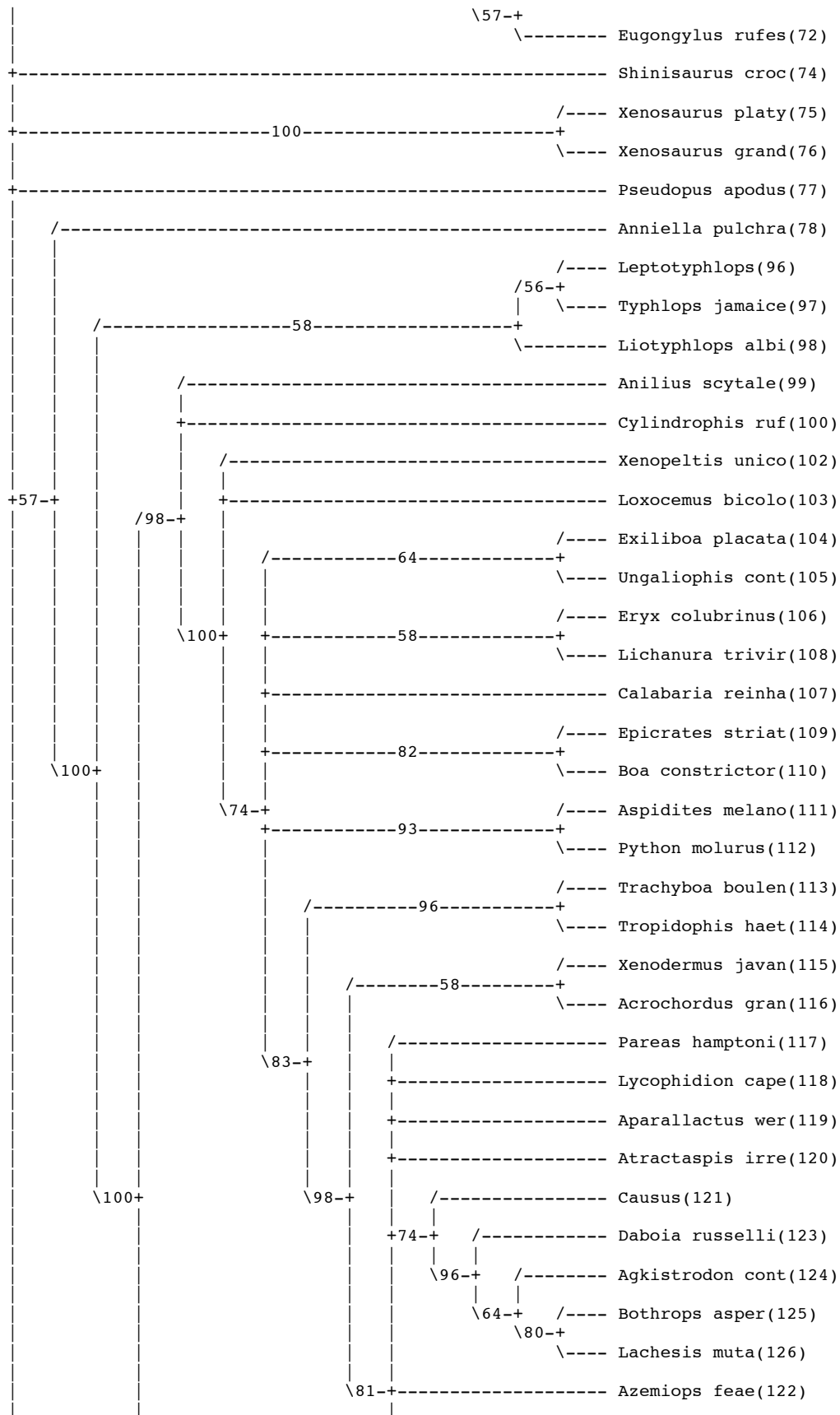

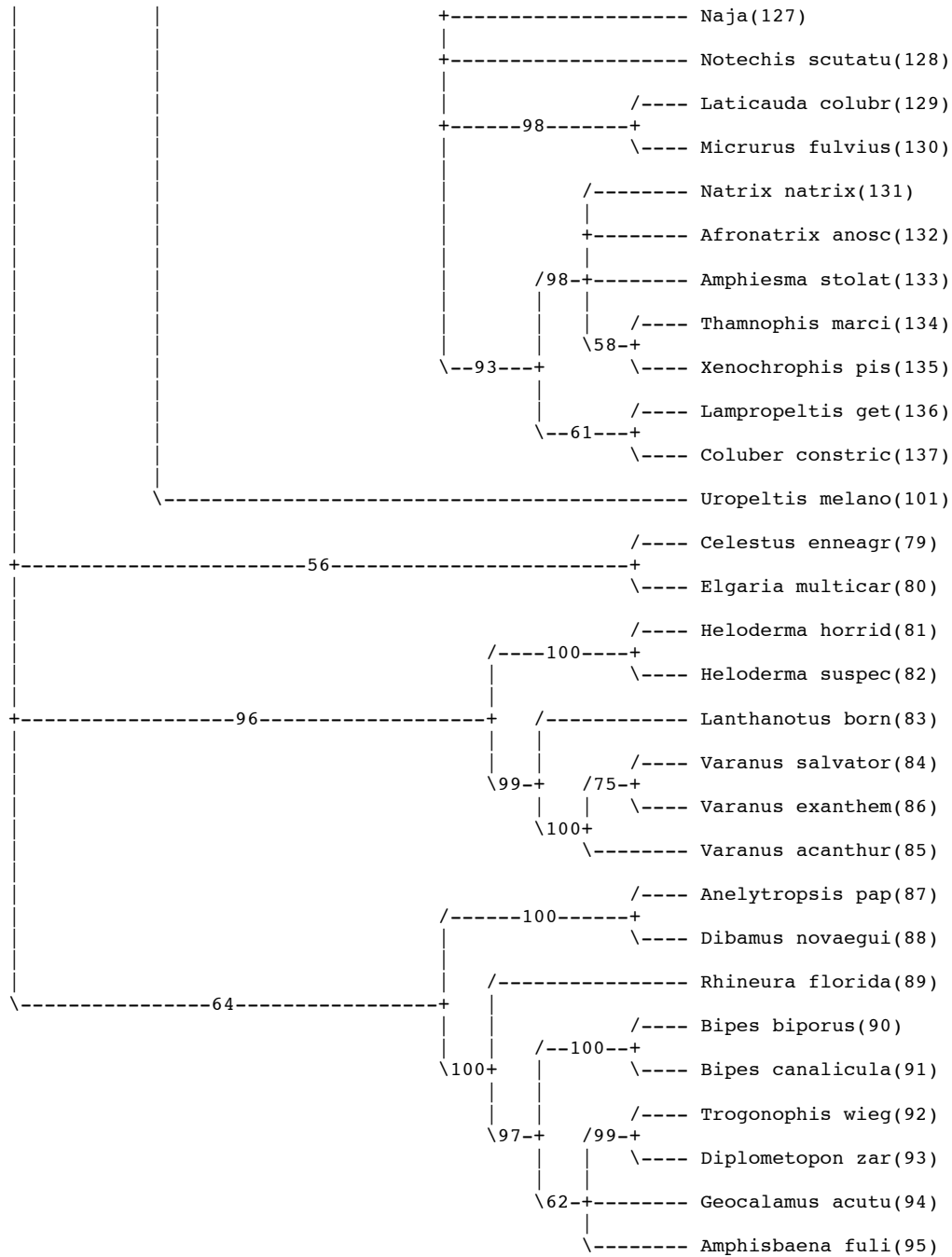

Supplement: S26 Fig — Bootstrap values less than 50% are not shown and the corresponding branch is collapsed. (PDF) [file pone.0118199.s028.pdf]

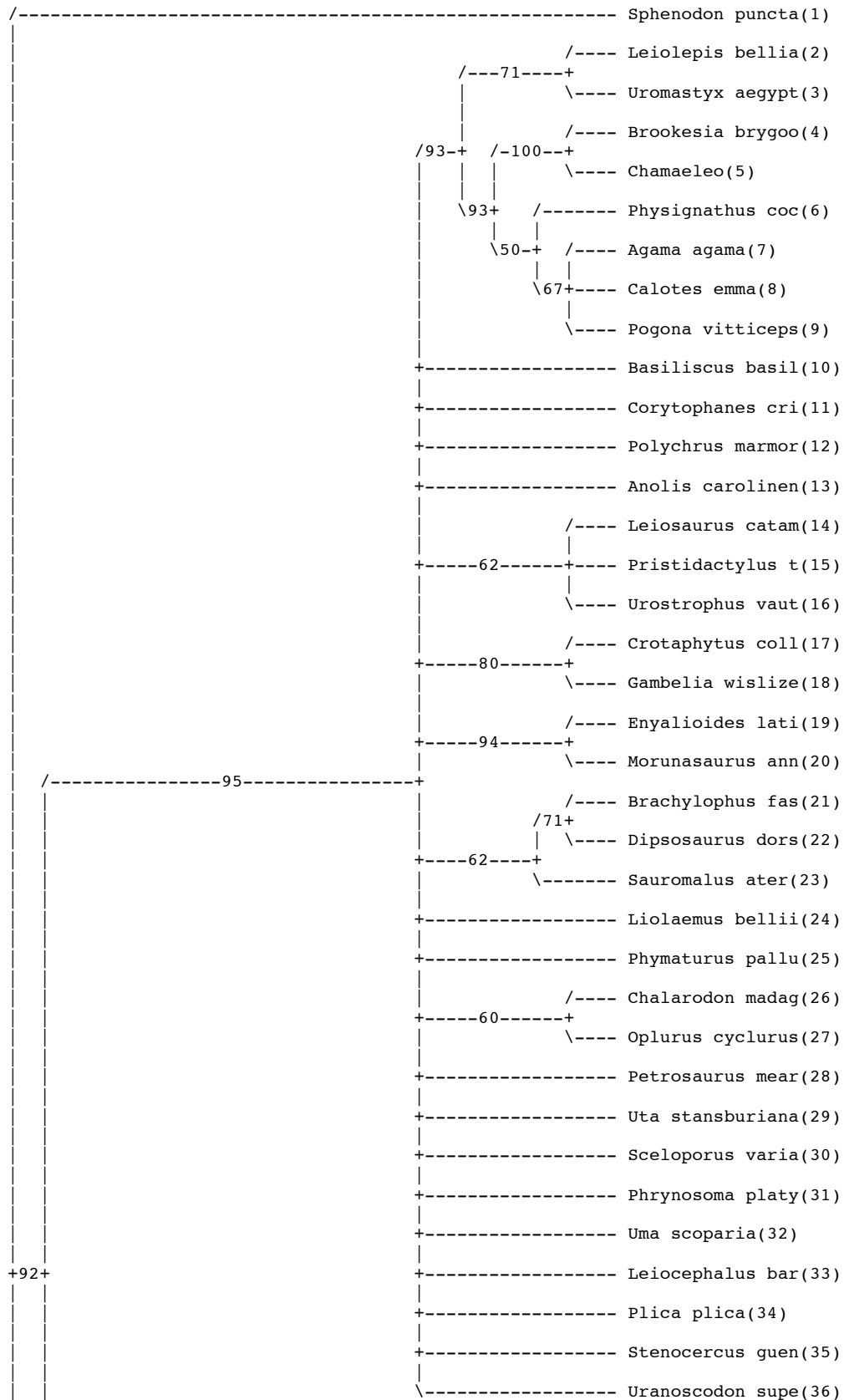

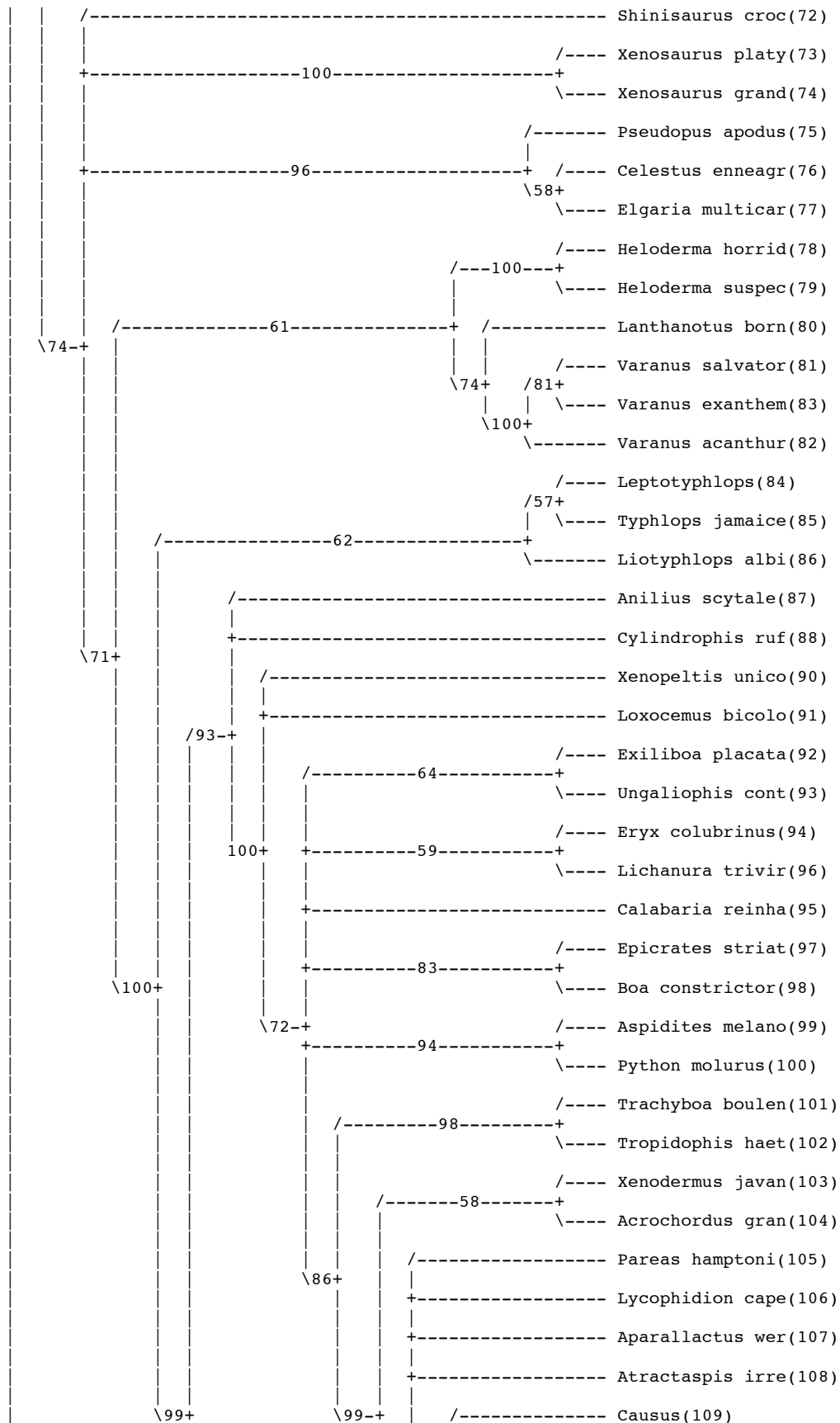

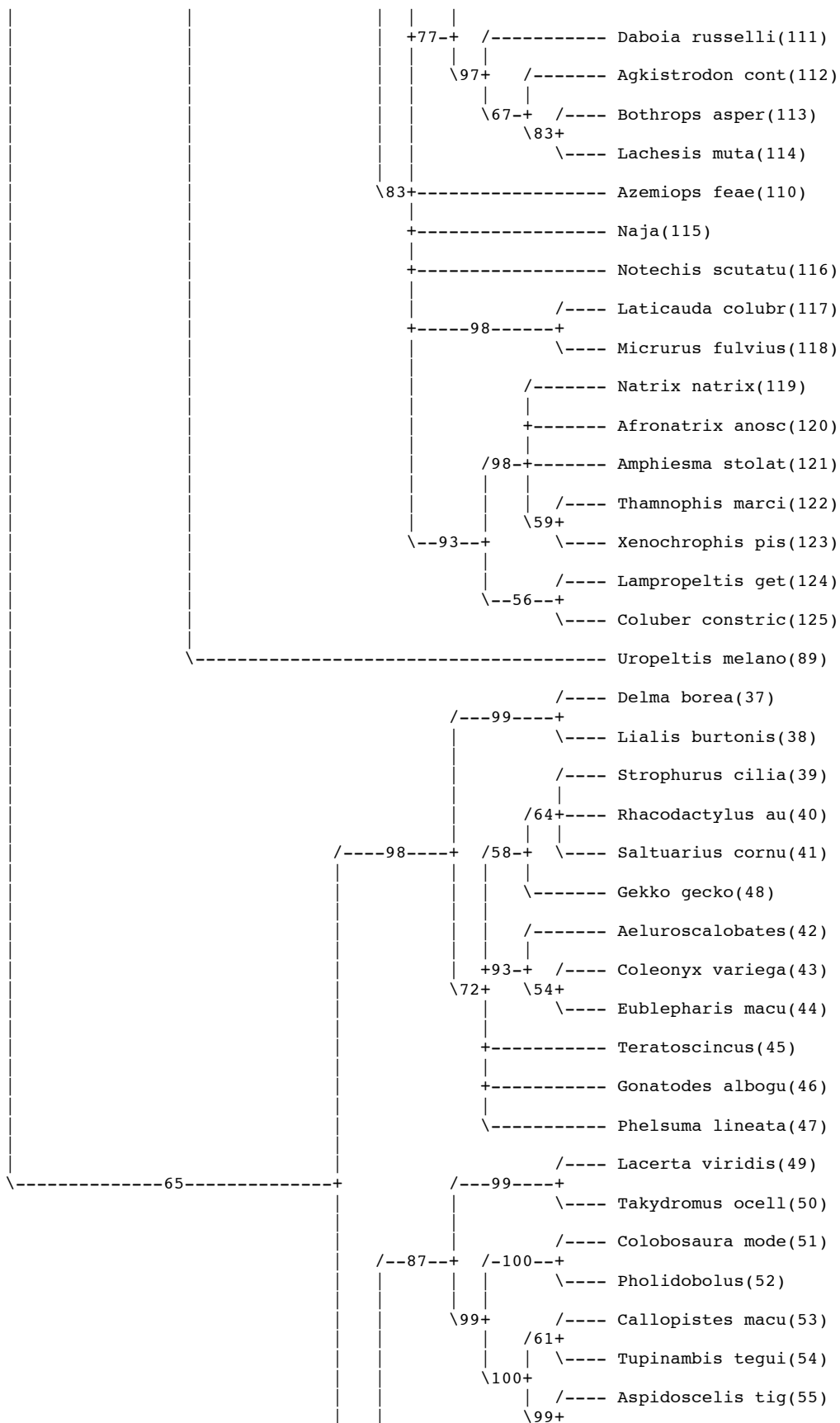

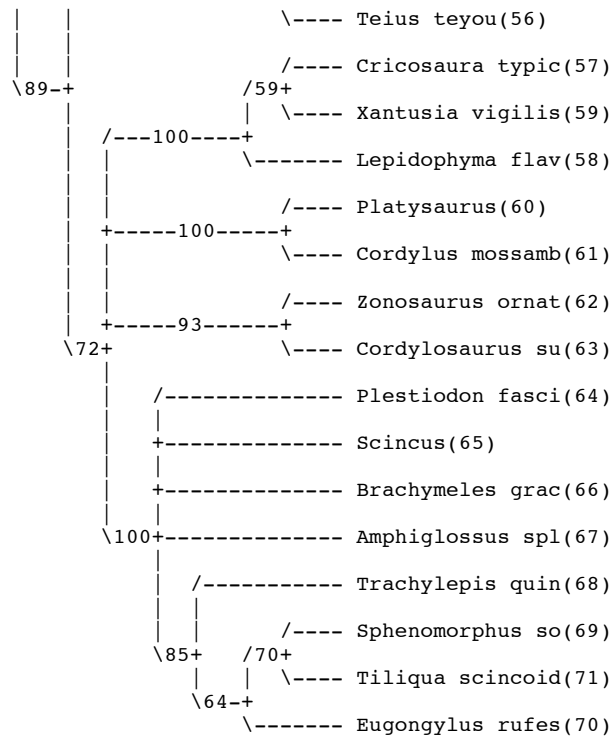

Supplement: S27 Fig — Bootstrap values less than 50% are not shown and the corresponding branch is collapsed. (PDF) [file pone.0118199.s029.pdf]

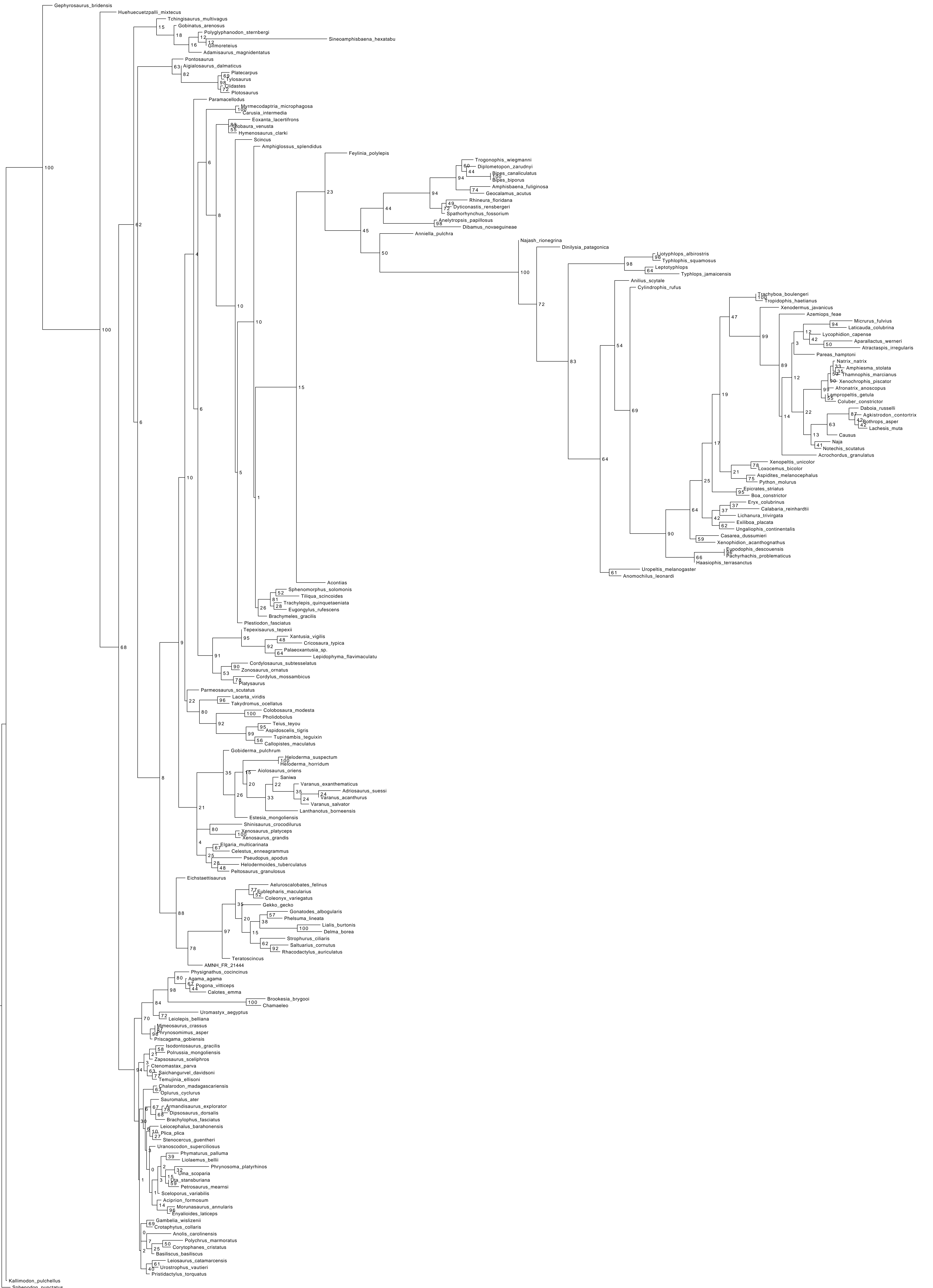

Supplement: S28 Fig — Numbers at nodes indicate bootstrap support values. (PDF) [file pone.0118199.s030.pdf]

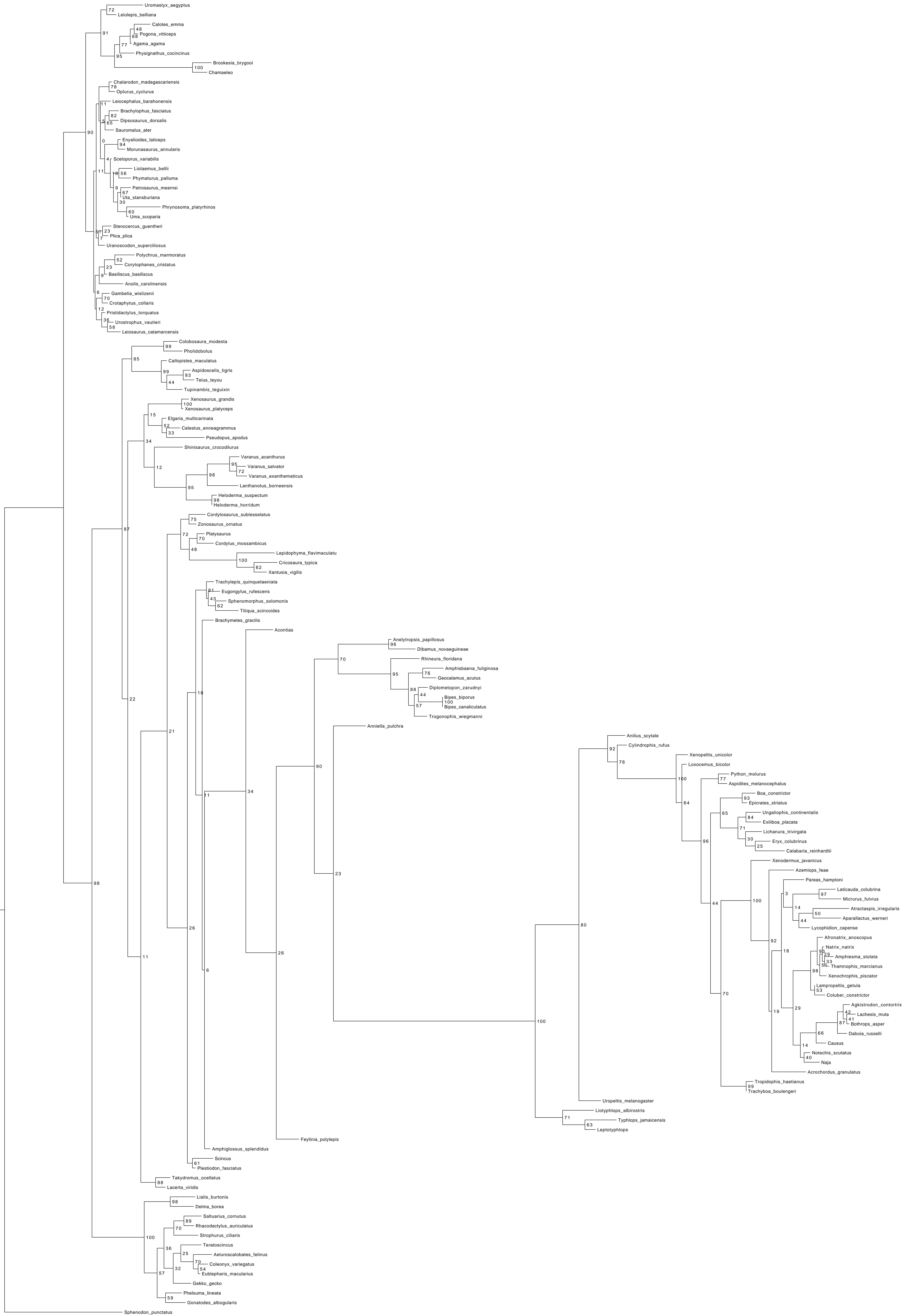

Supplement: S29 Fig — Numbers at nodes indicate bootstrap support values. (PDF) [file pone.0118199.s031.pdf]

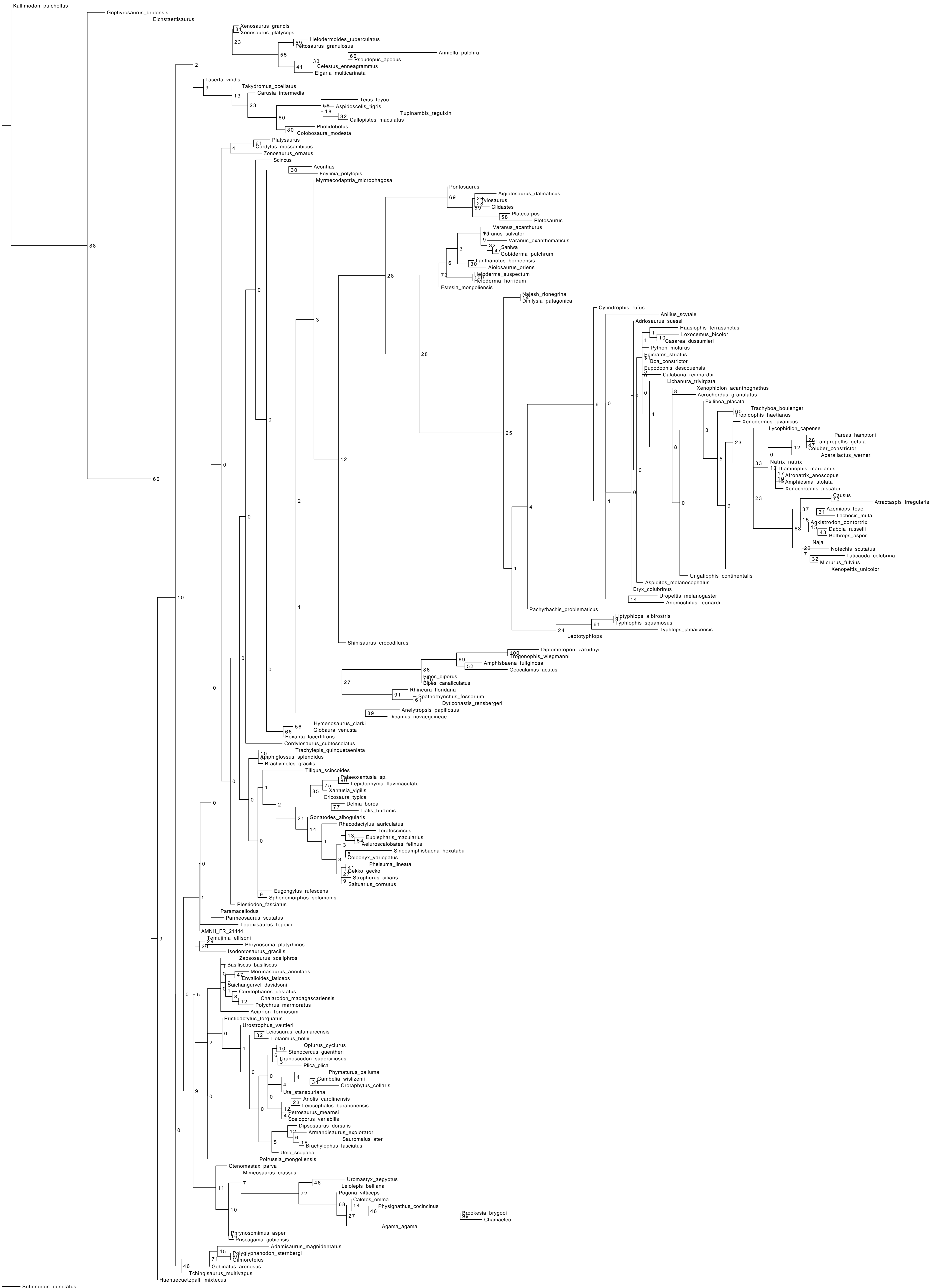

Supplement: S30 Fig — Numbers at nodes indicate bootstrap support values. (PDF) [file pone.0118199.s032.pdf]

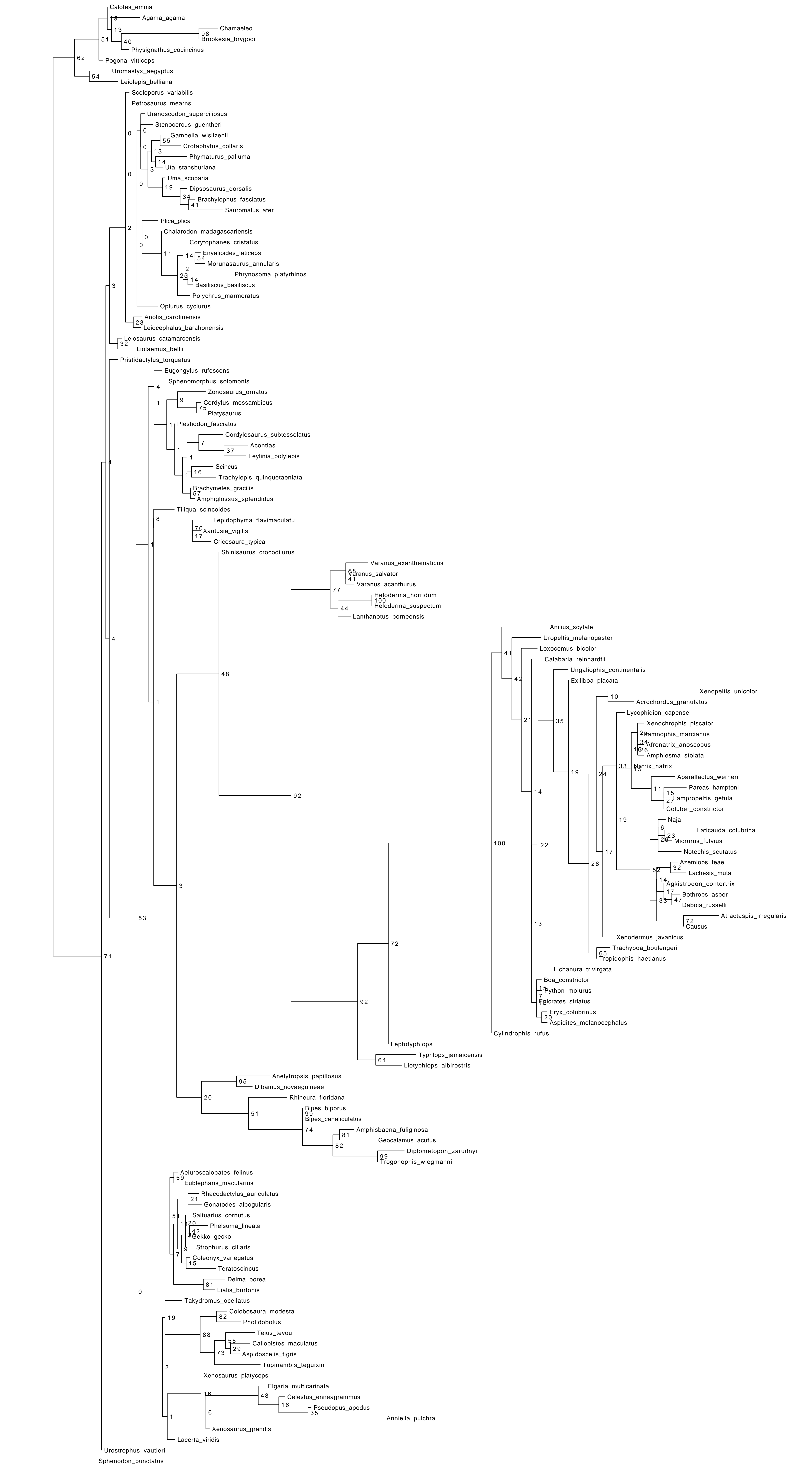

Supplement: S31 Fig — Numbers at nodes indicate bootstrap support values. (PDF) [file pone.0118199.s033.pdf]

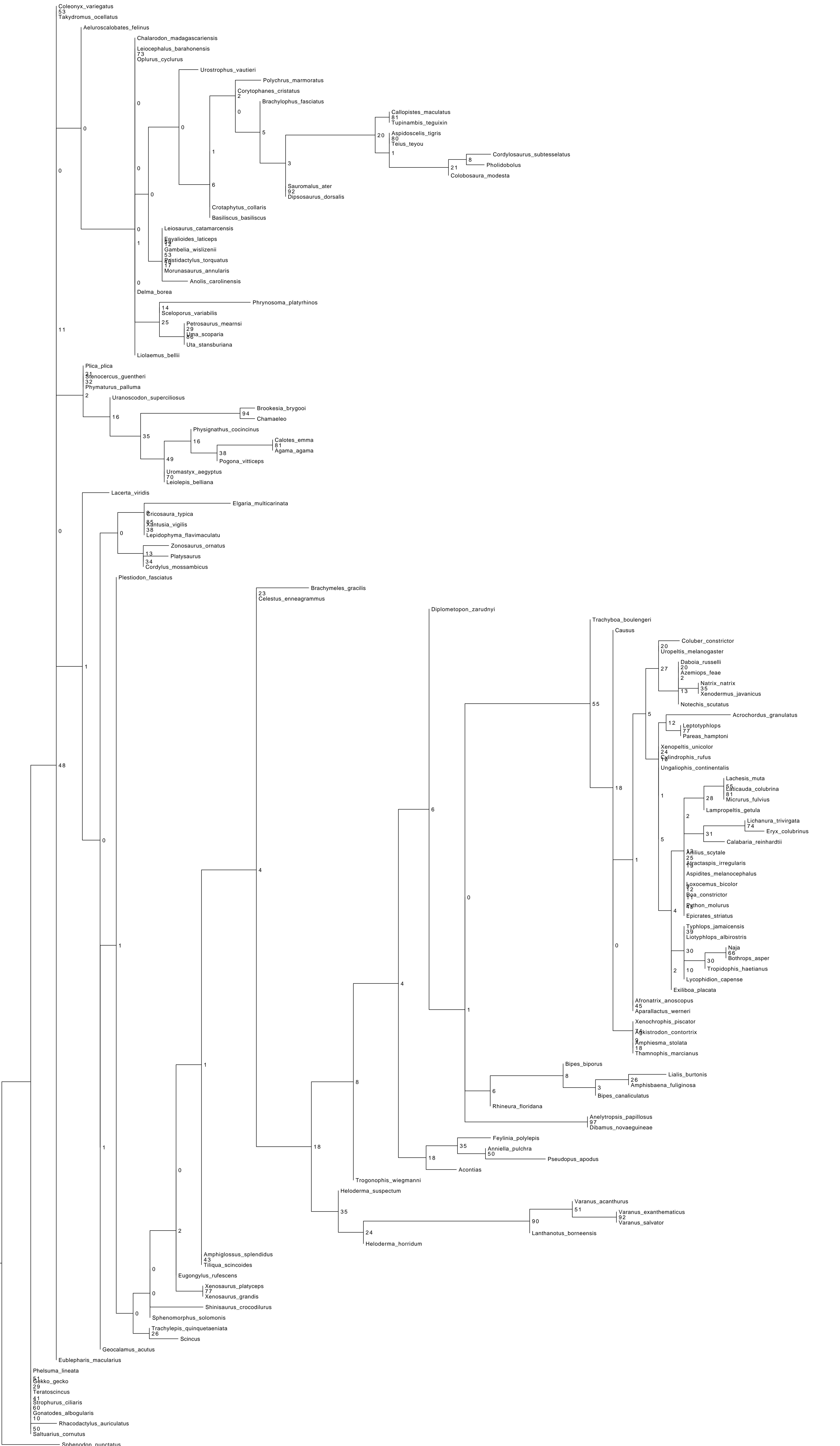

Supplement: S33 Fig — Numbers at nodes indicate bootstrap support values. (PDF) [file pone.0118199.s035.pdf]

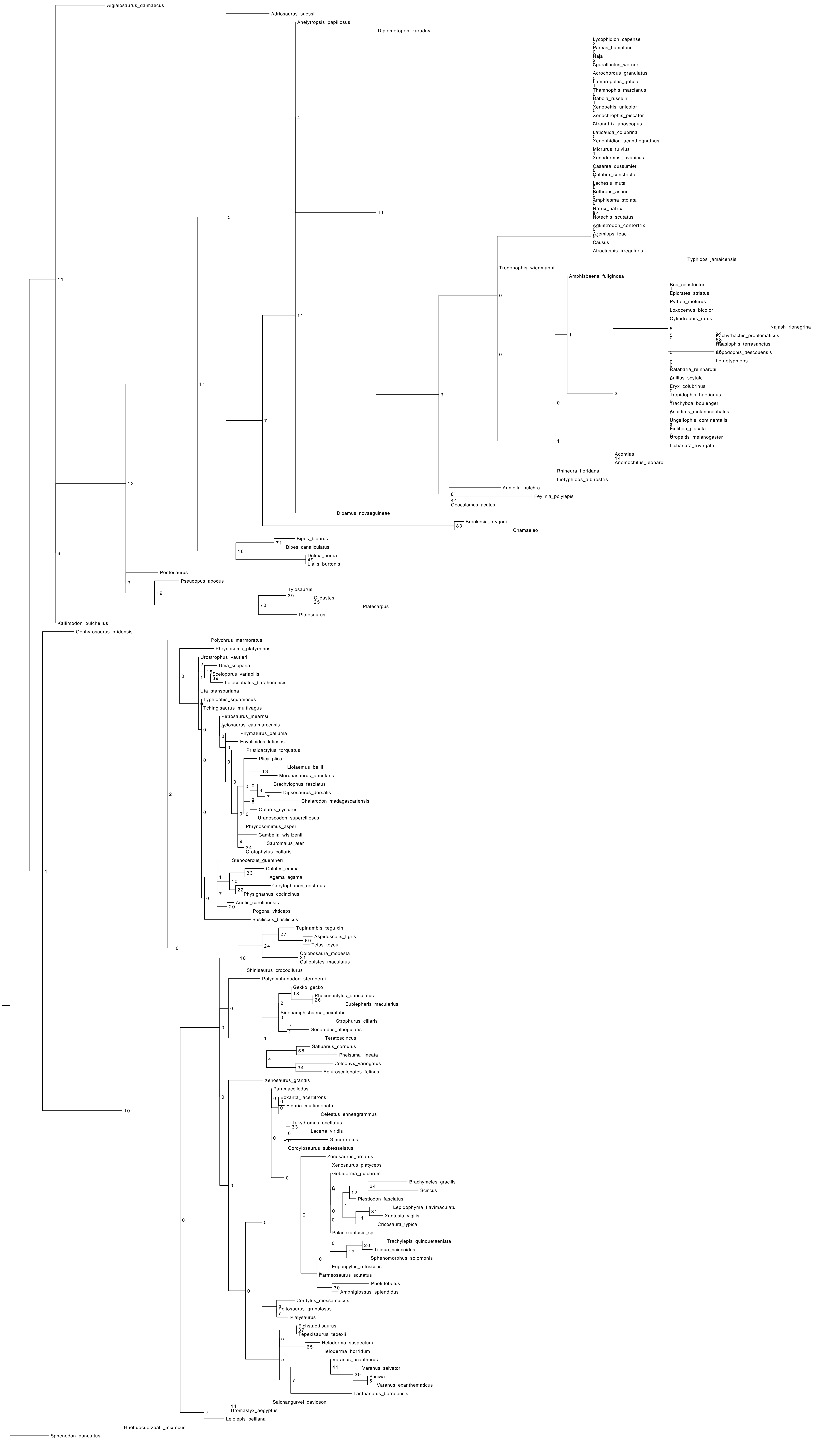

Supplement: S34 Fig — Numbers at nodes indicate bootstrap support values. (PDF) [file pone.0118199.s036.pdf]

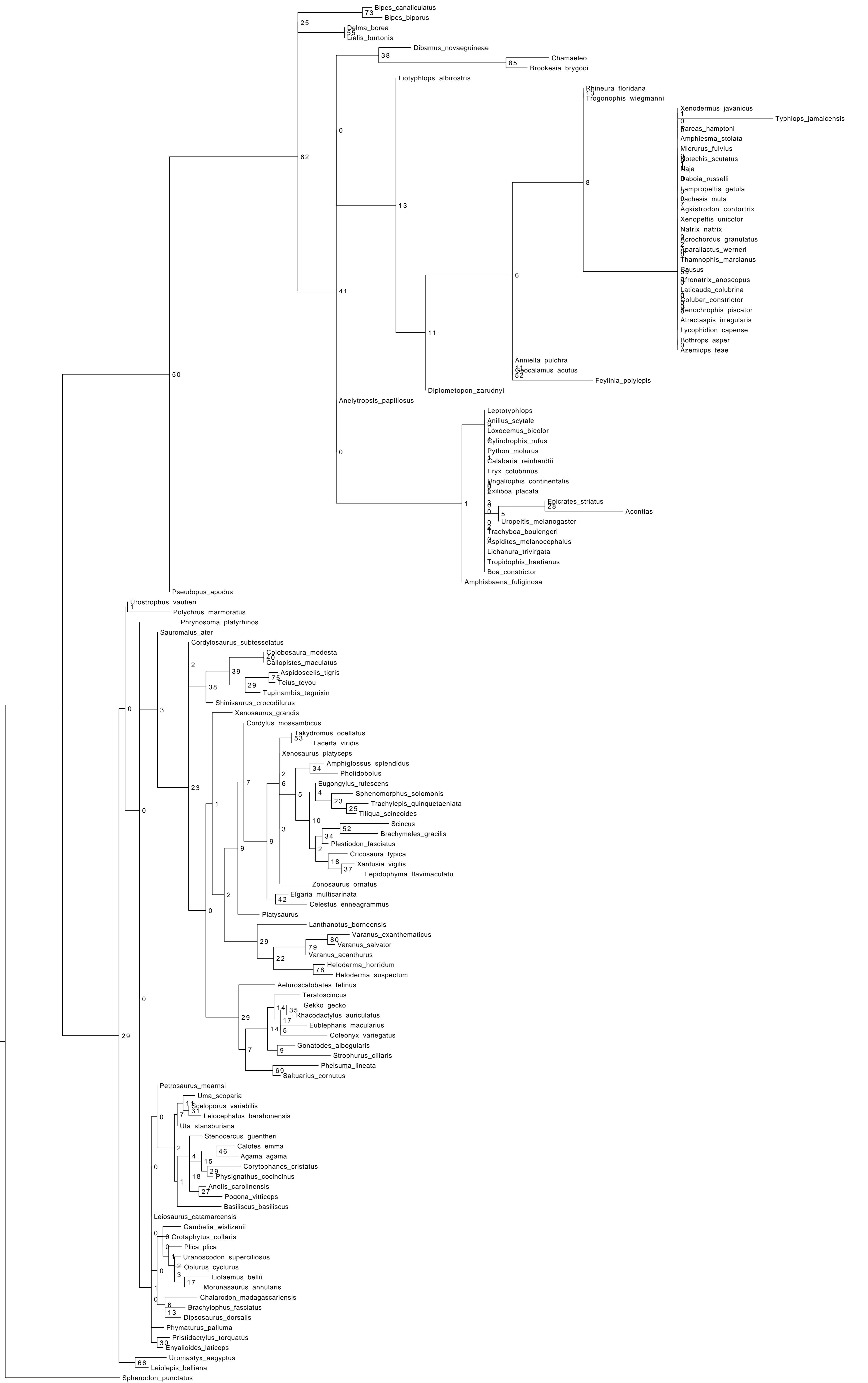

Supplement: S35 Fig — Numbers at nodes indicate bootstrap support values. (PDF) [file pone.0118199.s037.pdf]

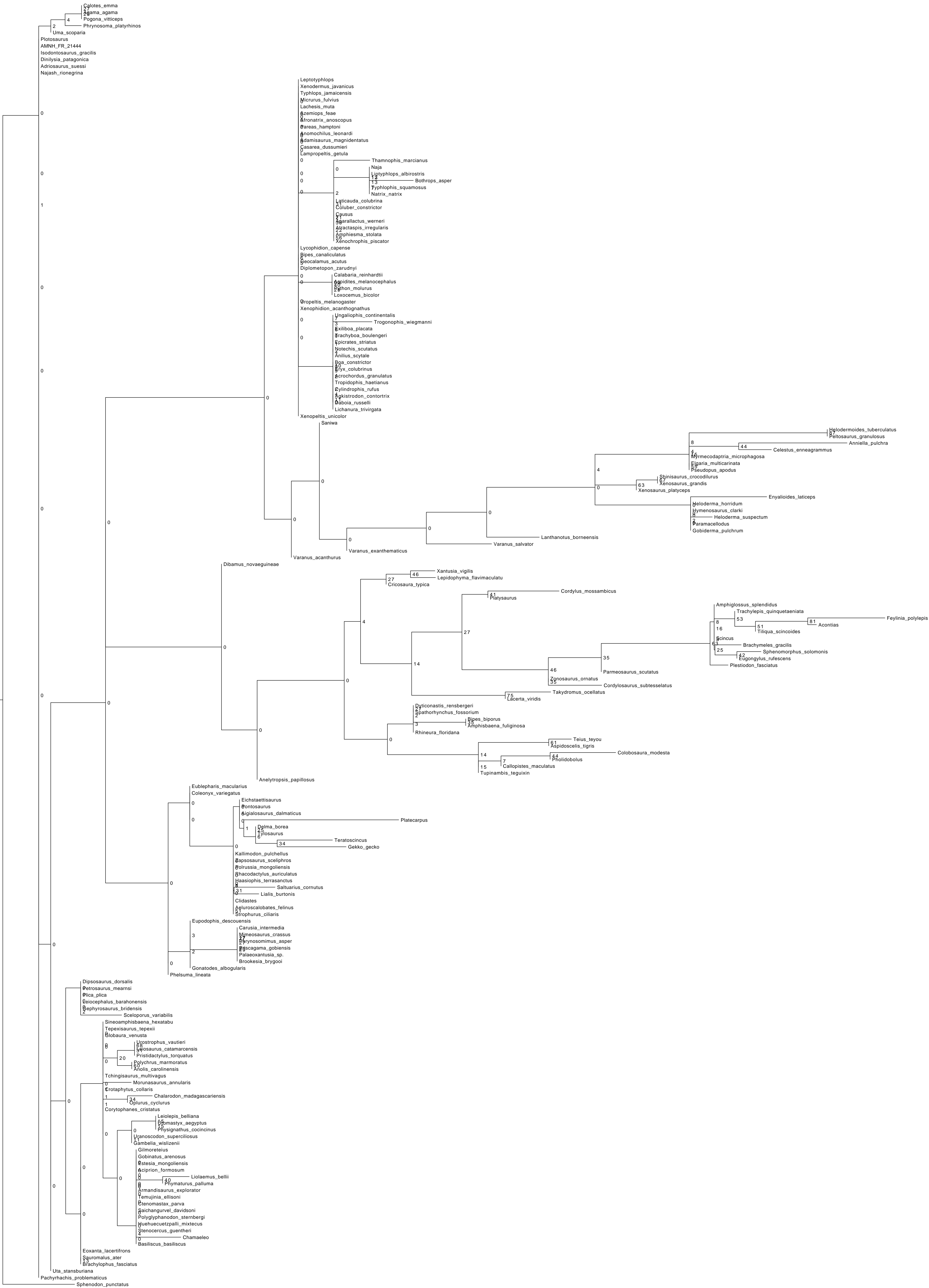

Supplement: S36 Fig — Numbers at nodes indicate bootstrap support values. (PDF) [file pone.0118199.s038.pdf]

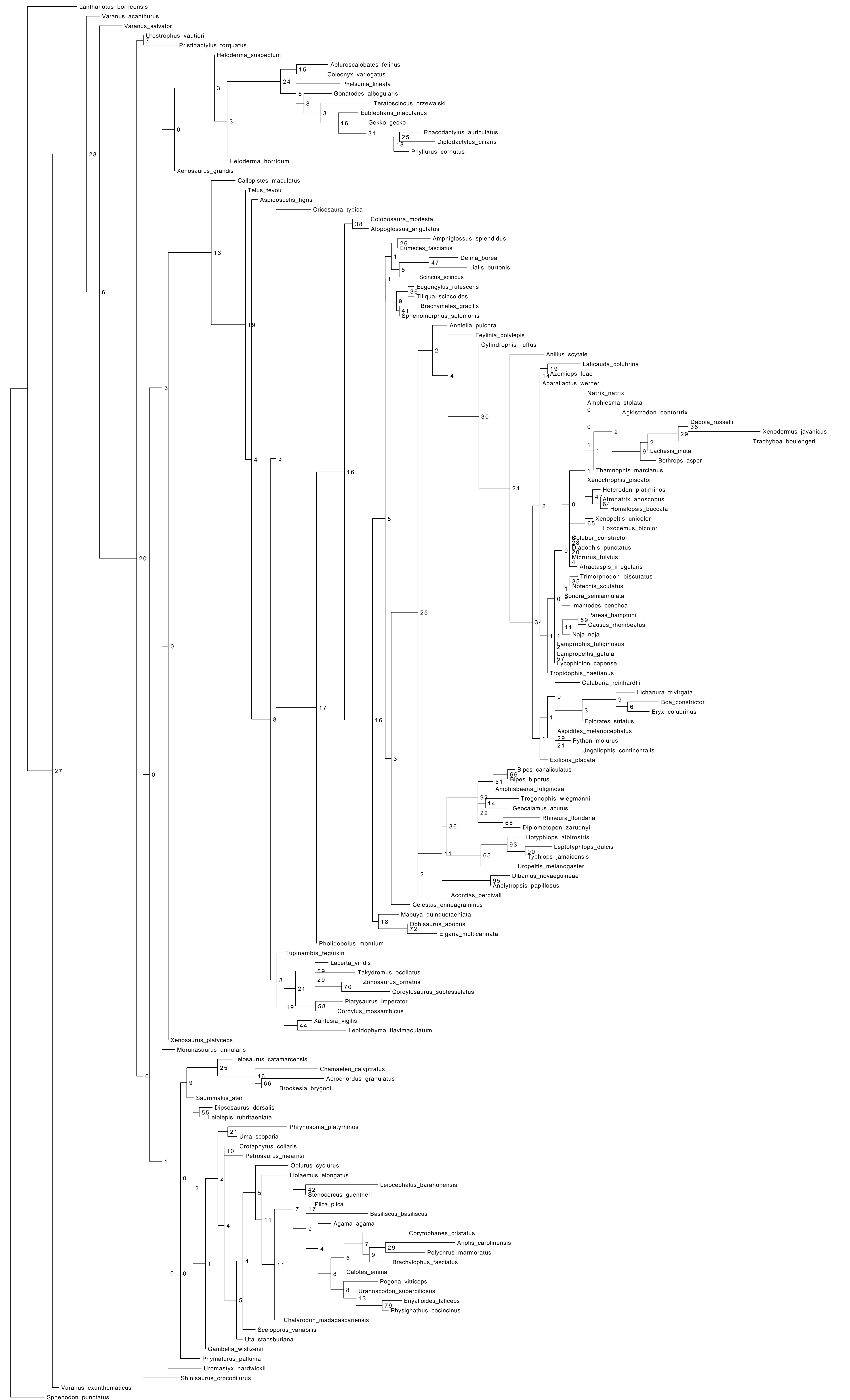

Supplement: S38 Fig — Numbers at nodes indicate bootstrap support values. (PDF) [file pone.0118199.s040.pdf]

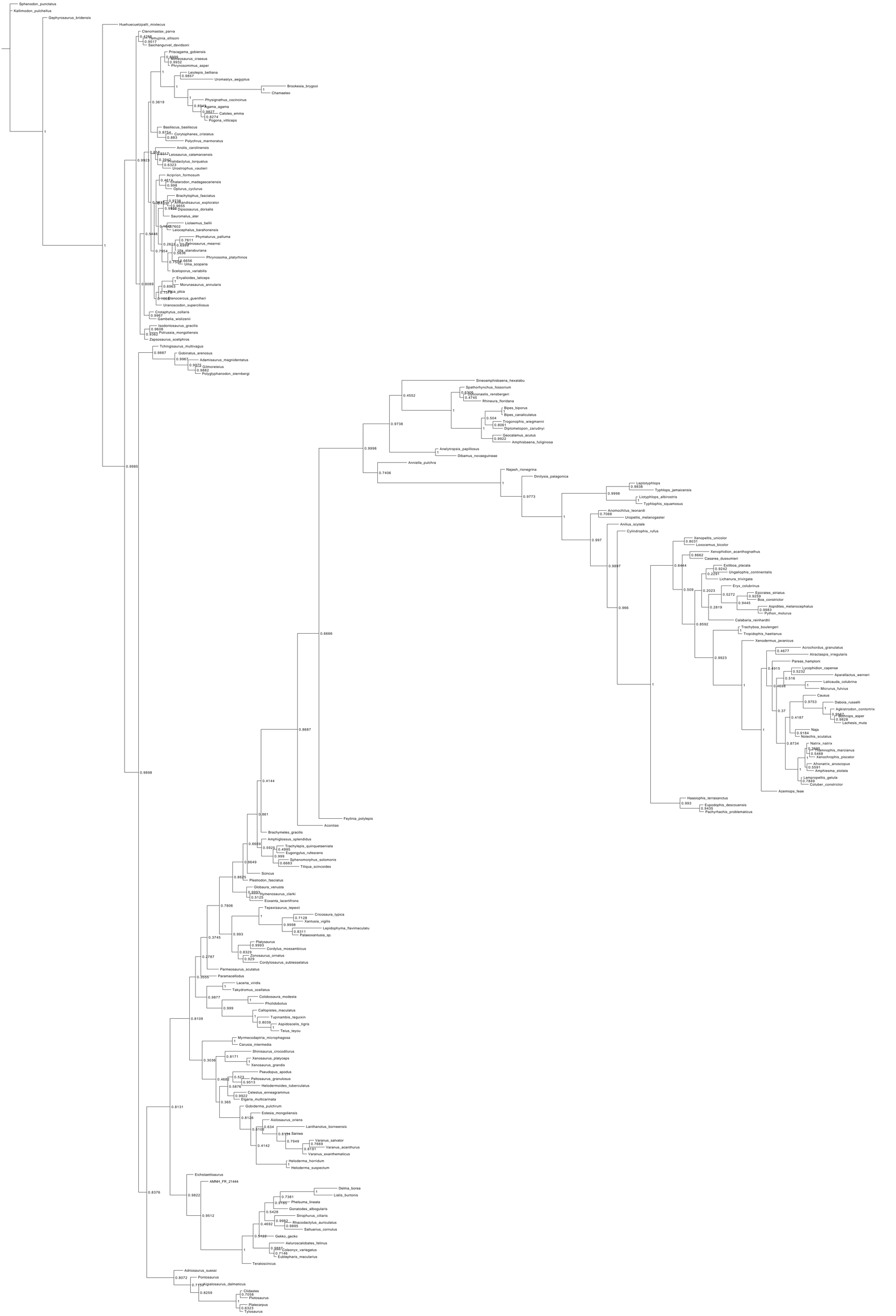

Supplement: S39 Fig — Numbers at nodes indicate posterior probabilities. (PDF) [file pone.0118199.s041.pdf]

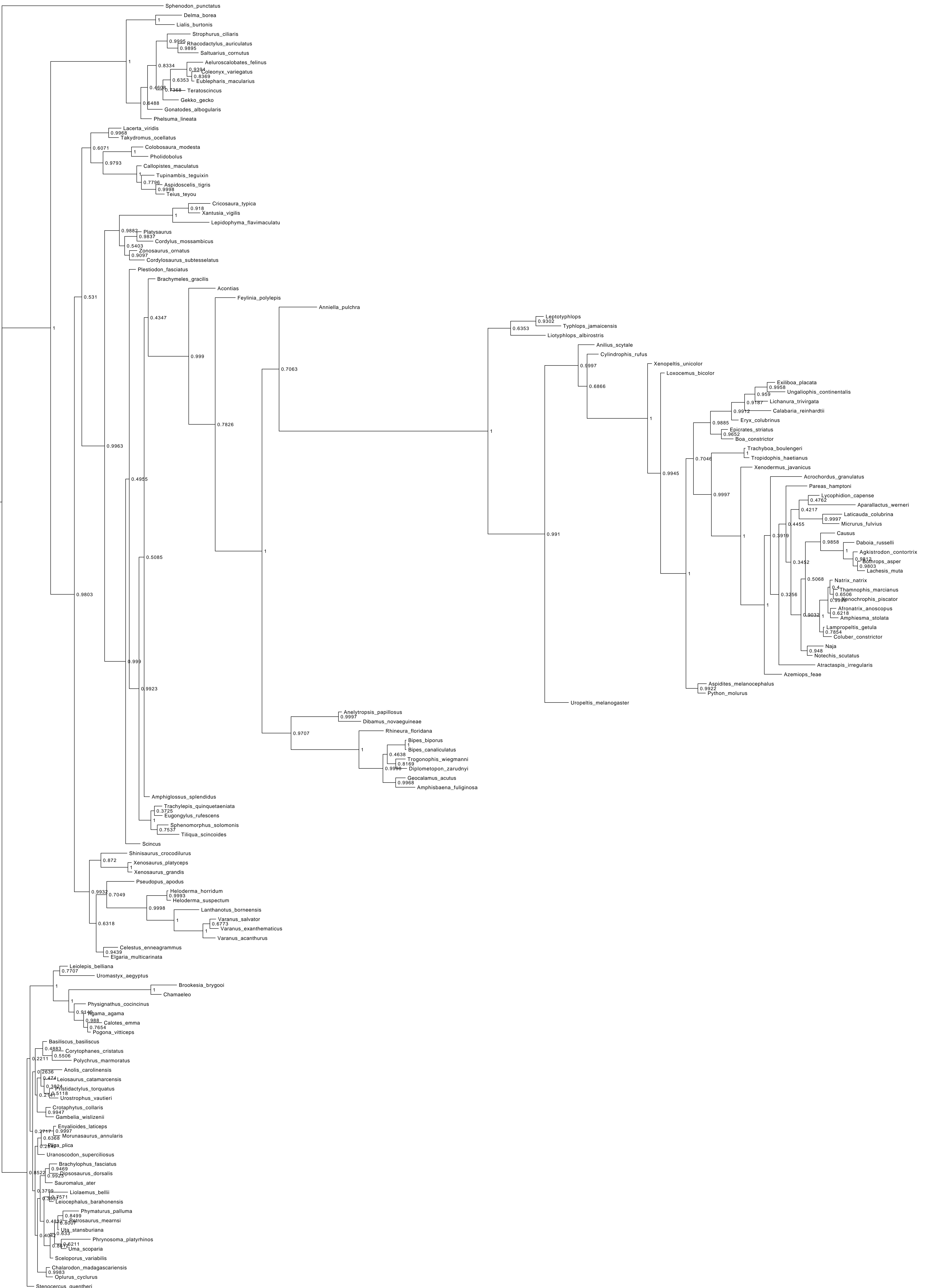

Supplement: S40 Fig — Numbers at nodes indicate posterior probabilities. (PDF) [file pone.0118199.s042.pdf]

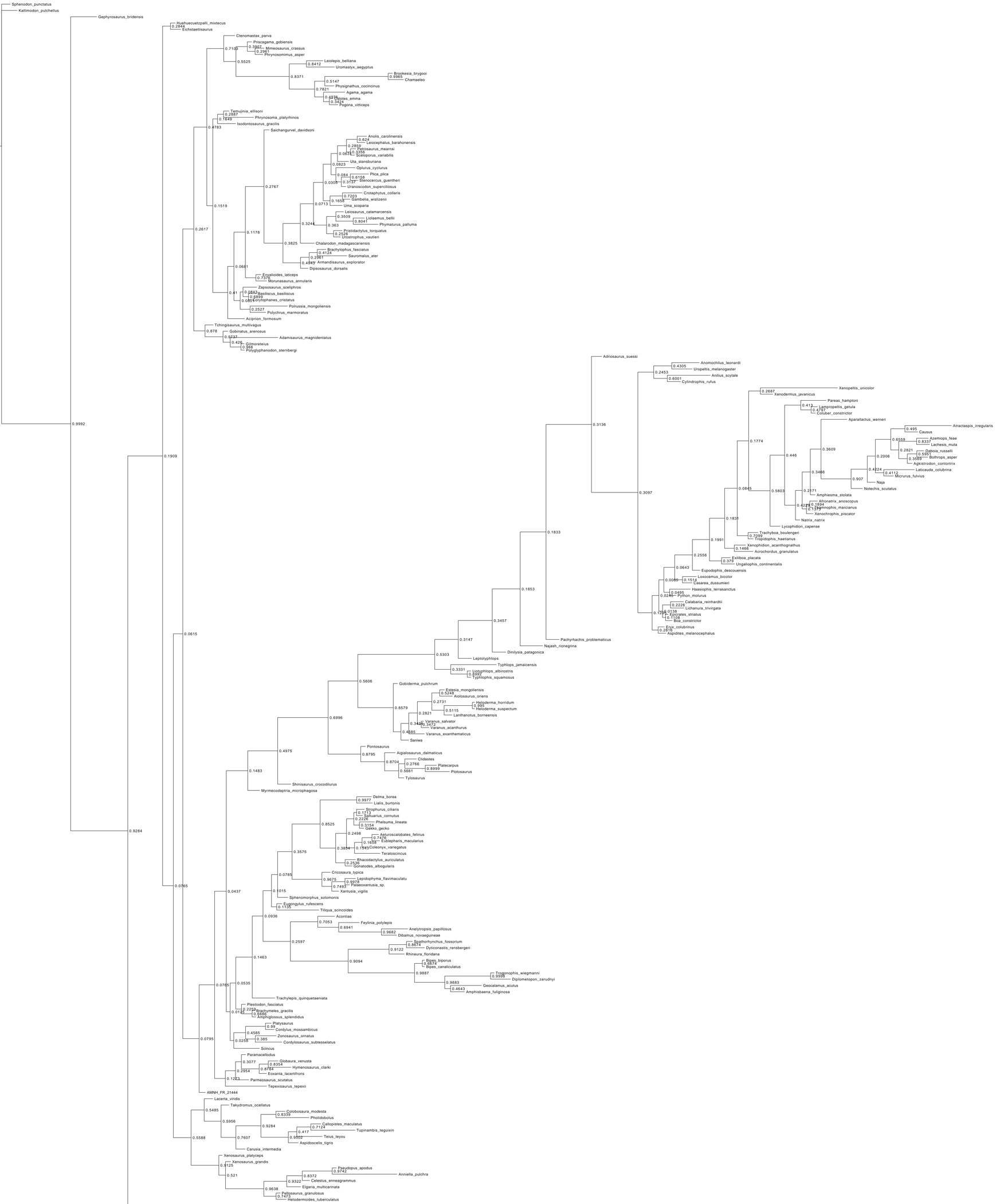

Supplement: S41 Fig — Numbers at nodes indicate posterior probabilities. (PDF) [file pone.0118199.s043.pdf]

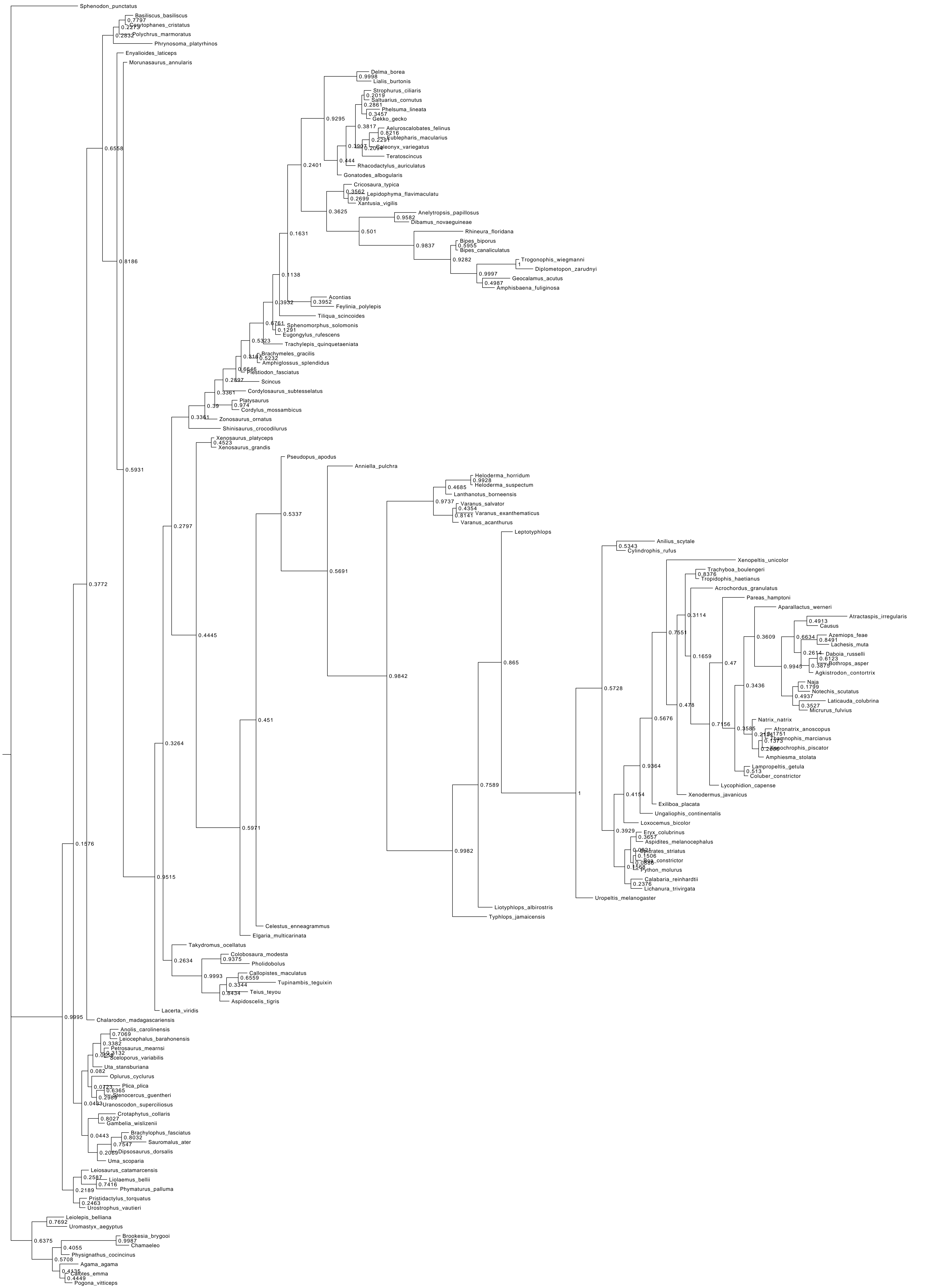

Supplement: S42 Fig — Numbers at nodes indicate posterior probabilities. (PDF) [file pone.0118199.s044.pdf]

– *Sphenodon punctatus*

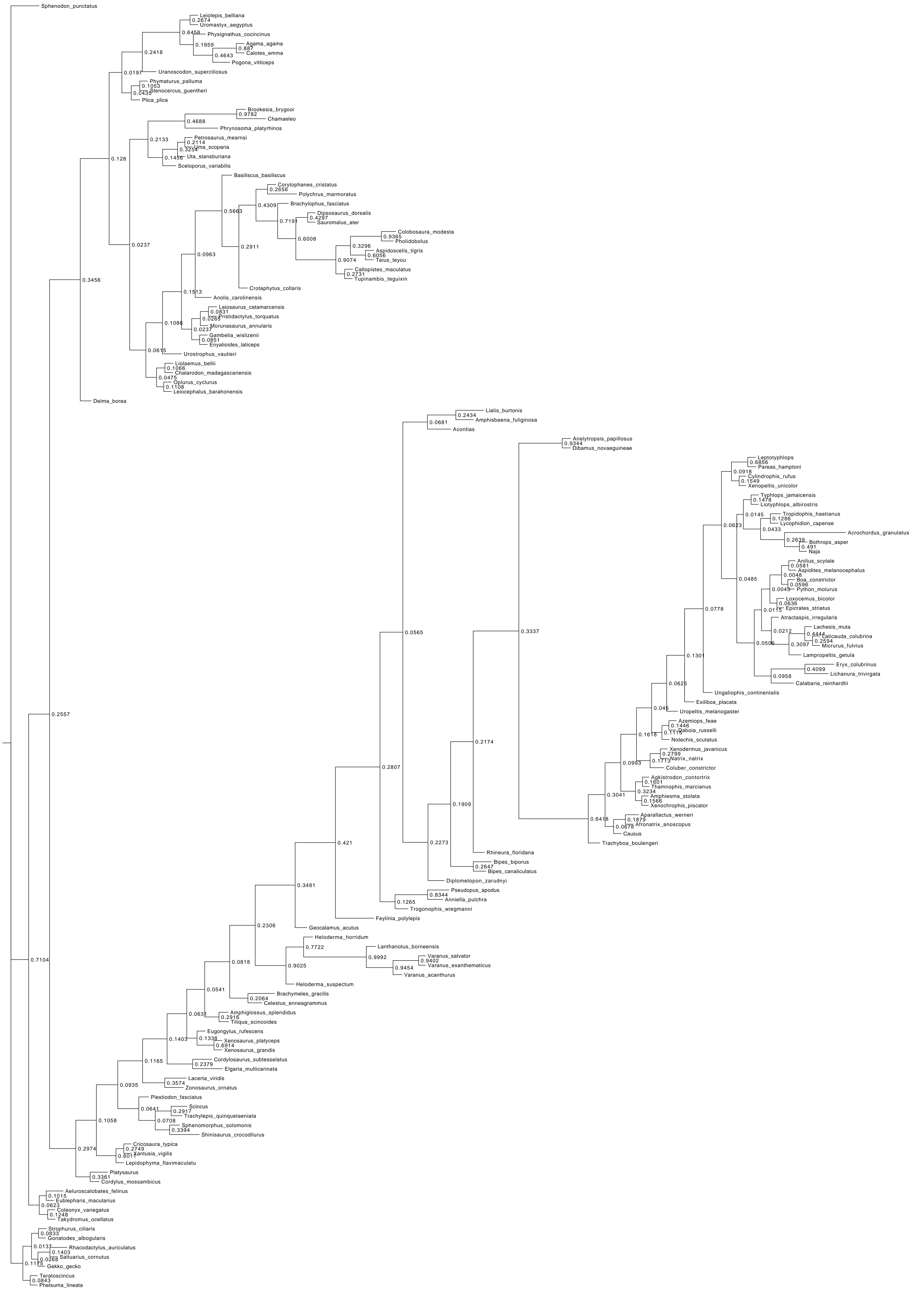

Supplement: S44 Fig — Numbers at nodes indicate posterior probabilities. (PDF) [file pone.0118199.s046.pdf]

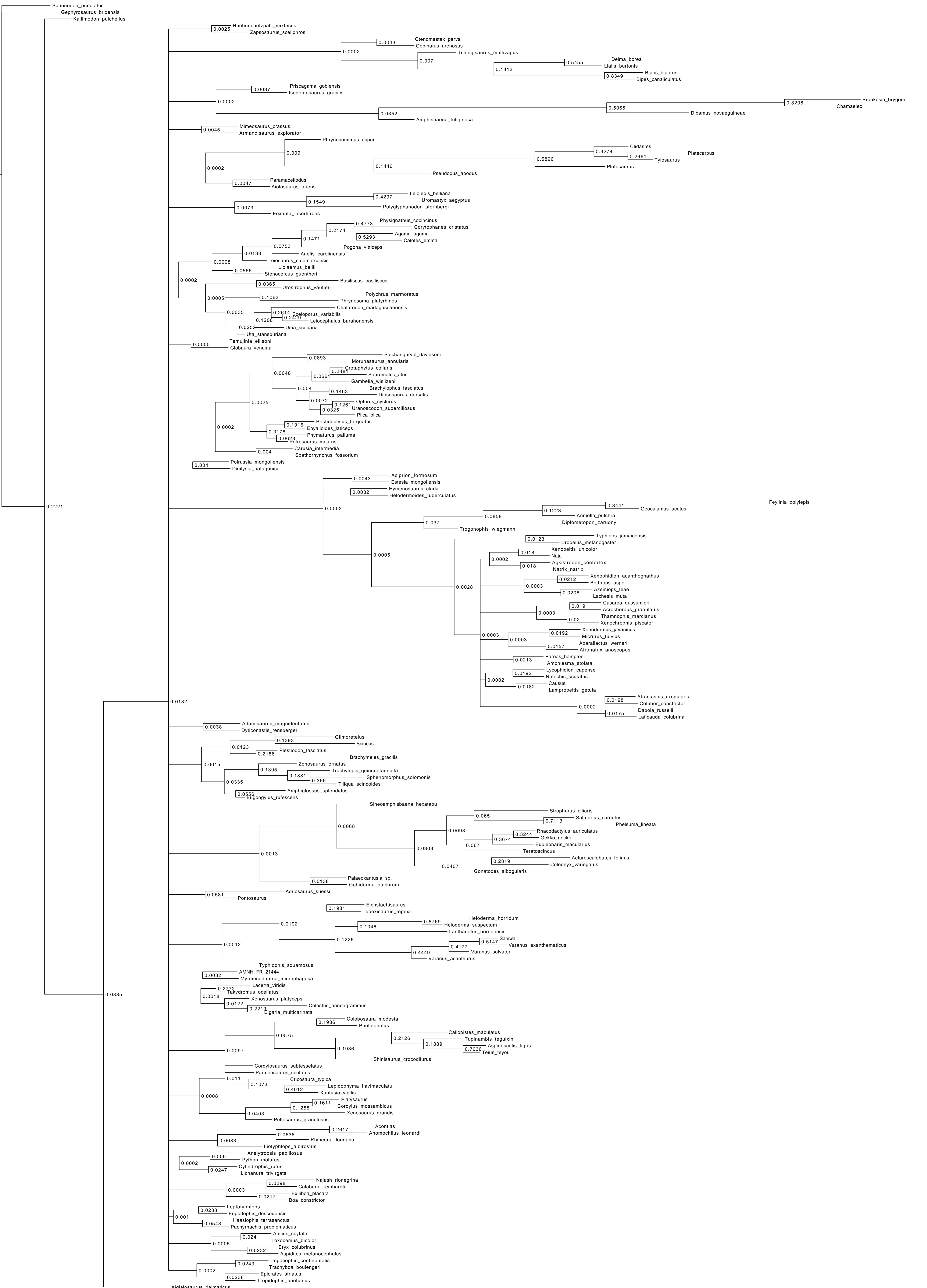

Supplement: S45 Fig — Numbers at nodes indicate posterior probabilities. (PDF) [file pone.0118199.s047.pdf]

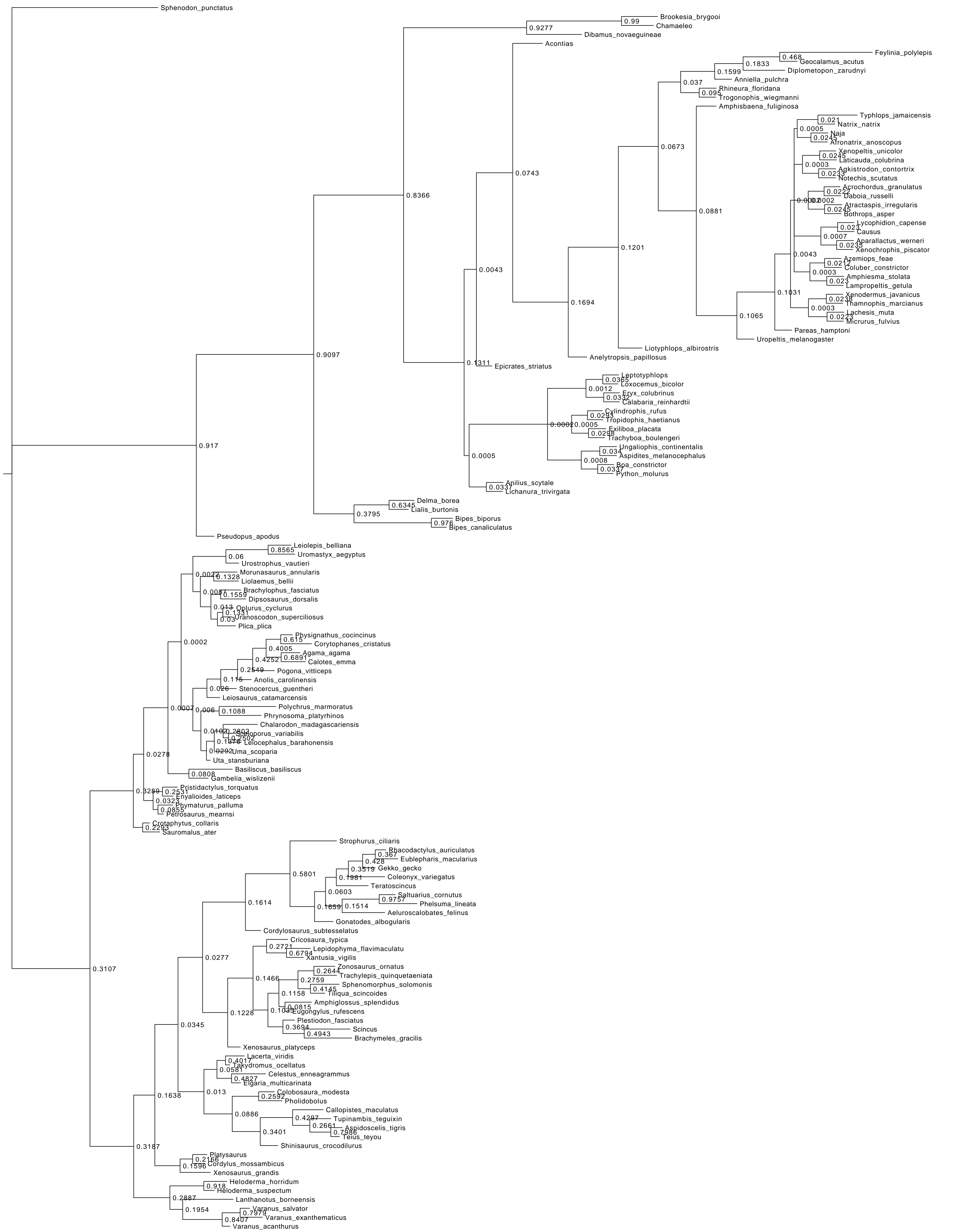

Supplement: S46 Fig — Numbers at nodes indicate posterior probabilities. (PDF) [file pone.0118199.s048.pdf]

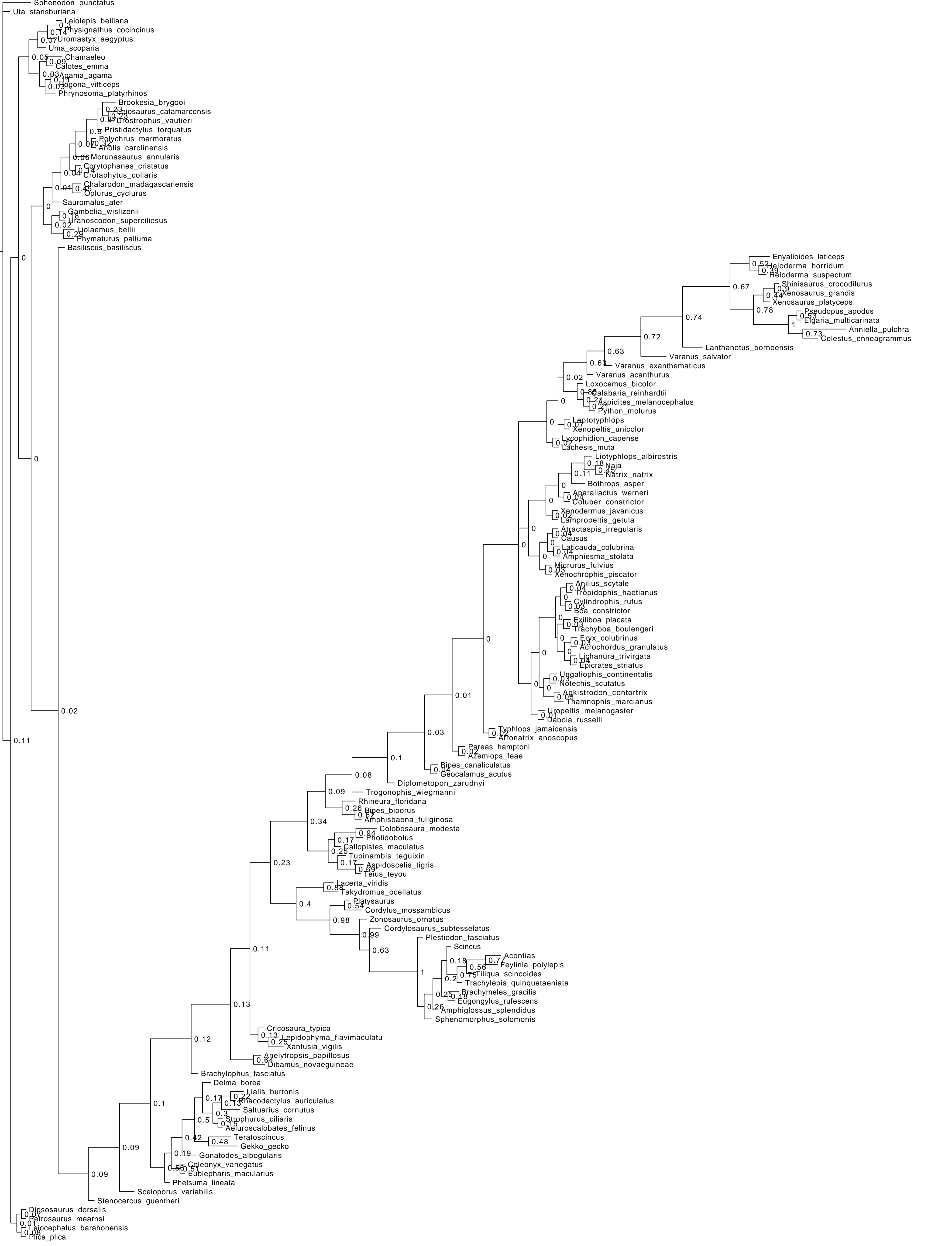

Supplement: S48 Fig — Numbers at nodes indicate posterior probabilities. (PDF) [file pone.0118199.s050.pdf]

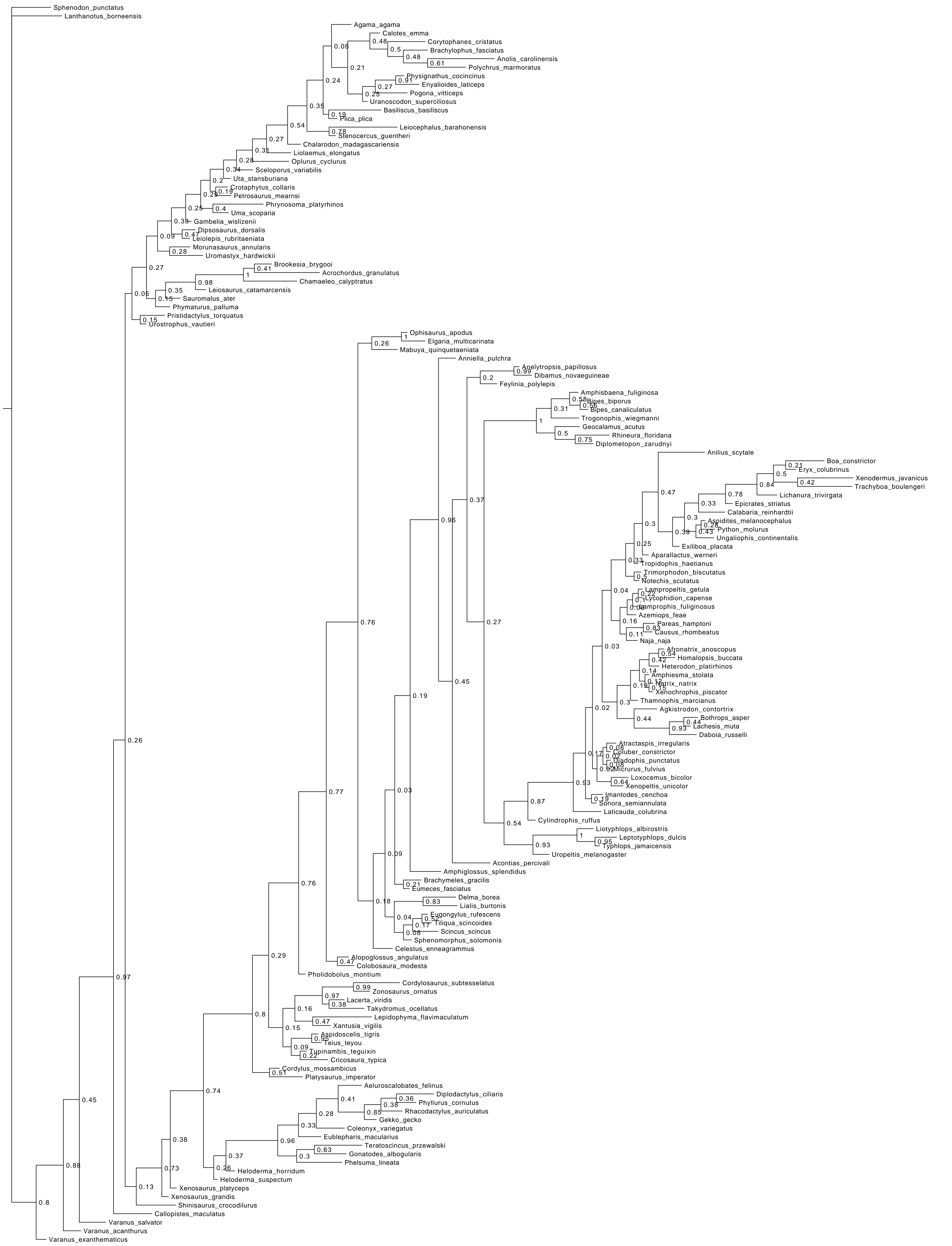

Supplement: S49 Fig — Numbers at nodes indicate posterior probabilities. (PDF) [file pone.0118199.s051.pdf]

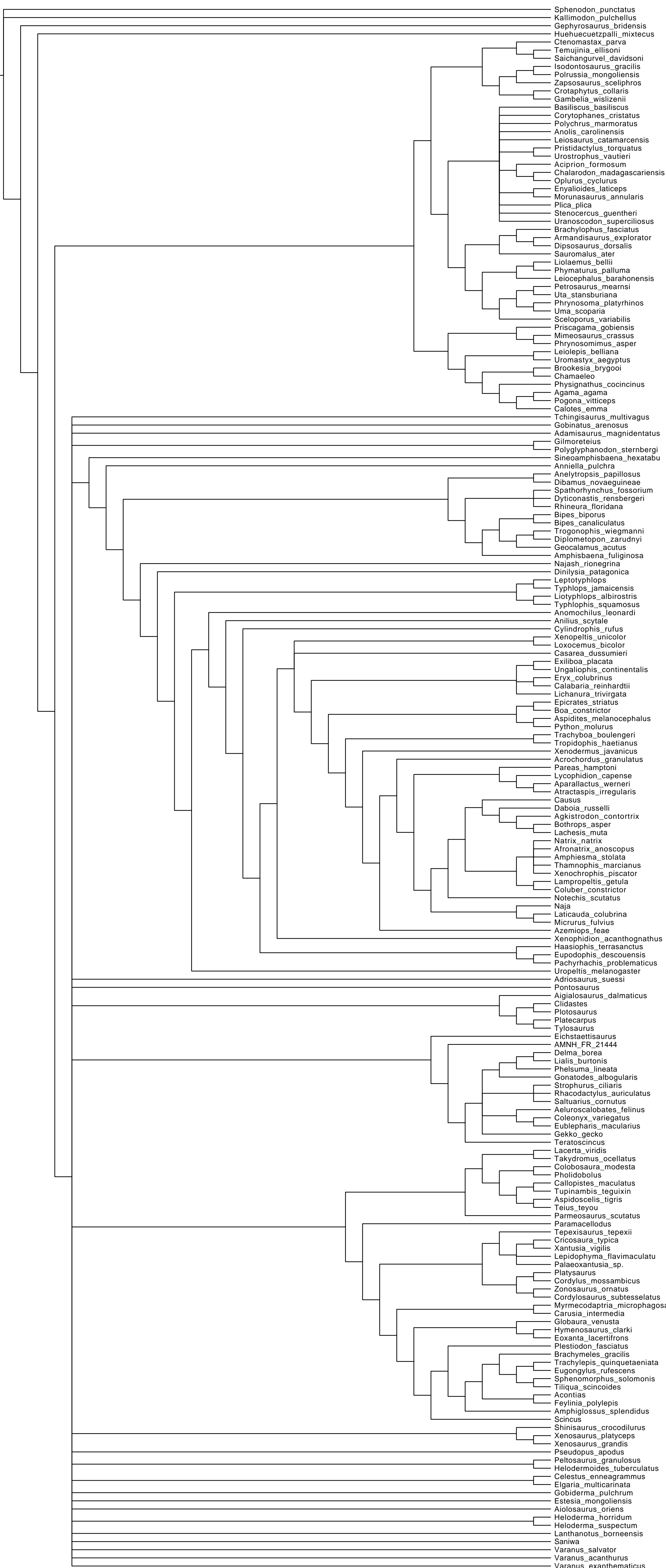

Supplement: S50 Fig — Strict consensus of 10,000 shortest trees (maximum number of trees retained) of length 2923. See S61 Fig. for bootstrap values. (PDF) [file pone.0118199.s052.pdf]

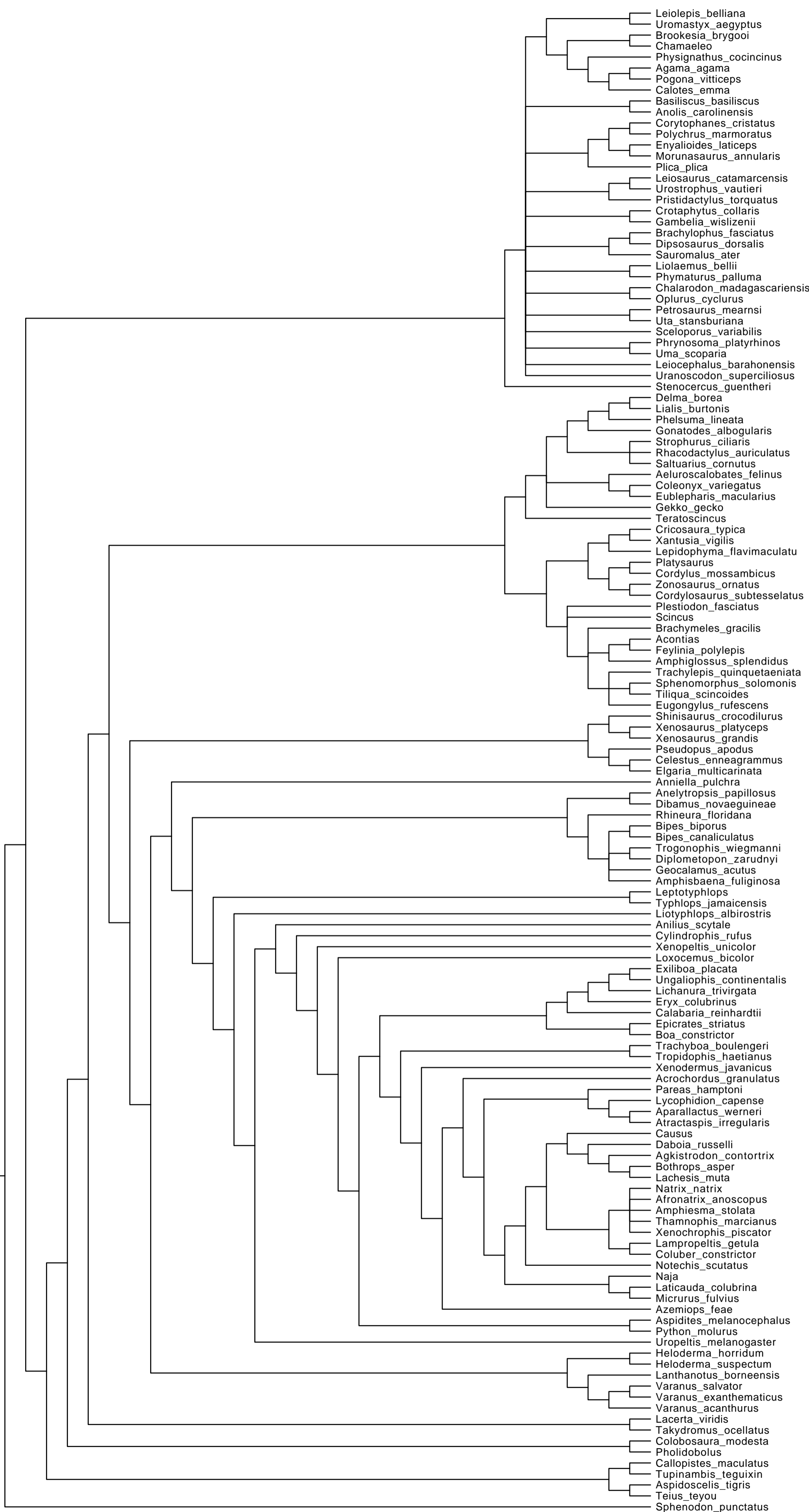

4.0

Supplement: S51 Fig — Strict consensus of 10,000 shortest trees (maximum number of trees retained) of length 2447. See S62 Fig. for bootstrap values. (PDF) [file pone.0118199.s053.pdf]

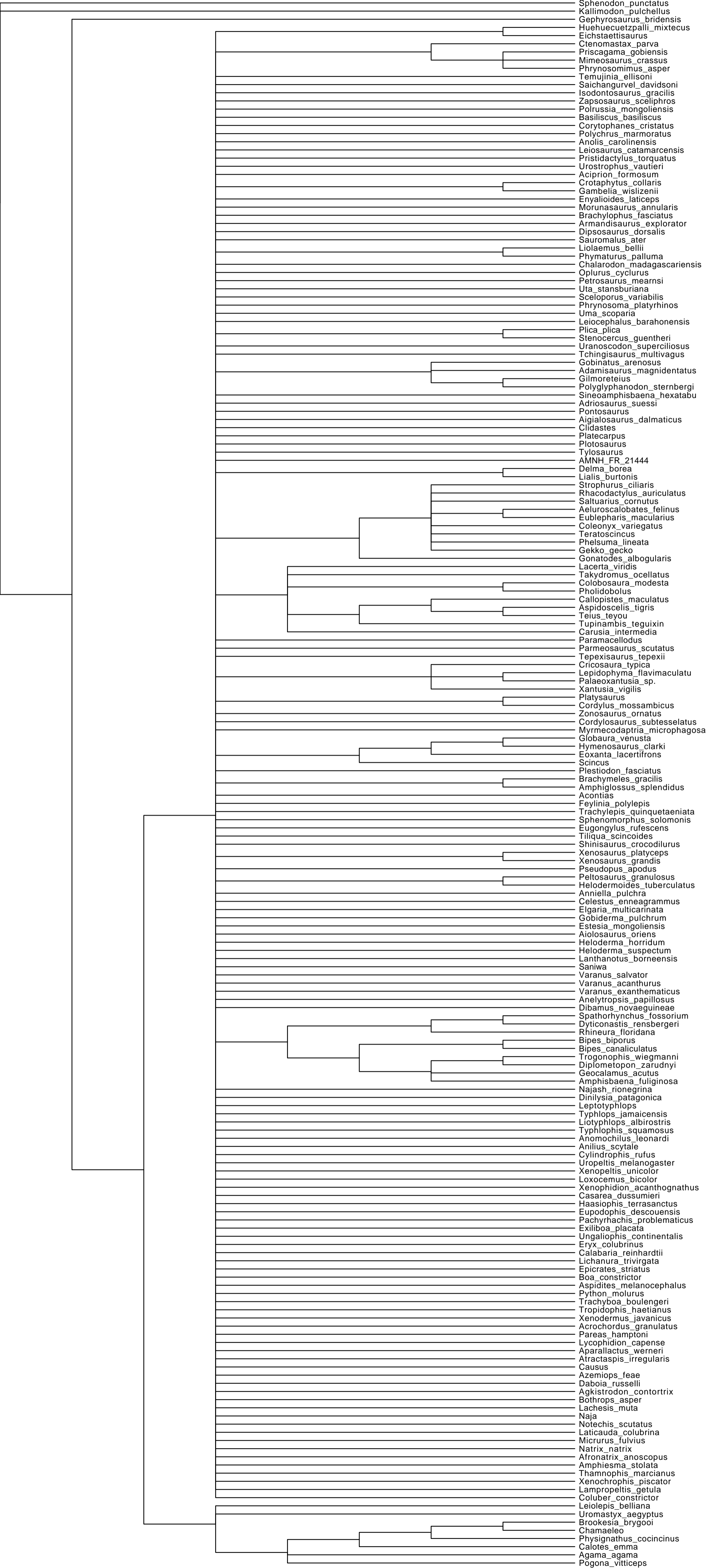

0.9

Supplement: S52 Fig — Strict consensus of 10,000 shortest trees (maximum number of trees retained) of length 993. See S63 Fig. for bootstrap values. (PDF) [file pone.0118199.s054.pdf]

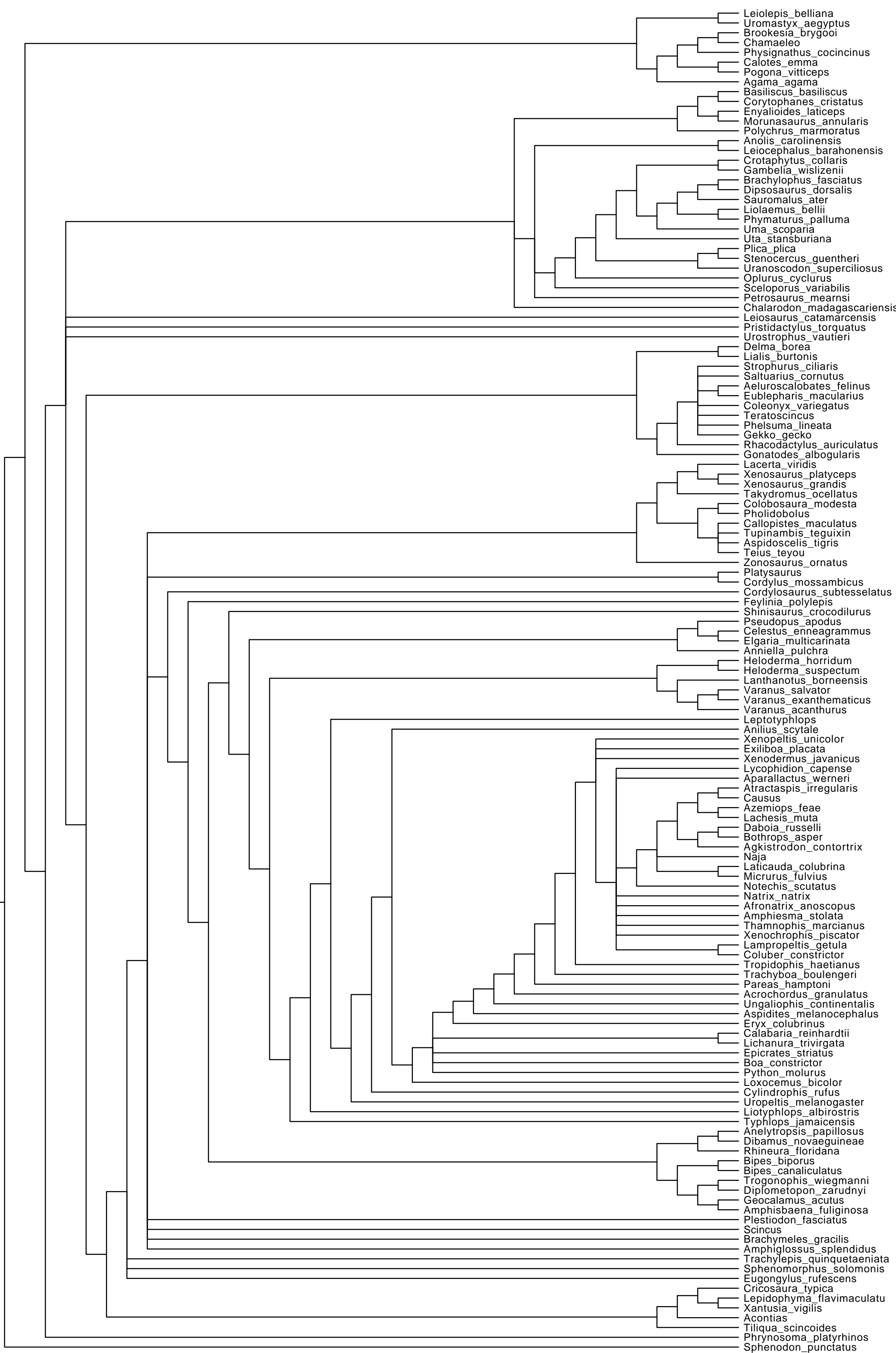

Supplement: S53 Fig — Strict consensus of 10,000 shortest trees (maximum number of trees) of length 810. See S64 Fig. for bootstrap values. (PDF) [file pone.0118199.s055.pdf]

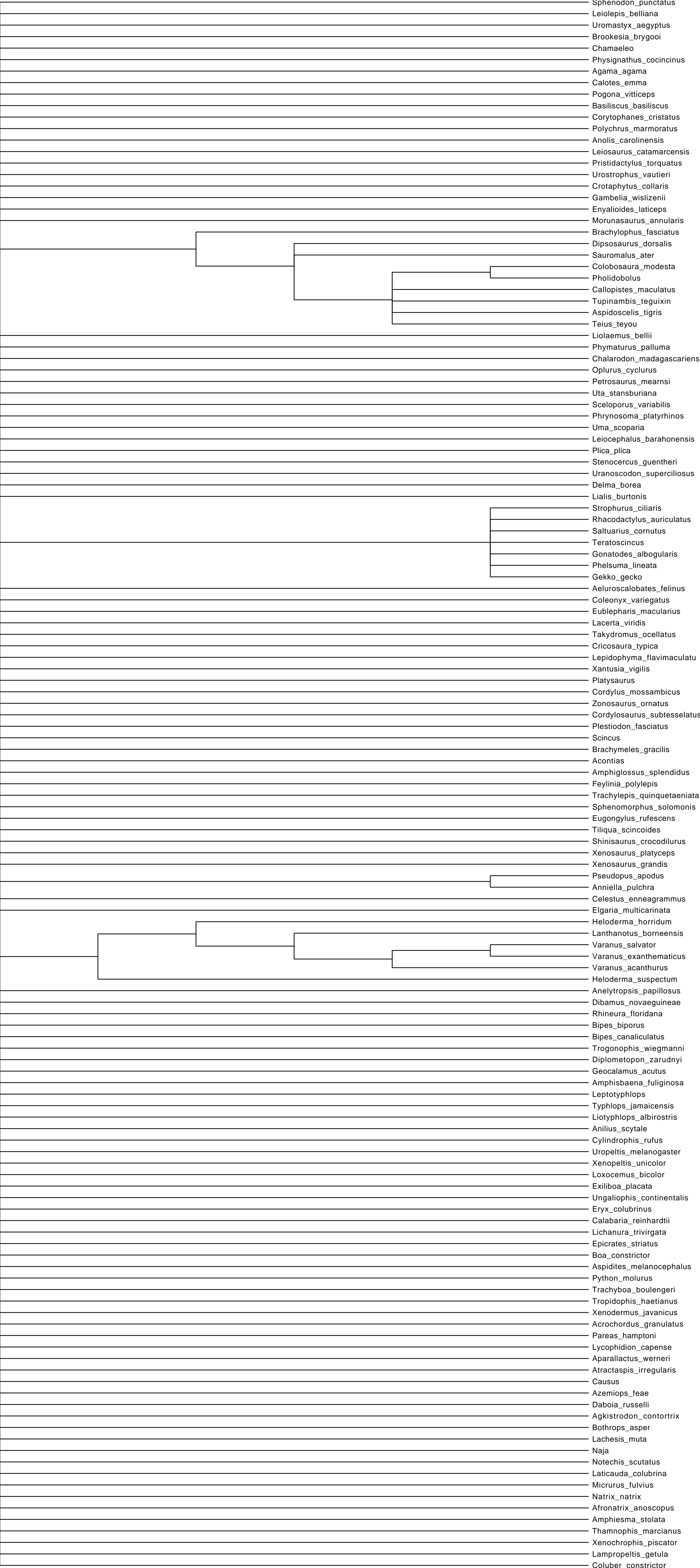

Supplement: S55 Fig — Strict consensus of 10,000 shortest trees (maximum number of trees retained) of length 145. See S66 Fig. for bootstrap values. (PDF) [file pone.0118199.s057.pdf]

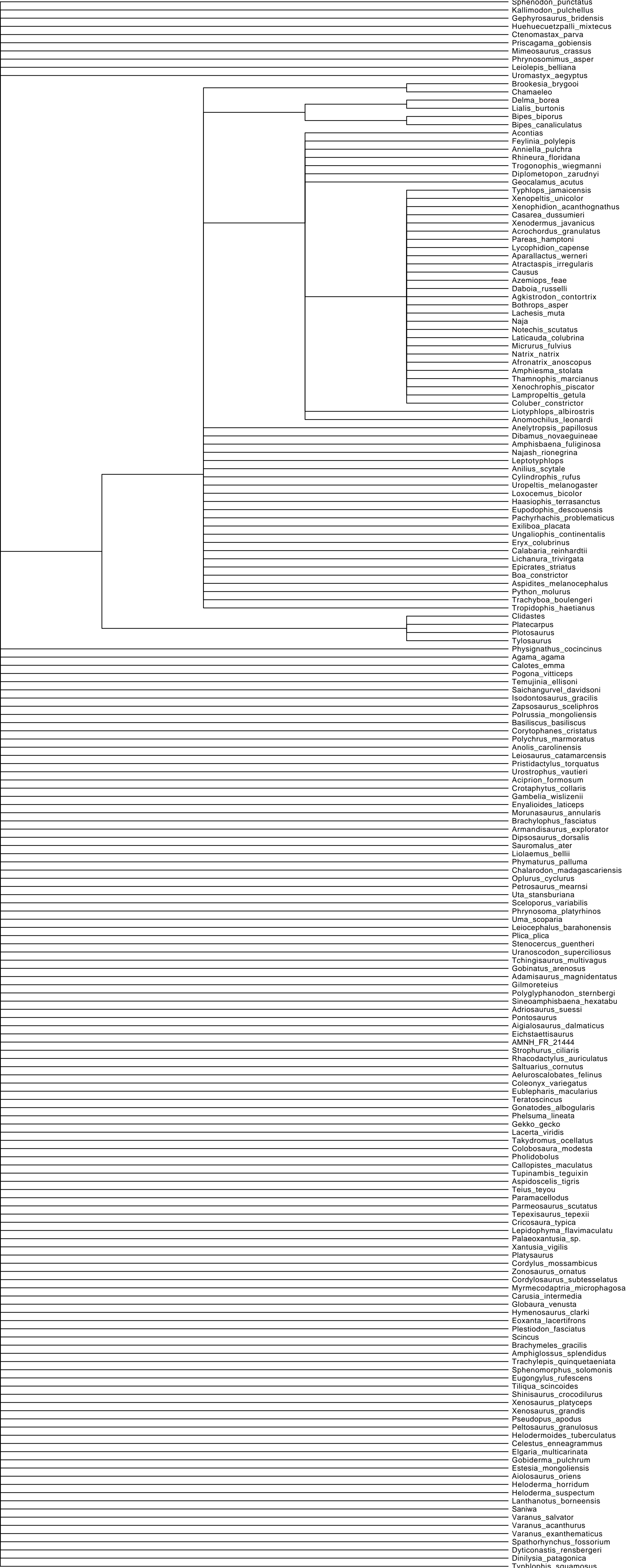

0.6

Supplement: S56 Fig — Strict consensus of 10,000 shortest trees (maximum number of trees retained) of length 481. See S67 Fig. for bootstrap values. (PDF) [file pone.0118199.s058.pdf]

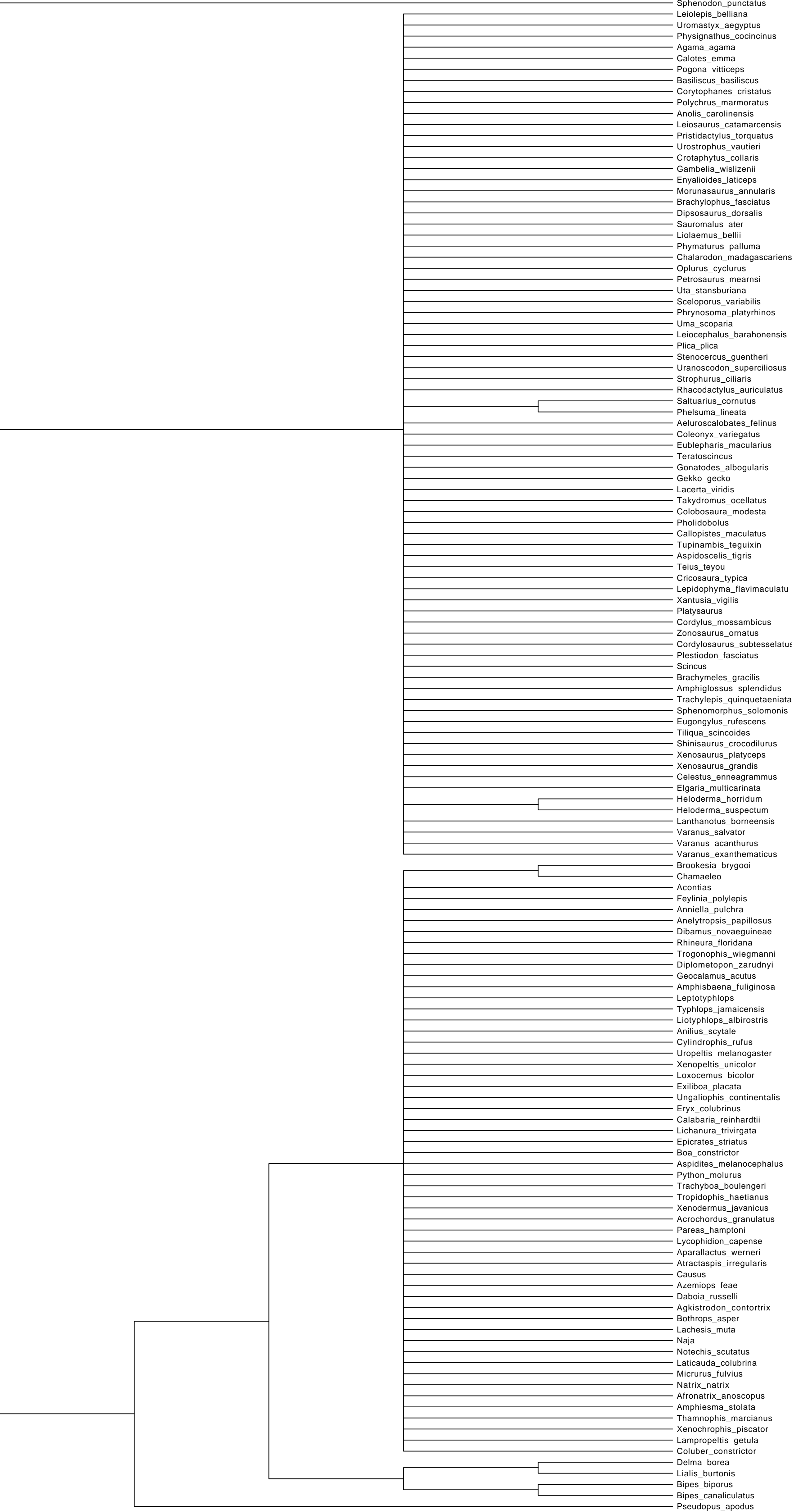

Supplement: S57 Fig — Strict consensus of 10,000 shortest trees (maximum number of trees retained) of length 442. See S68 Fig. for bootstrap values. (PDF) [file pone.0118199.s059.pdf]

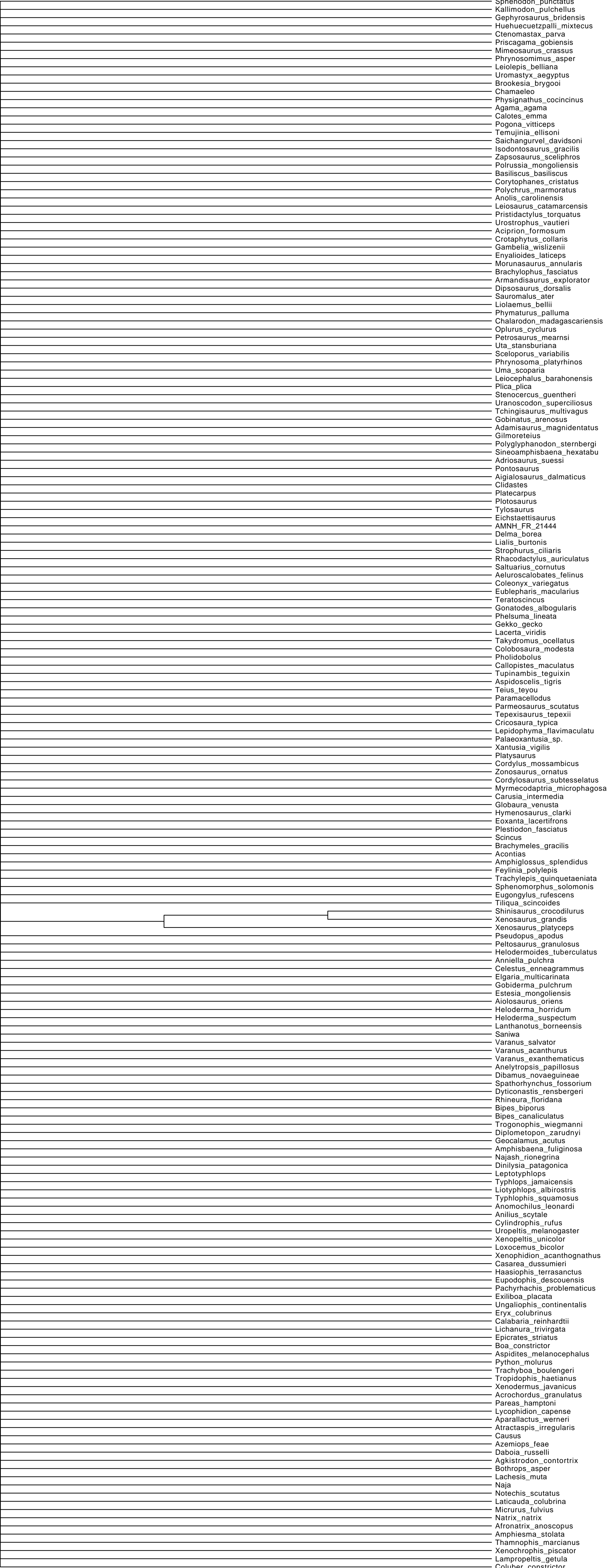

Supplement: S58 Fig — Strict consensus of 10,000 shortest trees (maximum number of trees retained) of length 188. See S69 Fig. for bootstrap values. (PDF) [file pone.0118199.s060.pdf]

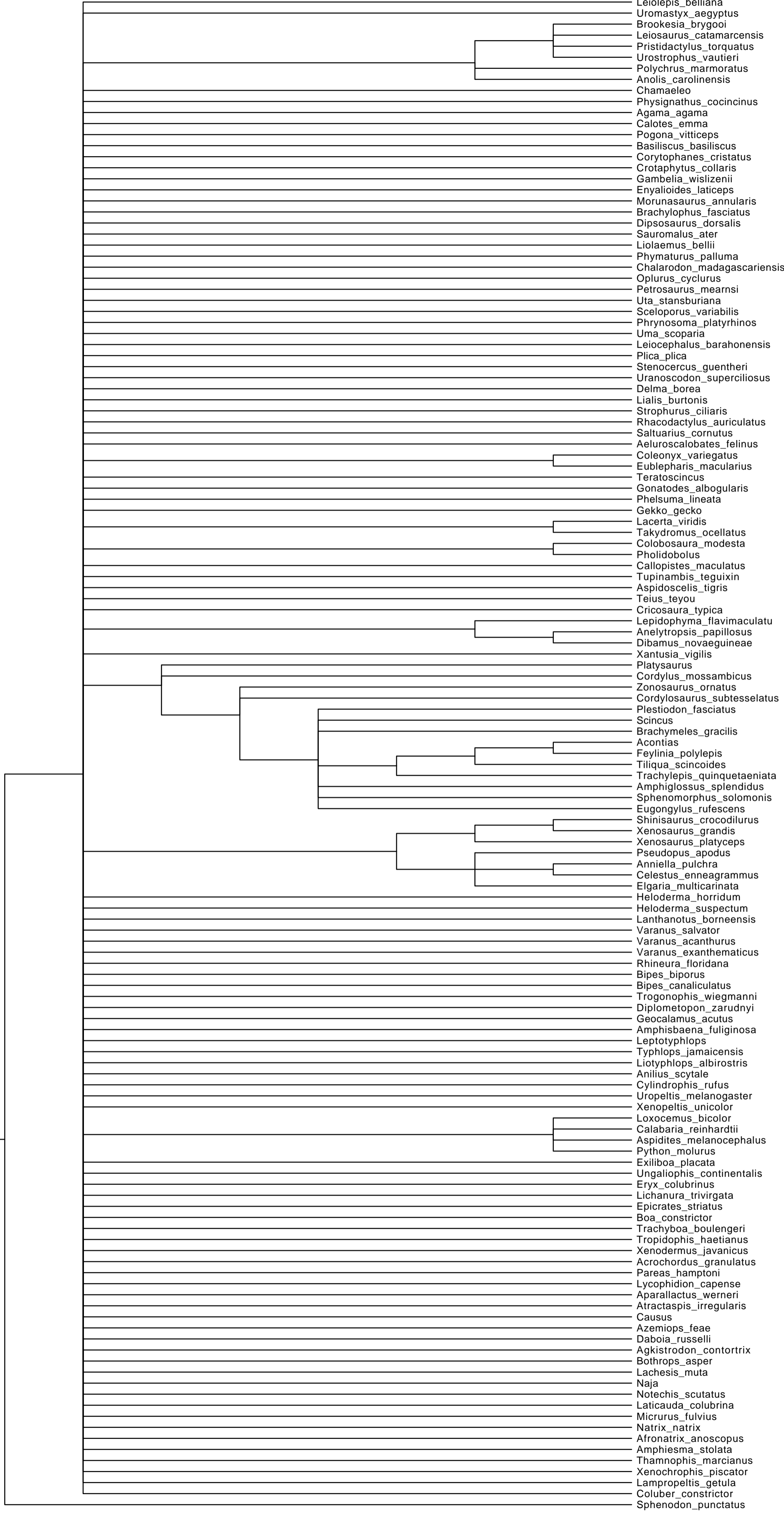

0.8

Supplement: S59 Fig — Strict consensus of 10,000 shortest trees (maximum number of trees retained) of length 181. See S70 Fig. for bootstrap values. (PDF) [file pone.0118199.s061.pdf]

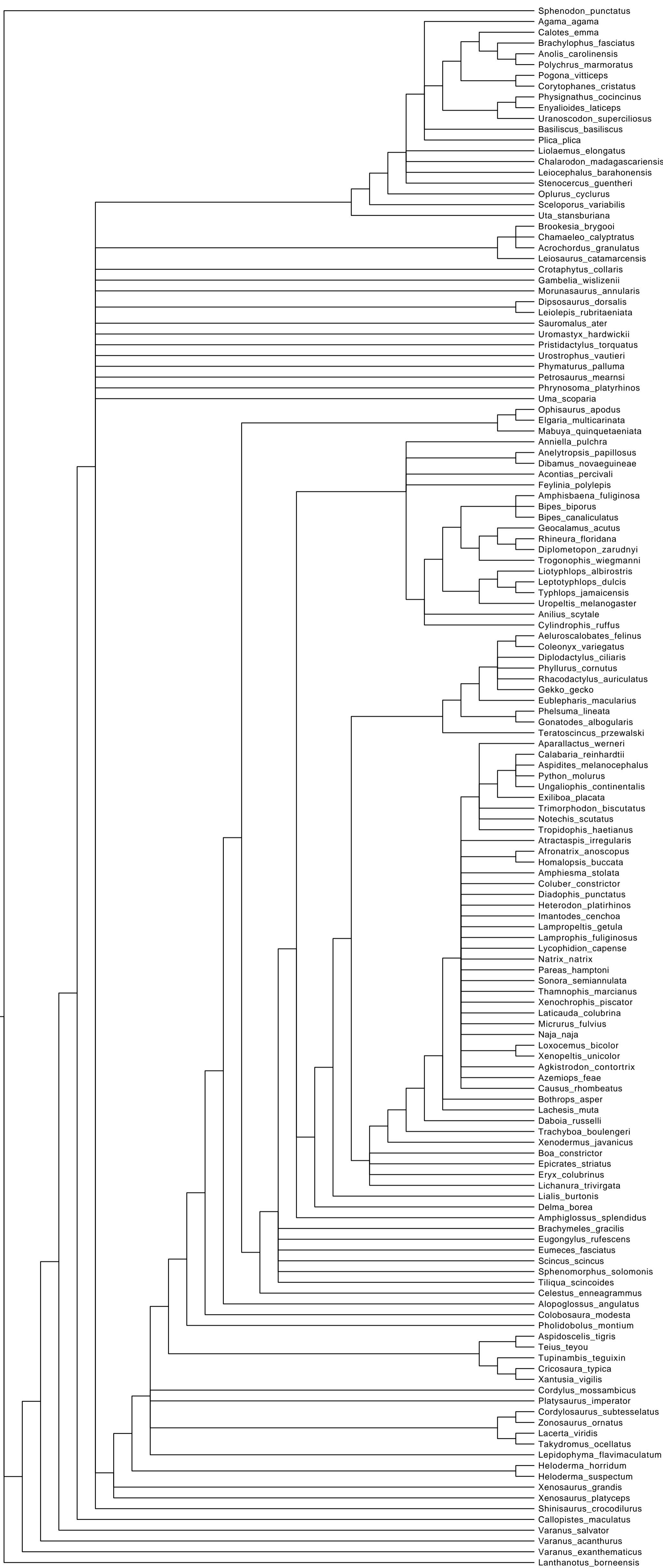

Supplement: S60 Fig — Strict consensus of 10,000 shortest trees (maximum number of trees retained) of length 660. See S71 Fig. for bootstrap values. (PDF) [file pone.0118199.s062.pdf]

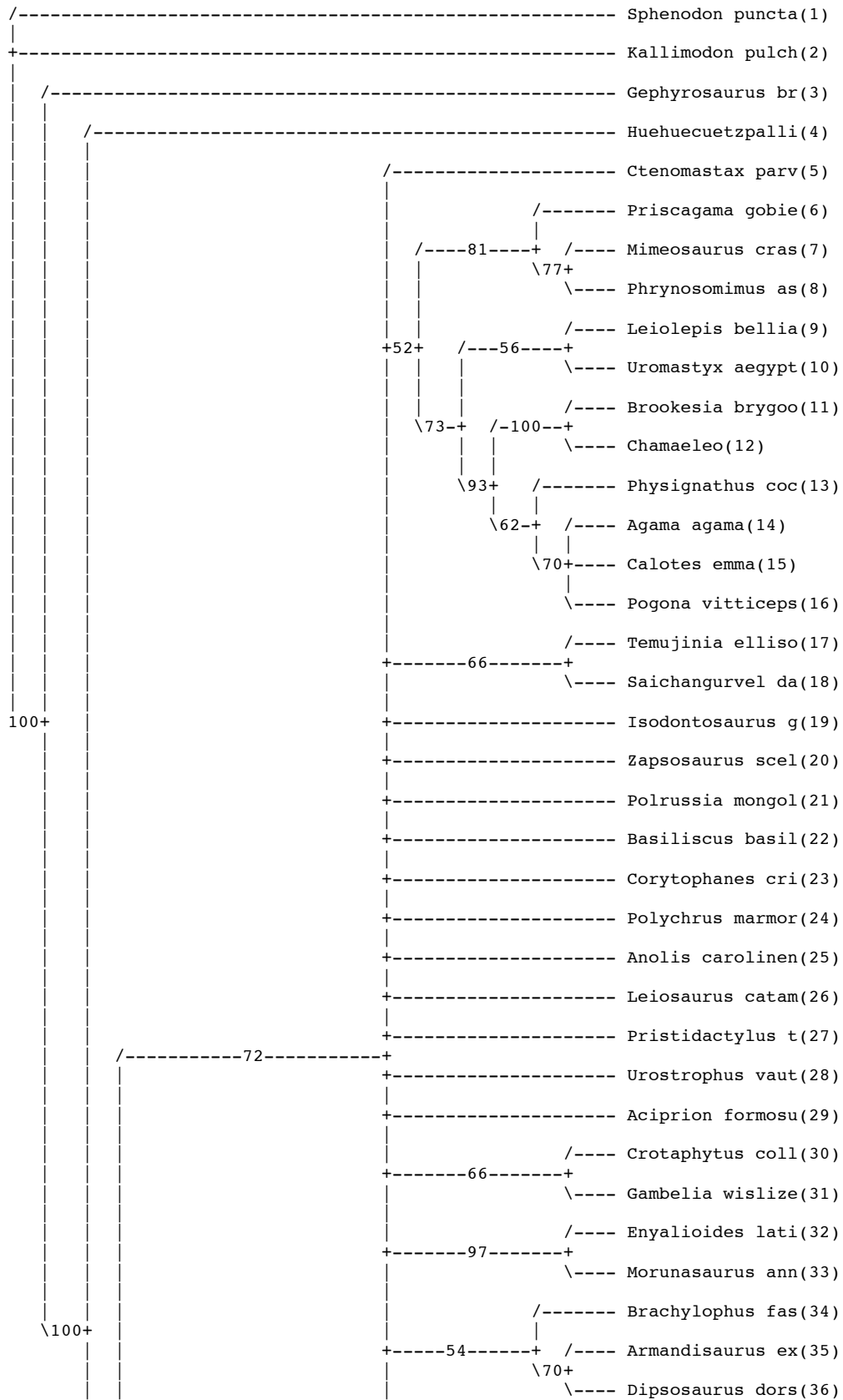

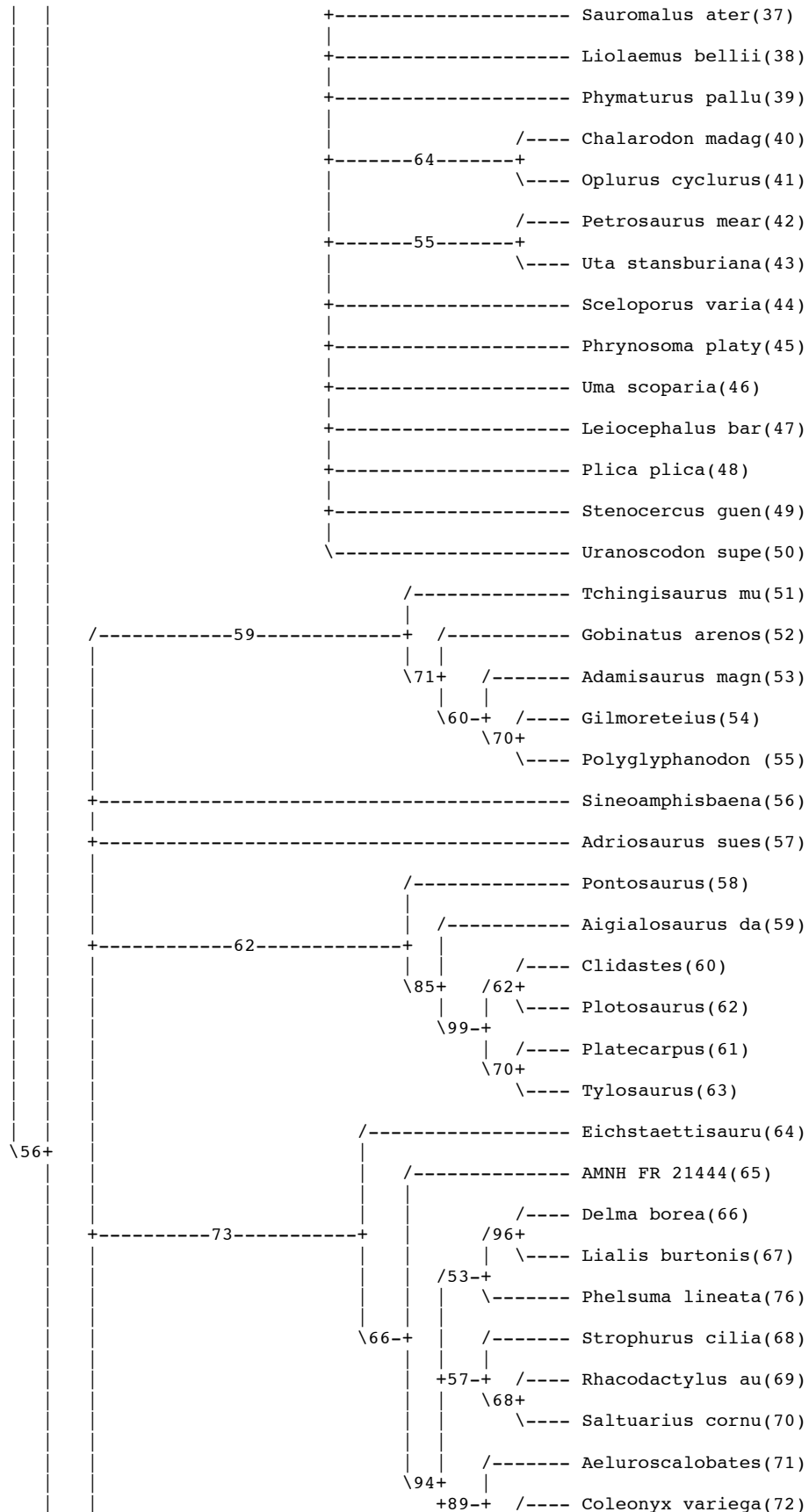

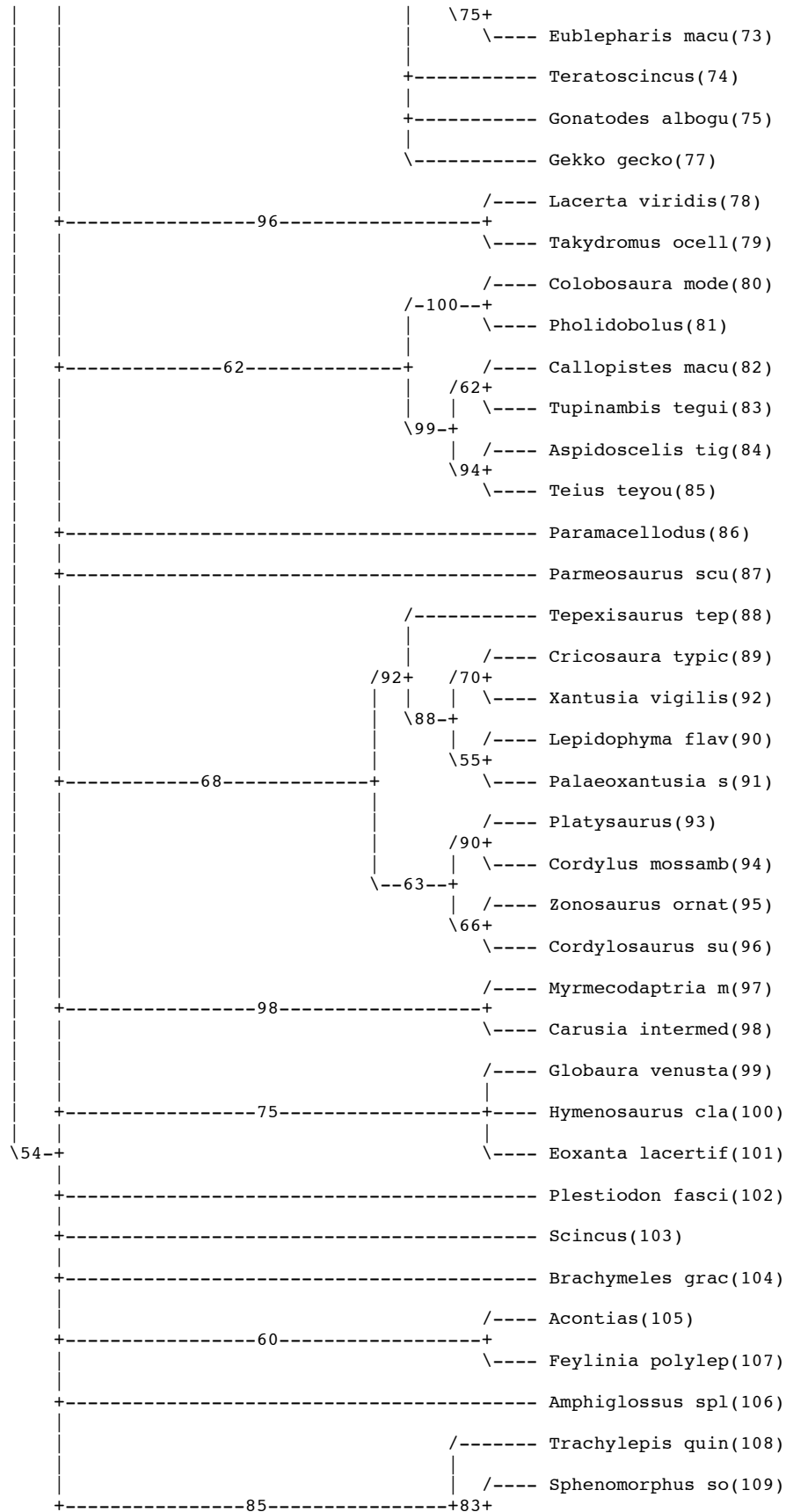

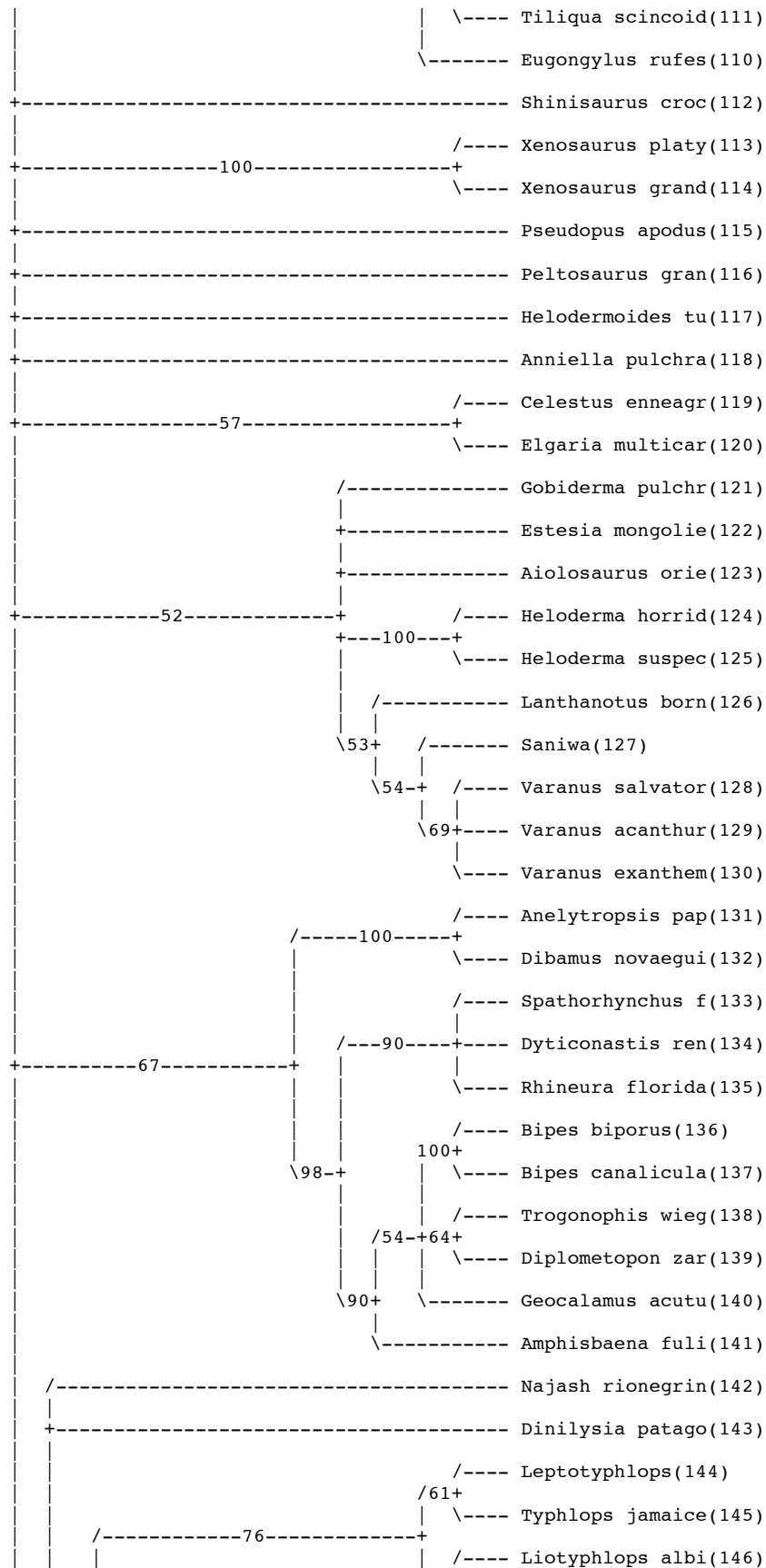

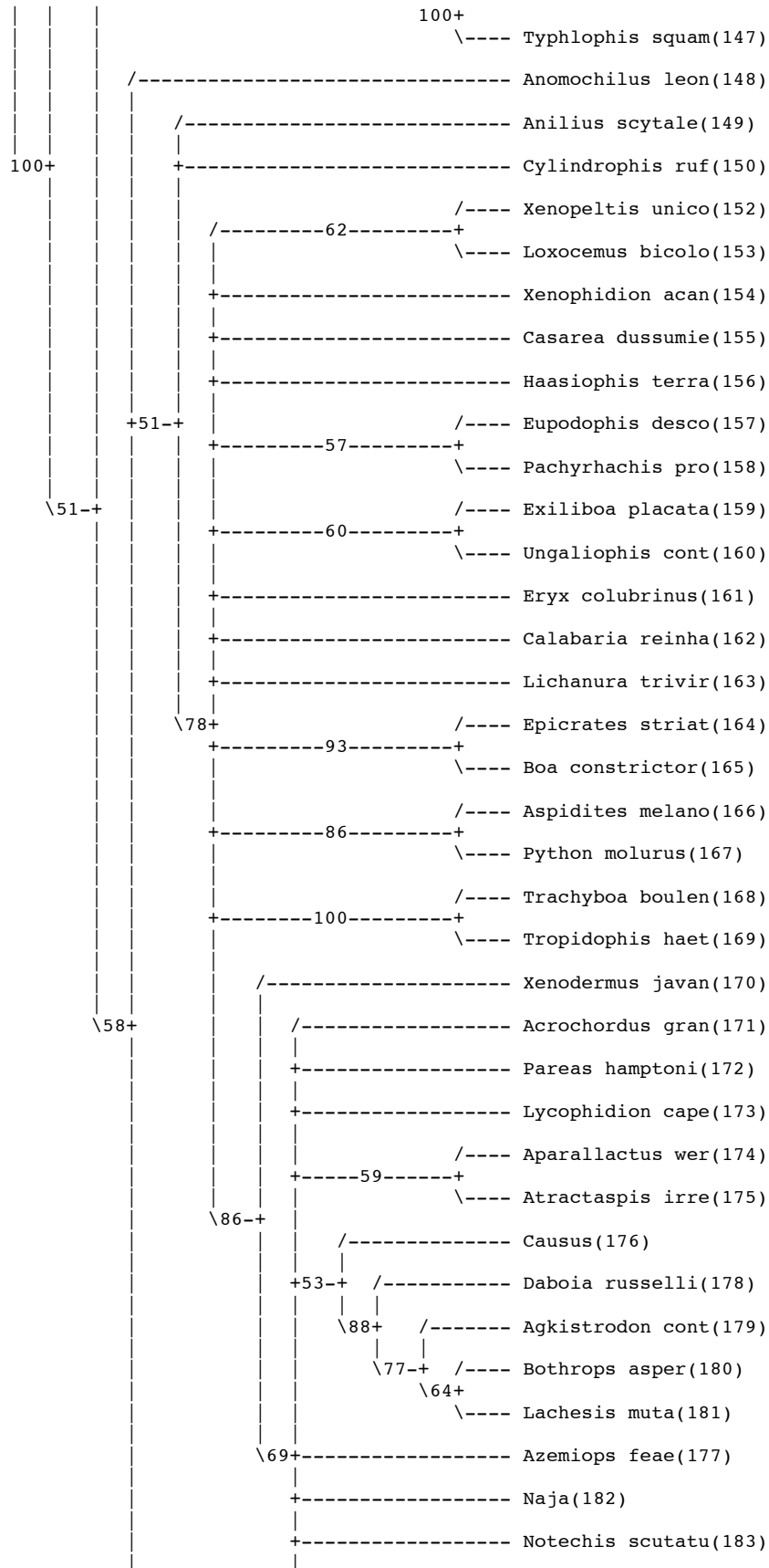

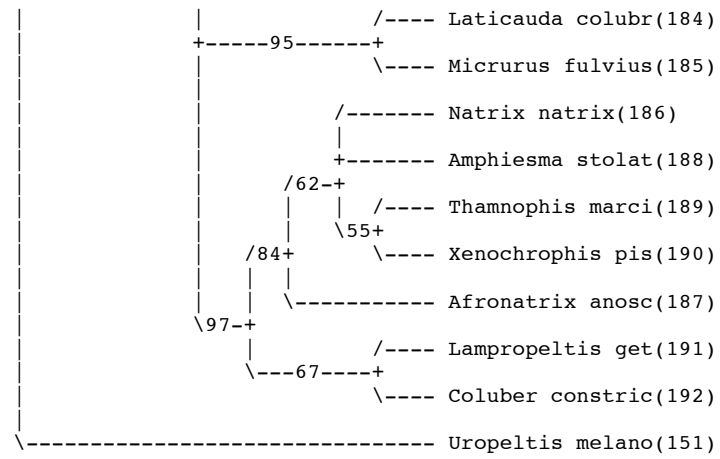

Supplement: S61 Fig — (PDF) [file pone.0118199.s063.pdf]

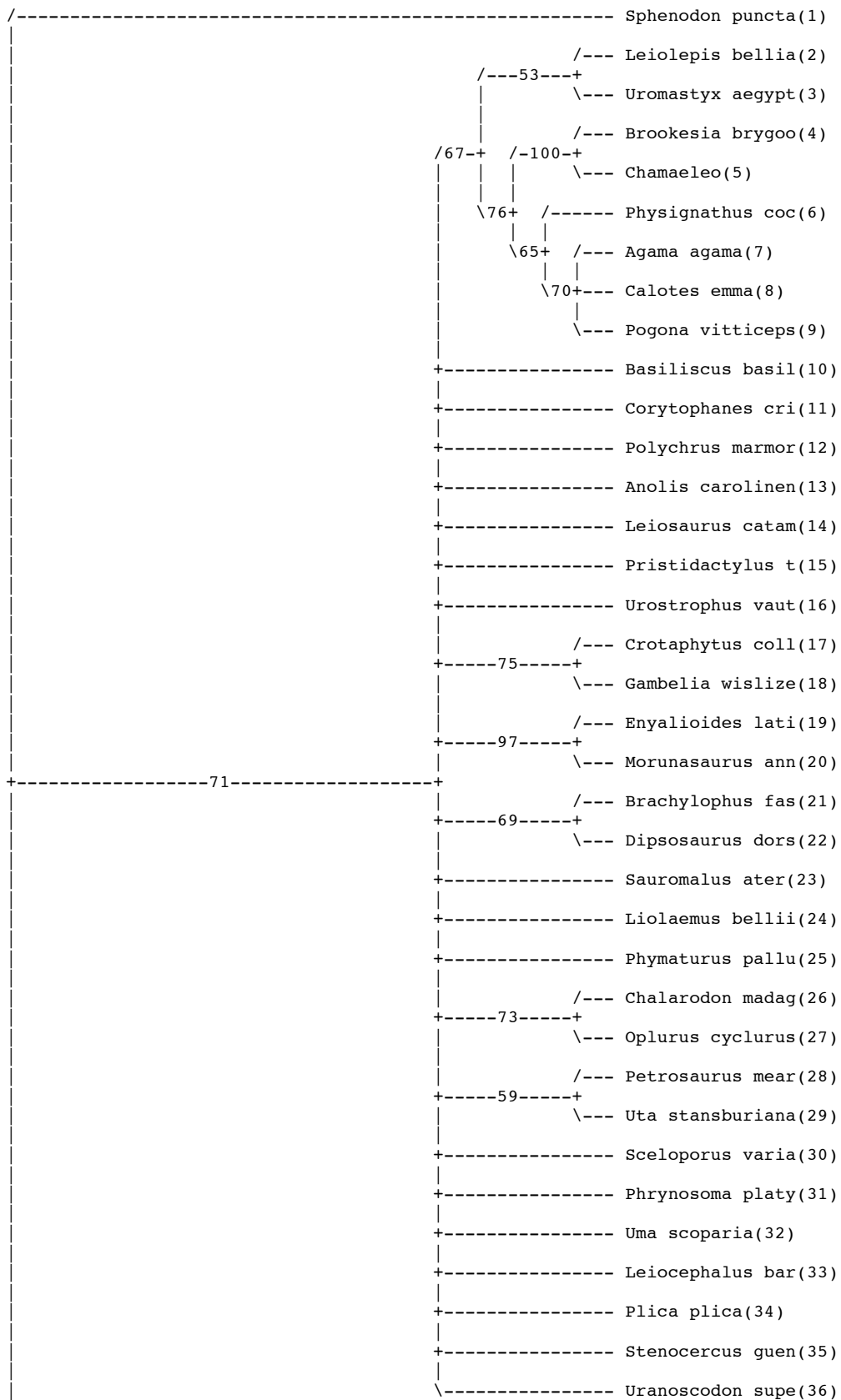

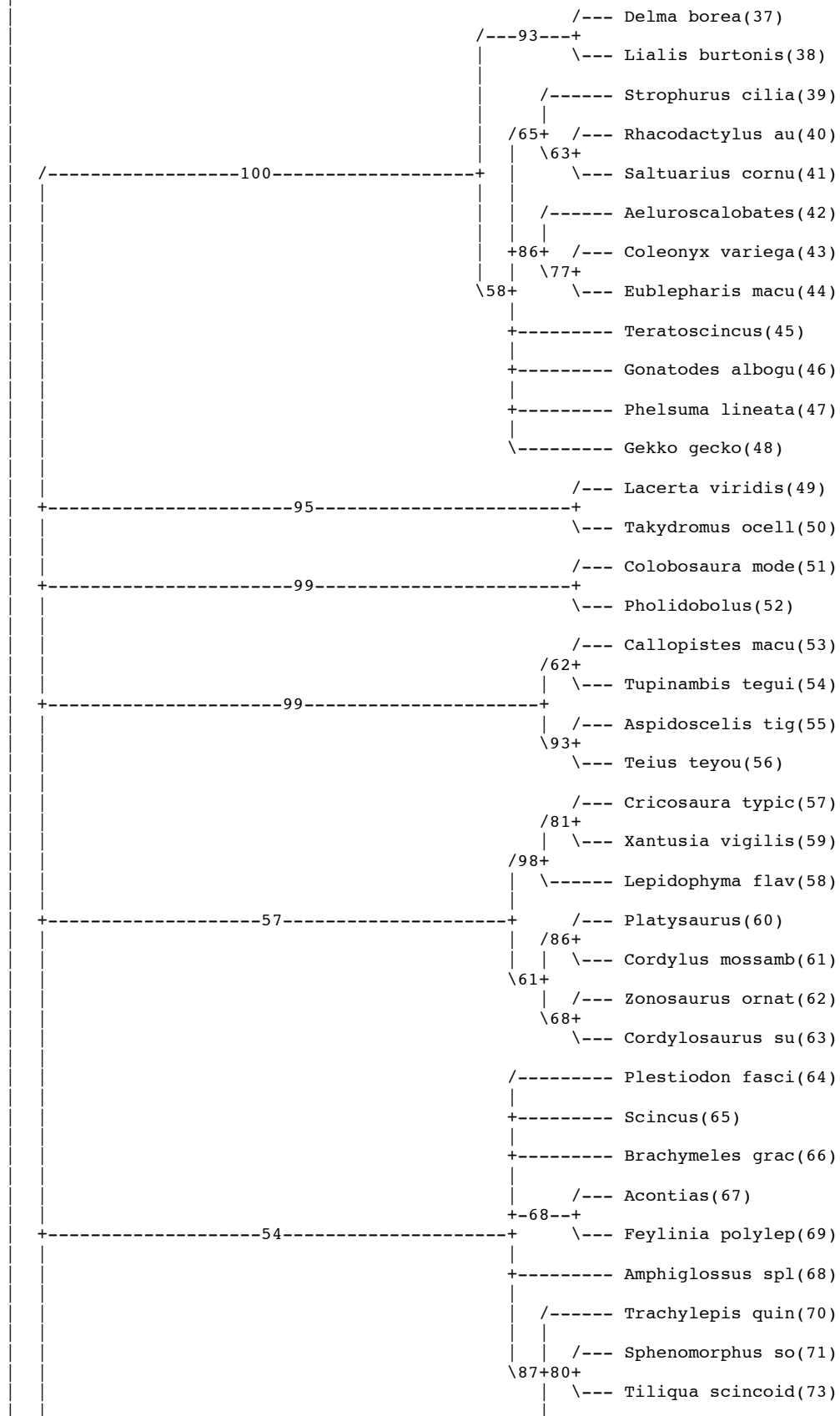

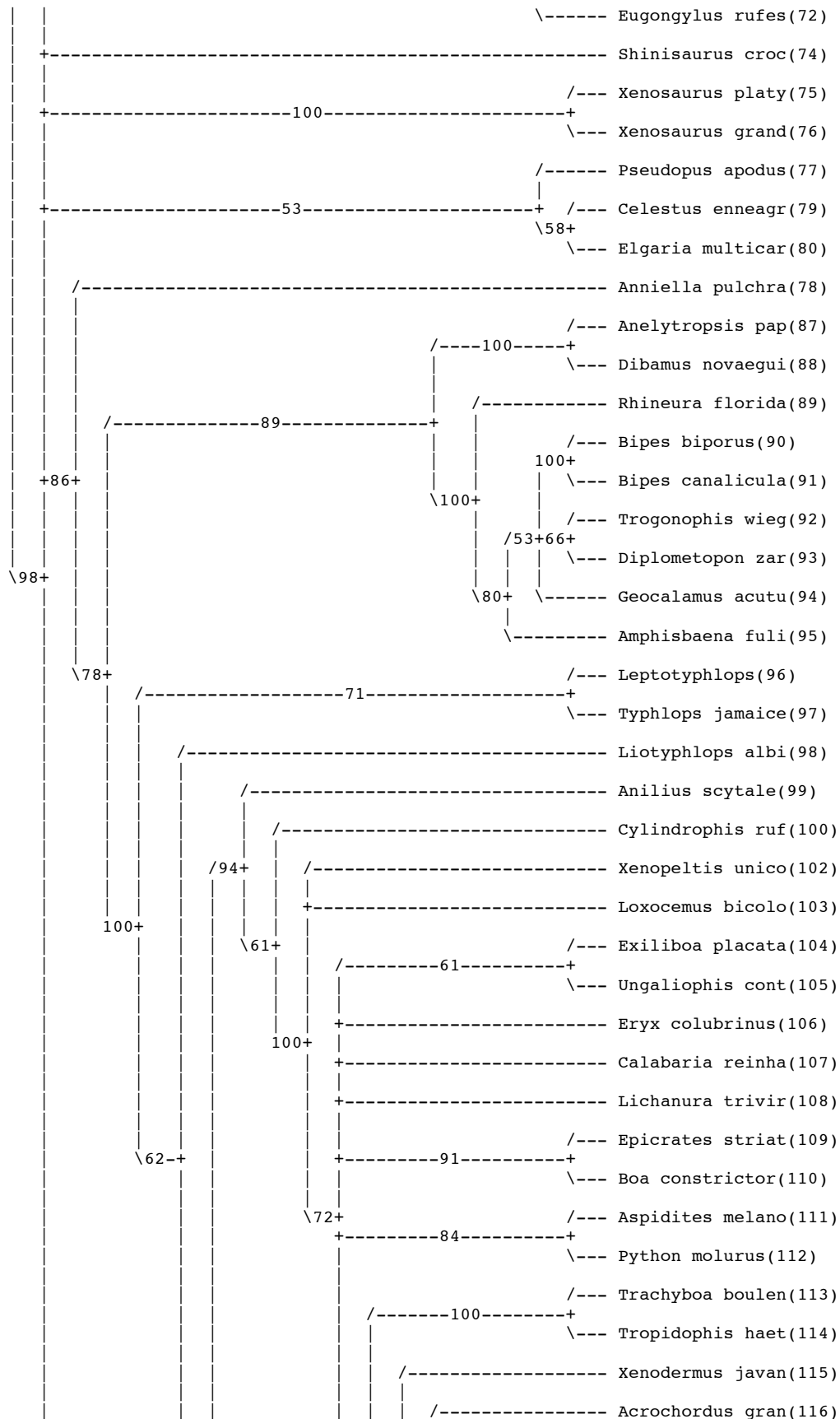

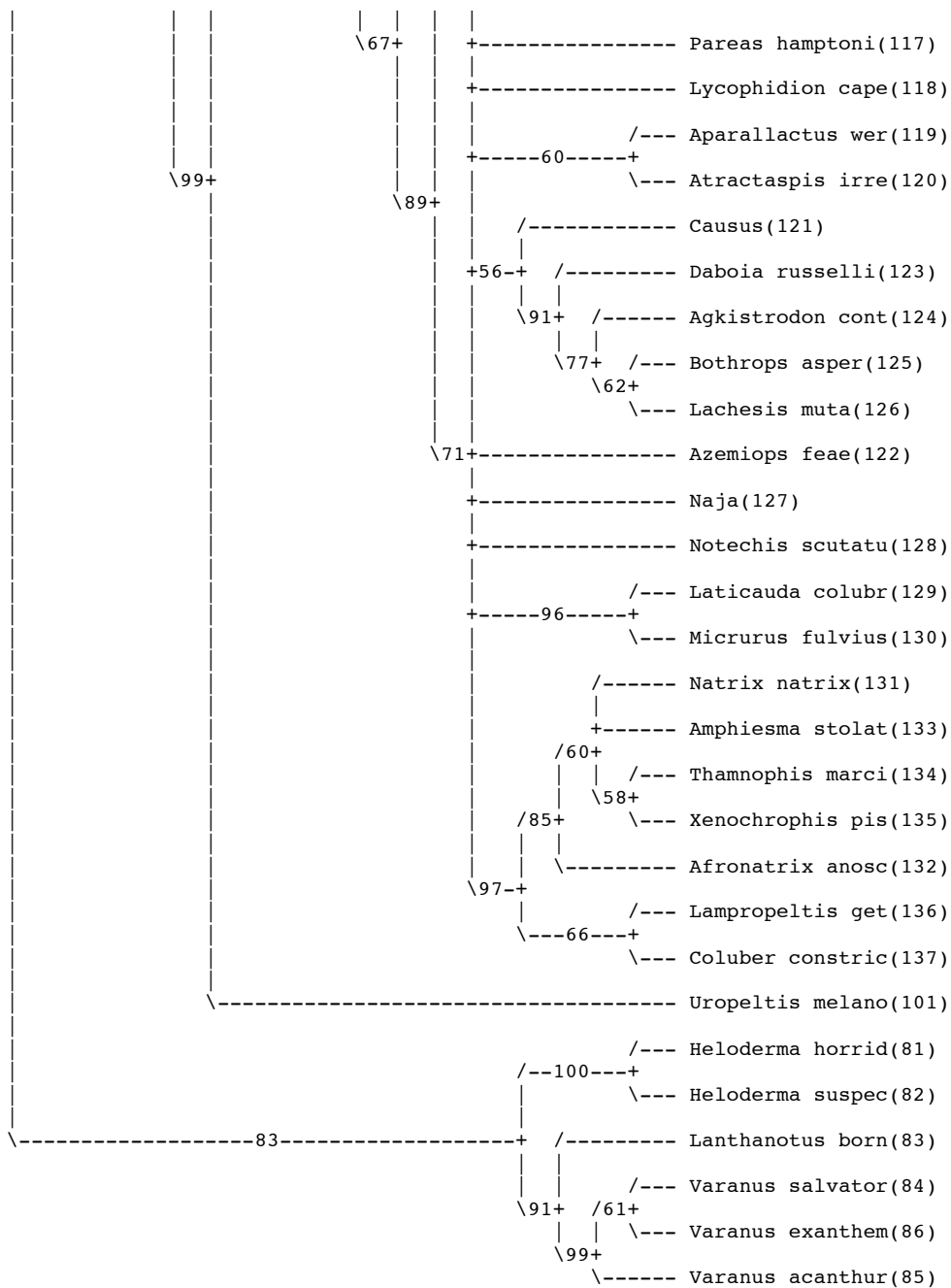

Supplement: S62 Fig — (PDF) [file pone.0118199.s064.pdf]
