# Supplementary material for: Integrated Analyses Resolve Conflicts over Squamate Reptile Phylogeny and Reveal Unexpected Placements for Fossil Taxa
Source: PLoS One. 2015 Mar 24;10(3):e0118199. doi: 10.1371/journal.pone.0118199 (PMC4372529; doi:10.1371/journal.pone.0118199)
Supplement: S54 Fig — Strict consensus of 10,000 shortest trees (maximum number of trees retained) of length 173. See S65 Fig. for bootstrap values. (PDF) [file pone.0118199.s056.pdf]

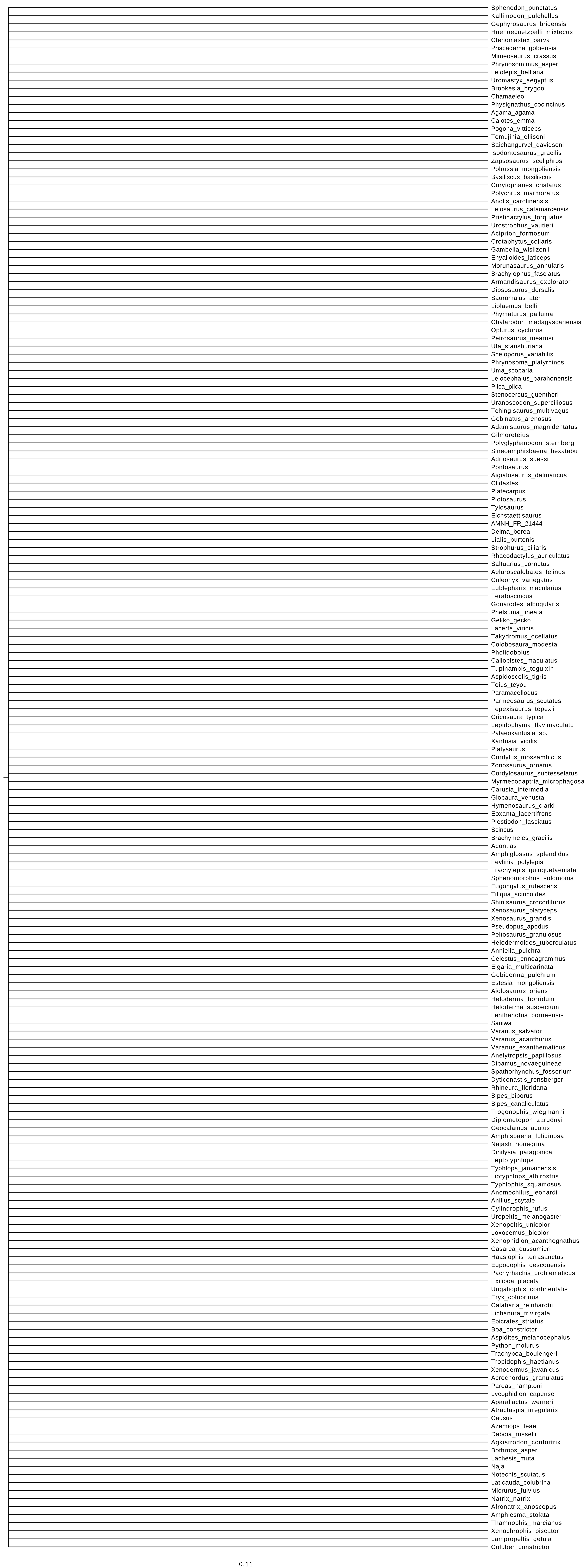
Sphenodon_punctatus
Kallimodon_pulchellus
Gephyrosaurus_bridensis
Huehuecuetzpalli_mixtecus
Ctenomastax_parva
Priscagama_gobiensis
Mimeosaurus_crassus
Phrynosomimus_asper
Leiolepis_belliana
Uromastyx_aegyptus
Brookesia_brygooi
Chamaeleo
Physignathus_cocincinus
Agama_agama
Calotes_emma
Pogona_vitticeps
Temujinia_ellisoni
Saichangurvel_davidsoni
Isodontosaurus_gracilis
Zapsosaurus_sceliphros
Polrussia_mongoliensis
Basiliscus_basiliscus
Corytophanes_cristatus
Polychrus_marmoratus
Anolis_carolinensis
Leiosaurus_catamarcensis
Pristidactylus_torquatus
Urostrophus_vautieri
Aciprion_formosum
Crotaphytus_collaris
Gambelia_wislizenii
Enyalioides_laticeps
Morunasaurus_annularis
Brachylophus_fasciatus
Armandisaurus_explorator
Dipsosaurus_dorsalis
Sauromalus_ater
Liolaemus_bellii
Phymaturus_palluma
Chalarodon_madagascariensis
Oplurus_cyclurus
Petrosaurus_mearnsi
Uta_stansburiana
Sceloporus_variabilis
Phrynosoma_platyrhinos
Uma_scoparia
Leiocephalus_barahonensis
Plica_plica
Stenocercus_guentheri
Uranoscodon_superciliosus
Tchingisaurus_multivagus
Gobinatus_arenosus
Adamisaurus_magnidentatus
Gilmoreteius
Polyglyphanodon_sternbergi
Sineoamphisbaena_hexatabu
Adriosaurus_suessi
Pontosaurus
Aigialosaurus_dalmaticus
Clidastes
Platecarpus
Plotosaurus
Tylosaurus
Eichstaettisaurus
AMNH_FR_21444
Delma_borea
Lialis_burtonis
Strophurus_ciliaris
Rhacodactylus_auriculatus
Saltuarius_cornutus
Aeluroscalobates_felinus
Coleonyx_variegatus
Eublepharis_macularius
Teratoscincus
Gonatodes_albogularis
Phelsuma_lineata
Gekko_gecko
Lacerta_viridis
Takydromus_ocellatus
Colobosaura_modesta
Pholidobolus
Callopistes_maculatus
Tupinambis_teguixin
Aspidoscelis_tigris
Teius_teyou
Paramacellodus
Parmeosaurus_scutatus
Tepexisaurus_tepexii
Cricosaura_typica
Lepidophyma_flavimaculatu
Palaeoxantusia_sp.
Xantusia_vigilis
Platysaurus
Cordylus_mossambicus
Zonosaurus_ornatus
Cordylosaurus_subtesselatus
Myrmecodaptria_microphagosa
Carusia_intermedia
Globaura_venusta
Hymenosaurus_clarki
Eoxanta_lacertifrons
Plestiodon_fasciatus
Scincus
Brachymeles_gracilis
Acontias
Amphiglossus_splendidus
Feylinia_polylepis
Trachylepis_quinquetaeniata
Sphenomorphus_solomonis
Eugongylus_rufescens
Tiliqua_scincoides
Shinisaurus_crocodilurus
Xenosaurus_platyceps
Xenosaurus_grandis
Pseudopus_apodus
Peltosaurus_granulosus
Helodermoides_tuberculatus
Anniella_pulchra
Celestus_enneagrammus
Elgaria_multicarinata
Gobiderma_pulchrum
Estesia_mongoliensis
Aiolosaurus_oriens
Heloderma_horridum
Heloderma_suspectum
Lanthanotus_borneensis
Saniwa
Varanus_salvator
Varanus_acanthurus
Varanus_exanthematicus
Anelytropsis_papillosus
Dibamus_novaeguineae
Spathorhynchus_fossorium
Dyticonastis_rensbergeri
Rhineura_floridana
Bipes_biporus
Bipes_canaliculatus
Trogonophis_wiegmanni
Diplometopon_zarudnyi
Geocalamus_acutus
Amphisbaena_fuliginosa
Najash_rionegrina
Dinilysia_patagonica
Leptotyphlops
Typhlops_jamaicensis
Liotyphlops_albirostris
Typhlophis_squamosus
Anomochilus_leonardi
Anilius_scytale
Cylindrophis_rufus
Uropeltis_melanogaster
Xenopeltis_unicolor
Loxocemus_bicolor
Xenophidion_acanthognathus
Casarea_dussumieri
Haasiophis_terrasanctus
Eupodophis_descouensis
Pachyrhachis_problematicus
Exiliboa_placata
Ungaliophis_continentalis
Eryx_colubrinus
Calabaria_reinhardtii
Lichanura_trivirgata
Epicrates_striatus
Boa_constrictor
Aspidites_melanocephalus
Python_molurus
Trachyboa_boulengeri
Tropidophis_haetianus
Xenodermus_javanicus
Acrochordus_granulatus
Pareas_hamptoni
Lycophidion_capense
Aparallactus_werneri
Atractaspis_irregularis
Causus
Azemiops_feae
Daboia_russelli
Agkistrodon_contortrix
Bothrops_asper
Lachesis_muta
Naja
Notechis_scutatus
Laticauda_colubrina
Micrurus_fulvius
Natrix_natrix
Afronatrix_anoscopus
Amphiesma_stolata
Thamnophis_marcianus
Xenochrophis_piscator
Lampropeltis_getula
Coluber_constrictor
0.11
