# Supplementary material for: Integrated Analyses Resolve Conflicts over Squamate Reptile Phylogeny and Reveal Unexpected Placements for Fossil Taxa
Source: PLoS One. 2015 Mar 24;10(3):e0118199. doi: 10.1371/journal.pone.0118199 (PMC4372529; doi:10.1371/journal.pone.0118199)
Supplement: S63 Fig — (PDF) [file pone.0118199.s065.pdf]

```
/-------------------------------------------------------- Sphenodon puncta(1)
|
+-------------------------------------------------------- Kallimodon pulch(2)
|
|          /--------------------------------------------- Gephyrosaurus br(3)
|          |
|          +--------------------------------------------- Huehuecuetzpalli(4)
|          |
|          +--------------------------------------------- Ctenomastax parv(5)
|          |
|          |                                 /----------- Priscagama gobie(6)
|          +---------------53----------------+
|          |                                 \----------- Mimeosaurus cras(7)
|          |
|          +--------------------------------------------- Phrynosomimus as(8)
|          |
|          +--------------------------------------------- Leiolepis bellia(9)
|          |
|          +--------------------------------------------- Uromastyx aegypt(10)
|          |
|          |                                 /----------- Brookesia brygoo(11)
|          +---------------97----------------+
|          |                                 \----------- Chamaeleo(12)
|          |
|          +--------------------------------------------- Physignathus coc(13)
|          |
|          +--------------------------------------------- Agama agama(14)
|          |
|          +--------------------------------------------- Calotes emma(15)
|          |
|          +--------------------------------------------- Pogona vitticeps(16)
|          |
|          +--------------------------------------------- Temujinia elliso(17)
|          |
|          +--------------------------------------------- Saichangurvel da(18)
|          |
|          +--------------------------------------------- Isodontosaurus g(19)
|          |
|          +--------------------------------------------- Zapsosaurus scel(20)
|          |
|          +--------------------------------------------- Polrussia mongol(21)
|          |
|          |                                 /----------- Basiliscus basil(22)
|          +---------------56----------------+
|          |                                 \----------- Corytophanes cri(23)
|          |
|          +--------------------------------------------- Polychrus marmor(24)
|          |
|          +--------------------------------------------- Anolis carolinen(25)
|          |
|          +--------------------------------------------- Leiosaurus catam(26)
|          |
|          +--------------------------------------------- Pristidactylus t(27)
|          |
|          +--------------------------------------------- Urostrophus vaut(28)
|          |
|          +--------------------------------------------- Aciprion formosu(29)
|          |
|          +--------------------------------------------- Crotaphytus coll(30)
|          |
|          +--------------------------------------------- Gambelia wislize(31)
|          |
|          |                                 /----------- Enyalioides lati(32)
|          +---------------51----------------+
|          |                                 \----------- Morunasaurus ann(33)
|          |
|          +--------------------------------------------- Brachylophus fas(34)
|          |
|          +--------------------------------------------- Armandisaurus ex(35)
|          |
|          +--------------------------------------------- Dipsosaurus dors(36)
|          |
```

```
|          +-------------------------------------------- Sauromalus ater(37)
|          |
|          +-------------------------------------------- Liolaemus bellii(38)
|          |
|          +-------------------------------------------- Phymaturus pallu(39)
|          |
|          +-------------------------------------------- Chalarodon madag(40)
|          |
|          +-------------------------------------------- Oplurus cyclurus(41)
|          |
|          +-------------------------------------------- Petrosaurus mear(42)
|          |
|          +-------------------------------------------- Uta stansburiana(43)
|          |
|          +-------------------------------------------- Sceloporus varia(44)
|          |
|          +-------------------------------------------- Phrynosoma platy(45)
|          |
|          +-------------------------------------------- Uma scoparia(46)
|          |
|          +-------------------------------------------- Leiocephalus bar(47)
|          |
|          +-------------------------------------------- Plica plica(48)
|          |
|          +-------------------------------------------- Stenocercus guen(49)
|          |
|          +-------------------------------------------- Uranoscodon supe(50)
|          |
|          +-------------------------------------------- Tchingisaurus mu(51)
|          |
|          +-------------------------------------------- Gobinatus arenos(52)
|          |
|          +-------------------------------------------- Adamisaurus magn(53)
|          |
|          |                                 /---------- Gilmoreteius(54)
|          +--------------78-----------------+
|          |                                 \---------- Polyglyphanodon (55)
|          |
|          +-------------------------------------------- Sineoamphisbaena(56)
|          |
|          +-------------------------------------------- Adriosaurus sues(57)
|          |
|          |          /--------------------------------- Pontosaurus(58)
|          |          |
|          |          |           /--------------------- Aigialosaurus da(59)
|          +----50----+           |
|          |          |           |          /---------- Clidastes(60)
|          |          \----52-----+          |
|          |                      |          +---------- Platecarpus(61)
|          |                      \----64----+
|          |                                 +---------- Plotosaurus(62)
|          |                                 |
|          |                                 \---------- Tylosaurus(63)
|          |
|          +-------------------------------------------- Eichstaettisauru(64)
|          |
|          +-------------------------------------------- AMNH FR 21444(65)
|          |
|          |                                 /---------- Delma borea(66)
|          +--------------75-----------------+
|          |                                 \---------- Lialis burtonis(67)
|          |
|          +-------------------------------------------- Strophurus cilia(68)
|          |
|          +-------------------------------------------- Rhacodactylus au(69)
|          |
|          +-------------------------------------------- Saltuarius cornu(70)
|          |
|          +-------------------------------------------- Aeluroscalobates(71)
|          |
|          +-------------------------------------------- Coleonyx variega(72)
|          |
|          +-------------------------------------------- Eublepharis macu(73)
```

```
|          |
|          +--------------------------------------------- Teratoscincus(74)
|          |
|          +--------------------------------------------- Gonatodes albogu(75)
|          |
|          +--------------------------------------------- Phelsuma lineata(76)
|          |
|          +--------------------------------------------- Gekko gecko(77)
|          |
|          +--------------------------------------------- Lacerta viridis(78)
|          |
|          +--------------------------------------------- Takydromus ocell(79)
|          |
|          |                                 /----------- Colobosaura mode(80)
|          +---------------59----------------+
|          |                                 \----------- Pholidobolus(81)
|          |
|          +--------------------------------------------- Callopistes macu(82)
|          |
|          +--------------------------------------------- Tupinambis tegui(83)
|          |
|          +--------------------------------------------- Aspidoscelis tig(84)
|          |
|          +--------------------------------------------- Teius teyou(85)
|          |
|          +--------------------------------------------- Paramacellodus(86)
|          |
|          +--------------------------------------------- Parmeosaurus scu(87)
|          |
|          +--------------------------------------------- Tepexisaurus tep(88)
|          |
|          |          /---------------------------------- Cricosaura typic(89)
|          |          |
|          |          |                      /----------- Lepidophyma flav(90)
|          +----78----+           /----77----+
|          |          |           |          \----------- Palaeoxantusia s(91)
|          |          \----62-----+
|          |                      \---------------------- Xantusia vigilis(92)
|          |
|          |                                 /----------- Platysaurus(93)
|          +---------------67----------------+
|          |                                 \----------- Cordylus mossamb(94)
|          |
|          +--------------------------------------------- Zonosaurus ornat(95)
|          |
|          +--------------------------------------------- Cordylosaurus su(96)
|          |
|          +--------------------------------------------- Myrmecodaptria m(97)
\----82----+
           +--------------------------------------------- Carusia intermed(98)
           |
           +--------------------------------------------- Globaura venusta(99)
           |
           +--------------------------------------------- Hymenosaurus cla(100)
           |
           +--------------------------------------------- Eoxanta lacertif(101)
           |
           +--------------------------------------------- Plestiodon fasci(102)
           |
           +--------------------------------------------- Scincus(103)
           |
           |                                 /----------- Brachymeles grac(104)
           +---------------59----------------+
           |                                 \----------- Amphiglossus spl(106)
           |
           +--------------------------------------------- Acontias(105)
           |
           +--------------------------------------------- Feylinia polylep(107)
           |
           +--------------------------------------------- Trachylepis quin(108)
           |
           +--------------------------------------------- Sphenomorphus so(109)
           |
```

```
+--------------------------------------------- Eugongylus rufes(110)
|
+--------------------------------------------- Tiliqua scincoid(111)
|
+--------------------------------------------- Shinisaurus croc(112)
|
|                                 /----------- Xenosaurus platy(113)
+---------------79----------------+
|                                 \----------- Xenosaurus grand(114)
|
+--------------------------------------------- Pseudopus apodus(115)
|
|                                 /----------- Peltosaurus gran(116)
+---------------59----------------+
|                                 \----------- Helodermoides tu(117)
|
+--------------------------------------------- Anniella pulchra(118)
|
+--------------------------------------------- Celestus enneagr(119)
|
+--------------------------------------------- Elgaria multicar(120)
|
|                      /---------------------- Gobiderma pulchr(121)
|                      |
|                      +---------------------- Estesia mongolie(122)
|                      |
|                      +---------------------- Aiolosaurus orie(123)
|                      |
|                      |          /----------- Heloderma horrid(124)
|                      +----97----+
|                      |          \----------- Heloderma suspec(125)
+----------80----------+
|                      +---------------------- Lanthanotus born(126)
|                      |
|                      +---------------------- Saniwa(127)
|                      |
|                      +---------------------- Varanus salvator(128)
|                      |
|                      +---------------------- Varanus acanthur(129)
|                      |
|                      \---------------------- Varanus exanthem(130)
|
|                                 /----------- Anelytropsis pap(131)
+---------------84----------------+
|                                 \----------- Dibamus novaegui(132)
|
|                                 /----------- Spathorhynchus f(133)
|                      /----65----+
|                      |          \----------- Dyticonastis ren(134)
+----------89----------+
|                      \---------------------- Rhineura florida(135)
|
|                                 /----------- Bipes biporus(136)
|          /----------93----------+
|          |                      \----------- Bipes canalicula(137)
|          |
|          |                      /----------- Trogonophis wieg(138)
+----84----+           /----98----+
|          |           |          \----------- Diplometopon zar(139)
|          |           |
|          \----62-----+---------------------- Geocalamus acutu(140)
|                      |
|                      \---------------------- Amphisbaena fuli(141)
|
+--------------------------------------------- Najash rionegrin(142)
|
+--------------------------------------------- Dinilysia patago(143)
|
+--------------------------------------------- Leptotyphlops(144)
|
+--------------------------------------------- Typhlops jamaice(145)
|
|                                 /----------- Liotyphlops albi(146)
```

```
+---------------75----------------+
|                                 \----------- Typhlophis squam(147)
|
+--------------------------------------------- Anomochilus leon(148)
|
+--------------------------------------------- Anilius scytale(149)
|
+--------------------------------------------- Cylindrophis ruf(150)
|
+--------------------------------------------- Uropeltis melano(151)
|
+--------------------------------------------- Xenopeltis unico(152)
|
+--------------------------------------------- Loxocemus bicolo(153)
|
+--------------------------------------------- Xenophidion acan(154)
|
+--------------------------------------------- Casarea dussumie(155)
|
+--------------------------------------------- Haasiophis terra(156)
|
+--------------------------------------------- Eupodophis desco(157)
|
+--------------------------------------------- Pachyrhachis pro(158)
|
+--------------------------------------------- Exiliboa placata(159)
|
+--------------------------------------------- Ungaliophis cont(160)
|
+--------------------------------------------- Eryx colubrinus(161)
|
+--------------------------------------------- Calabaria reinha(162)
|
+--------------------------------------------- Lichanura trivir(163)
|
+--------------------------------------------- Epicrates striat(164)
|
+--------------------------------------------- Boa constrictor(165)
|
+--------------------------------------------- Aspidites melano(166)
|
+--------------------------------------------- Python molurus(167)
|
+--------------------------------------------- Trachyboa boulen(168)
|
+--------------------------------------------- Tropidophis haet(169)
|
+--------------------------------------------- Xenodermus javan(170)
|
+--------------------------------------------- Acrochordus gran(171)
|
+--------------------------------------------- Pareas hamptoni(172)
|
+--------------------------------------------- Lycophidion cape(173)
|
+--------------------------------------------- Aparallactus wer(174)
|
+--------------------------------------------- Atractaspis irre(175)
|
+--------------------------------------------- Causus(176)
|
+--------------------------------------------- Azemiops feae(177)
|
+--------------------------------------------- Daboia russelli(178)
|
+--------------------------------------------- Agkistrodon cont(179)
|
+--------------------------------------------- Bothrops asper(180)
|
+--------------------------------------------- Lachesis muta(181)
|
+--------------------------------------------- Naja(182)
|
```

```
+--------------------------------------------- Notechis scutatu(183)
|
+--------------------------------------------- Laticauda colubr(184)
|
+--------------------------------------------- Micrurus fulvius(185)
|
+--------------------------------------------- Natrix natrix(186)
|
+--------------------------------------------- Afronatrix anosc(187)
|
+--------------------------------------------- Amphiesma stolat(188)
|
+--------------------------------------------- Thamnophis marci(189)
|
+--------------------------------------------- Xenochrophis pis(190)
|
+--------------------------------------------- Lampropeltis get(191)
|
\--------------------------------------------- Coluber constric(192)
```
