# Supplementary material for: Integrated Analyses Resolve Conflicts over Squamate Reptile Phylogeny and Reveal Unexpected Placements for Fossil Taxa
Source: PLoS One. 2015 Mar 24;10(3):e0118199. doi: 10.1371/journal.pone.0118199 (PMC4372529; doi:10.1371/journal.pone.0118199)
Supplement: S64 Fig — (PDF) [file pone.0118199.s066.pdf]

```
/-------------------------------------------------------- Sphenodon puncta(1)
|
+-------------------------------------------------------- Leiolepis bellia(2)
|
+-------------------------------------------------------- Uromastyx aegypt(3)
|
|                                              /--------- Brookesia brygoo(4)
+----------------------98----------------------+
|                                              \--------- Chamaeleo(5)
|
+-------------------------------------------------------- Physignathus coc(6)
|
+-------------------------------------------------------- Agama agama(7)
|
+-------------------------------------------------------- Calotes emma(8)
|
+-------------------------------------------------------- Pogona vitticeps(9)
|
|                                              /--------- Basiliscus basil(10)
|        /-----------------65------------------+
|        |                                     \--------- Corytophanes cri(11)
|        |
|        +----------------------------------------------- Polychrus marmor(12)
|        |
|        +----------------------------------------------- Anolis carolinen(13)
|        |
|        +----------------------------------------------- Leiosaurus catam(14)
|        |
|        +----------------------------------------------- Pristidactylus t(15)
|        |
|        +----------------------------------------------- Urostrophus vaut(16)
|        |
|        +----------------------------------------------- Crotaphytus coll(17)
|        |
|        +----------------------------------------------- Gambelia wislize(18)
|        |
|        |                                     /--------- Enyalioides lati(19)
|        +-----------------55------------------+
|        |                                     \--------- Morunasaurus ann(20)
|        |
|        +----------------------------------------------- Brachylophus fas(21)
|        |
|        +----------------------------------------------- Dipsosaurus dors(22)
|        |
|        +----------------------------------------------- Sauromalus ater(23)
|        |
|        +----------------------------------------------- Liolaemus bellii(24)
|        |
|        +----------------------------------------------- Phymaturus pallu(25)
|        |
|        +----------------------------------------------- Chalarodon madag(26)
|        |
|        +----------------------------------------------- Oplurus cyclurus(27)
|        |
|        +----------------------------------------------- Petrosaurus mear(28)
|        |
|        +----------------------------------------------- Uta stansburiana(29)
|        |
|        +----------------------------------------------- Sceloporus varia(30)
|        |
|        +----------------------------------------------- Phrynosoma platy(31)
|        |
|        +----------------------------------------------- Uma scoparia(32)
|        |
|        +----------------------------------------------- Leiocephalus bar(33)
|        |
|        +----------------------------------------------- Plica plica(34)
|        |
|        +----------------------------------------------- Stenocercus guen(35)
|        |
|        +----------------------------------------------- Uranoscodon supe(36)
|        |
```

```
|        |                                     /--------- Delma borea(37)
|        +-----------------80------------------+
|        |                                     \--------- Lialis burtonis(38)
|        |
|        +----------------------------------------------- Strophurus cilia(39)
|        |
|        +----------------------------------------------- Rhacodactylus au(40)
|        |
|        +----------------------------------------------- Saltuarius cornu(41)
|        |
|        |                                     /--------- Aeluroscalobates(42)
|        +-----------------55------------------+
|        |                                     \--------- Eublepharis macu(44)
|        |
|        +----------------------------------------------- Coleonyx variega(43)
|        |
|        +----------------------------------------------- Teratoscincus(45)
|        |
|        +----------------------------------------------- Gonatodes albogu(46)
|        |
|        +----------------------------------------------- Phelsuma lineata(47)
|        |
|        +----------------------------------------------- Gekko gecko(48)
|        |
|        +----------------------------------------------- Lacerta viridis(49)
|        |
|        +----------------------------------------------- Takydromus ocell(50)
|        |
|        |                                     /--------- Colobosaura mode(51)
|        |                           /---61----+
|        |                           |         \--------- Pholidobolus(52)
|        |                           |
|        |                           +------------------- Callopistes macu(53)
|        |                           |
|        +------------55-------------+------------------- Tupinambis tegui(54)
|        |                           |
|        |                           +------------------- Aspidoscelis tig(55)
|        |                           |
|        |                           \------------------- Teius teyou(56)
|        |
|        |                                     /--------- Cricosaura typic(57)
|        |                                     |
|        +-----------------69------------------+--------- Lepidophyma flav(58)
|        |                                     |
|        |                                     \--------- Xantusia vigilis(59)
|        |
|        |                                     /--------- Platysaurus(60)
|        +-----------------79------------------+
|        |                                     \--------- Cordylus mossamb(61)
|        |
|        +----------------------------------------------- Zonosaurus ornat(62)
|        |
|        +----------------------------------------------- Cordylosaurus su(63)
|        |
|        +----------------------------------------------- Plestiodon fasci(64)
|        |
|        +----------------------------------------------- Scincus(65)
|        |
|        |                                     /--------- Brachymeles grac(66)
|        +-----------------53------------------+
|        |                                     \--------- Amphiglossus spl(68)
|        |
|        +----------------------------------------------- Acontias(67)
|        |
|        +----------------------------------------------- Feylinia polylep(69)
|        |
|        +----------------------------------------------- Trachylepis quin(70)
|        |
|        +----------------------------------------------- Sphenomorphus so(71)
|        |
|        +----------------------------------------------- Eugongylus rufes(72)
\---74---+
         +----------------------------------------------- Tiliqua scincoid(73)
```

```
|
+----------------------------------------------- Shinisaurus croc(74)
|
|                                     /--------- Xenosaurus platy(75)
+-----------------90------------------+
|                                     \--------- Xenosaurus grand(76)
|
|                           /------------------- Pseudopus apodus(77)
|                           |
+------------51-------------+         /--------- Celestus enneagr(79)
|                           \---62----+
|                                     \--------- Elgaria multicar(80)
|
+----------------------------------------------- Anniella pulchra(78)
|
|                                     /--------- Heloderma horrid(81)
|                           /---98----+
|                           |         \--------- Heloderma suspec(82)
|                           |
|                           +------------------- Lanthanotus born(83)
|         /-------88--------+
|         |                 |         /--------- Varanus salvator(84)
|         |                 |         |
|         |                 \---71----+--------- Varanus acanthur(85)
|         |                           |
|         |                           \--------- Varanus exanthem(86)
|         |
|         |                 /------------------- Leptotyphlops(96)
|         |                 |
|         |                 |         /--------- Anilius scytale(99)
|         |                 |         |
|         |                 |         +--------- Cylindrophis ruf(100)
|         |                 |         |
|         |                 |         +--------- Uropeltis melano(101)
|         |                 |         |
|         |                 |         +--------- Xenopeltis unico(102)
|         |                 |         |
|         |                 |         +--------- Loxocemus bicolo(103)
|         |                 |         |
|         |                 |         +--------- Exiliboa placata(104)
|         |                 |         |
|         |                 |         +--------- Ungaliophis cont(105)
|         |                 |         |
|         |                 |         +--------- Eryx colubrinus(106)
|         |                 |         |
|         |                 |         +--------- Calabaria reinha(107)
|         |                 |         |
|         |        /---51---+         +--------- Lichanura trivir(108)
|         |        |        |         |
+---54----+        |        |         +--------- Epicrates striat(109)
|         |        |        |         |
|         |        |        |         +--------- Boa constrictor(110)
|         |        |        |         |
|         |        |        |         +--------- Aspidites melano(111)
|         |        |        |         |
|         |        |        |         +--------- Python molurus(112)
|         |        |        |         |
|         |        |        |         +--------- Trachyboa boulen(113)
|         |        |        |         |
|         |        |        |         +--------- Tropidophis haet(114)
|         |        |        |         |
|         |        |        |         +--------- Xenodermus javan(115)
|         |        |        |         |
|         |        |        |         +--------- Acrochordus gran(116)
|         |        |        |         |
|         |        |        |         +--------- Pareas hamptoni(117)
|         |        |        |         |
|         |        |        \---95----+--------- Lycophidion cape(118)
|         |        |                  |
|         |        |                  +--------- Aparallactus wer(119)
|         |        |                  |
|         |        |                  +--------- Atractaspis irre(120)
|         |        |                  |
```

```
|         |        |                 +--------- Causus(121)
|         |        |                 |
|         |        |                 +--------- Azemiops feae(122)
|         |        |                 |
|         |        |                 +--------- Daboia russelli(123)
|         \---88---+                 |
|                  |                 +--------- Agkistrodon cont(124)
|                  |                 |
|                  |                 +--------- Bothrops asper(125)
|                  |                 |
|                  |                 +--------- Lachesis muta(126)
|                  |                 |
|                  |                 +--------- Naja(127)
|                  |                 |
|                  |                 +--------- Notechis scutatu(128)
|                  |                 |
|                  |                 +--------- Laticauda colubr(129)
|                  |                 |
|                  |                 +--------- Micrurus fulvius(130)
|                  |                 |
|                  |                 +--------- Natrix natrix(131)
|                  |                 |
|                  |                 +--------- Afronatrix anosc(132)
|                  |                 |
|                  |                 +--------- Amphiesma stolat(133)
|                  |                 |
|                  |                 +--------- Thamnophis marci(134)
|                  |                 |
|                  |                 +--------- Xenochrophis pis(135)
|                  |                 |
|                  |                 +--------- Lampropeltis get(136)
|                  |                 |
|                  |                 \--------- Coluber constric(137)
|                  |
|                  +--------------------------- Typhlops jamaice(97)
|                  |
|                  \--------------------------- Liotyphlops albi(98)
|
|                                    /--------- Anelytropsis pap(87)
+-----------------83-----------------+
|                                    \--------- Dibamus novaegui(88)
|
+---------------------------------------------- Rhineura florida(89)
|
|                                    /--------- Bipes biporus(90)
|                  /--------90-------+
|                  |                 \--------- Bipes canalicula(91)
|                  |
|                  |                 /--------- Trogonophis wieg(92)
\--------81--------+        /---99---+
                   |        |        \--------- Diplometopon zar(93)
                   |        |
                   \---74---+------------------ Geocalamus acutu(94)
                            |
                            \------------------ Amphisbaena fuli(95)
```
