# Supplementary material for: Integrated Analyses Resolve Conflicts over Squamate Reptile Phylogeny and Reveal Unexpected Placements for Fossil Taxa
Source: PLoS One. 2015 Mar 24;10(3):e0118199. doi: 10.1371/journal.pone.0118199 (PMC4372529; doi:10.1371/journal.pone.0118199)
Supplement: S66 Fig — (PDF) [file pone.0118199.s068.pdf]

```
/-------------------------------------------------------- Sphenodon puncta(1)
|
+-------------------------------------------------------- Leiolepis bellia(2)
|
+-------------------------------------------------------- Uromastyx aegypt(3)
|
|                                         /-------------- Brookesia brygoo(4)
+-------------------87--------------------+
|                                         \-------------- Chamaeleo(5)
|
+-------------------------------------------------------- Physignathus coc(6)
|
|                                         /-------------- Agama agama(7)
+-------------------58--------------------+
|                                         \-------------- Calotes emma(8)
|
+-------------------------------------------------------- Pogona vitticeps(9)
|
+-------------------------------------------------------- Basiliscus basil(10)
|
+-------------------------------------------------------- Corytophanes cri(11)
|
+-------------------------------------------------------- Polychrus marmor(12)
|
+-------------------------------------------------------- Anolis carolinen(13)
|
+-------------------------------------------------------- Leiosaurus catam(14)
|
+-------------------------------------------------------- Pristidactylus t(15)
|
+-------------------------------------------------------- Urostrophus vaut(16)
|
+-------------------------------------------------------- Crotaphytus coll(17)
|
+-------------------------------------------------------- Gambelia wislize(18)
|
+-------------------------------------------------------- Enyalioides lati(19)
|
+-------------------------------------------------------- Morunasaurus ann(20)
|
+-------------------------------------------------------- Brachylophus fas(21)
|
+-------------------------------------------------------- Dipsosaurus dors(22)
|
+-------------------------------------------------------- Sauromalus ater(23)
|
+-------------------------------------------------------- Liolaemus bellii(24)
|
+-------------------------------------------------------- Phymaturus pallu(25)
|
+-------------------------------------------------------- Chalarodon madag(26)
|
+-------------------------------------------------------- Oplurus cyclurus(27)
|
+-------------------------------------------------------- Petrosaurus mear(28)
|
+-------------------------------------------------------- Uta stansburiana(29)
|
+-------------------------------------------------------- Sceloporus varia(30)
|
+-------------------------------------------------------- Phrynosoma platy(31)
|
+-------------------------------------------------------- Uma scoparia(32)
|
+-------------------------------------------------------- Leiocephalus bar(33)
|
+-------------------------------------------------------- Plica plica(34)
|
+-------------------------------------------------------- Stenocercus guen(35)
|
+-------------------------------------------------------- Uranoscodon supe(36)
```

```
|
+-------------------------------------------------------- Delma borea(37)
|
+-------------------------------------------------------- Lialis burtonis(38)
|
+-------------------------------------------------------- Strophurus cilia(39)
|
+-------------------------------------------------------- Rhacodactylus au(40)
|
+-------------------------------------------------------- Saltuarius cornu(41)
|
+-------------------------------------------------------- Aeluroscalobates(42)
|
+-------------------------------------------------------- Coleonyx variega(43)
|
+-------------------------------------------------------- Eublepharis macu(44)
|
+-------------------------------------------------------- Teratoscincus(45)
|
+-------------------------------------------------------- Gonatodes albogu(46)
|
+-------------------------------------------------------- Phelsuma lineata(47)
|
+-------------------------------------------------------- Gekko gecko(48)
|
+-------------------------------------------------------- Lacerta viridis(49)
|
+-------------------------------------------------------- Takydromus ocell(50)
|
+-------------------------------------------------------- Colobosaura mode(51)
|
+-------------------------------------------------------- Pholidobolus(52)
|
+-------------------------------------------------------- Callopistes macu(53)
|
+-------------------------------------------------------- Tupinambis tegui(54)
|
+-------------------------------------------------------- Aspidoscelis tig(55)
|
+-------------------------------------------------------- Teius teyou(56)
|
+-------------------------------------------------------- Cricosaura typic(57)
|
+-------------------------------------------------------- Lepidophyma flav(58)
|
+-------------------------------------------------------- Xantusia vigilis(59)
|
+-------------------------------------------------------- Platysaurus(60)
|
+-------------------------------------------------------- Cordylus mossamb(61)
|
+-------------------------------------------------------- Zonosaurus ornat(62)
|
+-------------------------------------------------------- Cordylosaurus su(63)
|
+-------------------------------------------------------- Plestiodon fasci(64)
|
+-------------------------------------------------------- Scincus(65)
|
+-------------------------------------------------------- Brachymeles grac(66)
|
+-------------------------------------------------------- Acontias(67)
|
+-------------------------------------------------------- Amphiglossus spl(68)
|
+-------------------------------------------------------- Feylinia polylep(69)
|
+-------------------------------------------------------- Trachylepis quin(70)
|
+-------------------------------------------------------- Sphenomorphus so(71)
|
+-------------------------------------------------------- Eugongylus rufes(72)
|
```

```
+-------------------------------------------------------- Tiliqua scincoid(73)
|
+-------------------------------------------------------- Shinisaurus croc(74)
|
+-------------------------------------------------------- Xenosaurus platy(75)
|
+-------------------------------------------------------- Xenosaurus grand(76)
|
|                                         /-------------- Pseudopus apodus(77)
+------------------51---------------------+
|                                         \-------------- Anniella pulchra(78)
|
+-------------------------------------------------------- Celestus enneagr(79)
|
+-------------------------------------------------------- Elgaria multicar(80)
|
+-------------------------------------------------------- Heloderma horrid(81)
|
+-------------------------------------------------------- Heloderma suspec(82)
|
|             /------------------------------------------ Lanthanotus born(83)
|             |
|             |                           /-------------- Varanus salvator(84)
+-----90------+             /-----82------+
|             |             |             \-------------- Varanus exanthem(86)
|             \-----70------+
|                           \---------------------------- Varanus acanthur(85)
|
|                                         /-------------- Anelytropsis pap(87)
+------------------92---------------------+
|                                         \-------------- Dibamus novaegui(88)
|
+-------------------------------------------------------- Rhineura florida(89)
|
+-------------------------------------------------------- Bipes biporus(90)
|
+-------------------------------------------------------- Bipes canalicula(91)
|
+-------------------------------------------------------- Trogonophis wieg(92)
|
+-------------------------------------------------------- Diplometopon zar(93)
|
+-------------------------------------------------------- Geocalamus acutu(94)
|
+-------------------------------------------------------- Amphisbaena fuli(95)
|
|                                         /-------------- Leptotyphlops(96)
+------------------61---------------------+
|                                         \-------------- Pareas hamptoni(117)
|
+-------------------------------------------------------- Typhlops jamaice(97)
|
+-------------------------------------------------------- Liotyphlops albi(98)
|
+-------------------------------------------------------- Anilius scytale(99)
|
+-------------------------------------------------------- Cylindrophis ruf(100)
|
+-------------------------------------------------------- Uropeltis melano(101)
|
+-------------------------------------------------------- Xenopeltis unico(102)
|
+-------------------------------------------------------- Loxocemus bicolo(103)
|
+-------------------------------------------------------- Exiliboa placata(104)
|
+-------------------------------------------------------- Ungaliophis cont(105)
|
|                                         /-------------- Eryx colubrinus(106)
+------------------60---------------------+
|                                         \-------------- Lichanura trivir(108)
|
+-------------------------------------------------------- Calabaria reinha(107)
```

```
|
+-------------------------------------------------------- Epicrates striat(109)
|
+-------------------------------------------------------- Boa constrictor(110)
|
+-------------------------------------------------------- Aspidites melano(111)
|
+-------------------------------------------------------- Python molurus(112)
|
+-------------------------------------------------------- Trachyboa boulen(113)
|
+-------------------------------------------------------- Tropidophis haet(114)
|
+-------------------------------------------------------- Xenodermus javan(115)
|
+-------------------------------------------------------- Acrochordus gran(116)
|
+-------------------------------------------------------- Lycophidion cape(118)
|
+-------------------------------------------------------- Aparallactus wer(119)
|
+-------------------------------------------------------- Atractaspis irre(120)
|
+-------------------------------------------------------- Causus(121)
|
+-------------------------------------------------------- Azemiops feae(122)
|
+-------------------------------------------------------- Daboia russelli(123)
|
+-------------------------------------------------------- Agkistrodon cont(124)
|
+-------------------------------------------------------- Bothrops asper(125)
|
+-------------------------------------------------------- Lachesis muta(126)
|
+-------------------------------------------------------- Naja(127)
|
+-------------------------------------------------------- Notechis scutatu(128)
|
+-------------------------------------------------------- Laticauda colubr(129)
|
+-------------------------------------------------------- Micrurus fulvius(130)
|
+-------------------------------------------------------- Natrix natrix(131)
|
+-------------------------------------------------------- Afronatrix anosc(132)
|
+-------------------------------------------------------- Amphiesma stolat(133)
|
+-------------------------------------------------------- Thamnophis marci(134)
|
+-------------------------------------------------------- Xenochrophis pis(135)
|
+-------------------------------------------------------- Lampropeltis get(136)
|
\-------------------------------------------------------- Coluber constric(137)
```
