# Supplementary material for: Integrated Analyses Resolve Conflicts over Squamate Reptile Phylogeny and Reveal Unexpected Placements for Fossil Taxa
Source: PLoS One. 2015 Mar 24;10(3):e0118199. doi: 10.1371/journal.pone.0118199 (PMC4372529; doi:10.1371/journal.pone.0118199)
Supplement: S67 Fig — (PDF) [file pone.0118199.s069.pdf]

```
/-------------------------------------------------------- Sphenodon puncta(1)
|
+-------------------------------------------------------- Kallimodon pulch(2)
|
+-------------------------------------------------------- Gephyrosaurus br(3)
|
+-------------------------------------------------------- Huehuecuetzpalli(4)
|
+-------------------------------------------------------- Ctenomastax parv(5)
|
+-------------------------------------------------------- Priscagama gobie(6)
|
+-------------------------------------------------------- Mimeosaurus cras(7)
|
+-------------------------------------------------------- Phrynosomimus as(8)
|
|                           /---------------------------- Leiolepis bellia(9)
+------------61-------------+
|                           \---------------------------- Uromastyx aegypt(10)
|
|                           /---------------------------- Brookesia brygoo(11)
+------------86-------------+
|                           \---------------------------- Chamaeleo(12)
|
+-------------------------------------------------------- Physignathus coc(13)
|
+-------------------------------------------------------- Agama agama(14)
|
+-------------------------------------------------------- Calotes emma(15)
|
+-------------------------------------------------------- Pogona vitticeps(16)
|
+-------------------------------------------------------- Temujinia elliso(17)
|
+-------------------------------------------------------- Saichangurvel da(18)
|
```

```
+-------------------------------------------------------- Isodontosaurus g(19)
|
+-------------------------------------------------------- Zapsosaurus scel(20)
|
+-------------------------------------------------------- Polrussia mongol(21)
|
+-------------------------------------------------------- Basiliscus basil(22)
|
+-------------------------------------------------------- Corytophanes cri(23)
|
+-------------------------------------------------------- Polychrus marmor(24)
|
+-------------------------------------------------------- Anolis carolinen(25)
|
+-------------------------------------------------------- Leiosaurus catam(26)
|
+-------------------------------------------------------- Pristidactylus t(27)
|
+-------------------------------------------------------- Urostrophus vaut(28)
|
+-------------------------------------------------------- Aciprion formosu(29)
|
+-------------------------------------------------------- Crotaphytus coll(30)
|
+-------------------------------------------------------- Gambelia wislize(31)
|
+-------------------------------------------------------- Enyalioides lati(32)
|
+-------------------------------------------------------- Morunasaurus ann(33)
|
+-------------------------------------------------------- Brachylophus fas(34)
|
+-------------------------------------------------------- Armandisaurus ex(35)
|
+-------------------------------------------------------- Dipsosaurus dors(36)
|
```

```
+-------------------------------------------------------- Sauromalus ater(37)
|
+-------------------------------------------------------- Liolaemus bellii(38)
|
+-------------------------------------------------------- Phymaturus pallu(39)
|
+-------------------------------------------------------- Chalarodon madag(40)
|
+-------------------------------------------------------- Oplurus cyclurus(41)
|
+-------------------------------------------------------- Petrosaurus mear(42)
|
+-------------------------------------------------------- Uta stansburiana(43)
|
+-------------------------------------------------------- Sceloporus varia(44)
|
+-------------------------------------------------------- Phrynosoma platy(45)
|
+-------------------------------------------------------- Uma scoparia(46)
|
+-------------------------------------------------------- Leiocephalus bar(47)
|
+-------------------------------------------------------- Plica plica(48)
|
+-------------------------------------------------------- Stenocercus guen(49)
|
+-------------------------------------------------------- Uranoscodon supe(50)
|
+-------------------------------------------------------- Tchingisaurus mu(51)
|
+-------------------------------------------------------- Gobinatus arenos(52)
|
+-------------------------------------------------------- Adamisaurus magn(53)
|
+-------------------------------------------------------- Gilmoreteius(54)
|
+-------------------------------------------------------- Polyglyphanodon (55)
```

```
|
+-------------------------------------------------------- Sineoamphisbaena(56)
|
+-------------------------------------------------------- Adriosaurus sues(57)
|
+-------------------------------------------------------- Pontosaurus(58)
|
+-------------------------------------------------------- Aigialosaurus da(59)
|
|                           /---------------------------- Clidastes(60)
|                           |
|                           +---------------------------- Platecarpus(61)
+------------60-------------+
|                           +---------------------------- Plotosaurus(62)
|                           |
|                           \---------------------------- Tylosaurus(63)
|
+-------------------------------------------------------- Eichstaettisauru(64)
|
+-------------------------------------------------------- AMNH FR 21444(65)
|
|                           /---------------------------- Delma borea(66)
+------------55-------------+
|                           \---------------------------- Lialis burtonis(67)
|
+-------------------------------------------------------- Strophurus cilia(68)
|
+-------------------------------------------------------- Rhacodactylus au(69)
|
+-------------------------------------------------------- Saltuarius cornu(70)
|
+-------------------------------------------------------- Aeluroscalobates(71)
|
+-------------------------------------------------------- Coleonyx variega(72)
|
+-------------------------------------------------------- Eublepharis macu(73)
```

```
|
+-------------------------------------------------------- Teratoscincus(74)
|
+-------------------------------------------------------- Gonatodes albogu(75)
|
+-------------------------------------------------------- Phelsuma lineata(76)
|
+-------------------------------------------------------- Gekko gecko(77)
|
+-------------------------------------------------------- Lacerta viridis(78)
|
+-------------------------------------------------------- Takydromus ocell(79)
|
+-------------------------------------------------------- Colobosaura mode(80)
|
+-------------------------------------------------------- Pholidobolus(81)
|
+-------------------------------------------------------- Callopistes macu(82)
|
+-------------------------------------------------------- Tupinambis tegui(83)
|
|                           /---------------------------- Aspidoscelis tig(84)
+------------62-------------+
|                           \---------------------------- Teius teyou(85)
|
+-------------------------------------------------------- Paramacellodus(86)
|
+-------------------------------------------------------- Parmeosaurus scu(87)
|
+-------------------------------------------------------- Tepexisaurus tep(88)
|
+-------------------------------------------------------- Cricosaura typic(89)
|
+-------------------------------------------------------- Lepidophyma flav(90)
|
+-------------------------------------------------------- Palaeoxantusia s(91)
|
```

```
+-------------------------------------------------------- Xantusia vigilis(92)
|
+-------------------------------------------------------- Platysaurus(93)
|
+-------------------------------------------------------- Cordylus mossamb(94)
|
+-------------------------------------------------------- Zonosaurus ornat(95)
|
+-------------------------------------------------------- Cordylosaurus su(96)
|
+-------------------------------------------------------- Myrmecodaptria m(97)
|
+-------------------------------------------------------- Carusia intermed(98)
|
+-------------------------------------------------------- Globaura venusta(99)
|
+-------------------------------------------------------- Hymenosaurus cla(100)
|
+-------------------------------------------------------- Eoxanta lacertif(101)
|
+-------------------------------------------------------- Plestiodon fasci(102)
|
+-------------------------------------------------------- Scincus(103)
|
+-------------------------------------------------------- Brachymeles grac(104)
|
+-------------------------------------------------------- Acontias(105)
|
+-------------------------------------------------------- Amphiglossus spl(106)
|
+-------------------------------------------------------- Feylinia polylep(107)
|
+-------------------------------------------------------- Trachylepis quin(108)
|
+-------------------------------------------------------- Sphenomorphus so(109)
|
```

```
+-------------------------------------------------------- Eugongylus rufes(110)
|
+-------------------------------------------------------- Tiliqua scincoid(111)
|
+-------------------------------------------------------- Shinisaurus croc(112)
|
+-------------------------------------------------------- Xenosaurus platy(113)
|
+-------------------------------------------------------- Xenosaurus grand(114)
|
+-------------------------------------------------------- Pseudopus apodus(115)
|
+-------------------------------------------------------- Peltosaurus gran(116)
|
+-------------------------------------------------------- Helodermoides tu(117)
|
+-------------------------------------------------------- Anniella pulchra(118)
|
+-------------------------------------------------------- Celestus enneagr(119)
|
+-------------------------------------------------------- Elgaria multicar(120)
|
+-------------------------------------------------------- Gobiderma pulchr(121)
|
+-------------------------------------------------------- Estesia mongolie(122)
|
+-------------------------------------------------------- Aiolosaurus orie(123)
|
|                           /---------------------------- Heloderma horrid(124)
+------------74-------------+
|                           \---------------------------- Heloderma suspec(125)
|
+-------------------------------------------------------- Lanthanotus born(126)
|
+-------------------------------------------------------- Saniwa(127)
|
+-------------------------------------------------------- Varanus salvator(128)
```

```
|
+-------------------------------------------------------- Varanus acanthur(129)
|
+-------------------------------------------------------- Varanus exanthem(130)
|
+-------------------------------------------------------- Anelytropsis pap(131)
|
+-------------------------------------------------------- Dibamus novaegui(132)
|
+-------------------------------------------------------- Spathorhynchus f(133)
|
+-------------------------------------------------------- Dyticonastis ren(134)
|
+-------------------------------------------------------- Rhineura florida(135)
|
|                           /---------------------------- Bipes biporus(136)
+------------71-------------+
|                           \---------------------------- Bipes canalicula(137)
|
+-------------------------------------------------------- Trogonophis wieg(138)
|
+-------------------------------------------------------- Diplometopon zar(139)
|
+-------------------------------------------------------- Geocalamus acutu(140)
|
+-------------------------------------------------------- Amphisbaena fuli(141)
|
+-------------------------------------------------------- Najash rionegrin(142)
|
+-------------------------------------------------------- Dinilysia patago(143)
|
+-------------------------------------------------------- Leptotyphlops(144)
|
+-------------------------------------------------------- Typhlops jamaice(145)
|
+-------------------------------------------------------- Liotyphlops albi(146)
```

```
|
+-------------------------------------------------------- Typhlophis squam(147)
|
+-------------------------------------------------------- Anomochilus leon(148)
|
+-------------------------------------------------------- Anilius scytale(149)
|
+-------------------------------------------------------- Cylindrophis ruf(150)
|
+-------------------------------------------------------- Uropeltis melano(151)
|
+-------------------------------------------------------- Xenopeltis unico(152)
|
+-------------------------------------------------------- Loxocemus bicolo(153)
|
+-------------------------------------------------------- Xenophidion acan(154)
|
+-------------------------------------------------------- Casarea dussumie(155)
|
+-------------------------------------------------------- Haasiophis terra(156)
|
+-------------------------------------------------------- Eupodophis desco(157)
|
+-------------------------------------------------------- Pachyrhachis pro(158)
|
+-------------------------------------------------------- Exiliboa placata(159)
|
+-------------------------------------------------------- Ungaliophis cont(160)
|
+-------------------------------------------------------- Eryx colubrinus(161)
|
+-------------------------------------------------------- Calabaria reinha(162)
|
+-------------------------------------------------------- Lichanura trivir(163)
|
+-------------------------------------------------------- Epicrates striat(164)
|
```

```
+-------------------------------------------------------- Boa constrictor(165)
|
+-------------------------------------------------------- Aspidites melano(166)
|
+-------------------------------------------------------- Python molurus(167)
|
+-------------------------------------------------------- Trachyboa boulen(168)
|
+-------------------------------------------------------- Tropidophis haet(169)
|
+-------------------------------------------------------- Xenodermus javan(170)
|
+-------------------------------------------------------- Acrochordus gran(171)
|
+-------------------------------------------------------- Pareas hamptoni(172)
|
+-------------------------------------------------------- Lycophidion cape(173)
|
+-------------------------------------------------------- Aparallactus wer(174)
|
+-------------------------------------------------------- Atractaspis irre(175)
|
+-------------------------------------------------------- Causus(176)
|
+-------------------------------------------------------- Azemiops feae(177)
|
+-------------------------------------------------------- Daboia russelli(178)
|
+-------------------------------------------------------- Agkistrodon cont(179)
|
+-------------------------------------------------------- Bothrops asper(180)
|
+-------------------------------------------------------- Lachesis muta(181)
|
+-------------------------------------------------------- Naja(182)
|
```

```
+-------------------------------------------------------- Notechis scutatu(183)
|
+-------------------------------------------------------- Laticauda colubr(184)
|
+-------------------------------------------------------- Micrurus fulvius(185)
|
+-------------------------------------------------------- Natrix natrix(186)
|
+-------------------------------------------------------- Afronatrix anosc(187)
|
+-------------------------------------------------------- Amphiesma stolat(188)
|
+-------------------------------------------------------- Thamnophis marci(189)
|
+-------------------------------------------------------- Xenochrophis pis(190)
|
+-------------------------------------------------------- Lampropeltis get(191)
|
\-------------------------------------------------------- Coluber constric(192)
```
