# Supplementary material for: Integrated Analyses Resolve Conflicts over Squamate Reptile Phylogeny and Reveal Unexpected Placements for Fossil Taxa
Source: PLoS One. 2015 Mar 24;10(3):e0118199. doi: 10.1371/journal.pone.0118199 (PMC4372529; doi:10.1371/journal.pone.0118199)
Supplement: S68 Fig — (PDF) [file pone.0118199.s070.pdf]

```
/-------------------------------------------------------- Sphenodon puncta(1)
|
|                                    /------------------- Leiolepis bellia(2)
+-----------------53-----------------+
|                                    \------------------- Uromastyx aegypt(3)
|
|                                    /------------------- Brookesia brygoo(4)
|                  /-------80--------+
|                  |                 \------------------- Chamaeleo(5)
|                  |
|                  |                 /------------------- Delma borea(37)
|                  +-------51--------+
|                  |                 \------------------- Lialis burtonis(38)
|                  |
|                  +------------------------------------- Acontias(67)
|                  |
|                  +------------------------------------- Feylinia polylep(69)
|                  |
|                  +------------------------------------- Anniella pulchra(78)
|                  |
|                  +------------------------------------- Anelytropsis pap(87)
|                  |
|                  +------------------------------------- Dibamus novaegui(88)
|                  |
|                  +------------------------------------- Rhineura florida(89)
|                  |
|                  |                 /------------------- Bipes biporus(90)
|                  +-------76--------+
|                  |                 \------------------- Bipes canalicula(91)
|                  |
|                  +------------------------------------- Trogonophis wieg(92)
|                  |
|                  +------------------------------------- Diplometopon zar(93)
|                  |
|                  +------------------------------------- Geocalamus acutu(94)
|                  |
```

```
|                  +------------------------------------- Amphisbaena fuli(95)
|                  |
|                  +------------------------------------- Leptotyphlops(96)
|                  |
|                  +------------------------------------- Typhlops jamaice(97)
|                  |
|                  +------------------------------------- Liotyphlops albi(98)
|                  |
|                  +------------------------------------- Anilius scytale(99)
|                  |
|                  +------------------------------------- Cylindrophis ruf(100)
|                  |
|                  +------------------------------------- Uropeltis melano(101)
|                  |
|                  +------------------------------------- Xenopeltis unico(102)
|                  |
|                  +------------------------------------- Loxocemus bicolo(103)
|                  |
|                  +------------------------------------- Exiliboa placata(104)
|                  |
|                  +------------------------------------- Ungaliophis cont(105)
|                  |
|                  +------------------------------------- Eryx colubrinus(106)
|                  |
|                  +------------------------------------- Calabaria reinha(107)
|                  |
|                  +------------------------------------- Lichanura trivir(108)
|                  |
+--------52--------+------------------------------------- Epicrates striat(109)
|                  |
|                  +------------------------------------- Boa constrictor(110)
|                  |
|                  +------------------------------------- Aspidites melano(111)
|                  |
|                  +------------------------------------- Python molurus(112)
|                  |
```

```
|                  +------------------------------------ Trachyboa boulen(113)
|                  |
|                  +------------------------------------ Tropidophis haet(114)
|                  |
|                  +------------------------------------ Xenodermus javan(115)
|                  |
|                  +------------------------------------ Acrochordus gran(116)
|                  |
|                  +------------------------------------ Pareas hamptoni(117)
|                  |
|                  +------------------------------------ Lycophidion cape(118)
|                  |
|                  +------------------------------------ Aparallactus wer(119)
|                  |
|                  +------------------------------------ Atractaspis irre(120)
|                  |
|                  +------------------------------------ Causus(121)
|                  |
|                  +------------------------------------ Azemiops feae(122)
|                  |
|                  +------------------------------------ Daboia russelli(123)
|                  |
|                  +------------------------------------ Agkistrodon cont(124)
|                  |
|                  +------------------------------------ Bothrops asper(125)
|                  |
|                  +------------------------------------ Lachesis muta(126)
|                  |
|                  +------------------------------------ Naja(127)
|                  |
|                  +------------------------------------ Notechis scutatu(128)
|                  |
|                  +------------------------------------ Laticauda colubr(129)
|                  |
|                  +------------------------------------ Micrurus fulvius(130)
|                  |
|                  +------------------------------------ Natrix natrix(131)
```

```
|                  |
|                  +------------------------------------ Afronatrix anosc(132)
|                  |
|                  +------------------------------------ Amphiesma stolat(133)
|                  |
|                  +------------------------------------ Thamnophis marci(134)
|                  |
|                  +------------------------------------ Xenochrophis pis(135)
|                  |
|                  +------------------------------------ Lampropeltis get(136)
|                  |
|                  \------------------------------------ Coluber constric(137)
|
+------------------------------------------------------- Physignathus coc(6)
|
+------------------------------------------------------- Agama agama(7)
|
+------------------------------------------------------- Calotes emma(8)
|
+------------------------------------------------------- Pogona vitticeps(9)
|
+------------------------------------------------------- Basiliscus basil(10)
|
+------------------------------------------------------- Corytophanes cri(11)
|
+------------------------------------------------------- Polychrus marmor(12)
|
+------------------------------------------------------- Anolis carolinen(13)
|
+------------------------------------------------------- Leiosaurus catam(14)
|
+------------------------------------------------------- Pristidactylus t(15)
|
+------------------------------------------------------- Urostrophus vaut(16)
|
+------------------------------------------------------- Crotaphytus coll(17)
```

```
|
+-------------------------------------------------------- Gambelia wislize(18)
|
+-------------------------------------------------------- Enyalioides lati(19)
|
+-------------------------------------------------------- Morunasaurus ann(20)
|
+-------------------------------------------------------- Brachylophus fas(21)
|
+-------------------------------------------------------- Dipsosaurus dors(22)
|
+-------------------------------------------------------- Sauromalus ater(23)
|
+-------------------------------------------------------- Liolaemus bellii(24)
|
+-------------------------------------------------------- Phymaturus pallu(25)
|
+-------------------------------------------------------- Chalarodon madag(26)
|
+-------------------------------------------------------- Oplurus cyclurus(27)
|
+-------------------------------------------------------- Petrosaurus mear(28)
|
+-------------------------------------------------------- Uta stansburiana(29)
|
+-------------------------------------------------------- Sceloporus varia(30)
|
+-------------------------------------------------------- Phrynosoma platy(31)
|
+-------------------------------------------------------- Uma scoparia(32)
|
+-------------------------------------------------------- Leiocephalus bar(33)
|
+-------------------------------------------------------- Plica plica(34)
|
+-------------------------------------------------------- Stenocercus guen(35)
|
```

```
+-------------------------------------------------------- Uranoscodon supe(36)
|
+-------------------------------------------------------- Strophurus cilia(39)
|
+-------------------------------------------------------- Rhacodactylus au(40)
|
|                                    /------------------- Saltuarius cornu(41)
+-----------------59-----------------+
|                                    \------------------- Phelsuma lineata(47)
|
+-------------------------------------------------------- Aeluroscalobates(42)
|
+-------------------------------------------------------- Coleonyx variega(43)
|
+-------------------------------------------------------- Eublepharis macu(44)
|
+-------------------------------------------------------- Teratoscincus(45)
|
+-------------------------------------------------------- Gonatodes albogu(46)
|
+-------------------------------------------------------- Gekko gecko(48)
|
+-------------------------------------------------------- Lacerta viridis(49)
|
+-------------------------------------------------------- Takydromus ocell(50)
|
+-------------------------------------------------------- Colobosaura mode(51)
|
+-------------------------------------------------------- Pholidobolus(52)
|
+-------------------------------------------------------- Callopistes macu(53)
|
+-------------------------------------------------------- Tupinambis tegui(54)
|
|                                    /------------------- Aspidoscelis tig(55)
+-----------------71-----------------+
```

```
|                                    \------------------- Teius teyou(56)
|
+-------------------------------------------------------- Cricosaura typic(57)
|
+-------------------------------------------------------- Lepidophyma flav(58)
|
+-------------------------------------------------------- Xantusia vigilis(59)
|
+-------------------------------------------------------- Platysaurus(60)
|
+-------------------------------------------------------- Cordylus mossamb(61)
|
+-------------------------------------------------------- Zonosaurus ornat(62)
|
+-------------------------------------------------------- Cordylosaurus su(63)
|
+-------------------------------------------------------- Plestiodon fasci(64)
|
+-------------------------------------------------------- Scincus(65)
|
+-------------------------------------------------------- Brachymeles grac(66)
|
+-------------------------------------------------------- Amphiglossus spl(68)
|
+-------------------------------------------------------- Trachylepis quin(70)
|
+-------------------------------------------------------- Sphenomorphus so(71)
|
+-------------------------------------------------------- Eugongylus rufes(72)
|
+-------------------------------------------------------- Tiliqua scincoid(73)
|
+-------------------------------------------------------- Shinisaurus croc(74)
|
+-------------------------------------------------------- Xenosaurus platy(75)
|
+-------------------------------------------------------- Xenosaurus grand(76)
```

```
|
+-------------------------------------------------------- Pseudopus apodus(77)
|
+-------------------------------------------------------- Celestus enneagr(79)
|
+-------------------------------------------------------- Elgaria multicar(80)
|
|                                    /------------------- Heloderma horrid(81)
+-----------------83-----------------+
|                                    \------------------- Heloderma suspec(82)
|
+-------------------------------------------------------- Lanthanotus born(83)
|
|                                    /------------------- Varanus salvator(84)
|                  /-------55--------+
|                  |                 \------------------- Varanus exanthem(86)
\--------63--------+
                   \------------------------------------- Varanus acanthur(85)
```
