# Supplementary material for: Integrated Analyses Resolve Conflicts over Squamate Reptile Phylogeny and Reveal Unexpected Placements for Fossil Taxa
Source: PLoS One. 2015 Mar 24;10(3):e0118199. doi: 10.1371/journal.pone.0118199 (PMC4372529; doi:10.1371/journal.pone.0118199)
Supplement: S70 Fig — (PDF) [file pone.0118199.s072.pdf]

```
/------------------------------------------------------- Sphenodon puncta(1)
|
+------------------------------------------------------- Leiolepis bellia(2)
|
+------------------------------------------------------- Uromastyx aegypt(3)
|
|                                              /-------- Brookesia brygoo(4)
|                                              |
|                                              +-------- Leiosaurus catam(14)
+----------------------52----------------------+
|                                              +-------- Pristidactylus t(15)
|                                              |
|                                              \-------- Urostrophus vaut(16)
|
+------------------------------------------------------- Chamaeleo(5)
|
+------------------------------------------------------- Physignathus coc(6)
|
+------------------------------------------------------- Agama agama(7)
|
+------------------------------------------------------- Calotes emma(8)
|
+------------------------------------------------------- Pogona vitticeps(9)
|
+------------------------------------------------------- Basiliscus basil(10)
|
+------------------------------------------------------- Corytophanes cri(11)
|
+------------------------------------------------------- Polychrus marmor(12)
|
+------------------------------------------------------- Anolis carolinen(13)
|
+------------------------------------------------------- Crotaphytus coll(17)
|
+------------------------------------------------------- Gambelia wislize(18)
|
+------------------------------------------------------- Enyalioides lati(19)
|
+------------------------------------------------------- Morunasaurus ann(20)
|
+------------------------------------------------------- Brachylophus fas(21)
|
+------------------------------------------------------- Dipsosaurus dors(22)
|
+------------------------------------------------------- Sauromalus ater(23)
|
+------------------------------------------------------- Liolaemus bellii(24)
|
+------------------------------------------------------- Phymaturus pallu(25)
|
+------------------------------------------------------- Chalarodon madag(26)
|
+------------------------------------------------------- Oplurus cyclurus(27)
|
+------------------------------------------------------- Petrosaurus mear(28)
|
+------------------------------------------------------- Uta stansburiana(29)
|
+------------------------------------------------------- Sceloporus varia(30)
|
+------------------------------------------------------- Phrynosoma platy(31)
|
+------------------------------------------------------- Uma scoparia(32)
|
+------------------------------------------------------- Leiocephalus bar(33)
|
+------------------------------------------------------- Plica plica(34)
|
+------------------------------------------------------- Stenocercus guen(35)
|
+------------------------------------------------------- Uranoscodon supe(36)
|
```

```
+-------------------------------------------------------- Delma borea(37)
|
+-------------------------------------------------------- Lialis burtonis(38)
|
+-------------------------------------------------------- Strophurus cilia(39)
|
+-------------------------------------------------------- Rhacodactylus au(40)
|
+-------------------------------------------------------- Saltuarius cornu(41)
|
+-------------------------------------------------------- Aeluroscalobates(42)
|
+-------------------------------------------------------- Coleonyx variega(43)
|
+-------------------------------------------------------- Eublepharis macu(44)
|
+-------------------------------------------------------- Teratoscincus(45)
|
+-------------------------------------------------------- Gonatodes albogu(46)
|
+-------------------------------------------------------- Phelsuma lineata(47)
|
+-------------------------------------------------------- Gekko gecko(48)
|
|                                              /--------- Lacerta viridis(49)
+----------------------70----------------------+
|                                              \--------- Takydromus ocell(50)
|
+-------------------------------------------------------- Colobosaura mode(51)
|
+-------------------------------------------------------- Pholidobolus(52)
|
+-------------------------------------------------------- Callopistes macu(53)
|
+-------------------------------------------------------- Tupinambis tegui(54)
|
|                                              /--------- Aspidoscelis tig(55)
+----------------------51----------------------+
|                                              \--------- Teius teyou(56)
|
+-------------------------------------------------------- Cricosaura typic(57)
|
+-------------------------------------------------------- Lepidophyma flav(58)
|
+-------------------------------------------------------- Xantusia vigilis(59)
|
|                                              /--------- Platysaurus(60)
|        /-----------------54------------------+
|        |                                     \--------- Cordylus mossamb(61)
|        |
|        |         /------------------------------------- Zonosaurus ornat(62)
|        |         |
+---67---+         +------------------------------------- Cordylosaurus su(63)
|        |         |
|        |         |        /---------------------------- Plestiodon fasci(64)
|        |         |        |
|        |         |        +---------------------------- Scincus(65)
|        \---76----+        |
|                  |        +---------------------------- Brachymeles grac(66)
|                  |        |
|                  |        |                  /--------- Acontias(67)
|                  |        |        /---66----+
|                  |        |        |         \--------- Feylinia polylep(69)
|                  \---95---+        |
|                           +---52---+------------------- Trachylepis quin(70)
|                           |        |
|                           |        \------------------- Tiliqua scincoid(73)
|                           |
|                           +---------------------------- Amphiglossus spl(68)
|                           |
|                           +---------------------------- Sphenomorphus so(71)
|                           |
|                           \---------------------------- Eugongylus rufes(72)
|
```

```
|
|                                              /--------- Shinisaurus croc(74)
|                                    /---58----+
|                                    |         \--------- Xenosaurus grand(76)
+-----------------75-----------------+
|                                    \------------------- Xenosaurus platy(75)
|
|                                    /------------------- Pseudopus apodus(77)
|                                    |
|                                    |         /--------- Anniella pulchra(78)
+-----------------74-----------------+---69----+
|                                    |         \--------- Celestus enneagr(79)
|                                    |
|                                    \------------------- Elgaria multicar(80)
|
|                                              /--------- Heloderma horrid(81)
+----------------------52----------------------+
|                                              \--------- Heloderma suspec(82)
|
+-------------------------------------------------------- Lanthanotus born(83)
|
+-------------------------------------------------------- Varanus salvator(84)
|
+-------------------------------------------------------- Varanus acanthur(85)
|
+-------------------------------------------------------- Varanus exanthem(86)
|
|                                              /--------- Anelytropsis pap(87)
+----------------------66----------------------+
|                                              \--------- Dibamus novaegui(88)
|
+-------------------------------------------------------- Rhineura florida(89)
|
+-------------------------------------------------------- Bipes biporus(90)
|
+-------------------------------------------------------- Bipes canalicula(91)
|
+-------------------------------------------------------- Trogonophis wieg(92)
|
+-------------------------------------------------------- Diplometopon zar(93)
|
+-------------------------------------------------------- Geocalamus acutu(94)
|
+-------------------------------------------------------- Amphisbaena fuli(95)
|
+-------------------------------------------------------- Leptotyphlops(96)
|
+-------------------------------------------------------- Typhlops jamaice(97)
|
+-------------------------------------------------------- Liotyphlops albi(98)
|
+-------------------------------------------------------- Anilius scytale(99)
|
+-------------------------------------------------------- Cylindrophis ruf(100)
|
+-------------------------------------------------------- Uropeltis melano(101)
|
+-------------------------------------------------------- Xenopeltis unico(102)
|
|                                              /--------- Loxocemus bicolo(103)
|                                              |
|                                              +--------- Calabaria reinha(107)
+----------------------64----------------------+
|                                              +--------- Aspidites melano(111)
|                                              |
|                                              \--------- Python molurus(112)
|
+-------------------------------------------------------- Exiliboa placata(104)
|
+-------------------------------------------------------- Ungaliophis cont(105)
|
+-------------------------------------------------------- Eryx colubrinus(106)
|
```

```
+-------------------------------------------------------- Lichanura trivir(108)
|
+-------------------------------------------------------- Epicrates striat(109)
|
+-------------------------------------------------------- Boa constrictor(110)
|
+-------------------------------------------------------- Trachyboa boulen(113)
|
+-------------------------------------------------------- Tropidophis haet(114)
|
+-------------------------------------------------------- Xenodermus javan(115)
|
+-------------------------------------------------------- Acrochordus gran(116)
|
+-------------------------------------------------------- Pareas hamptoni(117)
|
+-------------------------------------------------------- Lycophidion cape(118)
|
+-------------------------------------------------------- Aparallactus wer(119)
|
+-------------------------------------------------------- Atractaspis irre(120)
|
+-------------------------------------------------------- Causus(121)
|
+-------------------------------------------------------- Azemiops feae(122)
|
+-------------------------------------------------------- Daboia russelli(123)
|
+-------------------------------------------------------- Agkistrodon cont(124)
|
+-------------------------------------------------------- Bothrops asper(125)
|
+-------------------------------------------------------- Lachesis muta(126)
|
+-------------------------------------------------------- Naja(127)
|
+-------------------------------------------------------- Notechis scutatu(128)
|
+-------------------------------------------------------- Laticauda colubr(129)
|
+-------------------------------------------------------- Micrurus fulvius(130)
|
+-------------------------------------------------------- Natrix natrix(131)
|
+-------------------------------------------------------- Afronatrix anosc(132)
|
+-------------------------------------------------------- Amphiesma stolat(133)
|
+-------------------------------------------------------- Thamnophis marci(134)
|
+-------------------------------------------------------- Xenochrophis pis(135)
|
+-------------------------------------------------------- Lampropeltis get(136)
|
\-------------------------------------------------------- Coluber constric(137)
```
