# Supplementary material for: Integrated Analyses Resolve Conflicts over Squamate Reptile Phylogeny and Reveal Unexpected Placements for Fossil Taxa
Source: PLoS One. 2015 Mar 24;10(3):e0118199. doi: 10.1371/journal.pone.0118199 (PMC4372529; doi:10.1371/journal.pone.0118199)
Supplement: S71 Fig — (PDF) [file pone.0118199.s073.pdf]

```
/-------------------------------------------------------- Sphenodon puncta(1)
|
+-------------------------------------------------------- Agama agama(2)
|
+-------------------------------------------------------- Calotes emma(3)
|
|                                    /------------------- Physignathus coc(4)
+-----------------54-----------------+
|                                    \------------------- Enyalioides lati(12)
|
+-------------------------------------------------------- Pogona vitticeps(5)
|
+-------------------------------------------------------- Brookesia brygoo(6)
|
+-------------------------------------------------------- Chamaeleo calypt(7)
|
+-------------------------------------------------------- Basiliscus basil(8)
|
+-------------------------------------------------------- Corytophanes cri(9)
|
+-------------------------------------------------------- Crotaphytus coll(10)
|
+-------------------------------------------------------- Gambelia wislize(11)
|
+-------------------------------------------------------- Morunasaurus ann(13)
|
+-------------------------------------------------------- Brachylophus fas(14)
|
+-------------------------------------------------------- Dipsosaurus dors(15)
|
+-------------------------------------------------------- Sauromalus ater(16)
|
+-------------------------------------------------------- Leiolepis rubrit(17)
|
+-------------------------------------------------------- Uromastyx hardwi(18)
|
+-------------------------------------------------------- Leiosaurus catam(19)
|
+-------------------------------------------------------- Pristidactylus t(20)
|
+-------------------------------------------------------- Urostrophus vaut(21)
|
+-------------------------------------------------------- Liolaemus elonga(22)
|
+-------------------------------------------------------- Phymaturus pallu(23)
|
+-------------------------------------------------------- Chalarodon madag(24)
|
+-------------------------------------------------------- Oplurus cyclurus(25)
|
+-------------------------------------------------------- Petrosaurus mear(26)
|
+-------------------------------------------------------- Phrynosoma platy(27)
|
+-------------------------------------------------------- Sceloporus varia(28)
|
+-------------------------------------------------------- Uma scoparia(29)
|
+-------------------------------------------------------- Uta stansburiana(30)
|
+-------------------------------------------------------- Anolis carolinen(31)
|
+-------------------------------------------------------- Polychrus marmor(32)
|
+-------------------------------------------------------- Leiocephalus bar(33)
|
+-------------------------------------------------------- Plica plica(34)
|
+-------------------------------------------------------- Stenocercus guen(35)
|
+-------------------------------------------------------- Uranoscodon supe(36)
```

```
|
|                                    /------------------- Ophisaurus apodu(37)
+-----------------62-----------------+
|                                    \------------------- Elgaria multicar(40)
|
+-------------------------------------------------------- Anniella pulchra(38)
|
+-------------------------------------------------------- Celestus enneagr(39)
|
+-------------------------------------------------------- Cordylus mossamb(41)
|
+-------------------------------------------------------- Platysaurus impe(42)
|
|                                    /------------------- Anelytropsis pap(43)
+-----------------86-----------------+
|                                    \------------------- Dibamus novaegui(44)
|
+-------------------------------------------------------- Aeluroscalobates(45)
|
+-------------------------------------------------------- Coleonyx variega(46)
|
+-------------------------------------------------------- Eublepharis macu(47)
|
+-------------------------------------------------------- Teratoscincus pr(48)
|
+-------------------------------------------------------- Diplodactylus ci(49)
|
+-------------------------------------------------------- Phyllurus cornut(50)
|
+-------------------------------------------------------- Rhacodactylus au(51)
|
+-------------------------------------------------------- Gekko gecko(52)
|
+-------------------------------------------------------- Phelsuma lineata(53)
|
+-------------------------------------------------------- Gonatodes albogu(54)
|
|                                    /------------------- Cordylosaurus su(55)
+-----------------56-----------------+
|                                    \------------------- Zonosaurus ornat(56)
|
+-------------------------------------------------------- Alopoglossus ang(57)
|
+-------------------------------------------------------- Colobosaura mode(58)
|
+-------------------------------------------------------- Pholidobolus mon(59)
|
|                                    /------------------- Heloderma horrid(60)
+-----------------77-----------------+
|                                    \------------------- Heloderma suspec(61)
|
+-------------------------------------------------------- Lacerta viridis(62)
|
+-------------------------------------------------------- Takydromus ocell(63)
|
+-------------------------------------------------------- Delma borea(64)
|
+-------------------------------------------------------- Lialis burtonis(65)
|
+-------------------------------------------------------- Acontias perciva(66)
|
+-------------------------------------------------------- Amphiglossus spl(67)
|
+-------------------------------------------------------- Brachymeles grac(68)
|
+-------------------------------------------------------- Eugongylus rufes(69)
|
+-------------------------------------------------------- Eumeces fasciatu(70)
|
+-------------------------------------------------------- Feylinia polylep(71)
|
+-------------------------------------------------------- Mabuya quinqueta(72)
|
```

```
+-------------------------------------------------------- Scincus scincus(73)
|
+-------------------------------------------------------- Sphenomorphus so(74)
|
+-------------------------------------------------------- Tiliqua scincoid(75)
|
+-------------------------------------------------------- Shinisaurus croc(76)
|
|                                    /------------------- Aspidoscelis tig(77)
+-----------------78-----------------+
|                                    \------------------- Teius teyou(79)
|
+-------------------------------------------------------- Callopistes macu(78)
|
+-------------------------------------------------------- Tupinambis tegui(80)
|
+-------------------------------------------------------- Lanthanotus born(81)
|
+-------------------------------------------------------- Varanus acanthur(82)
|
+-------------------------------------------------------- Varanus exanthem(83)
|
+-------------------------------------------------------- Varanus salvator(84)
|
+-------------------------------------------------------- Cricosaura typic(85)
|
+-------------------------------------------------------- Lepidophyma flav(86)
|
+-------------------------------------------------------- Xantusia vigilis(87)
|
+-------------------------------------------------------- Xenosaurus grand(88)
|
+-------------------------------------------------------- Xenosaurus platy(89)
|
|                                    /------------------- Amphisbaena fuli(90)
|                                    |
|                  /-------51--------+------------------- Bipes biporus(92)
|                  |                 |
|                  |                 \------------------- Bipes canalicula(93)
|                  |
|                  +------------------------------------- Geocalamus acutu(91)
+--------79--------+
|                  |                 /------------------- Rhineura florida(94)
|                  +-------58--------+
|                  |                 \------------------- Diplometopon zar(95)
|                  |
|                  \------------------------------------- Trogonophis wieg(96)
|
+-------------------------------------------------------- Acrochordus gran(97)
|
+-------------------------------------------------------- Anilius scytale(98)
|
|                  /------------------------------------- Liotyphlops albi(99)
|                  |
+--------86--------+                 /------------------- Leptotyphlops du(129)
|                  \-------73--------+
|                                    \------------------- Typhlops jamaice(137)
|
+-------------------------------------------------------- Aparallactus wer(100)
|
+-------------------------------------------------------- Atractaspis irre(101)
|
+-------------------------------------------------------- Boa constrictor(102)
|
+-------------------------------------------------------- Calabaria reinha(103)
|
+-------------------------------------------------------- Epicrates striat(104)
|
+-------------------------------------------------------- Eryx colubrinus(105)
|
+-------------------------------------------------------- Lichanura trivir(106)
|
+-------------------------------------------------------- Afronatrix anosc(107)
```

```
|
+-------------------------------------------------------- Amphiesma stolat(108)
|
+-------------------------------------------------------- Coluber constric(109)
|
+-------------------------------------------------------- Diadophis puncta(110)
|
+-------------------------------------------------------- Heterodon platir(111)
|
+-------------------------------------------------------- Homalopsis bucca(112)
|
+-------------------------------------------------------- Imantodes cencho(113)
|
+-------------------------------------------------------- Lampropeltis get(114)
|
+-------------------------------------------------------- Lamprophis fulig(115)
|
+-------------------------------------------------------- Lycophidion cape(116)
|
+-------------------------------------------------------- Natrix natrix(117)
|
+-------------------------------------------------------- Pareas hamptoni(118)
|
+-------------------------------------------------------- Sonora semiannul(119)
|
+-------------------------------------------------------- Thamnophis marci(120)
|
+-------------------------------------------------------- Trimorphodon bis(121)
|
+-------------------------------------------------------- Xenochrophis pis(122)
|
+-------------------------------------------------------- Xenodermus javan(123)
|
+-------------------------------------------------------- Cylindrophis ruf(124)
|
+-------------------------------------------------------- Laticauda colubr(125)
|
+-------------------------------------------------------- Micrurus fulvius(126)
|
+-------------------------------------------------------- Naja naja(127)
|
+-------------------------------------------------------- Notechis scutatu(128)
|
+-------------------------------------------------------- Loxocemus bicolo(130)
|
+-------------------------------------------------------- Aspidites melano(131)
|
+-------------------------------------------------------- Python molurus(132)
|
+-------------------------------------------------------- Exiliboa placata(133)
|
+-------------------------------------------------------- Trachyboa boulen(134)
|
+-------------------------------------------------------- Tropidophis haet(135)
|
+-------------------------------------------------------- Ungaliophis cont(136)
|
+-------------------------------------------------------- Uropeltis melano(138)
|
+-------------------------------------------------------- Agkistrodon cont(139)
|
+-------------------------------------------------------- Azemiops feae(140)
|
+-------------------------------------------------------- Bothrops asper(141)
|
+-------------------------------------------------------- Causus rhombeatu(142)
|
+-------------------------------------------------------- Daboia russelli(143)
|
+-------------------------------------------------------- Lachesis muta(144)
|
\-------------------------------------------------------- Xenopeltis unico(145)
```
