# Supplementary material for: Integrated Analyses Resolve Conflicts over Squamate Reptile Phylogeny and Reveal Unexpected Placements for Fossil Taxa
Source: PLoS One. 2015 Mar 24;10(3):e0118199. doi: 10.1371/journal.pone.0118199 (PMC4372529; doi:10.1371/journal.pone.0118199)
Supplement: S1 Table — GenBank numbers for the two new loci (c-Mos and ND2) added to the molecular data matrix of Wiens et al. (10) for this study. For species denoted with “*” an exact match to those species used in the original matrix were not available, and related species (same genus or family) were used instead. The species used in the original matrix are in parentheses. (DOC) [file pone.0118199.s074.doc]

**S1 Table.** **GenBank numbers for the two new loci**. GenBank numbers for the two new loci (c-*Mos* and *ND2*) added to the molecular data matrix of Wiens et al. [10] for this study. For species denoted with “*” an exact match to those species used in the original matrix were not available, and related species (same genus or family) were used instead. The species used in the original matrix are in parentheses.

| Species | c-Mos | **ND2** |
| --- | --- | --- |
| *Homo sapiens* | NM_005372 | JX401415 |
| *Mus musculus* | NM_020021 | 167716836 |
| *Alligator mississippiensis* | JF315210 | 5835540 |
| *Chelydra serpentina* | FJ230855 | 197935706 |
| *Crocodylus porosus* | JF315245 | 108802842 |
| *Dromaius novaehollandiae* | U88427 | 14141877 |
| *Gallus gallus* | M19412 | 71658078 |
| *Podocnemis expansa* | AF109209 | --- |
| *Chelus fimbriata ** (*Podocnemis expansa*) | --- | 345894243 |
| *Sphenodon punctatus* | AF039483 | AY662533 |
| *Tachyglossus aculeatus* | AY168511 | 18077882 |
| *Achalinus meiguensis ** (*Xenodermus javanicus*) | --- | 212725550 |
| *Acontias meleagris* | DQ249058 | AY662553 |
| *Acrochordus granulatus* | HM234057 | 74310558 |
| *Aeluroscalobates felinus* | --- | JX041301 |
| *Afronatrix anoscopus* | AF471123 | --- |
| *Agama agama* | AF137530 | AF128504 |
| *Agkistrodon piscivorus* | AF471096 | 156878356 |
| *Alopoglossus angulatum* | AF420847 | --- |
| *Amphiesma stolata* | AF471097 | 380862825 |
| *Amphiglossus intermedius ** (*A. splendidus*) | AY391181 | --- |
| *Amphisbaena fuliginosa* | FJ441744 | 229335960 |
| *Anelytropsis papilosus* | HQ876394 | 321228121 |
| *Anilius scytale* | AF544722 | 301353287 |
| *Anniella pulchra* | AY487350 | AF085606 |
| *Anolis carolinensis* | --- | 319428597 |
| *Anolis nitens ** (*A. carolinensis*) | GQ853276 | --- |
| *Aparallactus werneri* | AF471116 | --- |
| *Aspidites melanocephalus* | DQ465557 | --- |
| *Aspidoscelis tigris* | AF039481 | U71332 |
| *Atractaspis boulengeri ** (*A. irregularis*) | FJ404237 | --- |
| *Azemiops feae* | AF544695 | --- |
| *Basiliscus plumifrons ** (*B. basiliscus*) | AY987986 | U82680 |
| *Bipes biporus* | AF039482 | U71335 |
| *Bipes canaliculatus* | FJ518700 | 52221108 |
| *Boa constrictor* | HQ399543 | 118196950 |
| *Bothrops atrox ** (*B. asper*) | DQ469788 | --- |
| *Brachylophus fasciatus* | AY987993 | AF528721 |
| *Brachymeles bicolor ** (*B. gracilis*) | AY818797 | --- |
| *Brachymeles bonitae ** (*B. gracilis*) | --- | 374092334 |
| *Brookesia brygooi* | FJ984305 | AF448774 |
| *Calabaria reinhardtii* | AF544682 | --- |
| *Callisaurus draconoides ** (*Uma scoparia*) | AF315401 | --- |
| *Calotes emma* | --- | DQ289460 |
| *Calotes versicolor ** (*C. emma*) | AF137525 | --- |
| *Calumma fallax ** (*Chamaeleo calyptratus*) | JN030468 | --- |
| *Casarea dussumieri* | AF544731 | --- |
| *Causus defilippi* | --- | 264681156 |
| *Causus resimus* | AF544696 | --- |
| *Celestus enneagrammus* | --- | AF085607 |
| *Chalarodon madagascariensis* | AY987987 | AF528722 |
| *Chamaeleo chamaeleon ** (*C. calyptratus*) | --- | 225676458 |
| *Chelosania brunnea* | DQ340664 | --- |
| *Chlamydosaurus kingii* | DQ340665 | --- |
| *Coleonyx variegatus* | HQ876391 | 121582399 |
| *Colobosaura modesta* | EU116677 | --- |
| *Coluber constrictor* | AY486937 | 46095054 |
| *Cordylosaurus subtesselatus* | AY217849 | 308095686 |
| *Cordylus mossambicus* | DQ100147 | 308095768 |
| *Corytophanes cristatus* | AF315390 | AF528717 |
| *Cricosaura typica* | EU116689 | --- |
| *Crotaphytus collaris* | AY987985 | U82681 |
| *Ctenophorus isolepis* | DQ340671 | --- |
| *Cylindrophis rufus* | AF544698 | 74310586 |
| *Daboia russelli* | AF471156 | 209886939 |
| *Delma borea* | AY134547 | AY134583 |
| *Diadophis punctatus* | AF544705 | --- |
| *Dibamus novaeguineae* | EF450999 | 321228131 |
| *Diplolaemus darwinii ** (*Pristidactylus torquatus*) | AY987988 | --- |
| *Diplometopon zarudnyi* | AY444023 | 52220996 |
| *Dipsosaurus dorsalis* | EU116680 | AF049857 |
| *Elgaria multicarinata* | AF039479 | AF085620 |
| *Enhydris plumbea ** (*Homalopsis buccata*) | --- | 164420865 |
| *Enyalioides laticeps* | --- | AF528719 |
| *Epicrates striatus* | AY099966 | --- |
| *Eryx colubrinus* | DQ465568 | --- |
| *Eublepharis macularius* | HQ876392 | 164457666 |
| *Eugongylus rufescens* | HQ655205 | 110628014 |
| *Eunectes notaeus * (Epicrates striatus)* | --- | 118196936 |
| *Exiliboa placata* | AY099973 | --- |
| *Feylinia polylepis* | AY818772 | AY662556 |
| *Gambelia wislizenii* | AY217842 | U82682 |
| *Gekko gecko* | EU366455 | AF114249 |
| *Geocalamus acutus* | AY444017 | 52221024 |
| *Gonatodes albogularis* | GU139825 | JX041354 |
| *Goniurosaurs kuroiwae* | HQ876393 | --- |
| *Heloderma horridum* | JN090140 | AF407539 |
| *Heloderma suspectum* | AY662566 | 121622372 |
| *Heterodon simus ** (*H. platyrhinos*) | AF471142 | 118738097 |
| *Homalopsis buccata* | AF544701 | --- |
| *Hydrosaurus amboinensis ** (*H*. sp.) | --- | 296940265 |
| *Hypsilurus boydi* | DQ340682 | --- |
| *Imantodes cenchoa* | GQ457865 | --- |
| *Lacerta viridis* | DQ097129 | 114052884 |
| *Lampropeltis calligaster ** (*L. getula*) | --- | 118738099 |
| *Lampropeltis getula* | FJ627796 | --- |
| *Lamprophis fuliginosus* | FJ404270 | --- |
| *Lanthanotus borneensis* | AY662564 | AY662537 |
| *Laticauda colubrina* | EU366446 | --- |
| *Leiocephalus barahonensis* | DQ119594 | EF591773 |
| *Leiolepis belliana* | FJ984253 | U82689 |
| *Leiosaurus catamarcensis* | --- | AF528731 |
| *Lepidophyma flavimaculatum* | DQ249070 | 121582620 |
| *Leposoma parietale ** (*Colobosaura modesta*) | --- | AY662543 |
| *Lialis burtonis* | EF534906 | JX024354 |
| *Lichanura trivirgata* | AF544687 | GQ200595 |
| *Liolaemus bellii* | --- | AF099223 |
| *Liolaemus elongatus* | AY367881 | --- |
| *Liotyphlops albirostris* | AF544727 | --- |
| *Loxocemus bicolor* | AY444035 | --- |
| *Lycophidion capense* | DQ486168 | --- |
| *Macroprotodon cucullatus ** (*Lycophidion capense*) | --- | 46095100 |
| *Microlophus grayii ** (*Uranoscodon superciliosus*) | FJ458604 | --- |
| *Micrurus fulvius* | EF137421 | 264681184 |
| *Moloch horridus* | DQ340697 | --- |
| *Morunasaurus annularis* | --- | AF528720 |
| *Naja kaouthia* | AY058938 | --- |
| *Naja naja ** (*N. kaouthia*) | --- | 164420837 |
| *Natrix natrix* | AF544697 | 58013186 |
| *Notechis ater ** (*N. scutatus*) | EU546944 | --- |
| *Ophisaurus apodus* | --- | AF085623 |
| *Ophisaurus gracilis ** (*O. ventralis*) | AY444030 | --- |
| *Oplurus cuvieri ** (*O. cyclurus*) | --- | U82685 |
| *Oplurus cyclurus* | EU099668 | --- |
| *Pareas hamptoni* | JF827703 | --- |
| *Petrosaurus mearnsi* | --- | GQ502768 |
| *Phelsuma lineata* | FJ830106 | EU423283 |
| *Pholidobolus macbrydei* | AY507896 | --- |
| *Phrynocephalus mystaceus* | AF137527 | --- |
| *Phrynosoma cornutum ** (*P. platyrhinos*) | AY987989 | --- |
| *Phrynosoma platyrhinos* | --- | AY297488 |
| *Phyllurus platurus **  (*S. cornutus*) | AY172941 | --- |
| *Phymaturus palluma* | --- | AF099216 |
| *Phymaturus patagonicus ** (*P. palluma*) | JF272880 | --- |
| *Physignathus cocincinus* | DQ340688 | U82690 |
| *Physignathus_lesueurii* | DQ340689 | --- |
| *Platysaurus capensis **  (*P. pungweensis*) | --- | U71329 |
| *Platysaurus pungweensis* | EU116686 | --- |
| *Plestiodon fasciatus* | HQ655218 | --- |
| *Plestiodon inexpectatus ** (*P. fasciatus*) | --- | AY662550 |
| *Plestiodon skiltonianus* | AF315396 | --- |
| *Pogona vitticeps* | DQ340691 | AY133026 |
| *Polychrus marmoratus* | AY987983 | AF528738 |
| *Pristidactylus scapulatus ** (*P. torquatus*) | --- | AF528732 |
| *Python molurus* | GQ225667 | 339906613 |
| *Rankinia adelaidensis* | DQ340692 | --- |
| *Rena humilis* | AY099979 | 49616774 |
| *Rhacodactylus auriculatus* | AY172944 | JX024427 |
| *Rhineura floridana* | AY444021 | 52221038 |
| *Rhinophis philippinus ** (*Uropeltus melanogaster*) | --- | GQ200594 |
| *Saltuarius swaini ** (*S. cornutus*) | --- | JX024356 |
| *Sauromalus obesus* | AF315400 | U82687 |
| *Sceloporus grammicus ** (*S. variabilis*) | AF039478 | --- |
| *Sceloporus variabilis* | --- | AY297507 |
| *Scincus scincus* | AY217873 | --- |
| *Shinisaurus crocodilurus* | AY099976 | AF085604 |
| *Sonora semiannulata* | AF471164 | --- |
| *Sphenomorphus solomonis* | HQ655224 | 326653266 |
| *Stenocercus guentheri* | --- | DQ080223 |
| *Strophurus ciliaris* | --- | AY368996 |
| *Strophurus intermedius ** (*S. ciliaris*) | AF039469 | --- |
| *Takydromus amurensis ** (*T. ocellatus*) | EF632287 | --- |
| *Takydromus tachydromoides ** (*T. ocellatus*) | --- | 121582435 |
| *Teratoscincus przewalskii ** (*T. scincus*) | --- | U71326 |
| *Teratoscincus scincus* | EF534927 | AF114251 |
| *Thamnophis marcianus* | --- | 18448357 |
| *Thamnophis sirtalis ** (*T. marcianus*) | DQ902094 | --- |
| *Tiliqua rugosa ** (*T. scincoides*) | --- | JX041462 |
| *Tiliqua scincoides* | AF039462 | --- |
| *Trachyboa gularis ** (*T. boulengeri*) | AY491999 | --- |
| *Trachylepis perrotetii ** (*T. quinquetaeniata*) | --- | 326653274 |
| *Trachylepis quinquetaeniata* | DQ238987 | --- |
| *Trimorphodon biscutatus* | GQ927319 | --- |
| *Trogonophis wiegmanni* | AY444025 | AY662542 |
| *Tropidophis haetianus* | AY099962 | 227018580 |
| *Tropiduris plica* | EF615737 | AF528748 |
| *Tupinambis quadrilineatus ** (*T. teguixin*) | AY217889 | --- |
| *Typhlops jamaicensis* | AF544733 | --- |
| *Typhlops reticulatus ** (*T. jamaicensis*) | --- | 190349594 |
| *Uma scoparia* | --- | EU543781 |
| *Ungaliophis continentalis* | AF544724 | --- |
| *Uranoscodon superciliosus* | --- | AF528749 |
| *Uromastyx aegyptus* | AF137531 | 67003447 |
| *Uropeltis phillipsi ** (*U. melanogaster*) | AF471100 | --- |
| *Urostrophus vautieri* | --- | AF528734 |
| *Uta stansburiana* | --- | EU543775 |
| *Varanus acanthurus* | --- | AF407488 |
| *Varanus exanthematicus* | JN090143 | AF407496 |
| *Varanus salvator* | AF435017 | AF407526 |
| *Xantusia vigilis* | EU116816 | U71328 |
| *Xenochrophis piscator* | GQ225669 | --- |
| *Xenochrophis punctulatus ** (*X. piscator*) | --- | 46095120 |
| *Xenodermus javanicus* | AF544711 | --- |
| *Xenopeltis unicolor* | AF544689 | 74310600 |
| *Xenosaurus grandis* | AY662567 | U71333 |
| *Zonosaurus ornatus* | EU571699 | --- |
| *Zonosaurus* sp. * *(Z. ornatus)* | --- | AY662560 |
