# Supplementary material for: Integrated Analyses Resolve Conflicts over Squamate Reptile Phylogeny and Reveal Unexpected Placements for Fossil Taxa
Source: PLoS One. 2015 Mar 24;10(3):e0118199. doi: 10.1371/journal.pone.0118199 (PMC4372529; doi:10.1371/journal.pone.0118199)
Supplement: S4 Table — Summary of RogueNaRok results, illustrating the impacts of excluding specific sets of taxa, where the maximum number of taxa in a drop set is 3 (but fewer taxa may be selected). “Raw improvement” is the overall fraction of bootstrap improvement. RIBC is the relative bipartition information criterion. (DOC) [file pone.0118199.s077.doc]

**S4 Table.** **Summary of RogueNaRok results, with maximum of 3 species in drop set.** Summary of RogueNaRok results, illustrating the impacts of excluding specific sets of taxa, where the maximum number of taxa in a drop set is 3 (but fewer taxa may be selected). “Raw improvement” is the overall fraction of bootstrap improvement. RIBC is the relative bipartition information criterion.

| Taxon | Raw improvement | RBIC |
| --- | --- | --- |
| *Sineoamphisbaena; Huehuecuetzpalli* | 2.61 | 0.791095 |
| *Eupodophis; Pachyrhachis; Haasiophis* | 1.43 | 0.807595 |
| AMNH FR 21444*; Eichstaettisaurus* | 0.97 | 0.800464 |
| *Aciprion* | 0.92 | 0.795655 |
| *Gobiderma* | 0.41 | 0.809652 |
| *Adriosaurus* | 0.19 | 0.810597 |
| *Celestus* | 0.08 | 0.811012 |
| *Tylosaurus* | 0.07 | 0.811343 |
| *Sauromalus* | 0.01 | 0.811393 |
