# Supplementary material for: Integrated Analyses Resolve Conflicts over Squamate Reptile Phylogeny and Reveal Unexpected Placements for Fossil Taxa
Source: PLoS One. 2015 Mar 24;10(3):e0118199. doi: 10.1371/journal.pone.0118199 (PMC4372529; doi:10.1371/journal.pone.0118199)
Supplement: S5 Table — Summary of phylogenetic placement of Iguania in the 46 genes analyzed in this study, showing the clade or clades reconstructed as the sister group to Iguania, whether Toxicofera is supported or not, and whether monophyly of Iguania is supported. None of the trees show Iguania as sister to all other Squamata. (DOC) [file pone.0118199.s078.doc]

**55 Table.** **Summary of phylogenetic placement of Iguania across the 46 genes.** Summary of phylogenetic placement of Iguania in the 46 genes analyzed in this study, showing the clade or clades reconstructed as the sister group to Iguania, whether Toxicofera is supported or not, and whether monophyly of Iguania is supported. None of the trees show Iguania as sister to all other Squamata.

| Gene | Sister to Iguania | Supports Toxicofera | Supports Iguania |
| --- | --- | --- | --- |
| ADNP | Lacertoidea (Lacertidae, Amphisbaenia, Teiidae, Gymnophthalmidae) | no | no (polyphyletic) |
| AHR | Snakes, Anguimorpha | yes | yes |
| AKAP9 | Snakes | yes | yes |
| BACH1 | Snakes | yes | yes |
| BDNF | Anguimorpha | no | yes |
| BHLHB2 | Snakes | yes | yes |
| BMP2 | Snakes, Anguimorpha | yes | yes |
| CAND1 | Snakes | yes | yes |
| CARD4 | Anguimorpha | no | yes |
| CILP | Gekkota, Scincoidea, snakes, Teioidea (all but Anguimorpha) | no | yes |
| CXCR4 | Anguimorpha | no | yes |
| DLL1 | Snakes | yes | yes |
| ECEL1 | Snakes | yes | yes |
| ENC1 | Anguimorpha | yes | yes |
| FSHR | Snakes, teiids, gymnophthalmids | no | yes |
| FSTL5 | Snakes | yes | yes |
| GALR1 | Snakes | yes | no (paraphyletic) |
| GHSR | Anguimorpha (except Lanthanotus, Varanus) | yes | yes |
| GPR37 | Snakes, Anguimorpha, Lacertoidea | no | no (polyphyletic) |
| HLCS | Anguimorpha, Lacertoidea | no | yes |
| INHIBA | Snakes | yes | yes |
| LRRN1 | Snakes, Anguimorpha | yes | no (polyphyletic) |
| LZTSS1 | Snakes, Lacertoidea | no | yes |
| MKL1 | Snakes | yes | yes |
| MLL3 | Anguimorpha | yes | yes |
| MSH6 | Anguimorpha, snakes, Lacertoidea | no | yes |
| NGFB | Snakes, Anguimorpha | yes | yes |
| NKTR | Anguimorpha | yes | yes |
| NTF3 | Snakes | no | yes |
| PNN | Snakes, anguimorpha | yes | yes |
| PRLR | Snakes, anguimorpha | yes | yes |
| PTGER4 (rooting problematic) | Not clear | no | no |
| PTPN | Anguimorpha | yes | yes |
| R35 | Anguimorpha | yes | yes |
| RAG1 | Anguimorpha, snakes | yes | yes |
| SINCAIP | Lacertoidea, Anguimorpha, snakes | no | no (polyphyletic) |
| SLC8A1 | Anguimorpha, snakes | yes | yes |
| SLC8A3 | Anguimorpha | yes | yes |
| SLC30A1 | Amphisbaenia, Lacertidae | no | yes |
| TRAF6 | Anguimorpha | yes | yes |
| UBN1 | Anguimorpha | no | yes |
| VCPIP1 | Snakes, Anguimorpha | yes | yes |
| ZEB2 | Snakes, Anguimorpha | yes | yes |
| ZFP36L1 | Xantusiidae | no | yes |
| CMOS | Anguimorpha | yes | yes |
| ND2 | Not clear | no | yes |
