# Supplementary material for: Integrated Analyses Resolve Conflicts over Squamate Reptile Phylogeny and Reveal Unexpected Placements for Fossil Taxa
Source: PLoS One. 2015 Mar 24;10(3):e0118199. doi: 10.1371/journal.pone.0118199 (PMC4372529; doi:10.1371/journal.pone.0118199)
Supplement: S6 Table — Length and number of parsimony-informative characters for each of the 46 genes used in the analyses of squamate phylogeny. The first 44 genes are listed alphabetically, whereas the last 2 genes listed are new to this study (relative to ref. [10]). (DOC) [file pone.0118199.s079.doc]

**S6 Table.** **Length (in base pairs) and number of parsimony-informative characters for each of the 46 genes**. Length and number of parsimony-informative characters for each of the 46 genes used in the analyses of squamate phylogeny. The first 44 genes are listed alphabetically, whereas the last 2 genes listed are new to this study (relative to ref. [10]).

| Gene | Length | Parsimony-informative characters |
| --- | --- | --- |
| ADNP | 801 | 405 |
| AHR | 469 | 350 |
| AKAP9 | 1508 | 1161 |
| BACH1 | 1341 | 981 |
| BDNF | 691 | 264 |
| BHLHB2 | 773 | 457 |
| BMP2 | 648 | 345 |
| CAND1 | 759 | 296 |
| CARD4 | 954 | 677 |
| CILP | 921 | 559 |
| CXCR4 | 738 | 396 |
| DLL1 | 579 | 281 |
| ECEL1 | 579 | 356 |
| ENC1 | 888 | 350 |
| FSHR | 753 | 366 |
| FSTL5 | 622 | 304 |
| GALR1 | 423 | 210 |
| GHSR | 448 | 216 |
| GPR37 | 509 | 230 |
| HLCS | 594 | 460 |
| INHIBA | 744 | 385 |
| LRRN1 | 681 | 275 |
| LZTSS1 | 552 | 350 |
| MKL1 | 1053 | 680 |
| MLL3 | 960 | 677 |
| MSH6 | 690 | 470 |
| NGFB | 594 | 417 |
| NKTR | 1304 | 988 |
| NTF3 | 531 | 348 |
| PNN | 1197 | 725 |
| PRLR | 585 | 526 |
| PTGER4 | 471 | 220 |
| PTPN | 585 | 521 |
| R35 | 738 | 558 |
| RAG1 | 1091 | 555 |
| SINCAIP | 498 | 295 |
| SLC8A1 | 996 | 409 |
| SLC8A3 | 1107 | 517 |
| SLC30A1 | 555 | 349 |
| TRAF6 | 651 | 414 |
| UBN1 | 737 | 528 |
| VCPIP1 | 801 | 353 |
| ZEB2 | 885 | 371 |
| ZFP36L1 | 611 | 275 |
| CMOS | 890 | 544 |
| ND2 | 1065 | 919 |
