# Supplementary material for: Integrated Analyses Resolve Conflicts over Squamate Reptile Phylogeny and Reveal Unexpected Placements for Fossil Taxa
Source: PLoS One. 2015 Mar 24;10(3):e0118199. doi: 10.1371/journal.pone.0118199 (PMC4372529; doi:10.1371/journal.pone.0118199)
Supplement: S20 Fig — (PDF) [file pone.0118199.s022.pdf]

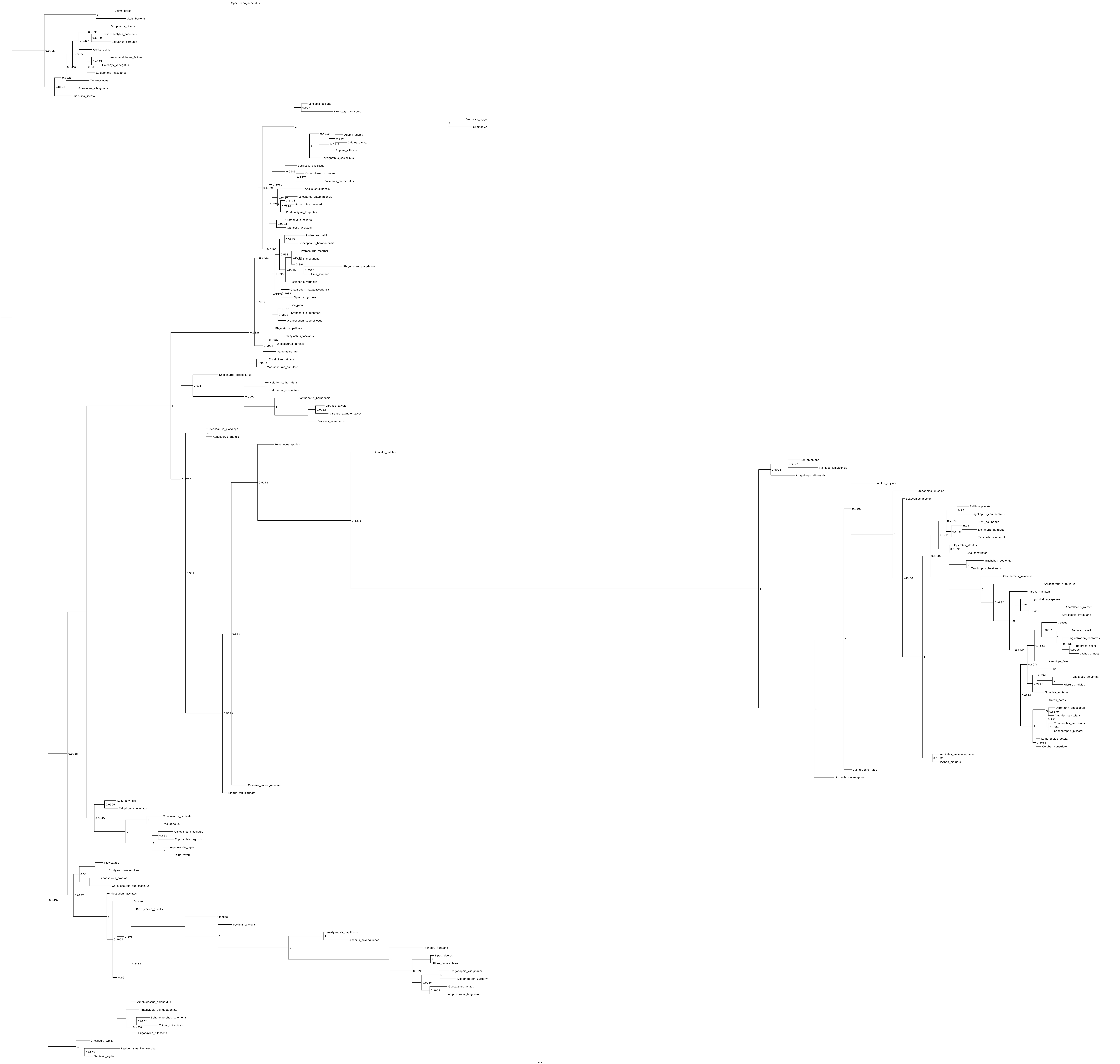
Sphenodon_punctatus
Delma_borea
Lialis_burtonis
Strophurus_ciliaris
Rhacodactylus_auriculatus
Saltuarius_cornutus
Gekko_gecko
Aeluroscalobates_felinus
Coleonyx_variegatus
Eublepharis_macularius
Teratoscincus
Gonatodes_albogularis
Phelsuma_lineata
Leiolepis_belliana
Uromastyx_aegyptus
Brookesia_brygooi
Chamaeleo
Agama_agama
Calotes_emma
Pogona_vitticeps
Physignathus_cocincinus
Basiliscus_basiliscus
Corytophanes_cristatus
Polychrus_marmoratus
Anolis_carolinensis
Leiosaurus_catamarcensis
Urostrophus_vautieri
Pristidactylus_torquatus
Crotaphytus_collaris
Gambelia_wislizenii
Liolaemus_bellii
Leiocephalus_barahonensis
Petrosaurus_mearnsi
Uta_stansburiana
Phrynosoma_platyrhinos
Uma_scoparia
Sceloporus_variabilis
Chalarodon_madagascariensis
Oplurus_cyclurus
Plica_plica
Stenocercus_guentheri
Uranoscodon_superciliosus
Phymaturus_palluma
Brachylophus_fasciatus
Dipsosaurus_dorsalis
Sauromalus_ater
Enyalioides_laticeps
Morunasaurus_annularis
Shinisaurus_crocodilurus
Heloderma_horridum
Heloderma_suspectum
Lanthanotus_borneensis
Varanus_salvator
Varanus_exanthematicus
Varanus_acanthurus
Xenosaurus_platyceps
Xenosaurus_grandis
Pseudopus_apodus
Anniella_pulchra
Leptotyphlops
Typhlops_jamaicensis
Liotyphlops_albirostris
Anilius_scytale
Xenopeltis_unicolor
Loxocemus_bicolor
Exiliboa_placata
Ungaliophis_continentalis
Eryx_colubrinus
Lichanura_trivirgata
Calabaria_reinhardtii
Epicrates_striatus
Boa_constrictor
Trachyboa_boulengeri
Tropidophis_haetianus
Xenodermus_javanicus
Acrochordus_granulatus
Pareas_hamptoni
Lycophidion_capense
Aparallactus_werneri
Atractaspis_irregularis
Causus
Daboia_russelli
Agkistrodon_contortrix
Bothrops_asper
Lachesis_muta
Azemiops_feae
Naja
Laticauda_colubrina
Micrurus_fulvius
Notechis_scutatus
Natrix_natrix
Afronatrix_anoscopus
Amphiesma_stolata
Thamnophis_marcianus
Xenochrophis_piscator
Lampropeltis_getula
Coluber_constrictor
Aspidites_melanocephalus
Python_molurus
Cylindrophis_rufus
Uropeltis_melanogaster
Celestus_enneagrammus
Elgaria_multicarinata
Lacerta_viridis
Takydromus_ocellatus
Colobosaura_modesta
Pholidobolus
Callopistes_maculatus
Tupinambis_teguixin
Aspidoscelis_tigris
Teius_teyou
Platysaurus
Cordylus_mossambicus
Zonosaurus_ornatus
Cordylosaurus_subtesselatus
Plestiodon_fasciatus
Scincus
Brachymeles_gracilis
Acontias
Feylinia_polylepis
Anelytropsis_papillosus
Dibamus_novaeguineae
Rhineura_floridana
Bipes_biporus
Bipes_canaliculatus
Trogonophis_wiegmanni
Diplometopon_zarudnyi
Geocalamus_acutus
Amphisbaena_fuliginosa
Amphiglossus_splendidus
Trachylepis_quinquetaeniata
Sphenomorphus_solomonis
Tiliqua_scincoides
Eugongylus_rufescens
Cricosaura_typica
Lepidophyma_flavimaculatu
Xantusia_vigilis
