# Supplementary material for: Integrated Analyses Resolve Conflicts over Squamate Reptile Phylogeny and Reveal Unexpected Placements for Fossil Taxa
Source: PLoS One. 2015 Mar 24;10(3):e0118199. doi: 10.1371/journal.pone.0118199 (PMC4372529; doi:10.1371/journal.pone.0118199)
Supplement: S32 Fig — Numbers at nodes indicate bootstrap support values. (PDF) [file pone.0118199.s034.pdf]

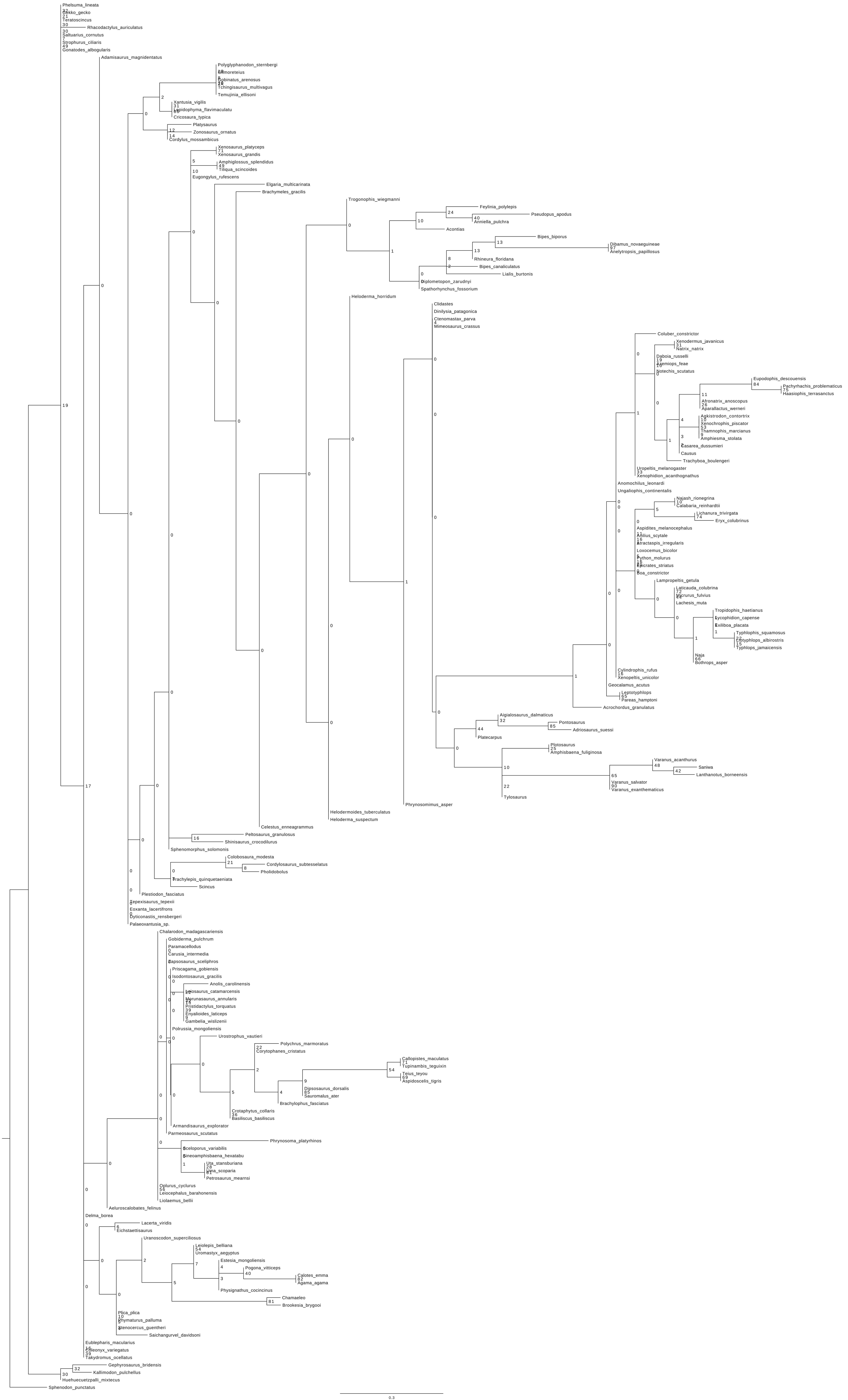
Phelsuma_lineata
Gekko_gecko
Teratoscincus
Rhacodactylus_auriculatus
Saltuarius_cornutus
Strophurus_ciliaris
Gonatodes_albogularis
Adamisaurus_magnidentatus
Polyglyphanodon_sternbergi
Gilmoreteius
Gobinatus_arenosus
Tchingisaurus_multivagus
Temujinia_ellisoni
Xantusia_vigilis
Lepidophyma_flavimaculatu
Cricosaura_typica
Platysaurus
Zonosaurus_ornatus
Cordylus_mossambicus
Xenosaurus_platyceps
Xenosaurus_grandis
Amphiglossus_splendidus
Tiliqua_scincoides
Eugongylus_rufescens
Elgaria_multicarinata
Brachymeles_gracilis
Trogonophis_wiegmanni
Feylinia_polylepis
Pseudopus_apodus
Anniella_pulchra
Acontias
Bipes_biporus
Dibamus_novaeguineae
Anelytropsis_papillosus
Rhineura_floridana
Bipes_canaliculatus
Lialis_burtonis
Diplometopon_zarudnyi
Spathorhynchus_fossorium
Heloderma_horridum
Clidastes
Dinilysia_patagonica
Ctenomastax_parva
Mimeosaurus_crassus
Coluber_constrictor
Xenodermus_javanicus
Natrix_natrix
Daboia_russelli
Azemiops_feae
Notechis_scutatus
Eupodophis_descouensis
Pachyrhachis_problematicus
Haasiophis_terrasanctus
Afronatrix_anoscopus
Aparallactus_werneri
Agkistrodon_contortrix
Xenochrophis_piscator
Thamnophis_marcianus
Amphiesma_stolata
Casarea_dussumieri
Causus
Trachyboa_boulengeri
Uropeltis_melanogaster
Xenophidion_acanthognathus
Anomochilus_leonardi
Ungaliophis_continentalis
Najash_rionegrina
Calabaria_reinhardtii
Lichanura_trivirgata
Eryx_colubrinus
Aspidites_melanocephalus
Anilius_scytale
Atractaspis_irregularis
Loxocemus_bicolor
Python_molurus
Epicrates_striatus
Boa_constrictor
Lampropeltis_getula
Laticauda_colubrina
Micrurus_fulvius
Lachesis_muta
Tropidophis_haetianus
Lycophidion_capense
Exiliboa_placata
Typhlophis_squamosus
Leptotyphlops_albirostris
Typhlops_jamaicensis
Naja
Bothrops_asper
Cylindrophis_rufus
Xenopeltis_unicolor
Geocalamus_acutus
Leptotyphlops
Pareas_hamptoni
Acrochordus_granulatus
Aigialosaurus_dalmaticus
Pontosaurus
Adriosaurus_suessi
Platecarpus
Plotosaurus
Amphisbaena_fuliginosa
Varanus_acanthurus
Saniwa
Lanthanotus_borneensis
Varanus_salvator
Varanus_exanthematicus
Tylosaurus
Phrynosomimus_asper
Helodermoides_tuberculatus
Heloderma_suspectum
Celestus_enneagrammus
Peltosaurus_granulosus
Shinisaurus_crocodilurus
Sphenomorphus_solomonis
Colobosaura_modesta
Cordylosaurus_subtesselatus
Pholidobolus
Trachylepis_quinquetaeniata
Scincus
Plestiodon_fasciatus
Tepexisaurus_tepexii
Eoxanta_lacertifrons
Dyticonastis_rensbergeri
Palaeoxantusia_sp.
Chalarodon_madagascariensis
Gobiderma_pulchrum
Paramacellodus
Carusia_intermedia
Dapsosaurus_sceliphros
Priscagama_gobiensis
Isodontosaurus_gracilis
Anolis_carolinensis
Leiosaurus_catamarcensis
Morunasaurus_annularis
Pristidactylus_torquatus
Enyalioides_laticeps
Gambelia_wislizenii
Polrussia_mongoliensis
Urostrophus_vautieri
Polychrus_marmoratus
Corytophanes_cristatus
Callopistes_maculatus
Tupinambis_teguixin
Teius_teyou
Aspidoscelis_tigris
Dipsosaurus_dorsalis
Sauromalus_ater
Brachylophus_fasciatus
Crotaphytus_collaris
Basiliscus_basiliscus
Armandisaurus_explorator
Parmeosaurus_scutatus
Phrynosoma_platyrhinos
Sceloporus_variabilis
Sineoamphisbaena_hexatabu
Uta_stansburiana
Uma_scoparia
Petrosaurus_mearnsi
Oplurus_cyclurus
Leiocephalus_barahonensis
Liolaemus_bellii
Aeluroscalobates_felinus
Delma_borea
Lacerta_viridis
Eichstaettisaurus
Uranoscodon_superciliosus
Leiolepis_belliana
Uromastyx_aegyptus
Estesia_mongoliensis
Pogona_vitticeps
Calotes_emma
Agama_agama
Physignathus_cocincinus
Chamaeleo
Brookesia_brygooi
Plica_plica
Phymaturus_palluma
Stenocercus_guentheri
Saichangurvel_davidsoni
Eublepharis_macularius
Coleonyx_variegatus
Takydromus_ocellatus
Gephyrosaurus_bridensis
Kallimodon_pulchellus
Huehuecuetzpalli_mixtecus
Sphenodon_punctatus
0.3
