# Supplementary material for: Integrated Analyses Resolve Conflicts over Squamate Reptile Phylogeny and Reveal Unexpected Placements for Fossil Taxa
Source: PLoS One. 2015 Mar 24;10(3):e0118199. doi: 10.1371/journal.pone.0118199 (PMC4372529; doi:10.1371/journal.pone.0118199)
Supplement: S37 Fig — Numbers at nodes indicate bootstrap support values. (PDF) [file pone.0118199.s039.pdf]

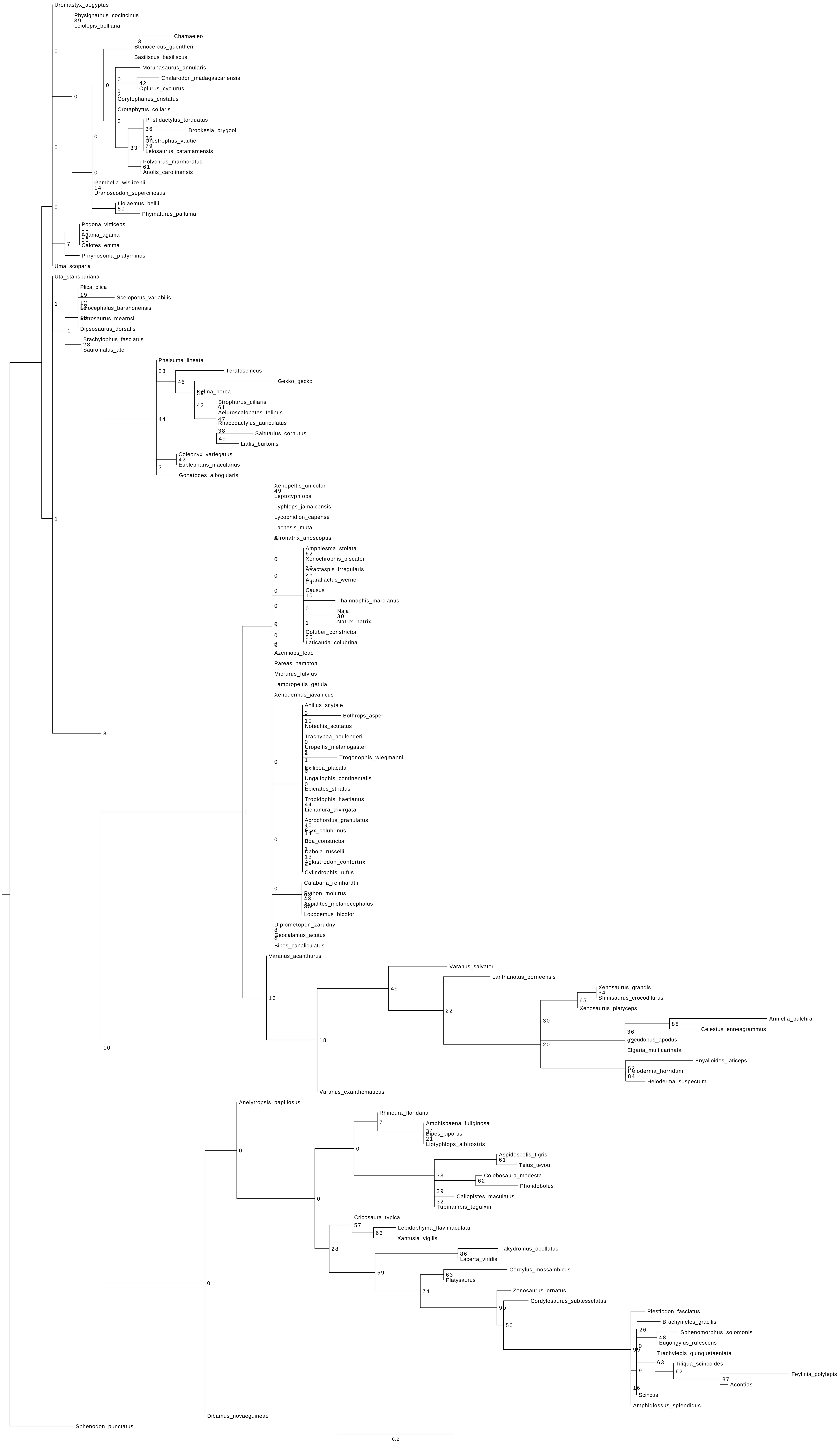
Uromastyx_aegyptus
Physignathus_cocincinus
39
Leiolepis_belliana
0
Chamaeleo
13
Stenocercus_guentheri
1
Basiliscus_basiliscus
0
Morunasaurus_annularis
0
Chalarodon_madagascariensis
42
Oplurus_cyclurus
1
2
Corytophanes_cristatus
Crotaphytus_collaris
3
Pristidactylus_torquatus
36
Brookesia_brygooi
36
Urostrophus_vautieri
79
Leiosaurus_catamarcensis
33
0
Polychrus_marmoratus
61
Anolis_carolinensis
0
0
Gambelia_wislizenii
14
Uranoscodon_superciliosus
Liolaemus_bellii
50
Phymaturus_palluma
0
Pogona_vitticeps
36
Agama_agama
30
Calotes_emma
7
Phrynosoma_platyrhinos
Uma_scoparia
Uta_stansburiana
Plica_plica
19
Sceloporus_variabilis
12
Leiocephalus_barahonensis
1
Petrosaurus_mearnsi
Dipsosaurus_dorsalis
1
Brachylophus_fasciatus
28
Sauromalus_ater
Phelsuma_lineata
23
Teratoscincus
45
Gekko_gecko
Delma_borea
39
42
Strophurus_ciliaris
61
Aeluroscalobates_felinus
47
Rhacodactylus_auriculatus
38
Saltuarius_cornutus
49
Lialis_burtonis
44
Coleonyx_variegatus
42
Eublepharis_macularius
3
Gonatodes_albogularis
1
Xenopeltis_unicolor
49
Leptotyphlops
Typhlops_jamaicensis
Lycophidion_capense
Lachesis_muta
Afronatrix_anoscopus
Amphiesma_stolata
62
Xenochrophis_piscator
Atractaspis_irregularis
26
54
Aparallactus_werneri
Causus
10
Thamnophis_marcianus
0
Naja
30
Natrix_natrix
1
Coluber_constrictor
55
Laticauda_colubrina
0
0
0
0
2
0
Azemiops_feae
Pareas_hamptoni
Micrurus_fulvius
Lampropeltis_getula
Xenodermus_javanicus
Anilius_scytale
3
Bothrops_asper
10
Notechis_scutatus
Trachyboa_boulengeri
0
Uropeltis_melanogaster
1
Trogonophis_wiegmanni
Exiliboa_placata
Ungaliophis_continentalis
0
Epicrates_striatus
Tropidophis_haetianus
44
Lichanura_trivirgata
Acrochordus_granulatus
10
Eryx_colubrinus
14
Boa_constrictor
1
Daboia_russelli
13
Agkistrodon_contortrix
4
Cylindrophis_rufus
0
0
Calabaria_reinhardtii
Python_molurus
43
Aspidites_melanocephalus
35
Loxocemus_bicolor
0
Diplometopon_zarudnyi
8
Geocalamus_acutus
Bipes_canaliculatus
1
8
Varanus_acanthurus
Varanus_salvator
Lanthanotus_borneensis
49
Xenosaurus_grandis
64
Shinisaurus_crocodilurus
65
Xenosaurus_platyceps
30
Anniella_pulchra
88
Celestus_enneagrammus
36
Pseudopus_apodus
Elgaria_multicarinata
20
22
Enyalioides_laticeps
52
Heloderma_horridum
84
Heloderma_suspectum
16
18
Varanus_exanthematicus
10
Anelytropsis_papillosus
Rhineura_floridana
7
Amphisbaena_fuliginosa
34
Bipes_biporus
21
Liotyphlops_albirostris
0
0
Aspidoscelis_tigris
61
Teius_teyou
33
Colobosaura_modesta
62
Pholidobolus
29
Callopistes_maculatus
32
Tupinambis_teguixin
0
Cricosaura_typica
57
Lepidophyma_flavimaculatu
63
Xantusia_vigilis
28
Takydromus_ocellatus
86
Lacerta_viridis
59
Cordylus_mossambicus
63
Platysaurus
74
Zonosaurus_ornatus
90
Cordylosaurus_subtesselatus
50
Plestiodon_fasciatus
Brachymeles_gracilis
26
Sphenomorphus_solomonis
48
Eugongylus_rufescens
0
99
Trachylepis_quinquetaeniata
63
Tiliqua_scincoides
62
Feylinia_polylepis
87
Acontias
9
16
Scincus
Amphiglossus_splendidus
0
Dibamus_novaeguineae
Sphenodon_punctatus
0.2
