# Supplementary material for: Integrated Analyses Resolve Conflicts over Squamate Reptile Phylogeny and Reveal Unexpected Placements for Fossil Taxa
Source: PLoS One. 2015 Mar 24;10(3):e0118199. doi: 10.1371/journal.pone.0118199 (PMC4372529; doi:10.1371/journal.pone.0118199)
Supplement: S43 Fig — Numbers at nodes indicate posterior probabilities. (PDF) [file pone.0118199.s045.pdf]

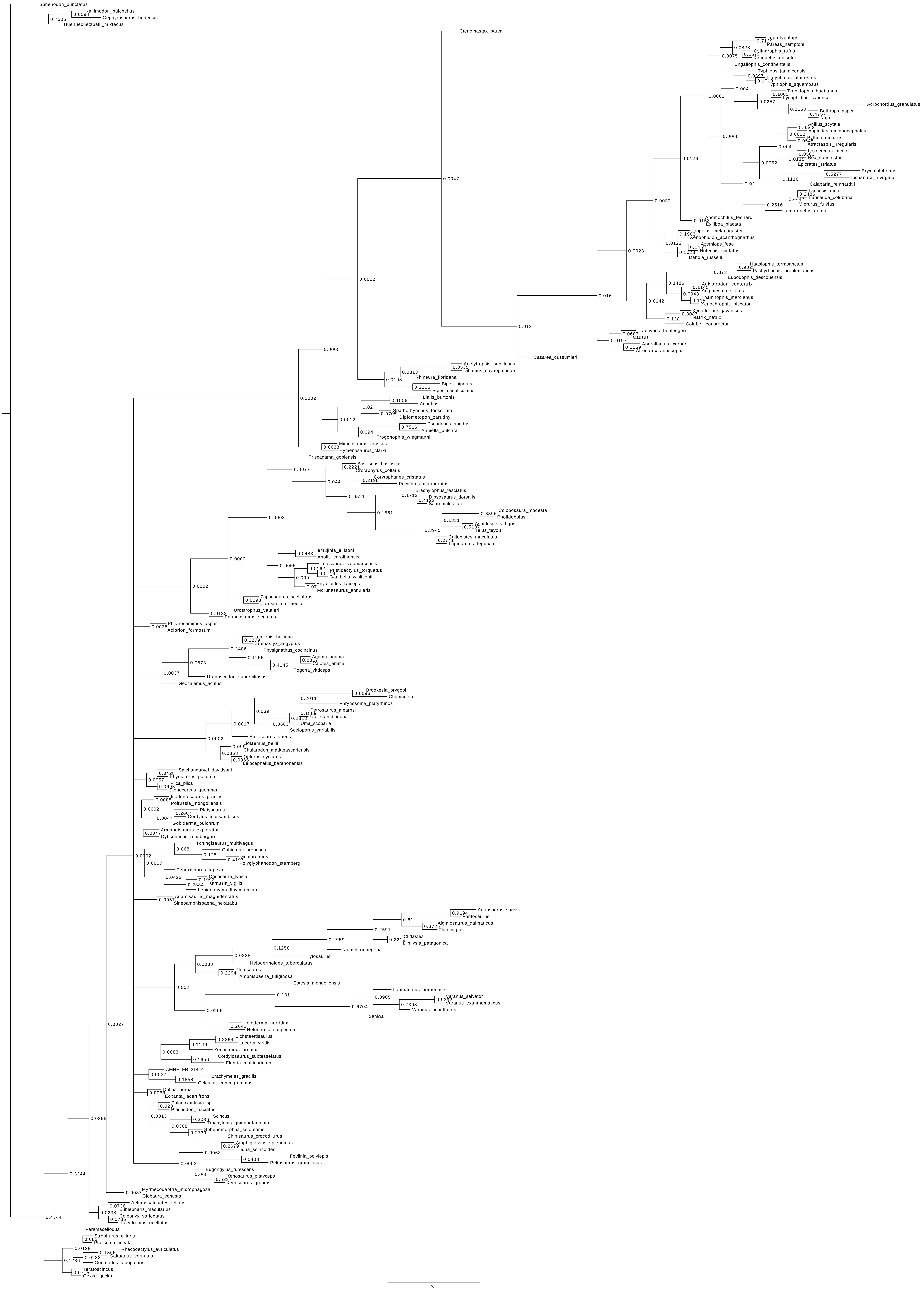

Sphenodon_punctatus
Kallimodon_pulchellus
Gephyrosaurus_bridensis
Huehuecuetzpalli_mixtecus
Ctenomastax_parva
Leptotyphlops
Pareas_hamptoni
Cylindrophis_rufus
Xenopeltis_unicolor
Ungaliophis_continentalis
Typhlops_jamaicensis
Liotyphlops_albirostris
Typhlophis_squamosus
Tropidophis_haetianus
Lycophidion_capense
Acrochordus_granulatus
Bothrops_asper
Naja
Anilius_scytale
Aspidites_melanocephalus
Python_molurus
Atractaspis_irregularis
Loxocemus_bicolor
Boa_constrictor
Epicrates_striatus
Eryx_colubrinus
Lichanura_trivirgata
Calabaria_reinhardtii
Lachesis_muta
Laticauda_colubrina
Micrurus_fulvius
Lampropeltis_getula
Anomochilus_leonardi
Exiliboa_placata
Uropeltis_melanogaster
Xenophidion_acanthognathus
Azemiops_feae
Notechis_scutatus
Daboia_russelli
Haasiophis_terrasanctus
Pachyrhachis_problematicus
Eupodophis_descouensis
Agkistrodon_contortrix
Amphiesma_stolata
Thamnophis_marcianus
Xenochrophis_piscator
Xenodermus_javanicus
Natrix_natrix
Coluber_constrictor
Trachyboa_boulengeri
Causus
Aparallactus_werneri
Afronatrix_anoscopus
Casarea_dussumieri
Anelytropsis_papillosus
Dibamus_novaeguineae
Rhineura_floridana
Bipes_biporus
Bipes_canaliculatus
Lialis_burtonis
Acontias
Spathorhynchus_fossorium
Diplometopon_zarudnyi
Pseudopus_apodus
Anniella_pulchra
Trogonophis_wiegmanni
Mimeosaurus_crassus
Hymenosaurus_clarki
Priscagama_gobiensis
Basiliscus_basiliscus
Crotaphytus_collaris
Corytophanes_cristatus
Polychrus_marmoratus
Brachylophus_fasciatus
Dipsosaurus_dorsalis
Sauromalus_ater
Colobosaura_modesta
Pholidobolus
Aspidoscelis_tigris
Teius_teyou
Callopistes_maculatus
Tupinambis_teguixin
Temujinia_ellisoni
Anolis_carolinensis
Leiosaurus_catamarcensis
Pristidactylus_torquatus
Gambelia_wislizenii
Enyalioides_laticeps
Morunasaurus_annularis
Zapsosaurus_sceliphros
Carusia_intermedia
Urostrophus_vautieri
Parmeosaurus_scutatus
Phrynosomimus_asper
Aciprion_formosum
Leiolepis_belliana
Uromastyx_aegyptus
Physignathus_cocincinus
Agama_agama
Calotes_emma
Pogona_vitticeps
Uranoscodon_superciliosus
Geocalamus_acutus
Brookesia_brygooi
Chamaeleo
Phrynosoma_platyrhinos
Petrosaurus_mearnsi
Uta_stansburiana
Uma_scoparia
Sceloporus_variabilis
Aiolosaurus_oriens
Liolaemus_bellii
Chalarodon_madagascariensis
Oplurus_cyclurus
Leiocephalus_barahonensis
Saichangurvel_davidsoni
Phymaturus_palluma
Plica_plica
Stenocercus_guentheri
Isodontosaurus_gracilis
Polrussia_mongoliensis
Platysaurus
Cordylus_mossambicus
Gobiderma_pulchrum
Armandisaurus_explorator
Dyticonastis_rensbergeri
Tchingisaurus_multivagus
Gobinatus_arenosus
Gilmoreteius
Polyglyphanodon_sternbergi
Tepexisaurus_tepexii
Cricosaura_typica
Xantusia_vigilis
Lepidophyma_flavimaculatu
Adamisaurus_magnidentatus
Sineoamphisbaena_hexatabu
Adriosaurus_suessi
Pontosaurus
Aigialosaurus_dalmaticus
Platecarpus
Clidastes
Dinilysia_patagonica
Najash_rionegrina
Tylosaurus
Helodermoides_tuberculatus
Plotosaurus
Amphisbaena_fuliginosa
Estesia_mongoliensis
Lanthanotus_borneensis
Varanus_salvator
Varanus_exanthematicus
Varanus_acanthurus
Saniwa
Heloderma_horridum
Heloderma_suspectum
Eichstaettisaurus
Lacerta_viridis
Zonosaurus_ornatus
Cordylosaurus_subtesselatus
Elgaria_multicarinata
AMNH_FR_21444
Brachymeles_gracilis
Celestus_enneagrammus
Delma_borea
Eoxanta_lacertifrons
Palaeoxantusia_sp.
Plestiodon_fasciatus
Scincus
Trachylepis_quinquetaeniata
Sphenomorphus_solomonis
Shinisaurus_crocodilurus
Amphiglossus_splendidus
Tiliqua_scincoides
Feylinia_polylepis
Peltosaurus_granulosus
Eugongylus_rufescens
Xenosaurus_platyceps
Xenosaurus_grandis
Myrmecodaptria_microphagosa
Globaura_venusta
Aeluroscalobates_felinus
Eublepharis_macularius
Coleonyx_variegatus
Takydromus_ocellatus
Paramacellodus
Strophurus_ciliaris
Phelsuma_lineata
Rhacodactylus_auriculatus
Saltuarius_cornutus
Gonatodes_albogularis
Teratoscincus
Gekko_gecko
0.4
