# Supplementary material for: Integrated Analyses Resolve Conflicts over Squamate Reptile Phylogeny and Reveal Unexpected Placements for Fossil Taxa
Source: PLoS One. 2015 Mar 24;10(3):e0118199. doi: 10.1371/journal.pone.0118199 (PMC4372529; doi:10.1371/journal.pone.0118199)
Supplement: S47 Fig — Numbers at nodes indicate posterior probabilities. (PDF) [file pone.0118199.s049.pdf]

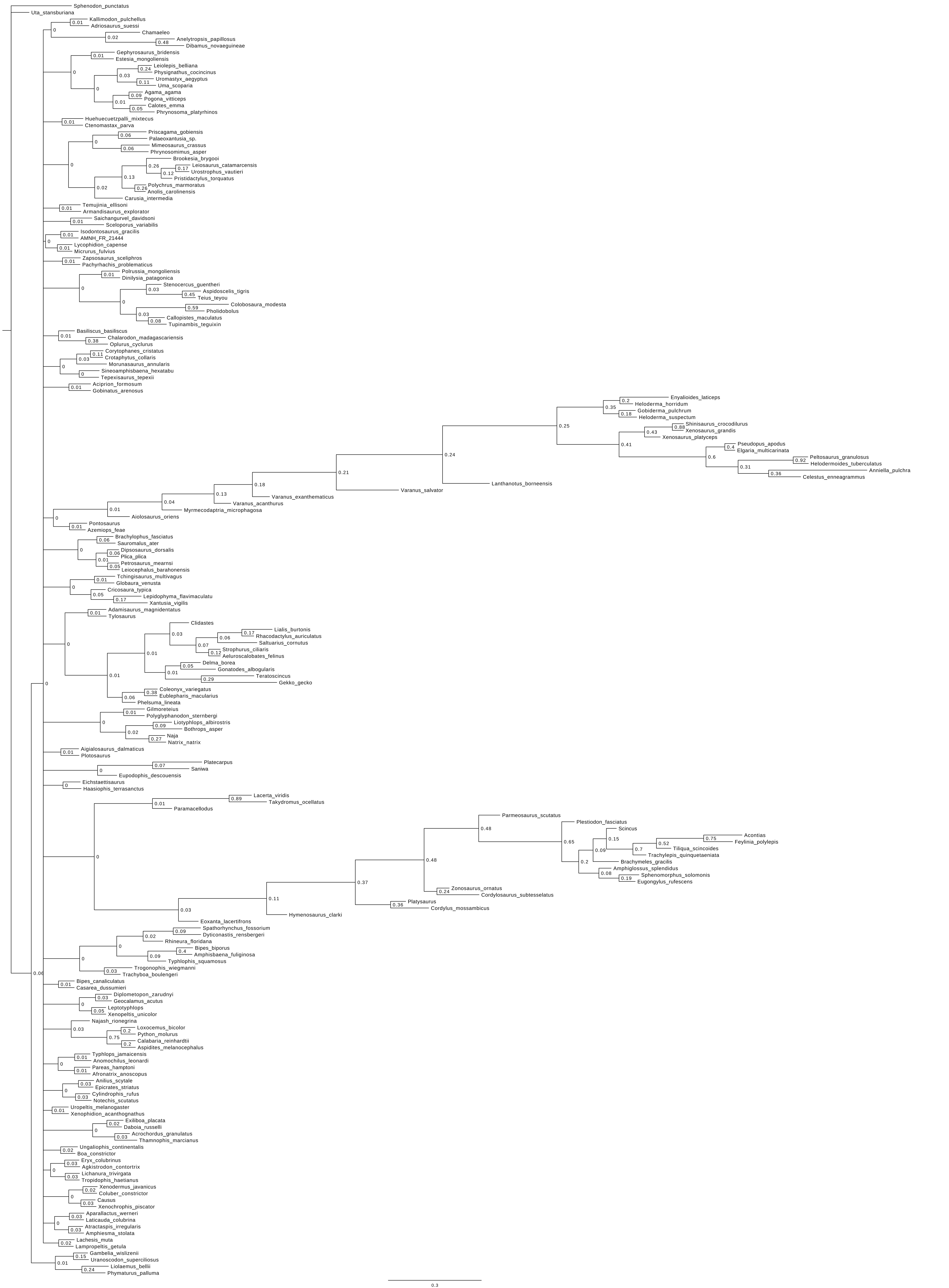

Sphenodon_punctatus
Uta_stansburiana
0.01 Kallimodon_pulchellus
Adriosaurus_suessi
0
0.02 Chamaeleo
0.48 Anelytropsis_papillosus
Dibamus_novaeguineae
0.01 Gephyrosaurus_bridensis
Estesia_mongoliensis
0
0.24 Leiolepis_belliana
0.03 Physignathus_cocincinus
0.11 Uromastyx_aegyptus
Uma_scoparia
0
0.09 Agama_agama
Pogona_vitticeps
0.01
0.05 Calotes_emma
Phrynosoma_platyrhinos
0.01 Huehuecuetzpalli_mixtecus
Ctenomastax_parva
0.06 Priscagama_gobiensis
0 Palaeoxantusia_sp.
0.06 Mimeosaurus_crassus
Phrynosomimus_asper
0
Brookesia_brygooi
0.26 Leiosaurus_catamarcensis
0.17 Urostrophus_vautieri
0.12 Pristidactylus_torquatus
0.13
0.26 Polychrus_marmoratus
0.02 Anolis_carolinensis
Carusia_intermedia
0.01 Temujinia_ellisoni
Armandisaurus_explorator
0.01 Saichangurvel_davidsoni
Sceloporus_variabilis
0.01 Isodontosaurus_gracilis
0 AMNH_FR_21444
0.01 Lycophidion_capense
Micrurus_fulvius
0.01 Zapsosaurus_sceliphros
Pachyrhachis_problematicus
0.01 Polrussia_mongoliensis
Dinilysia_patagonica
0
Stenocercus_guentheri
0.03 Aspidoscelis_tigris
0.45 Teius_teyou
0
0.59 Colobosaura_modesta
Pholidobolus
0.03 Callopistes_maculatus
0.08 Tupinambis_teguixin
Basiliscus_basiliscus
0.01 Chalarodon_madagascariensis
0.38 Oplurus_cyclurus
0.11 Corytophanes_cristatus
0.03 Crotaphytus_collaris
0 Morunasaurus_annularis
0 Sineoamphisbaena_hexatabu
Tepexisaurus_tepexii
0.01 Aciprion_formosum
Gobinatus_arenosus
0.2 Enyalioides_laticeps
0.35 Heloderma_horridum
0.18 Gobiderma_pulchrum
Heloderma_suspectum
0.25
0.88 Shinisaurus_crocodilurus
0.43 Xenosaurus_grandis
Xenosaurus_platyceps
0.41
0.4 Pseudopus_apodus
Elgaria_multicarinata
0.6
0.92 Peltosaurus_granulosus
Helodermoides_tuberculatus
0.31 Anniella_pulchra
0.36 Celestus_enneagrammus
0.24
Lanthanotus_borneensis
0.21
Varanus_salvator
0.18
Varanus_exanthematicus
0.13
Varanus_acanthurus
0.04
Myrmecodaptria_microphagosa
0.01
Aiolosaurus_oriens
0
0.01 Pontosaurus
Azemiops_feae
0.06 Brachylophus_fasciatus
Sauromalus_ater
0
0.06 Dipsosaurus_dorsalis
0.01 Plica_plica
0.05 Petrosaurus_mearnsi
Leiocephalus_barahonensis
0.01 Tchingisaurus_multivagus
Globaura_venusta
0
Cricosaura_typica
0.05 Lepidophyma_flavimaculatu
0.17 Xantusia_vigilis
0.01 Adamisaurus_magnidentatus
Tylosaurus
0
Clidastes
0.03 Lialis_burtonis
0.17 Rhacodactylus_auriculatus
0.06 Saltuarius_cornutus
0.07
0.12 Strophurus_ciliaris
Aeluroscalobates_felinus
0.01
0.05 Delma_borea
0.01 Gonatodes_albogularis
Teratoscincus
0.29 Gekko_gecko
0.01
0.38 Coleonyx_variegatus
0.06 Eublepharis_macularius
Phelsuma_lineata
0
0.01 Gilmoreteius
0 Polyglyphanodon_sternbergi
0.09 Liotyphlops_albirostris
0.02 Bothrops_asper
0.27 Naja
Natrix_natrix
0.01 Aigialosaurus_dalmaticus
Plotosaurus
0.07 Platecarpus
0 Saniwa
Eupodophis_descouensis
0 Eichstaettisaurus
Haasiophis_terrasanctus
0.89 Lacerta_viridis
0.01 Takydromus_ocellatus
Paramacellodus
Parmeosaurus_scutatus
0.48
Plestiodon_fasciatus
Scincus
0.15
0.75 Acontias
0.52 Feylinia_polylepis
0.7 Tiliqua_scincoides
0.09 Trachylepis_quinquetaeniata
0.65
0.2 Brachymeles_gracilis
Amphiglossus_splendidus
0.08 Sphenomorphus_solomonis
0.19 Eugongylus_rufescens
0
0.48
0.24 Zonosaurus_ornatus
Cordylosaurus_subtesselatus
0.37
0.36 Platysaurus
Cordylus_mossambicus
0.11
Hymenosaurus_clarki
0.03
Eoxanta_lacertifrons
0.09 Spathorhynchus_fossorium
0.02 Dyticonastis_rensbergeri
Rhineura_floridana
0
0.4 Bipes_biporus
0.09 Amphisbaena_fuliginosa
Typhlophis_squamosus
0
0.03 Trogonophis_wiegmanni
Trachyboa_boulengeri
0.06
Bipes_canaliculatus
0.01 Casarea_dussumieri
0.03 Diplometopon_zarudnyi
0 Geocalamus_acutus
0.05 Leptotyphlops
Xenopeltis_unicolor
Najash_rionegrina
0.03
0.2 Loxocemus_bicolor
0.75 Python_molurus
0.2 Calabaria_reinhardtii
Aspidites_melanocephalus
0.01 Typhlops_jamaicensis
0 Anomochilus_leonardi
0.01 Pareas_hamptoni
Afronatrix_anoscopus
0.03 Anilius_scytale
0 Epicrates_striatus
0.03 Cylindrophis_rufus
Notechis_scutatus
0.01 Uropeltis_melanogaster
Xenophidion_acanthognathus
0.02 Exiliboa_placata
0 Daboia_russelli
0.03 Acrochordus_granulatus
Thamnophis_marcianus
0.02 Ungaliophis_continentalis
Boa_constrictor
0.03 Eryx_colubrinus
0 Agkistrodon_contortrix
0.03 Lichanura_trivirgata
Tropidophis_haetianus
0.02 Xenodermus_javanicus
0 Coluber_constrictor
0.03 Causus
Xenochrophis_piscator
0.03 Aparallactus_werneri
0 Laticauda_colubrina
0.03 Atractaspis_irregularis
Amphiesma_stolata
0.02 Lachesis_muta
Lampropeltis_getula
0.15 Gambelia_wislizenii
0.01 Uranoscodon_superciliosus
Liolaemus_bellii
0.24 Phymaturus_palluma
0.3
